# Synthesis of ^18^F-difluoromethylarenes using aryl boronic acids, ethyl bromofluoroacetate and [^18^F]fluoride†
†Electronic supplementary information (ESI) available. See DOI: 10.1039/c8sc05096a


**DOI:** 10.1039/c8sc05096a

**Published:** 2019-01-29

**Authors:** Jeroen B. I. Sap, Thomas C. Wilson, Choon Wee Kee, Natan J. W. Straathof, Christopher W. am Ende, Paramita Mukherjee, Lei Zhang, Christophe Genicot, Véronique Gouverneur

**Affiliations:** a Chemistry Research Laboratory , Department of Chemistry , Oxford University , OX1 3TA Oxford , UK . Email: veronique.gouverneur@chem.ox.ac.uk ; Tel: +44 (0)1865 285002; b Pfizer Inc., Medicine Design , Eastern Point Road, Groton, Connecticut 06340, and 1 Portland Street , Cambridge , Massachusetts 02139 , USA; c Global Chemistry, UCB New Medicines , UCB Biopharma Sprl , 1420 Braine-L'Alleud , Belgium

## Abstract

Herein, we report the radiosynthesis of ^18^F-difluoromethylarenes *via* the assembly of three components, a boron reagent, ethyl bromofluoroacetate, and cyclotron-produced non-carrier added [^18^F]fluoride. The two key steps are a copper-catalysed cross-coupling reaction, and a Mn-mediated ^18^F-fluorodecarboxylation.

## Abstract 2

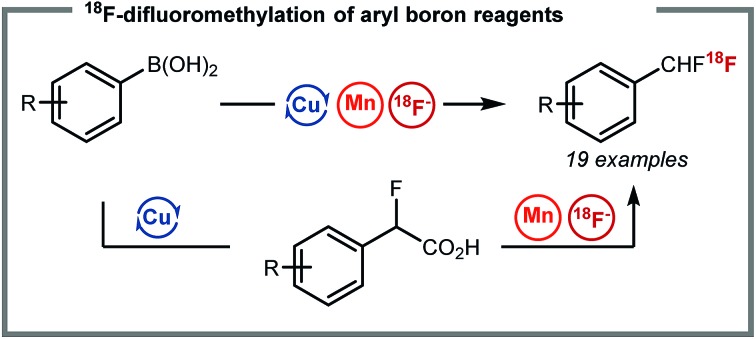
Herein, we report the radiosynthesis of ^18^F-difluoromethylarenes *via* the assembly of three components, a boron reagent, ethyl bromofluoroacetate, and cyclotron-produced non-carrier added [^18^F]fluoride.

## Introduction

**Scheme 1:**
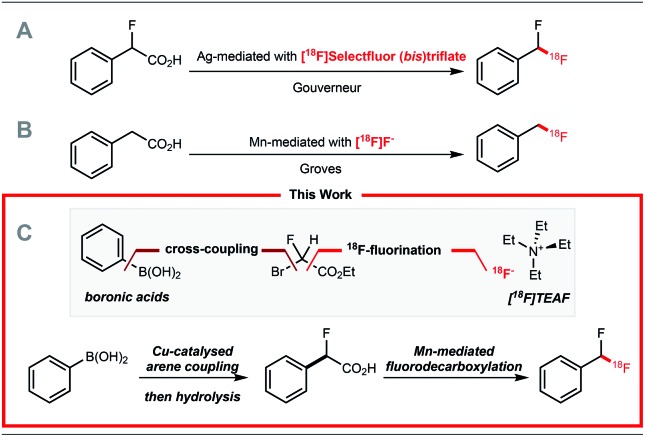
(A) Ag(i)-mediated ^18^F-fluorodecarboxylation with [^18^F]Selectfluor (bis)triflate. (B) Mn(iii)-mediated ^18^F-fluorodecarboxylation with [^18^F]fluoride towards [^18^F]ArCH_2_F. (C) Synthetic plan towards [^18^F]ArCF_2_H from boron reagents and [^18^F]fluoride.

Positron emission tomography (PET) is a molecular imaging technique that requires molecules labelled with a positron-emitting radionuclide. Fluorine-18 is a widely used positron emitting radionuclide in part due to its favourable decay properties, and the numerous clinical applications of 2-deoxy-2-[^18^F]fluoro-d-glucose, a radiopharmaceutical prepared from [^18^F]fluoride.^1^ While radiochemists have in recent years focused their efforts on methods enabling ^18^F-fluorination^2^ and ^18^F-trifluoromethylation of (hetero)arenes,^2^,^3^ ^18^F-difluoromethylation reactions have been less studied despite the importance of the CF_2_H motif^4^ in radioligand design for drug discovery programmes. In 2013, we reported a Ag(i)-mediated ^18^F-fluorodecarboxylation of 2-fluoro-2-arylacetic acids with [^18^F]Selectfluor (bis)triflate leading to [^18^F]ArCF_2_H.^5^ Subsequently, we disclosed a Ag(i)-mediated halogen exchange reaction using [^18^F]fluoride.^6^ In 2016, a multi-step method to label [^18^F]ArCF_2_H from aryl (pseudo)halides was disclosed by Ritter and co-workers.^7^ Later, Liang and co-workers demonstrated that halogen exchange of benzyl (pseudo)halides with [^18^F]fluoride followed by oxidative benzylic C–H fluorination with Selectfluor afforded [^18^F]ArCF_2_H with improved molar activity.^8^ Despite these advances, ^18^F-difluoromethylation remains a challenging problem, especially for structurally complex targets. We initially considered adapting difluoromethylation reactions operating *via* C–H functionalisation.^9^ Whilst this strategy is ideal for (hetero)arenes with innate reactivity leading to site-selective ^18^F-difluoro-methylation, substrates that are not reactive or too reactive would be unsuitable, thereby limiting applicability for radioligand synthesis. We therefore opted to develop a method using pre-functionalised aryl boron reagents; these are amenable to ^18^F-fluorination and ^18^F-trifluoromethylation,^10^ so extension to ^18^F-difluoromethylation was viewed as a valuable development. Building on our Ag(i)-mediated ^18^F-fluorodecarboxylation towards [^18^F]ArCF_2_H,^5^ a reaction requiring [^18^F]Selectfluor (bis)triflate (Scheme 1A),^11^ and on the Mn-mediated fluorodecarboxylation reported by Groves and co-workers, a reaction using [^18^F]fluoride (Scheme 1B),^12^,^13^ we envisaged that the ^18^F-fluorodecarboxylation of 2-fluoro-2-arylacetic acids with [^18^F]fluoride could afford [^18^F]ArCF_2_H. The beneficial effect of fluorine substitution on radical stabilisation would be favorable for this process.^5^,^14^ This approach would require a robust method to cross-couple the aryl boron reagent with ethyl bromofluoroacetate followed by hydrolysis to access the carboxylic acid precursor; we gave preference to a coupling methodology applying Cu-catalysis instead of Pd or Ni, a decision driven by guidelines for residual metals in (radio)pharmaceuticals.^15^ The proposed strategy therefore relies on three readily available components, the boron reagent, ethyl bromofluoroacetate, and [^18^F]fluoride (Scheme 1C).^16^

## Results and discussion

**Scheme 2:**
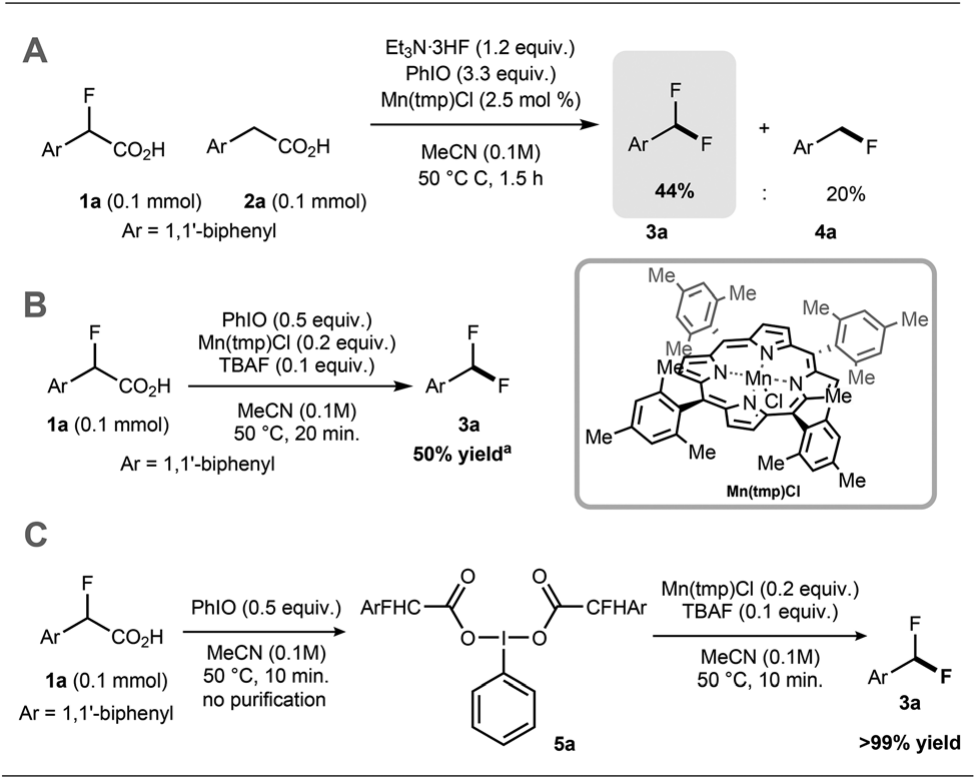
(A) Competition studies evaluating the effect of fluorine substitution on fluorodecarboxylation. (B) Reaction with sub-stoichiometric fluoride. (C) Reaction of iodine(iii) complex **5a** with sub-stoichiometric fluoride. Yields of isolated products. Mn(tmp)Cl = Mn(iii) *meso*-tetra(2,4,6-trimethylphenyl)porphyrin chloride. ^a^Yield determined by ^19^F NMR using α,α,α-trifluorotoluene as internal standard.

Preliminary experiments demonstrated that the model fluoro-substituted carboxylic acid **1a** is amenable to fluorodecarboxylation with fluoride. When an equimolar mixture of **1a** and **2a** was treated with Mn(tmp)Cl (2.5 mol%), Et_3_N·3HF (1.2 equiv.) and PhIO (3.3 equiv.) in MeCN at 50 °C, **3a** and **4a** were obtained in 44% and 20% yield, respectively. This result indicates that the fluorine-substituted precursor **1a** is more reactive than non-fluorinated **2a** towards fluorodecarboxylation (Scheme 2A). We verified that product **4a** did not undergo fluorination *via* C–H functionalisation under these conditions.^17^ When an excess of **1a** (1 equiv.) was treated with TBAF (0.1 equiv.), PhIO (0.5 equiv.) and Mn(tmp)Cl (0.2 equiv.) in MeCN, **3a** was obtained in 50% yield (determined by ^19^F NMR based on TBAF consumption) (Scheme 2B). Notably, quantitative fluoride incorporation was observed applying similar reaction conditions to the preformed hypervalent iodine complex **5a** (Scheme 2C). These preliminary data boded well for ^18^F-labeling with [^18^F]fluoride as the limiting reagent, and prompted the development of a robust protocol to convert aryl boron reagents into 2-fluoro-2-arylacetic acids.

The cross-coupling of arylboronic acids and ethyl bromofluoroacetate has been reported using an excess of boron reagent under Ni or Pd catalysis, but has not been accomplished under Cu catalysis.^18^–^22^ Initial studies reacting [1,1′-biphenyl]-4-ylboronic acid **6a** (2 equiv.) with ethyl bromofluoroacetate (1 equiv.) in the presence of 1,10-phenanthroline (**L1**, 20 mol%), CuI (20 mol%) and Cs_2_CO_3_ (2 equiv.) in dioxane (0.2 M) under N_2_ at 100 °C afforded **7a** in 7% yield (Table 1, entry 1). When 2,2′:6′,2′′-terpyridine (**L2**) was used as the ligand, the yield was significantly improved to 58% yield (Table 1, entry 2). When the stoichiometry was altered to 1 equivalent of **6a** and 2 equivalents of ethyl bromofluoroacetate in the presence of 4,4′,4′′-tri-*tert*-butyl-2,2′:6′,2′′-terpyridine (**L3**) in toluene instead of dioxane **7a** was obtained in 63% yield (Table 1, entry 3). Further optimisation increasing the concentration led to the optimal protocol consisting of treating **6a** (0.1 mmol) with ethyl bromofluoroacetate (0.2 mmol), Cs_2_CO_3_ (0.2 mmol), CuI (20 mol%) and **L3** (20 mol%) in toluene (0.4 M) at 100 °C. Under these reaction conditions, **7a** was isolated in 82% yield (Table 1, entry 4). A one-pot sequence involving cross-coupling followed by hydrolysis with MeOH and aqueous K_2_CO_3_ afforded **8a** isolated in 75% yield (Table 1, entry 5). In the absence of ligand and/or copper source (Table 1, entries 6, 7), no product formation was observed. Furthermore, no reaction was observed with CuCl_2_ (Table 1, entry 8), or when the reaction solvent was DMF or DMSO (Table 1, entry 9).

**Table 1:** Optimisation of the Cu-catalysed cross-coupling of aryl boronic acid **6a** with ethyl bromofluoroacetate towards ester **7a** and the corresponding carboxylic acid **8a**^*a*^

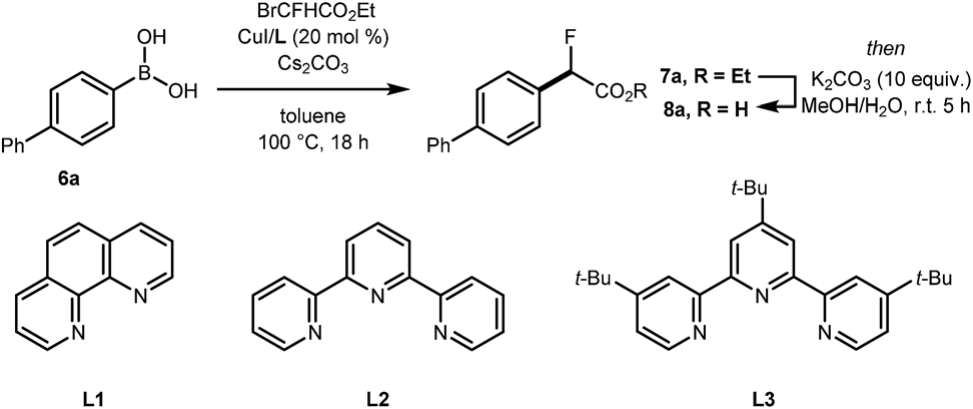					
Entry	Solvent	Cu-source	Ligand	Product	Yield^*b*^
1^*c*^	Dioxane (0.2 M)	CuI	**L1**	**7a**	7%
2^*c*^	Dioxane (0.2 M)	CuI	**L2**	**7a**	58%
3	Toluene (0.2 M)	CuI	**L3**	**7a**	63%
4^*d*^	Toluene (0.4 M)	CuI	**L3**	**7a**	82%^*e*^
5^*d*^	Toluene (0.4 M)	CuI	**L3**	**8a**	75%^*e*^ ^,^^*f*^
6^*d*^	Toluene (0.4 M)	CuI	—	**7a**	0%
7^*d*^	Toluene (0.4 M)	—	—	**7a**	0%
8^*d*^	Toluene (0.4 M)	CuCl_2_	**L2**	**7a**	0%
9^*d*^	DMF or DMSO (0.2 M)	CuI	**L3**	**7a**	0%

^*a*^Screening reactions performed on 0.1 mmol scale.

^*b*^Yield determined by ^19^F-NMR using α,α,α-trifluorotoluene as internal standard.

^*c*^2 equiv. of **6a** and 1 equiv. of ethyl bromofluoroacetate.

^*d*^1 equiv. of **6a**, and 2 equiv. of ethyl bromofluoroacetate.

^*e*^Yield of isolated product.

^*f*^One-pot procedure towards **8a**.

**Scheme 3:**
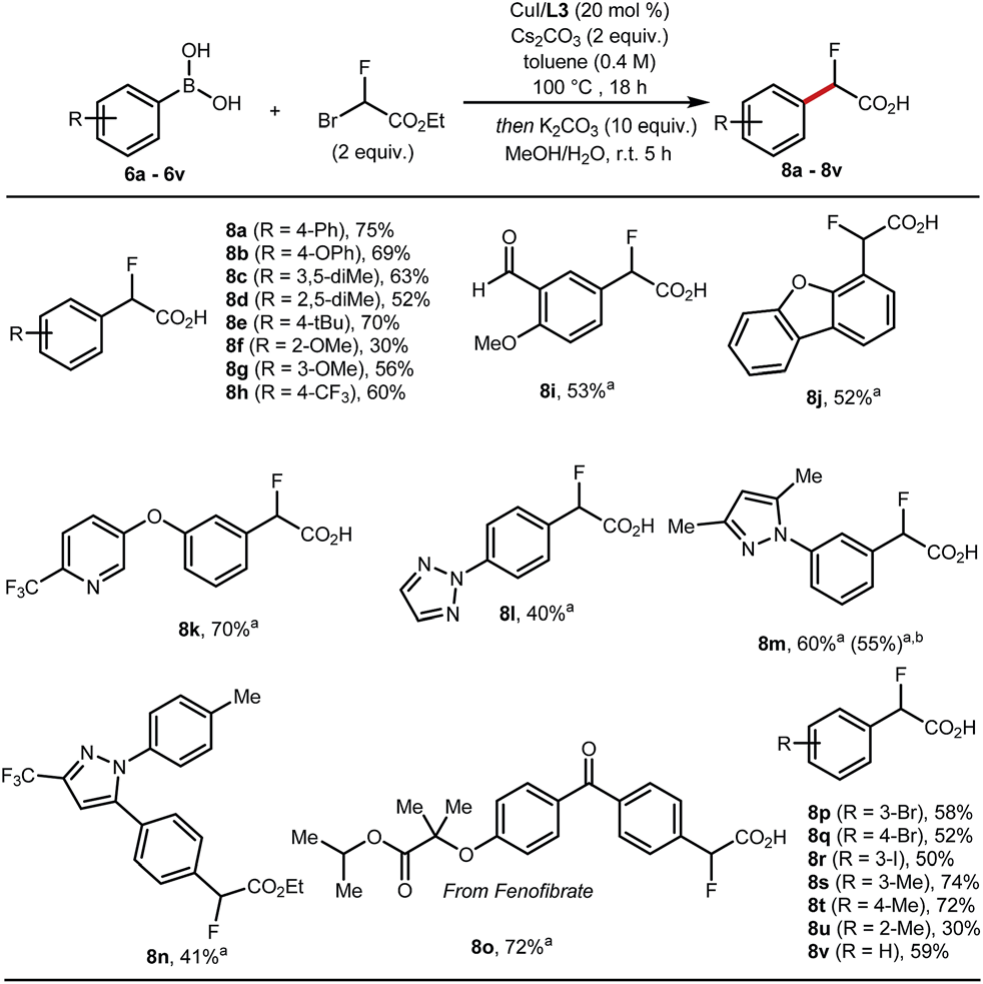
Scope of Cu-catalysed cross-coupling. The reactions were performed on a 0.3 mmol scale. Conditions: CuI (20 mol%), **L3** (20 mol%), aryl boronic acid (1 equiv.), ethyl bromofluoroacetate (2 equiv.), Cs_2_CO_3_ (2 equiv.), toluene (0.4 M) at 100 °C for 18 h then one-pot hydrolysis with K_2_CO_3_ (10 equiv.), MeOH/H_2_O (1 : 1), 5 h. ^a^Hydrolysis performed as a subsequent step with K_2_CO_3_ (5 equiv.). ^b^Reaction run on 5 mmol scale. All yields are of isolated products.

These optimised conditions gave access to a range of 2-fluoro-2-arylacetic acids (Scheme 3). The reaction is broad in scope and tolerates various functional groups, for example alkyl **8c–8e** and **8s–8u**, alkoxy **8f**, **8g**, trifluoromethyl **8h**, bromo **8p**, **8q**, iodo **8r**, and aldehyde **8i** all performed well. Substrates featuring heterocycles such as dibenzofuran **8j**, pyridine **8k**, triazole **8l**, and pyrazoles **8m**, **8n** are also suitable coupling partners applying our optimised protocol affording the desired products in 40% to 70% yield. Additionally, this cross-coupling chemistry afforded **8o**, a derivative of fenofibrate, in 72% yield. Finally, the reaction was amenable to scale-up to 5 mmol (Scheme 3, **8m**).

The key ^18^F-fluorodecarboxylation step was studied next (Table 2). We started our investigation applying protocol A that consists of reacting in one-pot **8b** (0.11 mmol) with PhIO (0.33 mmol), Mn(tmp)Cl (2 mg) and [^18^F]TEAF (20–30 MBq) in MeCN (600 μL) at 50 °C; this protocol led to only traces of **[^18^F]3b** (Table 2, entry 1). When the loading of PhIO (0.02 mmol) and MeCN (300 μL) was reduced, **[^18^F]3b** was obtained in 6% ± 1% radiochemical conversion (RCC) (Table 2, entry 2). Similar results were obtained in DMF (Table 2, entry 3). Reducing the stoichiometry of **8b** led to a significant increase in RCC (22% ± 7%) (Table 2, entry 4). When applying protocol B which consists of mixing **8b** with PhIO, a process generating complex **5b**, prior to the addition of Mn(tmp)Cl (2 mg) and [^18^F]TEAF (20–30 MBq) and DMF (300 μL), a drastic improvement was observed, and **[^18^F]3b** was obtained in 40% ± 10% RCC (*n* = 10) (Table 2, entry 5). When the reaction was run at 100 °C, the formation of **[^18^F]3b** was not observed (Table 2, entry 6). No ^18^F-labelled product was obtained when Mn(tmp)OTs was used as catalyst, or in the absence of Mn(tmp)Cl (Table 2, entries 7 and 8).

**Table 2:** Optimisation studies for the [^18^F]fluorodecarboxylation of **8b**

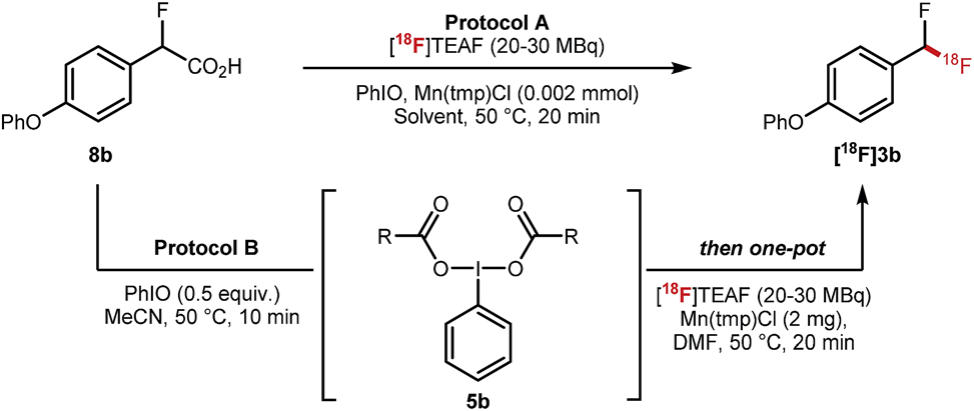					
Entry	Starting material (mmol)	Protocol	Solvent	PhIO (mmol)	RCC^*a*^ ^,^^*b*^ (*n* = 2)
1	**8b** (0.11)	A	MeCN^*c*^	0.33	3% ± 1%
2	**8b** (0.11)	A	MeCN^*d*^	0.02	6% ± 1%
3	**8b** (0.11)	A	DMF^*d*^	0.02	7% ± 2%
4	**8b** (0.055)	A	DMF^*d*^ ^,^^*e*^	0.02	22% ± 7%
**5**	**5b** **(0.014)**	**B**	**DMF** ^*d*^ ^**,**^ ^*e*^	**—**	**40% ± 10%** ^*f*^
6	**5b** (0.014)	B	DMF^*d*^ ^,^^*e*^	—	0% ± 0%^*g*^
7	**8b** (0.014)	A	MeCN^*d*^	0.02	0% ± 0%^*h*^
8	**5b** (0.014)	B	DMF^*d*^ ^,^^*e*^	—	0% ± 0%^*i*^

^*a*^Radiochemical conversion.

^*b*^ *n* = number of reactions.

^*c*^600 μL of MeCN.

^*d*^300 μL of MeCN.

^*e*^MeCN removed at 100 °C after dispensing [^18^F]TEAF.

^*f*^(*n* = 10).

^*g*^Reaction temperature = 100 °C.

^*h*^Catalyst is Mn(tmp)OTs.

^*i*^No Mn Catalyst.

**Scheme 4:**
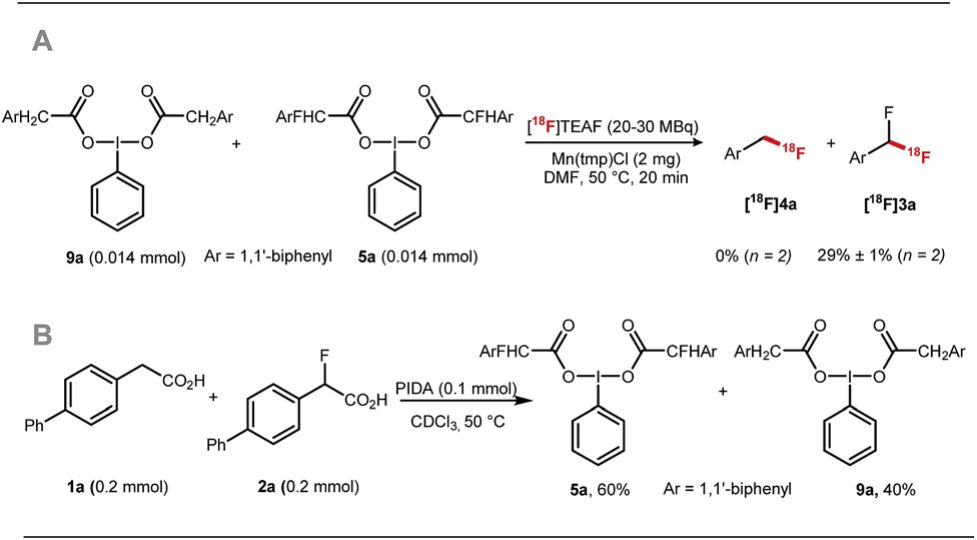
(A) Competition experiment subjecting equimolar amount of **9a** and **5a** to [^18^F]fluorodecarboxylation. (B) Competition experiment reacting equimolar amount of **1a** and **3a** with PIDA.

The fluorine substituent is advantageous for ^18^F-fluorodecarboxylation as demonstrated with a competition experiment subjecting equimolar amount of pre-formed hypervalent iodine(iii) complexes **9a** and **5a** to ^18^F-fluorination with [^18^F]TEAF, Mn(tmp)Cl at 50 °C in DMF. Difluoromethylarene **[^18^F]3a** was the only product observed in the crude reaction mixture (Scheme 4A). Furthermore, an additional competition experiment showed that the iodine(iii) complex **5a** is formed preferentially to **9a** (Scheme 4B). Fluorine substitution therefore facilitates the two steps of the process leading to fluorodecarboxylation.

**Scheme 5:**
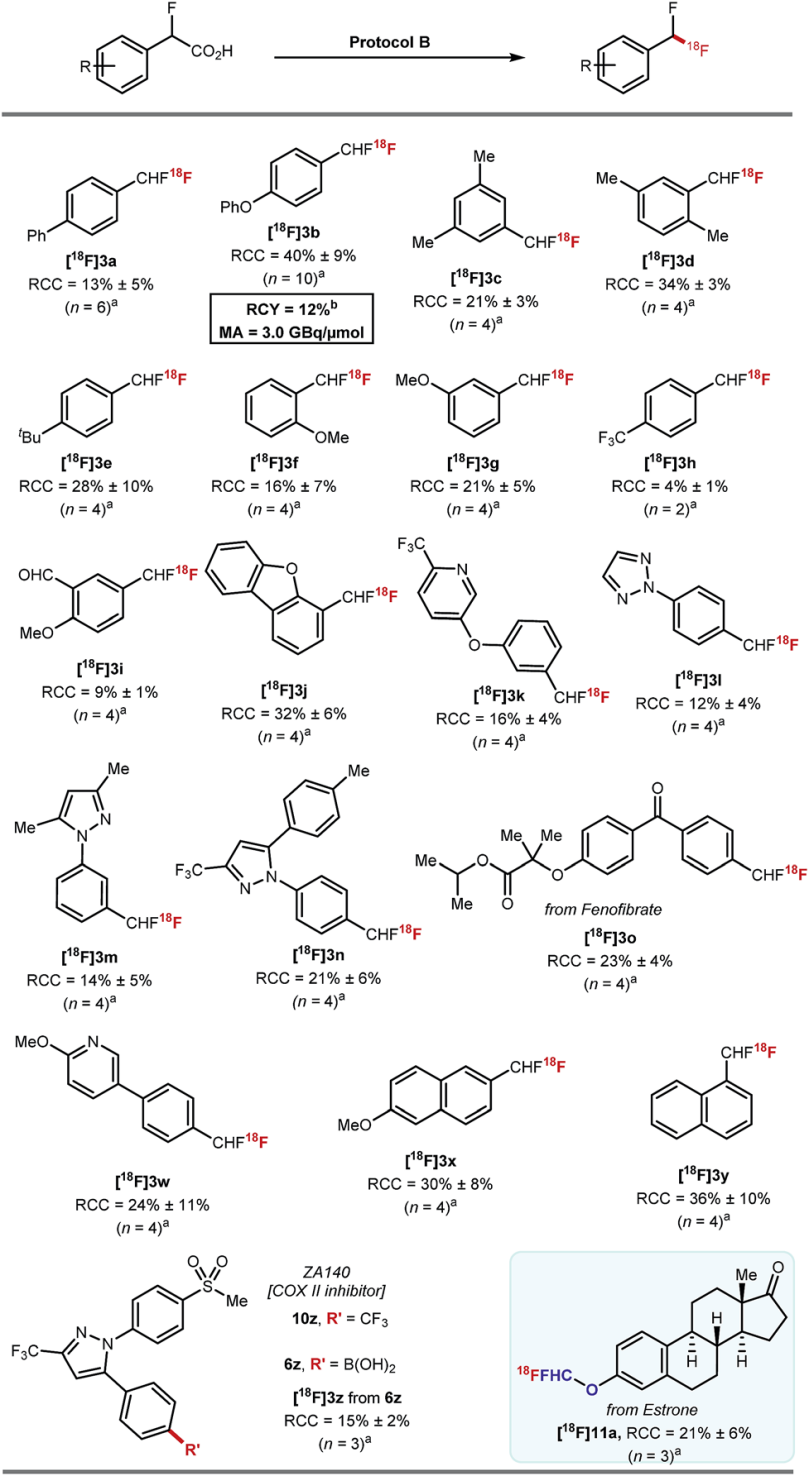
Scope of [^18^F]fluorodecarboxylation applying protocol B: ^a^ArCHFCO_2_H (0.028 mmol), PhIO (0.5 equiv.), MeCN (1 mL), 50 °C, 10 min then addition of [^18^F]TEAF (20–30 MBq) Mn(tmp)Cl (2 mg), DMF (300 μL), 50 °C, 20 min. ^b^ArCHFCO_2_H (0.014 mmol), PhIO (0.5 equiv.), MeCN (1 mL), 50 °C, 10 min then addition of [^18^F]Mn(tmp)F (841 MBq) DCE (300 μL), 60 °C, 20 min.

Protocol B was applied to a selection of arenes using 20–30 MBq of [^18^F]fluoride (Scheme 5). Ether, alkyl, aldehyde, ketone, pyridine, triazole, pyrazole, dibenzofuran motifs were all tolerated. The highest RCCs were obtained for electron rich arenes. **[^18^F]3o** derived from a boronic acid analogue of fenofibrate was successfully labelled in 23% ± 4% (*n* = 4). The boronic acid derivative of the COX-II inhibitor ZA140 **6z** was transformed into the labelled difluoromethylated product **[^18^F]3z** in 15% ± 2% RCC (*n* = 3).

The ^18^F-fluorodecarboxylation of **5b** performed with 841 MBq of [^18^F]fluoride required further optimisation. For this experiment, [^18^F]fluoride was captured on an anion exchange cartridge then eluted using a solution of Mn(tmp)Cl in methanol, resulting in 85% ^18^F-recovery. Lowering the starting material stoichiometry to 0.007 mmol of **5b** and changing the solvent from DMF to DCE afforded the cartridge-purified **[^18^F]3b** in a decay corrected RCY of 12% and a molar activity of 3.0 GBq μmol^–1^ in a total synthesis time of 30 minutes.^23^

Pleasingly, ^18^F-fluorodecarboxylation also enabled access to the [^18^F]ArOCF_2_H motif. The only known route to label this motif was reported by our group, and required a multi-step synthesis of the ArOCHFCl precursors which were themselves prepared from ArOCHFCO_2_H.^24^ The reaction of estrone (1.0 equiv.) with ethyl bromofluoroacetate (1.5 equiv.) and K_2_CO_3_ (2.5 equiv.) in DMF (2 mL) at room temperature followed by a subsequent hydrolysis with aqueous NaOH (2.5 equiv.) in 1 : 1 H_2_O/Et_2_O afforded the precursor required for fluorodecarboxylation. ^18^F-labelling applying protocol B afforded **[^18^F]11a** in 21% ± 6% RCC (*n* = 3).

## Conclusions

In summary, a novel method was developed to transform aryl boronic acids to [^18^F]ArCF_2_H. Prior to labelling, the cross-coupling with ethyl bromofluoroacetate was accomplished under Cu catalysis followed by *in situ* hydrolysis. The radioisotope ^18^F is then introduced in the last step applying a Mn-mediated fluorodecarboxylation with readily available [^18^F]fluoride. This study has unveiled three key features for this last transformation. Firstly, the fluorine substituent on the carboxylic acid precursor is advantageous for fluorodecarboxylation; secondly, the benefit of preforming the hypervalent iodine complex prior to ^18^F-fluorination; and thirdly, we have established that Mn-mediated fluorodecarboxylation enables access to [^18^F]ArOCF_2_H in addition to [^18^F]ArCF_2_H.

## Conflicts of interest

There are no conflicts to declare.

## Supplementary Material

Supplementary information

## References

[cit1] Ametamey S. M., Honer M., Schubiger P. A. (2008). Chem. Rev..

[cit2] Miller P. W., Long N. J., Vilar R., Gee A. D. (2008). Angew. Chem., Int. Ed..

[cit3] Huiban M., Tredwell M., Mizuta S., Wan Z., Zhang X., Collier T. L., Gouverneur V., Passchier J. (2013). Nat. Chem..

[cit4] Meanwell N. A. (2011). J. Med. Chem..

[cit5] Mizuta S., Stenhagen I. S., O'Duill M., Wolstenhulme J., Kirjavainen A. K., Forsback S. J., Tredwell M., Sandford G., Moore P. R., Huiban M., Luthra S. K., Passchier J., Solin O., Gouverneur V. (2013). Org. Lett..

[cit6] Verhoog S., Pfeifer L., Khotavivattana T., Calderwood S., Collier T. L., Wheelhouse K., Tredwell M., Gouverneur V. (2016). Synlett.

[cit7] Shi H., Braun A., Wang L., Liang S. H., Vasdev N., Ritter T. (2016). Angew. Chem., Int. Ed..

[cit8] Yuan G., Wang F., Stephenson N. A., Wang L., Rotstein B. H., Vasdev N., Tang P., Liang S. H. (2017). Chem. Commun..

[cit9] Fujiwara Y., Dixon J. A., Rodriguez R. A., Baxter R. D., Dixon D. D., Collins M. R., Blackmond D. G., Baran P. S. (2012). J. Am. Chem. Soc..

[cit10] Wilson T. C., Cailly T., Gouverneur V. (2018). Chem. Soc. Rev..

[cit11] Teare H., Robins E. G., Kirjavainen A., Forsback S., Sandford G., Solin O., Luthra S. K., Gouverneur V. (2010). Angew. Chem., Int. Ed..

[cit12] Huang X., Liu W., Hooker J. M., Groves J. T. (2015). Angew. Chem., Int. Ed..

[cit13] Huang X., Liu W., Ren H., Neelamegam R., Hooker J. M., Groves J. T. (2014). J. Am. Chem. Soc..

[cit14] Dolbier W. R. (1996). Chem. Rev..

[cit15] Source: http://www.ich.org/products/guidelines/quality/article/quality-guidelines.html, accessed on 20/09/18.

[cit16] Our attempts to assemble one-pot the aryl boron reagent, ethyl bromofluoroacetate and [^18^F]fluoride were not fruitful. Details in ESI.

[cit17] See the ESI.

[cit18] Wu Y., Zhang H.-R., Cao Y.-X., Lan Q., Wang X.-S. (2016). Org. Lett..

[cit19] Guo C., Yue X., Qing F. L. (2010). Synthesis.

[cit20] Su Y. M., Feng G. S., Wang Z. Y., Lan Q., Wang X. S. (2015). Angew. Chem., Int. Ed..

[cit21] Xia T., He L., Liu Y. A., Hartwig J. F., Liao X. (2017). Org. Lett..

[cit22] Fahandej-Sadi A., Lundgren R. J. (2017). Synlett.

[cit23] All radiochemical yields (RCYs) are decay corrected

[cit24] Khotavivattana T., Verhoog S., Tredwell M., Pfeifer L., Calderwood S., Wheelhouse K., Collier T. L., Gouverneur V. (2015). Angew. Chem., Int. Ed..

