# Supplementary material for: Synthesis of 18F-difluoromethylarenes using aryl boronic acids, ethyl bromofluoroacetate and [18F]fluoride
Source: Chem Sci. 2019 Jan 29;10(11):3237–41. doi: 10.1039/c8sc05096a (PMC6429591; doi:10.1039/c8sc05096a)
Supplement: Supplementary file 1 [file SC-010-C8SC05096A-s001.pdf]

Electronic Supplementary Material (ESI) for Chemical Science.
This journal is © The Royal Society of Chemistry 2019

# Supporting Information

## Synthesis of $^{18}$F-Difluoromethylarenes Using Aryl Boronic Acids, Ethyl bromofluoroacetate and [$^{18}$F]Fluoride

Jeroen B. I. Sap,[a] Thomas C. Wilson,[a] Choon Wee Kee,[a] Natan J. W. Straathof,[a] Christopher W. amEnde,[b] Paramita Mukherjee,[b] Lei Zhang,[b] Christophe Genicot[c] and Véronique Gouverneur*[a]

[a]*Chemistry Research Laboratory, Department of Chemistry, Oxford University, OX1 3TA (UK). E-mail: veronique.gouverneur@chem.ox.ac.uk; Fax: +44 (0)1865 285002*
[b]*Pfizer Inc., Medicine Design, Eastern Point Road, Groton, Connecticut 06340, and 1 Portland Street, Cambridge, Massachusetts 02139 (USA)*
[c]*Global Chemistry, UCB New Medicines, UCB Biopharma sprl, 1420 Braine-L'Alleud, (Belgium)*

## Table of Contents

# Contents

S1: General Information ........ 2
S2: Copper cross coupling reaction optimisation ........ 3
S3: Preliminary Fluorodecarboxylation Experiments ........ 4
S4: GC-Impurity Analysis of Manganese-Mediated Decarboxylative Fluorination ........ 6
S5: Synthetic Procedures and Characterisation of Compounds ........ 7
S6: Radiochemistry ........ 45

## S1: General Information

All chemicals purchased from commercial sources were used without further purification prior to use. All NMR spectra were recorded at a temperature of 298.15 K on Bruker AV400, or AV500 spectrometers. Appropriate deuterated solvents were used as an internal reference depending on the compound measured. All corresponding compound peaks were referenced to the residual solvent peak and reported in parts per million (ppm) using the Bruker internal referencing procedure (edlock). $^{19}F$ NMR spectral peaks were referenced to $CFCl_3$. All coupling constants are denoted as *J* and reported in Hz. The following abbreviations were used to define peak multiplicities s = singlet, d = doublet, t = triplet, q = quartet, pent = pentet, sept = septet, br = broad, m = multiplet. All NMR spectra were processed using Mestrenova 11.0 software. Mass spectra were recorded on Micromass GCT (CI) or Autospec-oaTof instruments. IR spectra were recorded as thin films on Bruker Tensor 27 FT-IR spectrometer. IR absorption are reported in wavenumbers ($cm^{-1}$). All reactions which required anhydrous conditions were carried out in flame-dried glassware under an inert atmosphere of nitrogen or argon. When dry solvents were used these were purified *a priori* on an alumina column using standard procedures. All reactions were monitored by TLC using Merck Kieselgel 60 F254 plates. High resolution mass spectra (HRMS, m/z) were recorded on a Bruker MicroTOF spectrometer using negative or positive electrospray ionization (ESI). Melting point measurements were recorded using a Griffin melting point apparatus.

# S2: Optimisation of the Cu-catalysed cross coupling reaction

## General Screening Procedure:

To a 4 mL vial containing a magnetic stir bar was added ligand (if solid), boronic acid, copper source and base. The vial was then evacuated and refilled with $N_2$ three times. Solvent (0.5 mL) and ligand (if liquid) were then added before heating the reaction mixture to 100 °C and stirring for 18 hours. The resulting solution was cooled to 0 °C before 0.1 mmol of $PhCF_3$ was added as an internal standard, the corresponding sample was then diluted with $CDCl_3$. Yields were calculated by quantitative $^{19}F$ NMR and referenced to $PhCF_3$. **Note:** All screening reactions were run on a 0.1 mmol scale, isolations were performed on 0.3 mmol scale and in some cases scaled up to illustrate scalability.

**Table 1.** Optimisation of Cu-catalysed cross-coupling of (hetero)aryl boronic acids with ethyl 2-bromo-2-fluoroacetate towards 2-fluoro-2-(hetero)arylacetic acids.

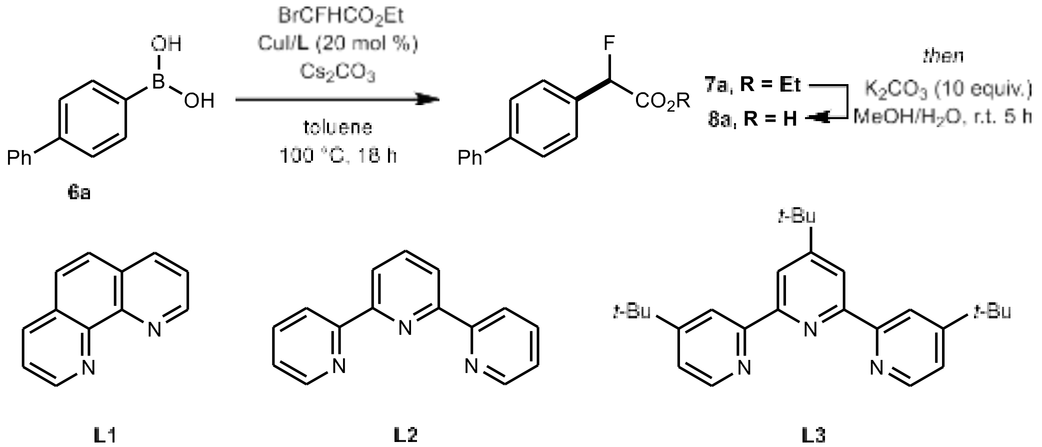


| Entry | Solvent | Cu-Source | Ligand | Product | Yield[a] |
|---|---|---|---|---|---|
| 1[b] | Dioxane (0.2 M) | CuI | L1 | **7a** | 7% |
| 2[b] | Dioxane (0.2 M) | CuI | L2 | **7a** | 58% |
| 3 | Toluene (0.2 M) | CuI | L3 | **7a** | 63% |
| 4[c] | Toluene (0.4 M) | CuI | L3 | **7a** | 82%[d] |
| 5[c] | Toluene (0.4 M) | CuI | L3 | **8a** | 75%[d,e] |
| 6[c] | Toluene (0.4 M) | CuI | - | **7a** | 0% |
| 7[c] | Toluene (0.4 M) | - | - | **7a** | 0% |
| 8[c] | Toluene (0.4 M) | $CuCl_2$ | L2 | **7a** | 0% |
| 9[c] | DMF or DMSO (0.2 M) | CuI | L3 | **7a** | 0% |

Screening reactions performed on 0.1 mmol scale. [a]Yield determined by $^{19}F$-NMR using $PhCF_3$ as internal standard. [b]Reactions with 2 equiv. of **6a** and 1 equiv. of 2-bromo-2-fluoroacetate. [b]Reactions with 1 equiv. of **6a** and 2 equiv. of 2-bromo-2-fluoroacetate [d]Yield of isolated product. [e]One-pot procedure.

## S3: Preliminary Fluorodecarboxylation Experiments

**A. Competition studies evaluating the effect of fluorine substitution on fluorodecarboxylation.**

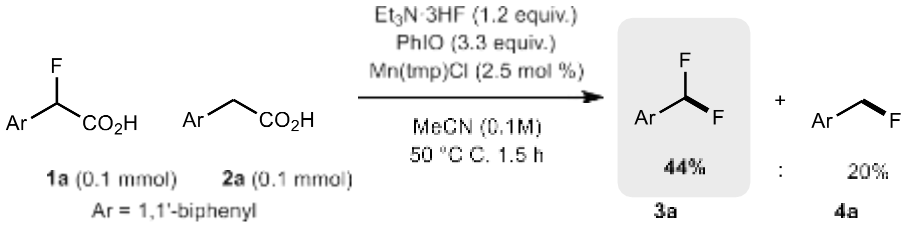


An oven-dried, 5 mL Schlenk flask equipped with a stir bar was placed under an atmosphere of $N_2$. Mn(tmp)Cl Catalyst (2.2 mg, 0.0025 mmol, 2.5 mol%), 2-([1,1'-biphenyl]-4-yl)-2-fluoroacetic acid (23.0 mg, 0.1 mmol), 2-([1,1'-biphenyl]-4-yl)acetic acid (21.2 mg, 0.1 mmol, 1 equiv.), $Et_3N$·3HF (200 µL, 0.12 mmol, 1.2 equiv.) were then added, followed by 1.0 mL of MeCN. The reaction mixture was then heated to 50 °C. Under a stream of $N_2$, iodosylbenzene (73 mg, 0.33 mmol, 3.3 equiv.) was added slowly to the reaction mixture in solid form over a period of 1.5 hours. After the addition of iodosylbenzene, the solution was cooled to r.t, 0.1 mmol of $PhCF_3$ was added as internal standard and conversion was measured by $^{19}F$ NMR.

**B. Reaction with sub-stoichiometric fluoride.**

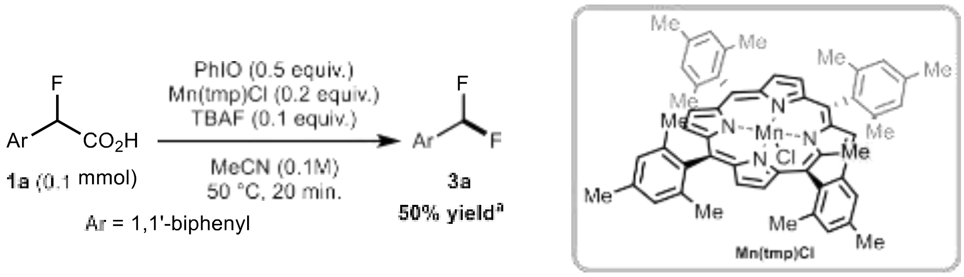


An oven-dried, 5 mL Schlenk flask equipped with a stir bar was placed under an atmosphere of $N_2$. Mn(tmp)Cl Catalyst (2.0 mg, 0.0020 mmol, 2 mol%), 2-([1,1'-biphenyl]-4-yl)-2-fluoroacetic acid, (23.0 mg, 0.1 mmol) TBAF (1M in THF) (10 µL, 0.01 mmol, 0.1 equiv.) were then added, followed by 1.0 mL of MeCN. The reaction mixture was then heated to 50 °C. Under a stream of $N_2$, iodosylbenzene (11.1 mg, 0.05 mmol, 0.5 equiv.) was added slowly to the reaction mixture in solid form over a period of 20 minutes. After the addition of iodosylbenzene, the solution was cooled to r.t, diluted with $H_2O$ (5 mL) and extracted with EtOAc (3 x 5 mL). The organic extracts was dried over $MgSO_4$ and concentrated under vacuum, prior to silica gel column chromatography.

**C. Reaction of iodine(III) complex 5a with sub-stoichiometric fluoride.**

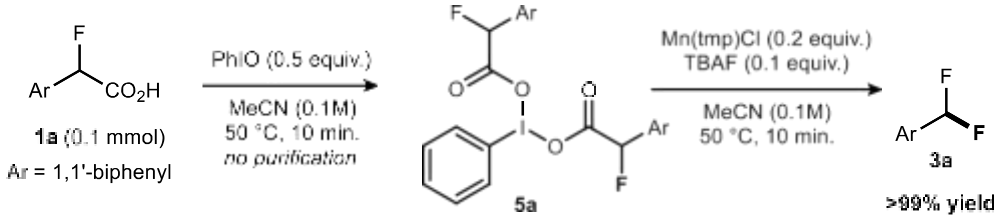


2-([1,1'-biphenyl]-4-yl)-2-fluoroacetic acid (0.2 mmol, 10 equiv.), PhIO (0.1 mmol, 5 equiv.) were added to a 4 mL vial containing a stir magnetic stir bar. $d_3$-$CH_3CN$ (2 mL) was then added to the reaction mixture and the mixture was stirred for 10 minutes at 50 °C. The solvent removed under vacuum. The residue was redissolved in $d_3$-$CH_3CN$ (2 mL). Mn(tmp)Cl (0.04 mmol, 2 equiv.) and TBAF (0.02 mmol, 1 equiv.) were then added to the reaction mixture, which was subsequently stirred for a further 10 minutes before cooling to 0 °C. 0.1 mmol of $PhCF_3$ was then added as internal standard. The yield was determined by quantitative $^{19}$F NMR (**99 %**, based on TBAF).

## S4: GC-Impurity Analysis of Manganese-Mediated Decarboxylative Fluorination

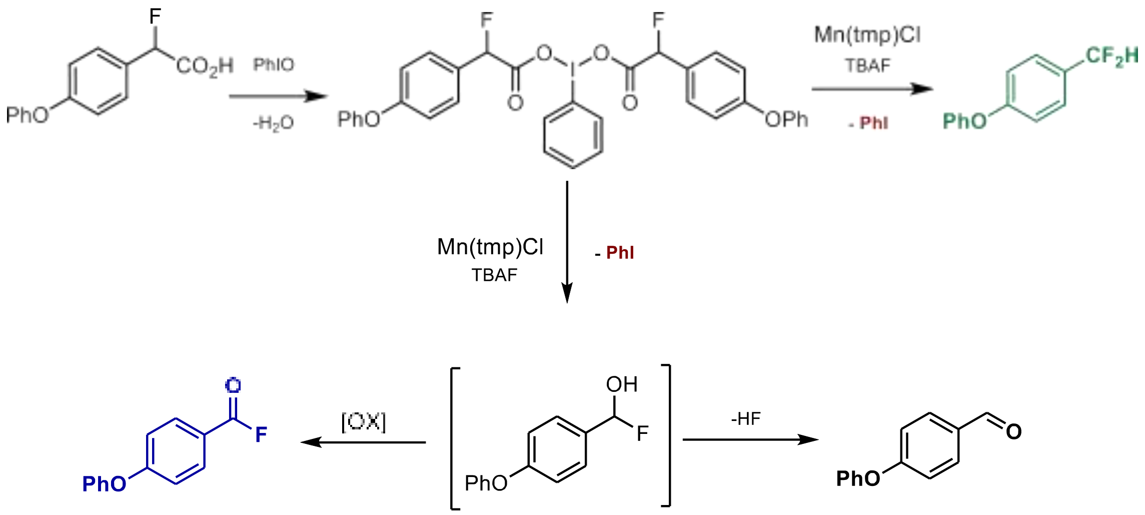


**Scheme SI-1:** GC-MS impurity analysis of manganese-mediated decarboxylative fluorination. For competitive hydroxylation in Manganese catalysed C-H fluorination see reference.[1]

TBAF (1 mmol, 1 equiv.,1.0 M in THF) and anhydrous $CH_3CN$ (2 mL) were added to a vial containing a magnetic stir bar. 2-fluoro-2-(4-phenoxyphenyl)acetic acid (1 mmol, 10 equiv.) and PhIO (5 mmol, 5 equiv.) were added to the solution (Note: PhIO dissolves as it reacts with 2-fluoro-2-(4-phenoxyphenyl)acetic acid). The mixture was stirred for 2 minutes at 50 °C before Mn(tmp)Cl (2 mmol, 2 equiv.) was added to the solution. The mixture was stirred for another 18 minutes at 50 °C before it was cooled to r.t and analysed by GC-MS (LRMS) to obtain an impurity profile, the products obtained are outlined (**Scheme SI-1**).

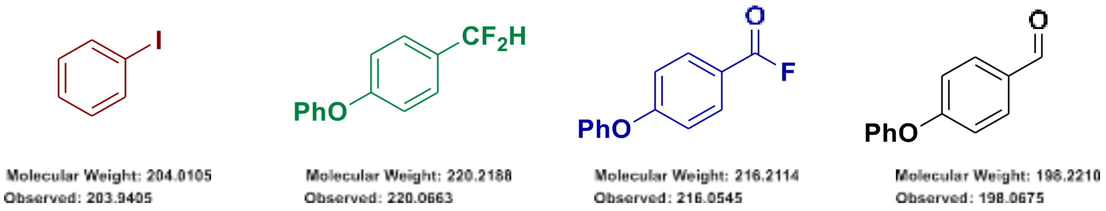

## S5: Synthetic Procedures and Characterisation of Compounds

### General Procedure (GP) 1: Synthesis of α-aryl-α-fluoroacetic acids.

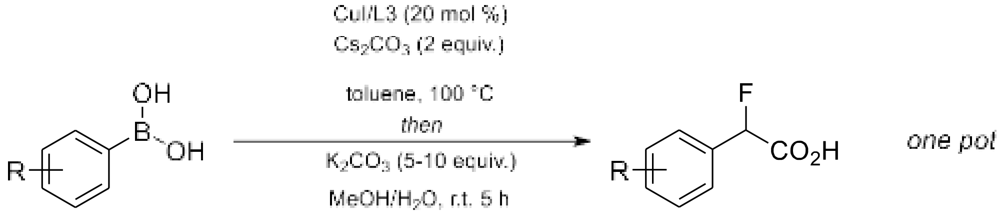


To a flame-dried pear-shaped round bottomed flask were added CuI (20 mol%), 4,4',4''-Tri-*tert*-butyl-2,2':6',2''-terpyridine (20 mol%), boronic acid (1 equiv.) and $Cs_2CO_3$ (2 equiv.), followed by argon degassed toluene (1.5 mL). The reaction mixture is stirred for 18 hours at 100 °C. The reaction mixture is cooled to r.t, solvent removed under reduced pressure. The resulting suspension was then dissolved in a 2:1 MeOH and aqueous $K_2CO_3$ (10 equiv.) mixture before it was left to stir at r.t until all the ester had been consumed (determined by TLC). MeOH was then removed under reduced pressure. The aqueous suspension was then washed with $Et_2O$ (3 x 10 mL), acidified using HCl (5 M) to pH = 2. The aqueous layer was then extracted with $Et_2O$ (3 x 10 mL) and washed with Brine (2 x 10 mL). The organic extracts were then dried over $MgSO_4$ and concentrated under vacuum and washed with pentane, resulting in an off-white solid. The resulting crude 2-fluoro-2-phenylacetic acids were then subjected to column chromatography (gradient of hexane/ethyl acetate with acetic acid (1%) to ethyl acetate/MeOH with acetic acid (1%)). *Note: Alternatively, the α-(hetero)aryl-α-fluoroacetates can be purified via column chromatography and then hydrolysed without subsequent column chromatography of the carboxylic acid (GP 2).*

### General Procedure (GP) 2: Synthesis of α-(hetero)aryl-α-fluoroacetate and subsequent hydrolysis

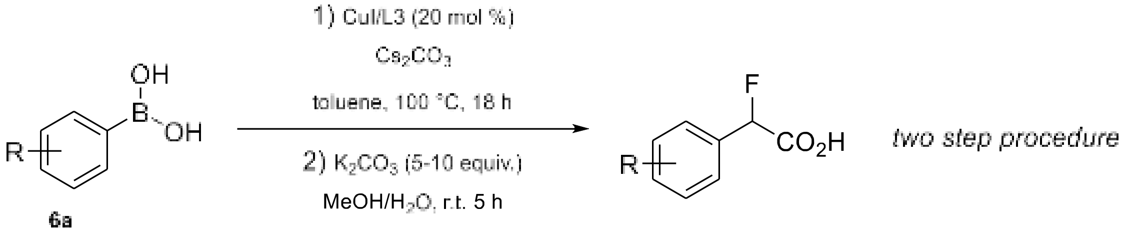


6a

To a flame-dried pear-shaped round bottomed flask were added CuI (20 mol%), 4,4',4''-Tri-*tert*-butyl-2,2':6',2''-terpyridine (20 mol%), boronic acid (1 equiv.) and $Cs_2CO_3$ (2 equiv.).

followed by argon degassed toluene (1.5 mL). The reaction mixture is stirred for 18 hours at 100 °C. The mixture is cooled to r.t, solvent removed under reduced pressure. The residue is then diluted with EtOAc and filtered through a plug of celite. EtOAc is then removed under reduced pressure. The resulting oil is purified by silica gel chromatography (EtOAc/Hexane), resulting in a clear colourless oil. This oil was then dissolved in a 2:1 MeOH and aqueous $K_2CO_3$ (5 equiv.) mixture before it was left to stir at r.t until all the ester had been consumed (determined by TLC). The resulting mixture was then acidified using HCl (5 M) to pH = 2. The aqueous layer was then extracted with $Et_2O$ (3 x 10 mL) and washed with Brine (2 x 10 mL). The organic extracts were dried using $MgSO_4$ and were then concentrated under vacuum and washed with pentane.

**General Procedure (GP) 3: Synthesis of (difluoromethyl)arenes reference compounds**

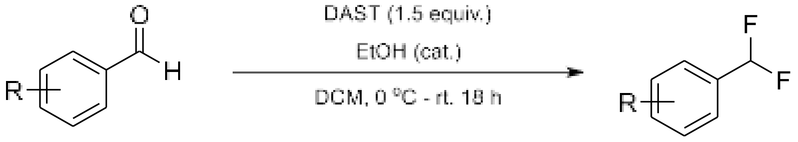


6a

A flame-dried two-necked round-bottomed flask was charged with a stir-magnetic stir bar and a starting material (if solid). To this flask was added anhydrous DCM and a drop of EtOH. The resulting mixture was cooled to 0 °C and DAST (1.5 equiv.) was added dropwise. The resulting mixture was stirred at 0 °C for a further 10 minutes before it was allowed to warm to r.t. The resulting reaction mixture was stirred until TLC showed all starting material had been consumed. The reaction mixture was then quenched with $NaHCO_3$ until gas evolution stopped and then purified by column chromatography (eluent: *n*-pentane/DCM). *Note: electron rich title compounds such as 1-(difluoromethyl)-2-methoxybenzene and 1-(difluoromethyl)-4-phenoxybenzene are unstable when neat in standard glassware. When removing solvent these compounds should be transferred into a falcon tube and stored in a freezer. In case of decomposition, the compound will turn deep purple, but can be recovered if it is passed through a plug of silica.*

**General Procedure (GP) 4: Synthesis of (difluoromethyl)arenes reference compounds**

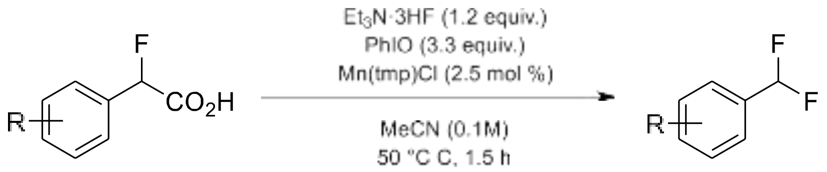


An oven-dried, 5 mL Schlenk flask equipped with a stir bar was placed under an atmosphere of $N_2$. Mn(tmp)Cl Catalyst (2.5 mol%) substrate (0.1 mmol) and DCE were then added, followed by $Et_3N$·3HF (1.2 equiv.). The reaction mixture was then heated to 50 °C. Under a stream of $N_2$, iodosylbenzene (3.3 equiv.) was added slowly to the reaction mixture in solid form over a period of 1.5 hours. After the addition of iodosylbenzene, the reaction was stirred until completion.

***Synthesis of α-aryl-α-fluoroacetic acids***

**2-([1,1'-Biphenyl]-4-yl)-2-fluoroacetic acid[2]**

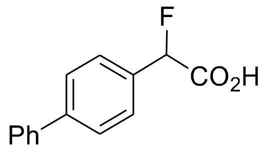


The title compound was prepared following GP1 using [1,1'-biphenyl]-4-ylboronic acid (59.4 mg, 0.3 mmol), ethyl bromofluoroacetate (67.2 µL, 0.6 mmol), $Cs_2CO_3$ (196 mg, 0.6 mmol), CuI (11.4 mg, 20 mol%) and 4,4′,4″-tri-*tert*-butyl-2,2′:6′,2″-terpyridine (24.1 mg, 20 mol%). The title compound (51.8 mg, 75% yield) was isolated as a white solid.

**Physical appearance:** White solid (yield: 75%). **$^1$H NMR** (400 MHz, DMSO-$d_6$) δ 7.74 (d, $J$ = 7.8 Hz, 2H), 7.70 – 7.66 (m, 2H), 7.56 – 7.52 (m, 2H), 7.48 (dd, $J$ = 8.4, 6.9 Hz, 2H), 7.42 – 7.37 (m, 1H), 6.04 (d, $J$ = 47.5 Hz, 1H). **$^{13}$C NMR** (101 MHz, DMSO-$d_6$) δ 170.2 (d, $J$ = 27.4 Hz), 141.7 (d, $J$ = 2.3 Hz), 140.0, 134.6 (d, $J$ = 19.6 Hz), 129.5, 128.3, 128.0 (d, $J$ = 6.0 Hz), 127.5, 127.3, 89.8 (d, $J$ = 183.2 Hz); **$^{19}$F NMR** (376 MHz, DMSO-$d_6$) δ -175.73 (d, $J$ = 47.5 Hz). The physical data were identical in all respects to those previously reported.[2]

**2-Fluoro-2-(4-phenoxyphenyl)acetic acid**

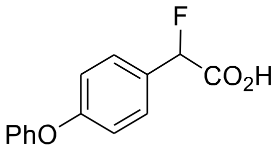


The title compound was prepared following General Procedure GP1 using (4-phenoxyphenyl)boronic acid (64.2 mg, 0.3 mmol), ethyl bromofluoroacetate (67.2 µL, 0.6 mmol), $Cs_2CO_3$ (196 mg, 0.6 mmol), CuI (11.4 mg, 20 mol%) and 4,4′,4″-tri-*tert*-butyl-2,2′:6′,2″-terpyridine (24.1 mg, 20 mol%). The title compound (51.0 mg, 69% yield) was isolated as a white solid.

**Physical appearance:** White solid (yield: 69%). **Mp:** 86 – 88 °C; **$^1$H NMR** (400 MHz, $CDCl_3$) δ: 7.45 (d, *J* = 9.4 Hz, 1H), 7.42 – 7.34 (m, 2H), 7.23 – 7.12 (m, 1H), 7.10 – 6.99 (m, 5H), 5.82 (d, *J* = 47.4 Hz, 1H); **$^{13}$C NMR** (101 MHz, $CDCl_3$) δ: 173.45 (d, *J* = 28.6 Hz), 159.25 (d, *J* = 2.4 Hz), 156.33, 130.08, 128.72 (d, *J* = 5.6 Hz), 127.74, 124.22, 119.76, 118.69, 88.59 (d, *J* = 186.4 Hz); **$^{19}$F NMR** (376 MHz, $CDCl_3$) δ: -177.21 (d, *J* = 47.7 Hz); Found, 245.06156; **IR** (film, $cm^{-1}$) υ 3038, 2917, 1768, 1589, 1243; **HRMS** (ESI) *m/z* calculated for $C_{14}H_{10}FO_3$ $[M – H]^-$ 245.06195, found, 245.06156.

**2-(3,5-Dimethylphenyl)-2-fluoroacetic acid**

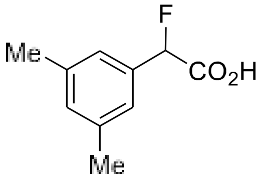


The title compound was prepared following General Procedure GP1 using (3,5-dimethylphenyl)boronic acid (45.0 mg, 0.3 mmol), ethyl bromofluoroacetate (67.2 µL, 0.6 mmol), $Cs_2CO_3$ (196 mg, 0.6 mmol), CuI (11.4 mg, 20 mol%) and 4,4′,4″-tri-*tert*-butyl-2,2′:6′,2″-terpyridine (24.1 mg, 20 mol%). The title compound (34.4 mg, 63% yield) was isolated as a white solid.

**Physical appearance:** White solid (yield: 63%). **Mp:** 68 – 70 °C; **$^1$H NMR** (400 MHz, $CDCl_3$) *δ*: 7.04 – 6.96 (m, 3H), 5.67 (d, 1H, *J* = 48.9 Hz), 2.26 (s, 6H). **$^{13}$C NMR** (101 MHz, $CDCl_3$) *δ*: 173.37 (d, *J* = 27. 8 Hz), 138.70, 133.21 (d, *J* = 20.3 Hz), 131.65 (d, *J* = 2.4 Hz), 124.50 (d, *J* = 5.9 Hz), 88.99 (d, *J* = 186.0 Hz), 21.23. **$^{19}$F NMR** (376 MHz, $CDCl_3$) *δ:* -179.17 (d, *J* = 47.4 Hz). **IR** (film, $cm^{-1}$) υ. 2919, 2166, 1725, 1066. **HRMS** (ESI) *m/z* calculated for $C_{10}H_{10}FO_2$ $[M – H]^-$ 181.06703, found, 181.06673.

**2-(2,5-Dimethylphenyl)-2-fluoroacetic acid**

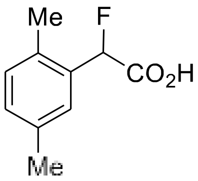


The title compound was prepared following General Procedure GP1 (3-iodophenyl)boronic acid (74.4 mg, 0.3 mmol), ethyl bromofluoroacetate (67.2 µL, 0.6 mmol), $Cs_2CO_3$ (196 mg, 0.6 mmol), CuI (11.4 mg, 20 mol%) and 4,4′,4″-tri-*tert*-butyl-2,2′:6′,2″-terpyridine (16.6 mg, 20 mol%). The title compound (42.0 mg, 52% yield) was isolated as a white solid.

**Physical appearance:** White solid (yield: 52%). **Mp**: 85 – 88 °C; **$^1$H NMR** (400 MHz, $CDCl_3$) *δ*: 7.22 (d, 1H, *J* = 10.9 Hz), 7.10 (s, 2H), 5.98 (d, 1H, *J* = 47.2 Hz), 2.38 (s, 3H), 2.31 (s, 3H); **$^{13}$C NMR** (101 MHz, $CDCl_3$) *δ:* 174.49 (d, *J* = 28.7 Hz), 136.13, 133.41 (d, *J* = 3.9 Hz), 131.68 (d, *J* = 19.1 Hz), 130.93, 130.72 (d, *J* = 2.5 Hz), 127.76 (d, *J* = 6.7 Hz), 86.64 (d, *J* = 185.6 Hz), 20.88, 18.68; **$^{19}$F NMR** (376 MHz, $CDCl_3$) *δ:* -180.02 (d, *J* = 47.2 Hz); **IR** (film, $cm^{-1}$) υ. 3024, 2929, 2845, 1708, 1243; **HRMS** (ESI) *m/z* calculated for $C_{10}H_{10}FO_2$ $[M-H]^-$ 181.06703, found, 181.06680.

**2-(4-(*tert*-Butyl)phenyl)-2-fluoroacetic acid[3]**

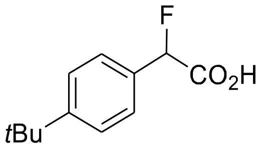


The title compound was prepared following General Procedure GP1 using (4-(tert-butyl)phenyl)boronic acid (53.4 mg, 0.3 mmol), ethyl bromofluoroacetate (67.2 µL, 0.6 mmol), $Cs_2CO_3$ (196 mg, 0.6 mmol), CuI (11.4 mg, 20 mol%) and 4,4′,4″-tri-*tert*-butyl-2,2′:6′,2″-terpyridine (24.1 mg, 20 mol%). The title compound (44.2 mg, 70% yield) was isolated as a white solid.

**Physical appearance:** White solid (yield: 70%). **$^1$H NMR** (400 MHz, $CDCl_3$**)** *δ*: 7.41 – 7.29 (m, 4H), 5.73 (d, *J* = 47.9 Hz, 1H), 1.25 (s, 9H); **$^{13}$C NMR** (101 MHz, $CDCl_3$) *δ*: 173.9 (d, *J* = 27.8 Hz), 153.3 (d, *J* = 2.0 Hz), 130.4 (d, *J* = 20.5 Hz), 126.6 (d, *J* = 5.7 Hz), 125.9, 88.7 (d, *J* = 185.4 Hz), 34.8, 31.2; **$^{19}$F NMR** (376 MHz, $CDCl_3$) δ -179.1 (d, *J* = 48.2 Hz). The physical data were identical in all respects to those previously reported.[3]

**2-Fluoro-2-(2-methoxyphenyl)acetic acid[3]**

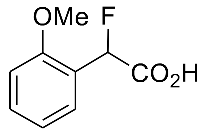


The title compound was prepared following General Procedure GP1 using (2-methoxyphenyl)boronic acid (45.6 mg, 0.3 mmol), ethyl bromofluoroacetate (67.2 µL, 0.6 mmol), $Cs_2CO_3$ (196 mg, 0.6 mmol), CuI (11.4 mg, 20 mol%) and 4,4′,4″-tri-*tert*-butyl-2,2′:6′,2″-terpyridine (16.6 mg, 20 mol%). The title compound (38.1 mg, 30% yield) was isolated as a white solid.

**Physical appearance:** White solid (yield: 30%). **$^1$H NMR** (400 MHz, $CDCl_3$) *δ*: 7.36 (t, *J* = 7.8 Hz, 1H), 7.05 (m, 1H), 7.02 (m, 1H), 6.99-6.94 (m, 1H), 5.84 (d, *J* = 46.9 Hz, 1H), 3.88 (s, 3H); **$^{13}$C NMR** (101 MHz, $CDCl_3$) *δ:* 173.66 (d, *J* = 28.8 Hz), 157.64 (d, *J* = 3.0 Hz), 132.17 (d, *J* = 2.8 Hz), 129.80 (d, *J* = 5.3 Hz), 122.62 (d, *J* = 19.2 Hz), 121.47 (d, *J* = 1.6 Hz), 111.81 (d, *J* = 1.0 Hz), 85.31 (d, *J* = 183.7 Hz), 56.27; **$^{19}$F NMR** (376 MHz, $CDCl_3$) *δ:* -178.49 (d, *J* = 46.4 Hz). The physical data were identical in all respects to those previously reported.[3]

**1-(1-Fluoro-2-hydroperoxy-2l2-ethyl)-3-methoxybenzene[2]**

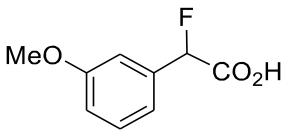


The title compound was prepared following General Procedure GP1 using (3-methoxyphenyl)boronic acid (45.6 mg, 0.3 mmol), ethyl bromofluoroacetate (67.2 µL, 0.6 mmol), $Cs_2CO_3$ (196 mg, 0.6 mmol), CuI (11.4 mg, 20 mol%) and 4,4′,4″-tri-*tert*-butyl-2,2′:6′,2″-terpyridine (24.1 mg, 20 mol%). The title compound (32.6 mg, 56% yield) was isolated as a white solid.

**Physical appearance:** White solid (yield: 56%). **$^1$H NMR** (400 MHz, $CDCl_3$**)** *δ*: 7.52 (bs, 1H), 7.30 – 7.21 (m, 1H), 7.02 – 6.91 (m, 2H), 6.88 (ddt, *J* = 8.3, 2.3, 1.0 Hz, 1H), 5.72 (d, *J* = 47.9 Hz, 1H), 3.75 (s, 3H); **$^{13}$C NMR** (100 MHz, $CDCl_3$) *δ*: 173.6 (d, *J* = 27.5 Hz), 159.9, 134.8 (d, *J* = 20.4 Hz), 130.00, 118.9 (d, *J* = 6.2 Hz) 115.7 (d, *J* = 1.4 Hz), 111.8 (d, *J* = 6.3 Hz), 88.6 (d, *J* = 186.8 Hz), 55.3; **$^{19}$F NMR** (376 MHz, $CDCl_3$) δ -181.19 (d, *J* = 48.2 Hz). The physical data were identical in all respects to those previously reported.[2]

**1-(1-Fluoro-2-hydroperoxy-2l2-ethyl)-4-(trifluoromethyl)benzene**

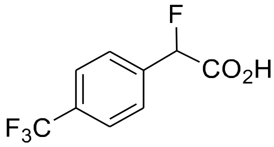


The title compound was prepared following General Procedure GP1 using (4-(trifluoromethyl)phenyl)boronic acid (57.0 mg, 0.3 mmol), ethyl bromofluoroacetate (67.2 µL, 0.6 mmol), $Cs_2CO_3$ (196 mg, 0.6 mmol), CuI (11.4 mg, 20 mol%) and 4,4′,4″-tri-*tert*-butyl-2,2′:6′,2″-terpyridine (24.1 mg, 20 mol%). The title compound (40.0 mg, 60% yield) was isolated as a yellow solid.

**Physical appearance:** Yellow solid (yield: 60%). **Mp:** 80 – 83 °C; **$^1$H NMR** (400 MHz, $CDCl_3$) δ: 7.66 – 7.51 (m, 4H), 5.83 (d, *J* = 47.1 Hz, 1H); **$^{13}$C NMR** (101 MHz, $CDCl_3$) *δ*: 173.2 (d, *J* =27.3 Hz), 133.5 – 131.1 (m), 127.7, 126.7 (d, *J* = 6.7 Hz), 125.9 (q, *J* = 3.8 Hz), 88.0 (d, *J* = 178.2 Hz); **$^{19}$F NMR** (376 MHz, $CDCl_3$) δ: -62.9 (s), -185.2 (d, *J* = 47.2 Hz); **IR** (neat, $cm^{-1}$) υ 2853, 1739, 1418, 1321, 1119, 1066. **HRMS** (ESI) *m/z* calculated for $C_9H_5F_4O_2$ $[M – H]^-$, 221.02312. found, 221.02290.

**Ethyl 2-fluoro-2-(3-formyl-4-methoxyphenyl)acetate**

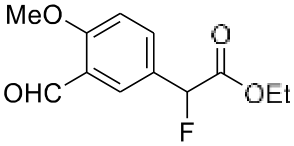


The title compound was prepared following General Procedure GP2 (3-formyl-4-methoxyphenyl)boronic acid (111.1 mg, 0.3 mmol), ethyl bromofluoroacetate (67.2 µL, 0.6 mmol), $Cs_2CO_3$ (196 mg, 0.6 mmol), CuI (11.4 mg, 20 mol%) and 4,4′,4″-tri-*tert*-butyl-2,2′:6′,2″-terpyridine (16.6 mg, 20 mol%).

**Physical appearance:** Colourless Oil. **$^1$H NMR** (400 MHz, $CDCl_3$) δ 10.45 (s, 1H), 7.91 (t, *J* = 2.0 Hz, 1H), 7.67 (ddd, *J* = 8.7, 2.5, 1.0 Hz, 1H), 7.05 (d, *J* = 8.6 Hz, 1H), 5.74 (d, *J* = 47.5 Hz, 1H), 4.33 – 4.16 (m, 2H), 3.96 (s, 3H), 1.25 (t, *J* = 7.1 Hz, 3H). **$^{13}$C NMR** (101 MHz, $CDCl_3$) δ 188.99, 168.25 (d, *J* = 27.9 Hz), 162.51 (d, *J* = 2.0 Hz), 134.05 (d, *J* = 5.4 Hz), 127.48 (d, *J* = 5.7 Hz), 126.84 (d, *J* = 21.5 Hz), 124.73, 112.29, 88.53 (d, *J* = 185.8 Hz), 61.97, 55.93, 14.04. **$^{19}$F NMR** (376 MHz, $CDCl_3$) δ -177.89 (d, *J* = 47.6 Hz). **IR** (neat, $cm^{-1}$) υ. 2921, 2159, 1978, 1758, 1644. **HRMS** (ESI) *m/z* calculated for $C_{12}H_{14}FO_4$ $[M + H]^+$, 241.0876, found, 241.0875.

**2-Fluoro-2-(3-formyl-4-methoxyphenyl)acetic acid**

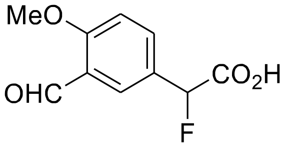

**Physical appearance:** yellow solid (yield: 53% based on two steps). **Mp**: Decomposes between 120 – 150 °C; **$^1$H NMR** (400 MHz, $CDCl_3$) δ 7.61 (s, 1H), 7.39 – 7.30 (m, 1H), 7.12 – 6.92 (m, 3H), 5.80 (d, *J* = 47.5 Hz, 1H), 3.83 (s, 3H); **$^{13}$C NMR** (101 MHz, $CDCl_3$) δ 173.58 (d, *J* = 27.9 Hz), 159.89, 134.77 (d, *J* = 20.5 Hz), 130.00, 118.91 (d, *J* = 6.3 Hz), 115.71 (d, *J* = 2.0 Hz), 111.84 (d, *J* = 6.6 Hz), 88.67 (d, *J* = 187.2 Hz), 55.37; **$^{19}$F NMR** (376 MHz, $CDCl_3$) δ -181.18 (d, *J* = 47.5 Hz); **IR** (film, $cm^{-1}$) υ. 2918, 2850, 1756, 1643, 1604, 1256, 1223; **HRMS** (ESI) *m/z* calculated for $C_{10}H_8FO_4$ $[M – H]^-$, 211.04121, Found, 211.04101.

**2-(Dibenzo[b,d]furan-4-yl)-2-fluoroacetic acid**

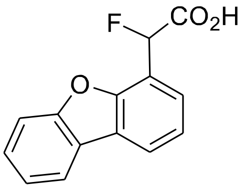

The title compound was prepared following General Procedure GP1 using dibenzo[b,d]furan-4-ylboronic acid (63.6 mg, 0.3 mmol), ethyl bromofluoroacetate (67.2 µL, 0.6 mmol), $Cs_2CO_3$ (196 mg, 0.6 mmol), CuI (11.4 mg, 20 mol%) and 4,4′,4″-tri-*tert*-butyl-2,2′:6′,2″-terpyridine (24.1 mg, 20 mol%). The title compound (38.1 mg, 52% yield) was isolated as a white solid.

**Physical appearance:** White solid (yield: 52%). **Mp**: 96 – 98 °C; **$^1$H NMR** (400 MHz, DMSO-*d*$_6$) *δ*: 13.70 (bs, 1H), 8.36 – 8.21 (m, 2H), 7.80 (d, *J* = 8.2 Hz, 1H), 7.69 – 7.43 (m, 4H), 6.51 (d, *J* = 48.2 Hz, 1H); **$^{13}$C NMR** (101 MHz, DMSO-*d*$_6$) δ: 169.6 (d, *J* = Hz), 155.9, 153.4 (d, *J* = 2.9 Hz), 128.6, 127.4 (d, *J* = 5.0 Hz Hz), 124.8, 123.9 (d, *J* = 0.9 Hz), 123.7, 123.2 (d, *J* = 2.8 Hz) 121.9, 119.6 (d, *J* = 20.1 Hz), 112.3, 85.1 (d, *J* = 179.4 Hz); **$^{19}$F NMR** (376 MHz, DMSO-*d*$_6$) δ: -176.0 (d, *J* = 48.5 Hz); **IR** (film, $cm^{-1}$) υ 3041, 1734, 1591, 1450, 743; **HRMS** (ESI) *m/z* calculated for $C_{14}H_8FO_3$ $[M – H]^-$, 243.04630. Found, 243.04589.

**Ethyl 2-fluoro-2-(3-((5-(trifluoromethyl)pyridin-2-yl)oxy)phenyl)acetate**

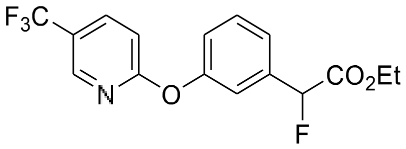

The title compound was prepared following General Procedure GP2 using (3-((5-(trifluoromethyl)pyridin-2-yl)oxy)phenyl)boronic acid (84.9 mg, 0.3 mmol), ethyl bromofluoroacetate (67.2 µL, 0.6 mmol), $Cs_2CO_3$ (196 mg, 0.6 mmol), CuI (11.4 mg, 20 mol%) and 4,4′,4″-tri-*tert*-butyl-2,2′:6′,2″-terpyridine (24.1 mg, 20 mol%).

**Physical appearance:** Yellow oil. **$^1$H NMR** (400 MHz, $CDCl_3$) δ 8.44 (dq, *J* = 2.8, 0.9 Hz, 1H), 7.96 – 7.91 (m, 1H), 7.48 (td, *J* = 7.9, 0.9 Hz, 1H), 7.40 – 7.35 (m, 1H), 7.32 – 7.29 (m, 1H), 7.21 (ddt, *J* = 8.1, 2.2, 1.0 Hz, 1H), 7.05 (dt, *J* = 8.6, 0.7 Hz, 1H), 5.81 (d, *J* = 47.6 Hz, 1H), 4.34 – 4.18 (m, 2H), 1.28 (t, *J* = 7.1 Hz, 3H); **$^{13}$C NMR** (101 MHz, $CDCl_3$) δ 168.09 (d, *J* = 27.0 Hz), 165.39 (d, *J* = 1.0 Hz), 153.39, 145.41 (q, *J* = 4.4 Hz), 136.83 (q, *J* = 3.2 Hz), 136.15 (d, *J* = 20.9 Hz), 124.97, 123.36 (d, *J* = 6.6 Hz), 122.55 (d, *J* = 1.9 Hz), 122.27, 121.86 (q, *J* = 34.3 Hz), 119.56 (d, *J* = 6.8 Hz), 111.57, 88.72 (d, *J* = 187.0 Hz), 62.03, 14.03; **$^{19}$F NMR** (376 MHz, $CDCl_3$) δ -61.71, -182.11 (d, *J* = 47.6 Hz); **IR** (film, $cm^{-1}$) υ 3005, 1751, 1280, 1126, 1064; **HRMS** (ESI) *m/z* calculated for $C_{16}H_{14}F_4NO_3$ $[M + H]^+$ 344.09043, Found, 344.09054.

**2-Fluoro-2-(3-((5-(trifluoromethyl)pyridin-2-yl)oxy)phenyl)acetic acid**

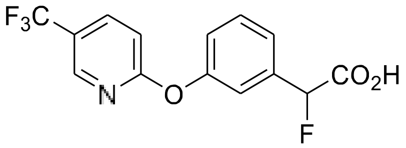

**Physical appearance:** White Solid (Yield = 70% based on two steps). **$^1$H NMR** (400 MHz, Methanol-*d*$_4$) δ 8.39 (s, 1H), 7.95 (d, *J* = 10.8 Hz, 1H), 7.67 – 7.52 (m, 4H), 6.88 (d, *J* = 8.6 Hz, 1H), 5.89 (d, *J* = 47.9 Hz, 1H), 3.95 (s, 3H). **$^{13}$C NMR** (101 MHz, $CDCl_3$) δ 170.27 (d, *J* = 27.5 Hz), 164.26, 152.25, 145.91 – 142.06 (m), 136.23 (q, *J* = 3.1 Hz), 134.61 (d, *J* = 20.8 Hz), 129.28, 123.81, 122.60 (d, *J* = 6.4 Hz), 121.77 (d, *J* = 1.7 Hz), 121.34 – 120.82 (m), 118.59 (d, *J* = 6.7 Hz), 110.83, 87.17 (d, *J* = 188.1 Hz), 28.68.**$^{19}$F NMR** (376 MHz, $CDCl_3$) δ -61.68 (s) -182.15 (d, *J* = 47.7 Hz); **IR** (film, $cm^{-1}$) υ 3735, 2361, 2341, 1748, 1329. **HRMS** (ESI) *m/z* calculated for $C_{14}H_8F_4NO_3$ $[M – H]^-$, 316.05913. Found, 316.05911.

**Ethyl 2-(4-(2H-1,2,3-triazol-2-yl)phenyl)-2-fluoroacetate**

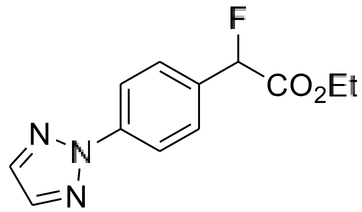

The title compound was prepared following General Procedure GP2 using (4-(2H-1,2,3-triazol-2-yl)phenyl)boronic acid (56.7 mg, 0.3 mmol), ethyl bromofluoroacetate (67.2 µL, 0.6 mmol), $Cs_2CO_3$ (196 mg, 0.6 mmol), CuI (11.4 mg, 20 mol %) and 4,4′,4″-tri-*tert*-butyl-2,2′:6′,2″-terpyridine (16.6 mg, 20 mol %).

**Physical appearance:** Colourless oil. **$^1$H NMR** (400 MHz, Methanol-*d*$_4$) δ 8.17 – 8.10 (m, 2H), 7.93 (s, 2H), 7.65 – 7.59 (m, 2H), 5.98 (d, *J* = 47.2 Hz, 1H), 4.40 – 4.10 (m, 2H), 1.23 (t, *J* = 7.1 Hz, 3H). **$^{13}$C NMR** (101 MHz, Methanol-*d*$_4$) δ 170.00 (d, *J* = 28.0 Hz), 137.32, 135.39 (d, *J* = 20.6 Hz), 129.14 (d, *J* = 6.2 Hz), 120.04, 89.90 (d, *J* = 183.6 Hz), 62.99, 14.31. **$^{19}$F NMR** (376 MHz, Methanol-*d*$_4$) δ -180.95 (d, *J* = 47.2 Hz). **IR** (neat, cm$^{-1}$) υ 1935, 1556, 1138, 1096; **HRMS** (ESI) *m/z* calculated for $C_{12}H_{13}FN_3O_2$ $[M + H]^+$ for 250.09863, Found, 250.09874.

**2-(4-(2H-1,2,3-Triazol-2-yl)phenyl)-2-fluoroacetic acid**

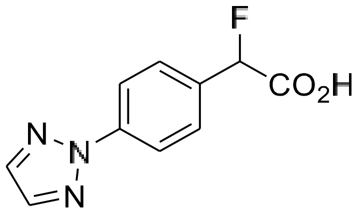


**Physical appearance:** White solid (yield: 40%, based on two steps). **Mp:** 140 – 142 °C; **$^1$H NMR** (500 MHz, Acetonitrile-*d*$_3$) δ 8.30 (d, *J* = 8.0 Hz, 2H), 8.09 (s, 2H), 7.82 – 7.75 (m, 2H), 6.12 (d, *J* = 47.2 Hz, 1H); **$^{13}$C NMR** (101 MHz, Methanol-*d*$_4$) δ 170.41 (d, *J* = 27.4 Hz), 140.35, 135.86, 134.42 (d, *J* = 20.7 Hz), 127.67 (d, *J* = 5.8 Hz), 118.57, 88.45 (d, *J* = 182.8 Hz); **$^{19}$F NMR** (470 MHz, Acetonitrile-*d*$_3$) δ -178.45 (d, *J* = 47.2 Hz); **IR** (neat, cm$^{-1}$) υ 2849, 1736, 1118, 1066; **HRMS** (ESI) *m/z* calculated for $C_{10}H_7FN_3O_2$ $[M – H]^-$, 220.05278, Found, 220.05232.

**Ethyl 2-(3-(3,5-dimethyl-1H-pyrazol-1-yl)phenyl)-2-fluoroacetate**

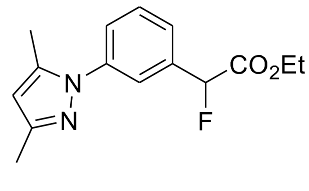


The title compound was prepared following General Procedure GP2 using (3-(3,5-dimethyl-1H-pyrazol-1-yl)phenyl)boronic acid (64.8 mg, 0.3 mmol), ethyl bromofluoroacetate (67.2 µL,

0.6 mmol), $Cs_2CO_3$ (196 mg, 0.6 mmol), CuI (11.4 mg, 20 mol %) and 4,4′,4″-tri-*tert*-butyl-2,2′:6′,2″-terpyridine (16mg, 20 mol %).

**Physical appearance:** Colourless oil. **$^1$H NMR** (500 MHz, $CDCl_3$) δ 7.56 (dt, *J* = 2.5, 1.2 Hz, 1H), 7.53 – 7.47 (m, 2H), 7.48 – 7.43 (m, 1H), 6.02 (s, 1H), 5.83 (d, *J* = 47.5 Hz, 1H), 4.33 – 4.17 (m, 2H), 2.34 – 2.28 (m, 6H), 1.27 (t, *J* = 7.1 Hz, 3H). **$^{13}$C NMR** (126 MHz, $CDCl_3$) δ 168.53 (d, *J* = 27.2 Hz), 149.78, 140.71, 139.86, 135.71 (d, *J* = 20.7 Hz), 129.92, 125.97 (d, *J* = 1.9 Hz), 125.38 (d, *J* = 6.4 Hz), 123.06 (d, *J* = 6.7 Hz), 107.80, 89.21 (d, *J* = 186.9 Hz), 62.45, 14.45, 13.90, 12.86. **$^{19}$F NMR** (470 MHz, $CDCl_3$) δ -181.63 (d, *J* = 47.6 Hz). **IR** (film, $cm^{-1}$) υ 1935, 1710, 1201; **HRMS** (ESI) *m/z* calculated for $C_{15}H_{17}FN_2O_2$ $[M + H]^+$ for 277.13468, Found, 277.13473.

**2-(3-(3,5-Dimethyl-1H-pyrazol-1-yl)phenyl)-2-fluoroacetic acid**

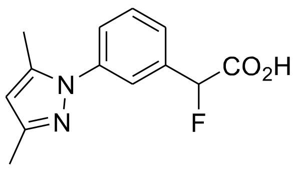


**Physical appearance:** White solid (yield: 60%, based on two steps). **Mp**: 122 – 125 °C; **$^1$H NMR** (400 MHz, Methanol-*d*$_4$) δ 7.61 – 7.53 (m, 3H), 7.51 (d, *J* = 2.6 Hz, 1H), 6.09 (1, 1H), 5.97 (d, *J* = 47.6 Hz, 1H), 2.28 (s, 3H), 2.25 (s, 3H); **$^{13}$C NMR** (101 MHz, Methanol-*d*$_4$) δ 170.18 (d, *J* = 26.8 Hz), 149.30, 140.51, 139.63, 136.58 (d, *J* = 20.7 Hz), 129.38, 126.70 – 125.12 (m), 122.97, 106.80, 88.39 (d, *J* = 183.4 Hz), 11.76, 10.76; **$^{19}$F NMR** (376 MHz, Methanol-*d*$_4$) δ -181.91 (d, *J* = 47.7 Hz); **IR** (film, $cm^{-1}$) υ 2917, 2849, 1745, 1148; **HRMS** (ESI) *m/z* calculated for $C_{13}H_{14}FN_2O_2$ $[M + H]^+$, 249.10338, Found, 249.10327.

**Ethyl 2-fluoro-2-(4-(5-(p-tolyl)-3-(trifluoromethyl)-1H-pyrazol-1-yl)phenyl)acetate**

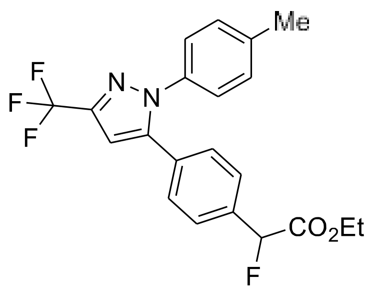

The title compound was prepared following General Procedure GP2 (4-(1-(p-tolyl)-3-(trifluoromethyl)-1H-pyrazol-5-yl)phenyl)boronic acid (104 mg, 0.3 mmol), ethyl bromofluoroacetate (67.2 µL, 0.6 mmol), $Cs_2CO_3$ (196 mg, 0.6 mmol), CuI (11.4 mg, 20 mol%) and 4,4′,4″-tri-*tert*-butyl-2,2′:6′,2″-terpyridine (16.6 mg, 20 mol%).

**Physical appearance:** Colourless Oil. **$^1$H NMR** (500 MHz, $CDCl_3$) δ 7.52 – 7.47 (m, 2H), 7.42 – 7.37 (m, 2H), 7.17 (d, *J* = 8.1 Hz, 2H), 7.14 – 7.12 (m, 2H), 6.75 (s, 1H), 5.82 (d, *J* = 47.6 Hz, 1H), 4.27 (qq, *J* = 10.8, 7.1 Hz, 2H), 2.39 (s, 3H), 1.28 (t, *J* = 7.1 Hz, 3H); **$^{13}$C NMR** (126 MHz, $CDCl_3$) δ 167.99 (d, *J* = 27.2 Hz), 144.92, 144.26 – 142.70 (m), 140.22 (d, *J* = 2.4 Hz), 139.37, 134.30 (d, *J* = 20.9 Hz), 129.53, 128.68, 127.28 (d, *J* = 6.4 Hz), 125.60, 124.69 – 117.40 (m), 105.69 (d, *J* = 2.4 Hz), 88.64 (d, *J* = 186.8 Hz), 62.09, 29.72, 21.32, 14.03; **$^{19}$F NMR** (470 MHz, $CDCl_3$) δ -62.33, -181.94 (d, *J* = 48.7 Hz); **IR** (film, $cm^{-1}$) υ 2985, 1759, 1235, 1131; **HRMS** (ESI) *m/z* calculated for $C_{21}H_{19}F_4N_2O_2$ $[M + H]^+$, 407.13772, Found, 407.13719

**2-Fluoro-2-(4-(5-(p-tolyl)-3-(trifluoromethyl)-1H-pyrazol-1-yl)phenyl)acetic acid**

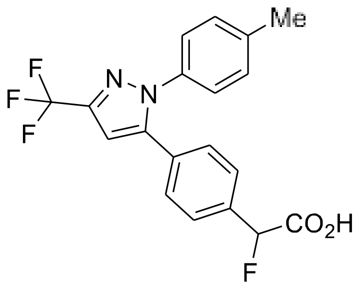

**Physical appearance:** White solid (yield: 41%, based on two steps). **Mp**: 148 – 152 °C. **$^1$H NMR** (400 MHz, Methanol-*d*4) δ 7.46 – 7.40 (m, 2H), 7.31 – 7.24 (m, 2H), 7.09 – 7.02 (m, 4H), 6.77 (s, 1H), 5.82 (d, *J* = 47.6 Hz, 1H), 2.23 (s, 3H); **$^{19}$F NMR** (376 MHz, Methanol-*d*4) δ -63.77, -181.72 (d, *J* = 47.9 Hz); **$^{13}$C NMR** (101 MHz, Methanol-*d*4) δ 170.12 (d, *J* = 27.1 Hz), 145.49, -142.98 (q, *J* = 38.5 Hz), 139.96 (d, *J* = 2.3 Hz), 139.38, 135.72 (d, *J* = 20.6 Hz), 129.09, 128.59, 127.23 (d, *J* = 6.2 Hz), 125.92, 125.58, 121.31 (q, *J* = 290.3 Hz), 105.0, 88.30 (d, *J* = 183.4 Hz), 19.86; **IR** (film, $cm^{-1}$) υ 2918, 2360, 1748, 1510, 1475, 1456; **HRMS** (ESI) *m/z* calculated for $C_{19}H_{14}F_4N_2O_2$ $[M + H]^+$, 379.10642, Found, 379.10639.

**2-Fluoro-2-(4-(4-((1-isopropoxy-2-methyl-1-oxopropan-2-yl)oxy)benzoyl)phenyl)acetic acid**

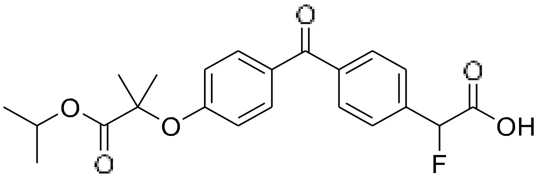

The title compound was prepared following General Procedure GP2 using (4-(4-((1-isopropoxy-2-methyl-1-oxopropan-2-yl)oxy)benzoyl)phenyl)boronic acid (111.1 mg, 0.3 mmol), ethyl bromofluoroacetate (67.2 µL, 0.6 mmol), $Cs_2CO_3$ (196 mg, 0.6 mmol), CuI (11.4 mg, 20 mol%) and 4,4′,4″-tri-*tert*-butyl-2,2′:6′,2″-terpyridine (16.6 mg, 20 mol%). The title compound (60.4 mg, 72% yield) was isolated as a brown solid.

**Physical appearance:** brown solid (yield: 72%). **Mp**: 140 – 142 °C; **$^1$H NMR** (400 MHz, $CDCl_3$) $\delta$: 7.69 (dd, *J* = 8.4, 4.2 Hz, 4H), 7.52 (d, *J* = 7.9 Hz, 2H), 6.78 (d, *J* = 8.4 Hz, 2H), 5.82 (d, *J* = 47.5 Hz, 1H), 5.00 (m, 1H), 1.59 (s, 6H), 1.13 (d, *J* = 6.2 Hz, 6H); **$^{13}$C NMR** (101 MHz, $CDCl_3$) $\delta$: 195.41, 173.17, 171.80 (d, *J* = 28.9 Hz), 160.05, 139.18, 137.31 (d, *J* = 20.4 Hz), 132.32, 130.09, 129.87, 126.27 (d, *J* = 6.6 Hz), 117.24, 88.35 (d, *J* = 188.8 Hz), 79.48, 69.49, 25.36, 21.51; **$^{19}$F NMR** (376 MHz, $CDCl_3$) $\delta$: -183.28 (d, *J* = 47.8 Hz); **IR** (film, $cm^{-1}$) υ. 2985, 2361, 1718, 1558, 1503, 1252, 1146, 680; **HRMS** (ESI) *m/z* calculated for $[M + H]^+$, 403.15514, Found, 403.15535.

**2-(3-Bromophenyl)-2-fluoroacetic acid**

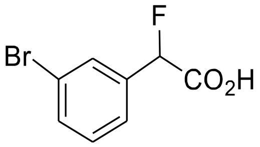

The title compound was prepared following General Procedure GP1 using (3-bromophenyl)boronic acid (60.2 mg, 0.3 mmol), ethyl bromofluoroacetate (67.2 µL, 0.6 mmol), $Cs_2CO_3$ (196 mg, 0.6 mmol), CuI (11.4 mg, 20 mol%) and 4,4′,4″-tri-*tert*-butyl-2,2′:6′,2″-terpyridine (16.6 mg, 20 mol%). The title compound (35.0 mg, 58% yield) was isolated as a brown solid.

**Physical appearance:** Brown Solid (yield: 58%). **Mp**: 81 – 83 °C; **$^1$H NMR** (400 MHz, $CDCl_3$) $\delta$: 10.10 (bs, 1H), 7.56 (d, *J* = 2.0 Hz, 1H), 7.47 (dd, *J* = 8.0, 2.0 Hz, 1H), 7.34 (dd, *J* = 7.8, 1.5 Hz, 1H), 7.21 (t, *J* = 7.9 Hz, 1H), 5.71 (d, *J* = Hz, H); **$^{13}$C NMR** (100 MHz, $CDCl_3$) $\delta$: 173.7 (d, *J* =27.3 Hz), 135.4 (d, *J* = 21.2 Hz), 133.0 (d, *J* = 2.1 Hz), 130.5, 129.5 (d, *J* =7.1 Hz), 125.1 (d, *J* = 6.0 Hz), 122.9, 87.3 (d, *J* = 178.3 Hz); **$^{19}$F NMR** (376 MHz, $CDCl_3$) δ -

183.16 (d, *J* = 47.1 Hz); **IR** (film, $cm^{-1}$) υ 3035, 1759, 1689, 156, 818; **HRMS** (ESI) *m/z* calculated for $C_8H_5BrFO_2$ $[M – H]^-$, 230.94624. Found, 230.94586.

**2-(4-Bromophenyl)-2-fluoroacetic acid**[3]

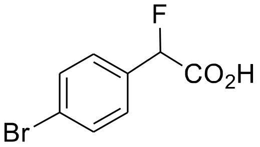


The title compound was prepared following General Procedure GP1 using (4-bromophenyl)boronic acid (60.3 mg, 0.3 mmol), ethyl bromofluoroacetate (67.2 µL, 0.6 mmol), $Cs_2CO_3$ (196 mg, 0.6 mmol), CuI (11.4 mg, 20 mol%) and 4,4′,4″-tri-*tert*-butyl-2,2′:6′,2″-terpyridine (24.1 mg, 20 mol%). The title compound (36.3 mg, 52% yield) was isolated as a brown solid.

**Physical appearance:** Brown solid (yield: 52%). **$^1$H NMR** (400 MHz, $CDCl_3$**)** *δ*: 10.44 (bs, 1H), 7.58 (d, *J* = 8.2 Hz, 2H), 7.38 (d, *J* = 8.2 Hz, 2H), 5.80 (d, *J* = 47.2 Hz, 1H); **$^{13}$C NMR** (100 MHz, $CDCl_3$) *δ*: 173.8 (d, *J* = 27.4 Hz), 132.4 (d, *J* = 21.1 Hz), 132.1, 128.2 (d, *J* = 6.1 Hz), 124.3 (d, *J* = 2.9 Hz), 88.2 (d, *J* = 175.4 Hz); **$^{19}$F NMR** (376 MHz, $CDCl_3$) δ -182.3 (d, *J* = 47.2 Hz). The physical data were identical in all respects to those previously reported.[3]

**2-Fluoro-2-(3-iodophenyl)acetic acid**

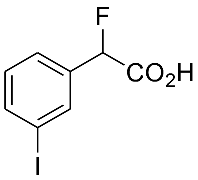


The title compound was prepared following General Procedure GP1 (3-iodophenyl)boronic acid (74.4 mg, 0.3 mmol), ethyl bromofluoroacetate (67.2 µL, 0.6 mmol), $Cs_2CO_3$ (196 mg, 0.6 mmol), CuI (11.4 mg, 20 mol%) and 4,4′,4″-tri-*tert*-butyl-2,2′:6′,2″-terpyridine (16.6 mg, 20 mol%). The title compound (42.0 mg, 50% yield) was isolated as a white solid.

**Physical appearance:** White Solid (yield: 50%). **Mp**: 102 – 104 °C; **$^1$H NMR** (400 MHz, Methanol-$d_4$) δ 7.83 (s, 1H), 7.77 (d, *J* = 8.7 Hz, 1H), 7.48 (d, *J* = 6.8 Hz, 1H), 7.19 (t, *J* = 7.8 Hz, 1H), 5.83 (d, *J* = 47.6 Hz, 1H). **$^{13}$C NMR** (101 MHz, Methanol-$d_4$) δ 171.6 (d, *J* = 27.4 Hz), 139.7, 138.8 (d, *J* = 20.6 Hz), 136.7 (d, *J* = 6.4 Hz), 127.1 (d, *J* = 6.1 Hz), 89.5 (d, *J* = 183.2 Hz); **$^{19}$F NMR** (376 MHz, Methanol-$d_4$) δ -181.77 (d, *J* = 47.8 Hz); **IR** (film, $cm^{-1}$) υ

3021, 2613, 1689, 1766, 1703; **HRMS** (ESI) *m/z* calculated for $C_8H_5FIO_2$ [M – H]$^-$ for 278.93237, Found, 278.93228.

**2-Fluoro-2-(m-tolyl)acetic acid**[4]

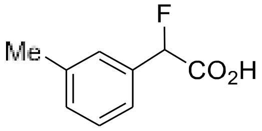


The title compound was prepared following General Procedure GP1 using m-tolylboronic acid (40.8 mg, 0.3 mmol), ethyl bromofluoroacetate (67.2 µL, 0.6 mmol), $Cs_2CO_3$ (196 mg, 0.6 mmol), CuI (11.4 mg, 20 mol%) and 4,4′,4″-tri-*tert*-butyl-2,2′:6′,2″-terpyridine (24.1 mg, 20 mol%). The title compound (36.3 mg, 74% yield) was isolated as a white solid.

**Physical appearance:** White solid (yield: 74%). **$^1$H NMR** (400 MHz, $CDCl_3$**)** *δ*: 10.16 (bs, 1H), 7.26 – 7.12 (m, 4H), 5.70 (d, *J* = 48.8 Hz, 1H), 2.30 (s, 3H); **$^{13}$C NMR** (100 MHz, $CDCl_3$) *δ*: 174.4 (d, *J* = 28.1 Hz), 138.8, 133.3 (d, *J* = 20.1 Hz), 130.8 (d, *J* = 2.4 Hz), 128.8, 127.3 (d, *J* = 6.1 Hz), 123.9 (d, *J* = 6.3 Hz), 88.9 (d, *J* = 186.2 Hz), 21.4; **$^{19}$F NMR** (376 MHz, $CDCl_3$) δ -180.3 (d, *J* = 48.9 Hz). The physical data were identical in all respects to those previously reported.[4]

**2-Fluoro-4-(p-tolyl)acetic acid**[4]

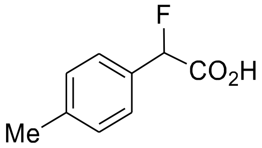


The title compound was prepared following General Procedure GP1 using p-tolylboronic acid (40.8 mg, 0.3 mmol), ethyl bromofluoroacetate (67.2 µL, 0.6 mmol), $Cs_2CO_3$ (196 mg, 0.6 mmol), CuI (11.4 mg, 20 mol%) and 4,4′,4″-tri-*tert*-butyl-2,2′:6′,2″-terpyridine (24.1 mg, 20 mol%). The title compound (36.3 mg, 72% yield) was isolated as a white solid.[3]

**Physical appearance:** White solid (yield: 72%). **$^1$H NMR** (400 MHz, $CDCl_3$**)** *δ*: 7.11 (d, 2H), 7.30 (d, 2H), 5.45 (d, *J* = 47.6 Hz, 1H), 2.15 (s, 3H); **$^{19}$F NMR** (376 MHz, $CDCl_3$) δ: 178.4 (d, *J* = 47.4 Hz); The physical data were identical in all respects to those previously[4]

**2-Fluoro-2-(o-tolyl)acetic acid**[5]

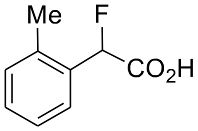

The title compound was prepared following General Procedure GP1 using o-tolylboronic acid (40.8 mg, 0.3 mmol), ethyl bromofluoroacetate (67.2 µL, 0.6 mmol), $Cs_2CO_3$ (196 mg, 0.6 mmol), CuI (11.4 mg, 20 mol%) and 4,4′,4″-tri-*tert*-butyl-2,2′:6′,2″-terpyridine (24.1 mg, 20 mol%). The title compound (15.1 mg, 30% yield) was isolated as a white solid.

**Physical appearance:** White solid (yield: 30%). **$^1$H NMR** (400 MHz, $CDCl_3$**)** *δ*: 7.18-7.28 (m, 4H), 5.48 (d, *J* = 48.2 Hz, 1H,), 2.28 (t, 3H); **$^{19}$F NMR** (376 MHz, $CDCl_3$) δ: 178.8 (d, *J* = 48.4 Hz). The physical data were identical in all respects to those previously reported.[5]

**2-Fluoro-2-phenylacetic acid[2]**

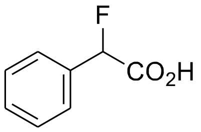


The title compound was prepared following General Procedure GP1 using phenylboronic acid (36.6 mg, 0.3 mmol), ethyl bromofluoroacetate (67.2 µL, 0.6 mmol), $Cs_2CO_3$ (196 mg, 0.6 mmol), CuI (11.4 mg, 20 mol%) and 4,4′,4″-tri-*tert*-butyl-2,2′:6′,2″-terpyridine (24.1 mg, 20 mol%). The title compound (27.3 mg, 59% yield) was isolated as a white solid.

**Physical appearance:** White solid (yield: 59%). **$^1$H NMR** (400 MHz, $CDCl_3$**)** *δ*: 8.67 (bs, 1H), 7.46 – 7.31 (m, 5H), 5.75 (d, *J* = Hz, 1H); **$^{13}$C NMR** (100 MHz, $CDCl_3$) *δ*: 173.8 (d, *J* = 27.8 Hz), 133.4 (d, *J* = 20.6 Hz), 130.0 (d *J* = 2.2 Hz), 128.9, 126.7 (d, *J* = 5.9 Hz), 88.8 (d, *J* = 186.8 Hz); **$^{19}$F NMR** (376 MHz, $CDCl_3$) δ -180.8 (d, *J* = 47.5 Hz). The physical data were identical in all respects to those previously reported.[2]

**Ethyl 2-diazo-2-(4-(6-methoxypyridin-3-yl)phenyl)acetate**

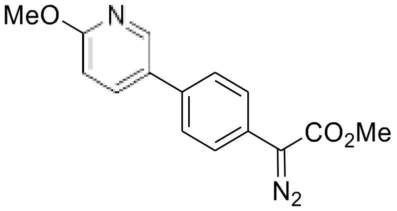

To a mixture of methyl 2-(4-(6-methoxypyridin-3-yl)phenyl)acetate (10 mmol, 1 equiv.) and *p*-ABSA (12 mmol, 1.2 equiv.) in anhydrous MeCN (30 mL), DBU (14 mmol, 1.4 equiv.) was added at 0°C. Th reaction mixture was stirred at r.t overnight. Upon the complete consumption of the starting materials, the reaction mixture was diluted with distilled water (20 mL), followed by extraction with $Et_2O$ (3 × 10 mL). After washing with 10% $NH_4Cl$ solution (3×10 mL) and brine (3×10 mL), the combined organic extracts were dried over $MgSO_4$, concentrated and chromatographed EtOAc:Hexane (10:90) to yield the diazoester.

**Physical appearance:** Orange solid. **Mp:** 135 – 137 °C; **$^1$H NMR** (400 MHz, $CDCl_3$**)** $\delta$: 8.39 (dd, $J$ = 2.7, 0.8 Hz, 1H), 7.79 (dd, $J$ = 8.6, 2.6 Hz, 1H), 7.56 (bs, 4H), 6.82 (dd, $J$ = 8.6, 0.7 Hz, 1H), 3.98 (s, 3H), 3.89 (s, 3H). **$^{13}$C NMR** (100 MHz, $CDCl_3$) $\delta$: 165.5, 163.7, 144.8, 137.2, 135.4, 129.3, 127.1, 124.5, 124.5, 110.9, 53.6, 52.1. **IR** (film, $cm^{-1}$) υ 3012, 2951, 2094, 1686, 1160, 815. **HRMS** (ESI) *m/z* calculated for $C_{15}H_{14}N_3O_3$ [M + H]$^+$, 284.10297 Found, 284.10294.

**Methyl 2-fluoro-2-(4-(6-methoxypyridin-3-yl)phenyl)acetate**

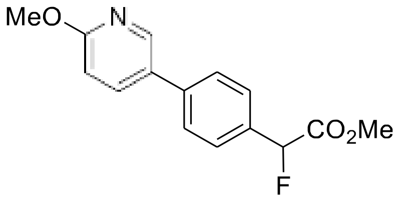


In a 20 mL falcon tube was charged with methyl 2-diazo-2-(4-(6-methoxypyridin-3-yl)phenyl)acetate (0.3 mmol) in 2mL of DCM and cooled to 0°C. HF pyridine (70 %) (0.36 mmol) was then added slowly. The reaction was temperature was raised to r.t after gas evolution. The reaction was monitored by TLC until completion. The reaction was then quenched with $NaHCO_3$ and the organic layer was extracted with DCM (3 x 5 mL). The organic layer was dried over $MgSO_4$, concentrated and chromatographed EtOAc:Hexane (10:90).

**Physical appearance:** White solid. **Mp:** 64 – 65 °C; **$^1$H NMR** (400 MHz, $CDCl_3$**)** $\delta$: 8.32 (dd, $J$ = 2.6, 0.8 Hz, 1H), 7.71 (dd, $J$ = 8.6, 2.6 Hz, 1H), 7.54 – 7.43 (m, 4H), 6.76 (dd, $J$ = 8.6, 0.7 Hz, 2H), 5.76 (d, $J$ = 48.2 Hz, 1H), 3.92 (s, 3H), 3.74 (s, 3H); **$^{13}$C NMR** (100 MHz, $CDCl_3$) $\delta$: 168.9 (d, $J$ = 27.2 Hz), 163.9, 145.1, 139.4 (d, $J$ = 3.2 Hz), 137.4, 133.1 (d, $J$ =20.0 Hz), 129.2, 127.3 (d, $J$ = 6.2 Hz), 127.1, 111.0, 89.1 (d, $J$ = 184.3 Hz), 53.6, 52.8; **$^{19}$F NMR** (376 MHz, $CDCl_3$) δ -179.9 (d, $J$ = 48.2 Hz); **IR** (film, $cm^{-1}$) υ 3014, 2949, 1498, 1195, 822; **HRMS** (ESI) *m/z* calculated for $C_{15}H_{15}FNO_3$ [M + H]$^+$, 276.10305. Found, 276.10293.

**2-Fluoro-2-(4-(6-methoxypyridin-3-yl)phenyl)acetic acid**

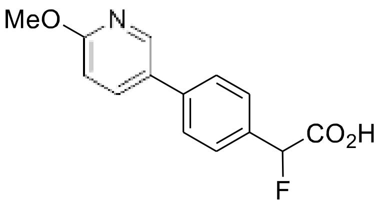


In a 50 mL round bottom flask, methyl 2-fluoro-2-(4-(6-methoxypyridin-3-yl)phenyl)acetate (5 mmol, 1.0 equiv.) was added to a mixture of MeOH (15 mL, 0.3 M) and 1 M $K_2CO_3$ aq. (15 mL) and stirred at r.t until TLC showed consumption of starting material. The reaction was then poured into 1 M HCl aq. to acidify to pH 5, and the aqueous phase was extracted with $Et_2O$ (3 × 10 mL), washed with brine (10 mL), dried over $MgSO_4$. The resulting gum was then washed with pentane, until a white free-flowing white solid formed.

**Physical appearance:** White solid (yield: 45% (over 3 steps)). **Mp:** 150 – 153 °C; **$^1$H NMR** (400 MHz, Methanol-*d*$_4$) *δ*: 8.44 – 8.38 (m, 1H), 7.97 (dd, *J* = 8.7, 2.5 Hz, 1H), 7.66 (d, *J* = 8.0 Hz, 2H), 7.58 (d, *J* = 8.0 Hz, 2H), 6.90 (d, *J* = 8.6 Hz, 1H), 5.91 (d, *J* = 48.7 Hz, 1H), 3.97 (s, 3H); **$^{13}$C NMR** (100 MHz, Methanol-*d*$_4$) *δ*: 170.8 (d, *J* = 27.9 Hz ), 163.9, 144.5, 138.7 (d, *J* = 2.1 Hz), 134.3 (d, *J* = 20.1 Hz), 127.2 (d, *J* = 6.1 Hz), 126.5, 110.4, 88.8 (d, *J* = 180.2 Hz), 52.8; **$^{19}$F NMR** (376 MHz, Methanol-*d*$_4$) -179.6 (d, *J* = 47.9 Hz); **IR** (film, cm$^{-1}$) υ 2360, 1736, 1607, 1041, 825; **HRMS** (ESI) *m/z* calculated for $C_{14}H_{13}FNO_3$ [M + H]$^+$ 262.08740 Found, 262.08731.

**2-Fluoro-2-(6-methoxynaphthalen-2-yl)acetic acid**

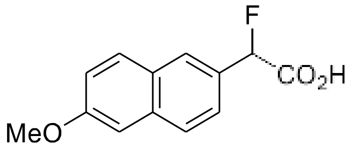


An oven-dried Schlenk tube containing a magnetic stir bar was charged with $Pd(OAc)_2$ (5.6 mg, 0.025 mmol, 5 mol%), $PPh_3$ (26.2 mg, 0.10 mmol, 20 mol%), boronic acid (1.0 mmol, 2.0 equiv.), and $K_3PO_4 \cdot 3\ H_2O$ (400 mg, 1.5 mmol, 3.0 equiv.). The Schlenk tube was sealed with a rubber septum and then evacuated and backfilled with $N_2$ three times. Toluene (5.0 mL) was added through the septum via syringe and the resulting mixture was stirred at r.t for 5 min. Ethyl *a*-bromo-*a*-fluoroacetate (92.5 mg, 0.50 mmol, 1.0 equiv.) was then added dropwise via syringe. The Schlenk tube was sealed and the mixture was heated at 100 °C with vigorous stirring for 3 h. The mixture was then allowed to cool to r.t and quenched with $H_2O$ (10 mL). The layers were separated and the aqueous phase was extracted with EtOAc (3 × 5 mL). The combined organic phases were washed with brine (20 mL), dried with $MgSO_4$, filtered, and concentrated. The crude product was purified by flash chromatography (silica gel (Pentane–EtOAc, 50:1) to give the title the ester product. The ester was then dissolved in a 2:1 MeOH and aqueous $K_2CO_3$ (5-10 equiv.) mixture before it was left to stir at r.t until all the ester had been consumed (determined by TLC). The resulting mixture was then acidified using HCl (5 M) to pH = 2. The aqueous layer was then extracted with $Et_2O$ (3 x 20 mL) and washed with Brine (2 x 20 mL). The organic extracts were dried using $MgSO_4$ and were then concentrated under vacuum and washed with pentane, resulting in the title compound as a white solid.

**Physical appearance:** White solid (yield: 50% over two steps). **Mp**: 150 – 152 °C; **$^1$H NMR** (400 MHz, Methanol-$d_4$**)** $\delta$: 7.78 (t, $J$ = 2.0 Hz, 1H), 7.70 (t, $J$ = 9.4 Hz, 2H), 7.40 (dd, $J$ = 8.5, 1.8 Hz, 1H), 7.16 (d, $J$ = 2.5 Hz, 1H), 7.07 (dd, $J$ = 8.9, 2.5 Hz, 1H), 5.86 (d, 1H, $J$ = 47.8 Hz), 3.82 (s, 3H); **$^{13}$C NMR** (100 MHz, Methanol-$d_4$) $\delta$: 158.58, 135.19 (d, $J$ = 1.8 Hz) 130.03 (d, $J$ = 20.4 Hz), 129.29, 128.44, 127.12, 126.40 (d, $J$ = 6.8 Hz) 123.95 (d, $J$ = 5.1 Hz), 119.09, 105.32, 89.35 (d, $J$ = 181.3 Hz) 54.39. **$^{19}$F NMR** (376 MHz, Methanol-$d_4$) $\delta$: -177.22 (d, $J$ = 48.0 Hz); **IR** (film, $cm^{-1}$) $\upsilon$.3157, 1764, 1703, 1606. 1267, 1234, 1051, 1029, 815; **HRMS** (ESI) *m/z* calculated for $C_{13}H_{10}FO_3$ $[M – H]^-$ for 233.06195, Found, 233.06185.

**2-Fluoro-2-(naphthalen-1-yl)acetic acid[3]**

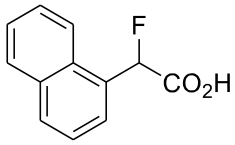


***Step 1:*** A flame dried flask containing a solution of arylacetic acid (559 mg, 3.0 mmol, 1.0 equiv.) and TBSCl (1.04 g, 6.90 mmol, 2.3 equiv.) in THF (0.50 M) was cooled to 0 °C. LiHMDS (1.0 M in THF; 2.2 equiv.) was added slowly and the resulting mixture was stirred at 0 °C for 15 min and then warmed to r.t. It was then stirred at r.t over night before volatiles were removed in vacuo. The crude was taken up in hexane and solid LiCl was filtered off. The filtrate was washed with hexane and the combined organic fractions were concentrated to dryness in vacuo.

***Step 2:*** The residue was dissolved in MeCN (0.50 M) and slowly added to a solution of Selectfluor (2.30 g, 6.50 mmol, 1.3 equiv.) in MeCN (30 mL) at r.t. After stirring for 15 min at r.t the reaction mixture was poured into aq. HCl (1.0 M, 10 mL/mmol acid). This solution was extracted with $Et_2O$ (2x) and the combined $Et_2O$ layers were then extracted with NaOH (1.0 M; 2x). The combined aqueous layers were washed with $Et_2O$ (2x), acidified with HCl (6.0 M) until pH = 1 and extracted with $Et_2O$ (3x). The combined organic fractions were dried over $MgSO_4$ and concentrated to dryness in vacuo to give the desired aryl(fluoro)acetic acid. The title compound (817 mg, 80%) as a white solid.

**Physical appearance:** White solid (yield: 80%). **$^1$H NMR** (400 MHz, $CDCl_3$): δ = 9.55 (br s, 1H), 8.17 (d, *J* = 8.2 Hz, 1H), 7.96 -7.89 (m, 2H), 7.64 (d, *J* = 7.2 Hz, 1H), 7.61 -7.52 (m, 2H), 7.52 -7.47 (m, 1H), 6.42 (d, *J* = 46.8 Hz, 1H); **$^{13}$C NMR** (101 MHz, $CDCl_3$): δ = 174.3 (d, *J* = 28.6 Hz), 133.9, 130.8 (d, *J* = 2.2 Hz), 130.5 (d, *J* = 1.5 Hz), 129.3 (d, *J* = 18.3 Hz), 128.9, 127.2, 126.9 (d, *J* = 8.1 Hz), 126.3, 125.0, 123.5 (d, *J* = 1.5 Hz), 88.0 (d, *J* = 186.3 Hz); **$^{19}$F NMR** (376 MHz, $CDCl_3$): δ = -178.7 (d, *J* = 46.3 Hz); The physical data were identical in all respects to those previously reported.[3]

**Ethyl 2-fluoro-2-(4-(1-(4-(methylsulfonyl)phenyl)-3-(trifluoromethyl)-1H-pyrazol-5-yl)phenyl)acetate**

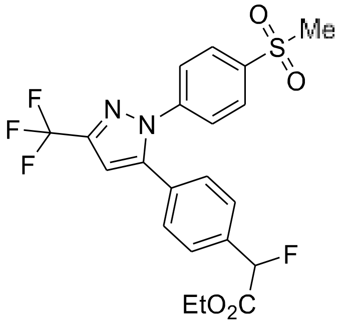


To a dry screw-cap tube equipped with a magnetic stirrer, 30 mg of 1-(4-(methylsulfonyl)phenyl)-1H-pyrazole (0.13 mmol, 1.1 equiv.), 1.5 mg of $Pd(OAc)_2$ (0.006 mmol, 5 mol%), 5 mg of L-proline (0.012 mmol, 10 mol%), 18 mg of $K_2CO_3$ (0.18 mmol, 1.5 equiv.), 17 mg of $Ag_2CO_3$ (0.06 mmol, 0.5 equiv.) and 4 mg of PivOH (0.04 mmol, 30 mol%) were added. After 3 cycles of vacuum/nitrogen, ethyl 2-fluoro-2-(4-iodophenyl)acetate (0.12 mmol, 1.0 eq.) and DMA (2 mL) were added. The tube was closed and stirred at 100 °C overnight. The reaction was cooled, diluted with a solution of saturated LiCl and extracted with $Et_2O$ (3 x 10 mL), dried over $MgSO_4$. Solvent was removed under reduced pressure and the corresponding crude compound was purified using column chromatography (hexane/EtOAc = 7:3).

**Physical appearance:** Colourless oil (yield: 43%). **$^1$H NMR** (500 MHz, $CDCl_3$) δ 8.00 – 7.96 (m, 2H), 7.58 – 7.52 (m, 4H), 7.34 – 7.30 (m, 2H), 6.84 (s, 1H), 5.84 (d, *J* = 47.5 Hz, 1H), 4.36 – 4.25 (m, 2H), 3.10 (s, 3H), 1.31 (t, *J* = 7.1 Hz, 3H); **$^{13}$C NMR** (126 MHz, $CDCl_3$) δ 167.91 (d, *J* = 26.8 Hz), 144.98 – 144.30 (m), 144.24, 143.15, 140.19, 135.84 (d, *J* = 20.6 Hz), 129.68, 129.16, 128.70, 127.15 (d, *J* = 6.5 Hz), 125.73, 120.86 (q, *J* = 269.3 Hz), 107.13, 88.65 (d, *J* = 187.3 Hz), 62.22, 44.49, 14.09; **$^{19}$F NMR** (376 MHz, $CDCl_3$) δ -62.55 (s), -182.97 (d, *J* = 47.7 Hz). **IR** (film, $cm^{-1}$) υ 2985, 1756, 1151, 1096; **HRMS** (ESI) *m/z* calculated for: $C_{21}H_{18}F_4N_2O_4SNa$ $[M+ Na]^+$, 493.08156, found, 493.08145.

**2-Fluoro-2-(4-(1-(4-(methylsulfonyl)phenyl)-3-(trifluoromethyl)-1H-pyrazol-5-yl)phenyl)acetic acid**

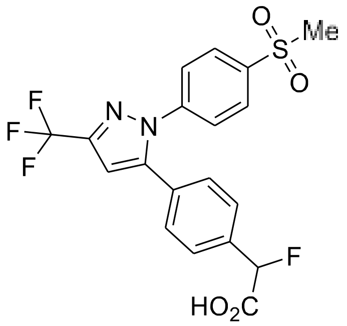

Ethyl 2-fluoro-2-(4-(1-(4-(methylsulfonyl)phenyl)-3-(trifluoromethyl)-1H-pyrazol-5-yl)phenyl)acetate (42 mg, 0.1 mmol, 1 equiv.) was dissolved in a 2:1 MeOH and aqueous $K_2CO_3$ (5 equiv.) (5 mL) mixture before it was left to stir at r.t until all the ester had been consumed (determined by TLC). The resulting mixture was then acidified using HCl (5 M) to pH = 2. The aqueous layer was then extracted with $Et_2O$ (3 x 5 mL) and washed with Brine (2 x 5 mL). The organic extracts were dried using $MgSO_4$ and were then concentrated under vacuum and washed with pentane, resulting in the titled compound in 76% yield.

**Physical appearance:** White solid (yield 76%). **Mp =** 158 °C. **$^1$H NMR** (400 MHz, Methanol-*d*$_4$) δ 7.97 – 7.85 (m, 2H), 7.55 – 7.46 (m, 2H), 7.42 (dd, *J* = 8.2, 1.2 Hz, 2H), 7.32 – 7.20 (m, 2H), 5.79 (d, *J* = 47.7 Hz, 1H), 3.06 (s, 3H); **$^{13}$C NMR** (101 MHz, Methanol-*d*$_4$) δ 170.22 (d, *J* = 26.8 Hz), 144.89, 143.97 (t, *J* = 38.5 Hz), 143.08, 140.71, 136.65 (d, *J* = 20.5 Hz), 129.61 (d, *J* = 2.1 Hz), 129.11, 128.41, 126.96 (d, *J* = 6.1 Hz), 125.94, 122.52, 119.86, 106.43, 88.50 (d, *J* = 183.4 Hz), 42.77; **$^{19}$F NMR** (376 MHz, Methanol-*d*$_4$) δ -63.94, -181.97 (d, *J* = 48.4 Hz). **IR** (film, cm$^{-1}$) υ 2919, 2361, 1749, 1136, 1095. **HRMS** (ESI) *m/z* calculated for $C_{19}H_{14}F_4N_2O_4SNa$ $[M+Na]^+$, 465.05026, found, 465.05000.

*Synthesis of (difluoromethyl)arenes.*

**4-(Difluoromethyl)-1,1'-biphenyl**[3]

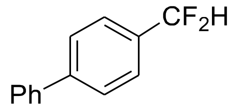


The title compound was prepared following General Procedure GP3 using 4-(difluoromethyl)-1,1'-biphenyl (55 mg, 0.3 mmol), DAST (73 mg, 0.45 mmol) and ethanol (few drops), in DCM (0.5 M). The title compound (48 mg, 78% yield) was isolated as a white solid.

**Physical appearance:** White solid (yield: 78 %). **$^1$H NMR** (400 MHz, $CDCl_3$**)** $\delta$: 7.68 (d, $J$ = 8.0 Hz, 2H), 7.64 – 7.55 (m, 4H), 7.47 (dd, $J$ = 8.4, 6.8 Hz, 2H), 7.44 – 7.34 (m, 1H), 6.70 (t, $J$ = 56.2 Hz, 1H); **$^{19}$F NMR** (376 MHz, $CDCl_3$) $\delta$ -110.35 (d, $J$ = 55.9 Hz). The physical data were identical in all respects to those previously reported.[3]

**1-(Difluoromethyl)-4-phenoxybenzene**[6]

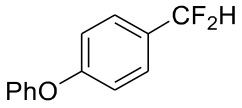


The title compound was prepared following General Procedure GP3 using 4-phenoxybenzaldehyde (59 mg, 0.3 mmol), DAST (73 mg, 0.45 mmol) and ethanol (few drops), in DCM (0.5 M). The title compound (28 mg, 43% yield) was isolated as a colourless oil.

**Physical appearance:** Colourless Oil (yield: 43%). **$^1$H NMR** (400 MHz, $CDCl_3$**)** $\delta$: 7.39 (d, $J$ = 8.2 Hz, 2H), 7.30 (t, $J$ = 7.7 Hz, 2H), 7.09 (t, $J$ = 7.4 Hz, 1H), 6.97 (d, $J$ = 8.1 Hz, 4H), 6.55 (t, $J$ = 58.3 Hz, 1H); **$^{13}$C NMR** (100 MHz, $CDCl_3$) $\delta$: 159.6, 156.2, 130.0, 128.9 (t, $J$ = 22.4 Hz) 127.3 (t, $J$ = 6.1 Hz), 124.1, 119.6, 118.2, 114.6 (t, $J$ = 235.8 Hz); **$^{19}$F NMR** (376 MHz, $CDCl_3$) $\delta$: -109.0 (d, $J$ = 56.4 Hz). The physical data were identical in all respects to those previously reported.[6]

**1-(Difluoromethyl)-3,5-dimethylbenzene**[7]

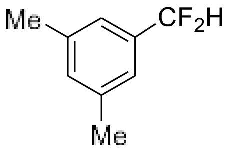


The title compound was prepared following General Procedure GP3 using 3,5-dimethylbenzaldehyde (40 mg, 0.3 mmol), DAST (73 mg, 0.45 mmol) and ethanol (few drops), in DCM (0.5 M). The title compound (25 mg, 54%) was isolated as a yellow oil

**Physical appearance:** Yellow Oil (yield: 54%). **$^1$H NMR** (400 MHz, $CDCl_3$) δ 7.12 (d, *J* = 5.4 Hz, 3H), 6.58 (t, *J* = 56.6 Hz, 1H), 2.37 (s, 6H); **$^{19}$F NMR** (376 MHz, $CDCl_3$) δ -110.17 (d, *J* = 56.6 Hz); **$^{13}$C NMR** (101 MHz, $CDCl_3$) δ 138.44, 132.27 (t, *J* = 2.1 Hz), 123.20 (t, *J* = 6.0 Hz), 114.99 (t, *J* = 238.4 Hz), 21.23. The physical data were identical in all respects to those previously reported.[7]

**2-(Difluoromethyl)-1,4-dimethylbenzene**[6]

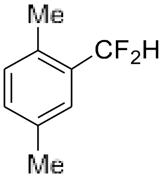


The title compound was prepared following General Procedure GP3 using 3,5-dimethylbenzaldehyde (40 mg, 0.3 mmol), DAST (73 mg, 0.45 mmol) and ethanol (few drops), in DCM (0.5 M). The title compound (20 mg, 43%) was isolated as yellow oil.

**Physical appearance:** Yellow oil (yield: 43%). **$^1$H NMR** (400 MHz, $CDCl_3$) δ 7.35 (s, 1H), 7.23 – 7.17 (m, 1H), 7.14 (d, *J* = 7.8 Hz, 1H), 6.76 (t, *J* = 55.6 Hz, 1H), 2.42 (s, 3H), 2.39 (s, 3H); **$^{13}$C NMR** (101 MHz, $CDCl_3$) δ 135.61, 133.01 (t, *J* = 4.6 Hz), 131.99 (t, *J* = 20.6 Hz), 131.21 (d, *J* = 2.2 Hz), 130.97, 126.35 (t, *J* = 7.3 Hz), 114.53 (t, *J* = 237.7 Hz), 20.91, 17.99. **$^{19}$F NMR** (376 MHz, $CDCl_3$) δ -112.87 (d, *J* = 56.0 Hz); The physical data were identical in all respects to those previously reported.[6]

**1-(*tert*-Butyl)-4-(difluoromethyl)benzene[6]**

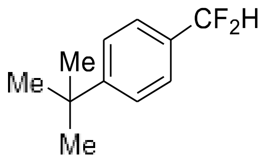


The title compound was prepared following General Procedure GP3 using 4-(tert-butyl)benzaldehyde (49 mg, 0.3 mmol), DAST (73 mg, 0.45 mmol) and ethanol (few drops), in DCM (0.5 M). The title compound (29 mg, 52%) was isolated as a colourless oil.

**Physical appearance:** Colourless oil (yield: 52%). **$^1$H NMR** (400 MHz, $CDCl_3$) δ 7.58 – 7.41 (m, 4H), 6.66 (t, *J* = 56.6 Hz, 1H), 1.36 (s, 9H); **$^{19}$F NMR** (376 MHz, $CDCl_3$) δ -109.87 (d, *J* = 56.6 Hz). The physical data were identical in all respects to those previously reported.[6]

**1-(Difluoromethyl)-2-methoxybenzene[3]**

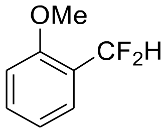


The title compound was prepared following General Procedure GP3 using 2-methoxybenzaldehyde (41 mg, 0.3 mmol), DAST (73 mg, 0.45 mmol) and ethanol (few drops), in DCM (0.5 M). The title compound (19 mg, 40%) was isolated as a yellow oil.

**Physical appearance: Y**ellow oil (yield: 40%). **$^1$H NMR** (400 MHz, $CDCl_3$): δ = 7.59 (d, *J* = 7.3 Hz, 1H), 7.45 (t, *J* = 7.4 Hz, 1H), 7.06 (t, *J* = 7.4 Hz, 1H), 6.99 (t, *J* = 55.8 Hz, 1H), 6.96 (d, *J* = 8.5 Hz, 1H), 3.88 (s, 3H); **$^{13}$C NMR (**101 MHz, $CDCl_3$): δ =157.2 (t, *J* = 6.0 Hz), 131.9 (t, *J* = 1.8 Hz), 126.1 (t, *J* = 6.0 Hz), 122.6 (t, *J* = 22.2 Hz), 120.5, 111.6 (t, *J* = 234.9 Hz), 110.8, 55.5; **$^{19}$F NMR** (376 MHz, $CDCl_3$): δ = -115.3 (d, *J* = 55.9 Hz). The physical data were identical in all respects to those previously reported.[3]

**1-(Difluoromethyl)-3-methoxybenzene[3]**

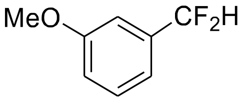


The title compound was prepared following General Procedure GP3 using 3-methoxybenzaldehyde (41 mg, 0.3 mmol), DAST (73 mg, 0.45 mmol) and ethanol (few drops), in DCM (0.5 M). The title compound (24 mg, 50%) was isolated as a colourless oil.

**Physical appearance:** Colourless Oil (yield = 50%) **$^1$H NMR** (400 MHz, $CDCl_3$) δ: δ = 7.38 (t, *J* = 7.9 Hz, 1H), 7.10 (d, *J* = 7.6 Hz, 1H), 7.06 (s, 1H), 7.03 (d, *J* = 8.4 Hz, 1H), 6.63 (t, *J* = 56.5 Hz, 1H), 3.85 (s, 3H); **$^{13}$C NMR** (101 MHz, $CDCl_3$): δ = 159.8, 135.7 (t, *J* = 22.0 Hz), 129.8, 117.8 (t, *J* = 6.2 Hz), 116.6 (t, *J* = 1.8 Hz), 114.6 (t, *J* = 239.2 Hz), 110.7 (t, *J* = 5.9 Hz), 55.3; **$^{19}$F NMR** (376 MHz, $CDCl_3$): δ = -110.7 (d, *J* = 55.9 Hz). The physical data were identical in all respects to those previously reported.[3]

**1-(Difluoromethyl)-4-(trifluoromethyl)benzene[6]**

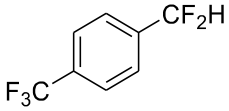


The title compound was prepared following General Procedure GP3 using 4-(trifluoromethyl)benzaldehyde (52 mg, 0.3 mmol), DAST (73 mg, 0.45 mmol) and ethanol (few drops), in DCM (0.5 M). The title compound (36 mg, 61%) was isolated as a colourless oil.

**Physical appearance:** Colourless oil (yield: 61%) **$^1$H NMR** (400 MHz, $CDCl_3$) δ 7.65 (d, *J* = 8.1 Hz, 2H), 7.56 (d, *J* = 8.1 Hz, 2H), 6.62 (t, *J* = 56.0 Hz, 1H); **$^{13}$C NMR** (101 MHz, $CDCl_3$) δ 137.79 (d, *J* = 1.4 Hz), 132.85 (d, *J* = 32.7 Hz), 126.08 (t, *J* = 6.1 Hz), 125.78 (q, *J* = 3.8 Hz), 122.61 (q, *J* = 271.5 Hz), 113.70 (t, *J* = 240.0 Hz); **$^{19}$F NMR** (376 MHz, $CDCl_3$) δ -63.04 (s), -112.37 (d, *J* = 55.8 Hz); The physical data were identical in all respects to those previously reported.[6]

**5-(Difluoromethyl)-2-methoxybenzaldehyde**

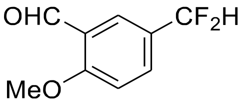


To a mixture of 2-bromo-4-(difluoromethyl)-1-methoxybenzene (450 mg, 1.9 mmol) in THF (20 mL) at -78 °C was added *n*BuLi (1.03 mL, 2.47 mmol, 2.4 M in hexanes). The mixture was stirred at -78 °C for 30 min and then DMF (208 mg, 2.85 mmol) was added. The mixture was stirred at -78 °C for an additional 30 min and then quenched with saturated $NH_4Cl$ (20 mL). The mixture was extracted with EtOAc (2x) and the combined organic layers were washed with brine (2x), dried over anhydrous $Na_2SO_4$, filtered and concentrated under reduced pressure. The crude residue was purified by silica gel flash chromatography eluting with petroleum ether/ethyl acetate to yield the title compound as a white solid (150 mg, 42% yield).

**Physical appearance:** White solid (yield: 42%). **$^1$H NMR** (400 MHz, $CDCl_3$) δ 10.40 (s, 1H), 7.89 (dt, *J* = 2.5, 1.2 Hz, 1H), 7.65 (ddt, *J* = 8.8, 2.3, 1.1 Hz, 1H), 7.01 (d, *J* = 8.7 Hz, 1H), 6.56 (t, *J* = 56.4 Hz, 1H), 3.91 (s, 3H); **$^{13}$C NMR** (101 MHz, $CDCl_3$) δ 188.87, 163.17, 132.82 (t, *J* = 5.4 Hz), 127.04, 126.39 (t, *J* = 6.3 Hz), 124.62, 116.47, 114.10, 112.14, 111.73, 56.00. **$^{19}$F NMR** (376 MHz, $CDCl_3$) δ -109.59 (d, *J* = 56.7 Hz). **IR** (film, $cm^{-1}$) υ 1980, 1461, 1257, 1180.

**4-(Difluoromethyl)dibenzo[b,d]furan[6]**

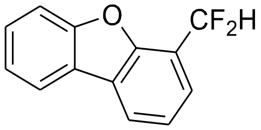


The title compound was prepared following General Procedure GP3 using dibenzo[b,d]furan-4-carbaldehyde (59 mg, 0.3 mmol), DAST (73 mg, 0.45 mmol) and ethanol (few drops), in DCM (0.5 M). The title compound (29 mg, 45%) was isolated as a colourless oil.

**Physical appearance:** Colourless oil (yield: 45%). **$^1$H NMR** (400 MHz, $CDCl_3$**)** *δ*: 7.91 (d, 1H, *J* = 7.6 Hz), 7.83 (d, 1H), 7.55 (dd, 1H, *J* = 7.6 Hz, *J* = 1.1 Hz), 7.53 (d, 1H *J* = 7.6 Hz), 7.47 – 7.30 (td, 1H, *J* = 8.3 Hz, *J* = 1.1 Hz), 7.33 – 7.21 (m, 1H), 7.11 (t, 1H, *J* = Hz); **$^{19}$F NMR** (376 MHz, $CDCl_3$) *δ*: -113.01 (d, *J* = 54.9 Hz); **$^{13}$C NMR** (100 MHz, $CDCl_3$) *δ*: 156.33, 153.03 (t, *J* = 5.2 Hz), 127.83, 125.17, 123.72 (t, *J* = 6.2 Hz), 123.37, 123.25, 123.10 (t, *J* = 1.6 Hz), 122.74, 120.79, 118.47 (t, *J* = 23.3 Hz), 111.95, 111.91 (t, *J* = 236.6 Hz). The physical data were identical in all respects to those previously reported.[6]

**2-(3-(Difluoromethyl)phenoxy)-5-(trifluoromethyl)pyridine**

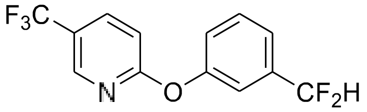


To trifluoromethyl)pyridin-2-yl)oxy)benzaldehyde (0.3 g, 1.12 mmol) in $CH_2Cl_2$ (4 mL) was added Deoxo-Fluor (497 mg, 2.25 mmol) and heated at 40 °C for 16 hours. Starting material aldehyde remained and therefore the reaction mixture was charged with additional Deoxo-Fluor (497 mg, 2.25 mmol) and heated at 40 °C for 6 hours, followed by an additional addition of Deoxo-Fluor (497 mg, 2.25 mmol) and heating at 40 °C for 16 hours. Saturated $NaHCO_3$ was added and the mixture was extracted with $CH_2Cl_2$ (2x) and the combined organic fraction was concentrated under reduced pressure. The crude residue was purified by silica gel flash chromatography eluting with petroleum ether/ethyl acetate, followed by purification by preparative reverse-phase HPLC (Column: Gemini-C18, 100*21.2 mm 5 µm; Mobile phase: MeCN-$H_2O$ (0.1% FA); Gradient: 55% to 65%; Flow rate: 25 ml/min) to yield the title compound as a yellow oil (108 mg, 33% yield).

**Physical appearance:** Yellow oil (yield: 33%). **$^1$H NMR** (400 MHz, $CDCl_3$) *δ*: 8.35 (dt, *J* = 2.6, 1.0 Hz, 1H), 7.85 (dd, *J* = 8.6, 2.5 Hz, 1H), 7.45 (t, *J* = 7.9 Hz, 1H), 7.32 (dp, *J* = 7.8, 1.2 Hz, 1H), 7.21 – 7.17 (m, 1H), 6.98 (dt, *J* = 8.7, 0.8 Hz, 1H), 6.59 (t, *J* = 56.4 Hz, 1H); **$^{13}$C NMR** (101 MHz, $CDCl_3$) δ 165.32, 153.36, 145.40 (q, *J* = 4.4 Hz), 136.90 (q, *J* = 3.2 Hz), 136.20 (t, *J* = 22.7 Hz), 130.22, 123.88 (t, *J* = 1.8 Hz), 123.50 (q, *J* = 271.6 Hz), 122.56 (t, *J* = 6.2 Hz), 118.83 (t, *J* = 6.2 Hz), 116.36, 113.98, 111.61 (d, *J* = 3.1 Hz); **$^{19}$F NMR** (376 MHz, $CDCl_3$) *δ*: -61.71, -111.43 (d, *J* = 56.4 Hz); **IR** (film, $cm^{-1}$) υ 2916, 1448, 1395, 1030. **HRMS** (ESI) *m/z* calculated for $C_{13}H_9F_5NO$ $[M + Na]^+$ 290.05988 Found, 290.05998.

**2-(4-(Difluoromethyl)phenyl)-2H-1,2,3-triazole**

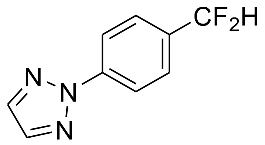


To a mixture of 4-(2*H*-1,2,3-triazol-2-yl)benzaldehyde (400 mg, 2.3 mmol) in $CH_2Cl_2$ (10 mL) was added EtOH (21.3 mg, 0.462 mmol) and Deoxo-Fluor (869 mg, 3.93 mmol) and heated at 40 °C for 18 hours. The reaction mixture was washed with water and brine, dried over anhydrous $Na_2SO_4$, filtered and concentrated under reduced pressure. The crude residue was purified by silica gel flash chromatography eluting with petroleum ether/ethyl acetate to yield the title compound as a white solid (131 mg, 29% yield).

**Physical appearance:** White solid (yield: 29%). $^{1}$**H NMR** (400 MHz, $CDCl_3$) δ 8.12 (d, *J* = 7.9, 2H), 7.78 (s, 2H), 7.57 (t, *J* = 8.7 Hz, 2H), 6.63 (s, 1H); $^{13}$**C NMR** (101 MHz, $CDCl_3$) δ 136.05, 133.37, 126.82 (t, *J* = 6.1 Hz), 119.04, 114.20 (t, *J* = 239.1 Hz). $^{19}$**F NMR** (376 MHz, $CDCl_3$) δ -110.77 (d, *J* = 56.3 Hz).

**1-(3-(Difluoromethyl)phenyl)-3,5-dimethyl-1H-pyrazole**

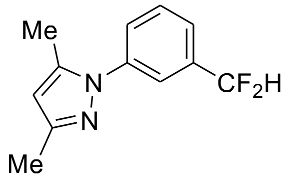


To a mixture of 3-(3,5-dimethyl-1*H*-pyrazol-1-yl)benzaldehyde (200 mg, 1 mmol) in $CH_2Cl_2$ (10 mL) was added EtOH (9.2 mg, 0.20 mmol) and Deoxo-Fluor (376 mg, 1.70 mmol) and heated at 40 °C for 18 hours. The reaction mixture was washed with water and brine, dried over anhydrous $Na_2SO_4$, filtered and concentrated under reduced pressure. The crude residue was purified by silica gel flash chromatography eluting with petroleum ether/ethyl acetate to yield the titled compound as a yellow oil (88 mg, 40% yield).

**Physical appearance:** Yellow oil (yield: 40%). $^{1}$**H NMR** (400 MHz, $CDCl_3$) δ 7.57 – 7.51 (m, 1H), 7.49 – 7.37 (m, 3H), 6.60 (t, *J* = 56.3 Hz, 1H), 5.94 (s, 1H), 2.24 (d, *J* = 0.8 Hz, 3H), 2.22 (s, 3H); $^{19}$**F NMR** (376 MHz, $CDCl_3$) δ -111.30 (d, *J* = 56.2 Hz); $^{13}$**C NMR** (101 MHz, $CDCl_3$) δ 149.53, 140.35, 139.49, 135.48 (t, *J* = 22.7 Hz), 129.49, 126.46 (t, *J* = 1.9 Hz), 124.05 (t, *J* = 6.0 Hz), 121.79 (t, *J* = 6.3 Hz), 117.62 – 110.90 (m), 107.56, 13.49,12.46; **IR** (film, $cm^{-1}$) υ 2982, 1452, 1275, 1059, 1024. **HRMS** (ESI) *m/z* calculated for $C_{12}H_{13}F_2N_2$ $[M + H]^+$, 223.10413, Found 223.10418.

**Isopropyl 2-(4-(4-(difluoromethyl)benzoyl)phenoxy)-2-methylpropanoate[8]**

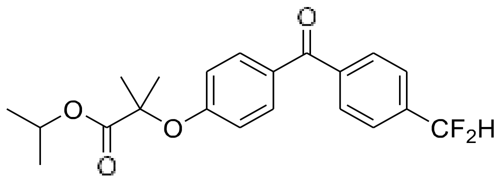


The title compound was prepared following General Procedure GP 4 using isopropyl 2-(4-(4-(difluoromethyl)benzoyl)phenoxy)-2-methylpropanoate (37.6 mg, 0.1 mmol, 1.0 equiv.), Mn(tmp)Cl (2.5 mol%), PhIO (0.33 mmol, 3.3 equiv.) $Et_3N{\cdot}3HF$ (0.12 mmol, 1.2 equiv.) and DCE (1 mL) and purified using column chromatography (DCM/*n*-pentane)

**Physical appearance:** White solid (yield: 22%). **$^1$H NMR** (400 MHz, $CDCl_3$) δ 7.77 (d, *J* = 7.9 Hz, 2H), 7.71 (d, *J* = 8.5 Hz, 2H), 7.56 (d, *J* = 7.6 Hz, 2H), 6.85 (d, *J* = 8.5 Hz, 2H), 6.67 (t, *J* = 56.2 Hz, 1H), 5.56-4.36 (m, 1H), 1.62 (s, 6H), 1.18 (d, *J* = 6.1 Hz, 6 H); **$^{19}$F NMR** (376 MHz, $CDCl_3$) δ -111.64 (d, *J* = 56.1 Hz). The physical data were identical in all respects to those previously reported.[8]

**5-(4-(Difluoromethyl)phenyl)-2-methoxypyridine**

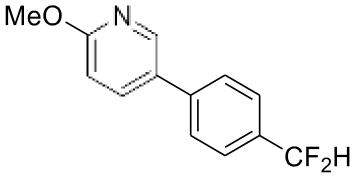


The title compound was prepared following General Procedure GP 4 using 2-fluoro-2-(4-(6-methoxypyridin-3-yl)phenyl)acetic acid (26.1 mg, 0.1 mmol, 1.0 equiv.), Mn(tmp)Cl (2.5 mol%), PhIO (0.33 mmol, 3.3 equiv.) $Et_3N{\cdot}3HF$ (0.12 mmol, 1.2 equiv.) and DCE (1 mL) and purified using column chromatography (DCM/*n*-pentane)

**Physical appearance:** White Solid (yield: 21%). **Mp**: 59 – 62 °C; **$^1$H NMR** (400 MHz, Methanol-*d*$_4$) δ 8.47 – 8.27 (m, 1H), 7.96 (dd, *J* = 8.7, 2.6 Hz, 1H), 7.70 (d, *J* = 8.6 Hz, 2H), 7.61 (d, *J* = 8.2 Hz, 2H), 6.89 (d, *J* = 8.6 Hz, 1H), 6.80 (t, *J* = 52.6 Hz, 1H), 3.95 (s, 3H); **$^{13}$C NMR** (101 MHz, Methanol-*d*$_4$) δ 165.56, 146.12, 141.49, 139.13, 135.22 (t, *J* = 22.4 Hz), 130.64, 129.53 – 125.64 (m), 116.36 (t, *J* = 236.6 Hz), 111.94, 54.31; **$^{19}$F NMR** (376 MHz, Methanol-*d*$_4$) δ -111.83 (d, *J* = 56.3 Hz); **IR** (film, $cm^{-1}$) υ 3010, 1402, 1256, 1120. **HRMS** (ESI) *m/z* calculated for $C_{13}H_{12}F_2NO$ $[M + H]^+$, 236.08924, Found, 236.08813.

**2-(Difluoromethyl)-6-methoxynaphthalene[9]**

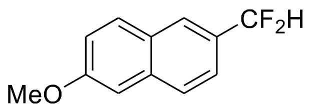

A dried Schlenk tube was charged with (0.2 mmol), PDFA (356 mg, 1.0 mmol), 1,3-Cyclopendione (19.6 mg, 0.2 mmol), $Ca(OH)_2$ (59.3 mg, 0.8 mmol), $Pd(PPh_3)_4$ (46.2 mg, 0.04 mmol), $H_2O$ (9 mg, 0.5 mmol) and p-xylene (2 mL). The mixture was stirred at 90 °C for 3 h under $N_2$ atmosphere. After being cooled to room temperature, the mixture was subjected to flash column chromatography (petroleum ether / dichloromethane) to afford the pure product as a white solid.

**Physical appearance:** White solid (yield: 42%). $^1$H **NMR** (200 MHz, $CDCl_3$) δ 7.95 – 7.76 (m, 3H), 7.57 (dd, *J* = 8.5, 1.8 Hz, 1H), 7.26 – 7.15 (m, 2H), 6.78 (t, *J* = 56.5 Hz, 1H), 3.95 (s, 3H); **$^{19}$F NMR** (376 MHz, $CDCl_3$) δ -109.13 (d, *J* = 56.5 Hz). The physical data were identical in all respects to those previously reported.[9]

**1-(Difluoromethyl)naphthalene[6]**

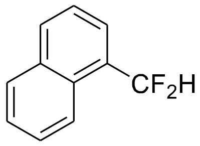

The title compound was prepared following General Procedure GP3 using 1-naphthaldehyde (47 mg, 0.3 mmol). DAST (73 mg, 0.45 mmol) and ethanol (few drops), in DCM (0.5 M). The title compound (27 mg, 55%) was isolated as a white solid.

**Physical appearance:** White solid (yield: 55%). **$^1$H NMR** (400 MHz, $CDCl_3$) δ 8.09 (d, *J* = 8.2 Hz, 1H), 7.88 (d, *J* = 8.3 Hz, 1H), 7.85 – 7.81 (m, 1H), 7.65 – 7.57 (m, 1H), 7.56 – 7.39 (m, 3H), 7.05 (s, 1H); **$^{19}$F NMR** (376 MHz, $CDCl_3$) δ -110.88 (d, *J* = 55.4 Hz). The physical data were identical in all respects to those previously reported.[6]

**5-(4-(Difluoromethyl)phenyl)-1-(4-(methylsulfonyl)phenyl)-3-(trifluoromethyl)-1H-pyrazole[8]**

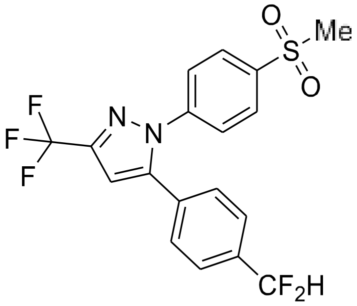


Synthesised according to a reported procedure.[8]

**Physical appearance:** Colourless oil (yield: 20%). **$^1$H NMR** (400 MHz, $CDCl_3$) δ 8.02 – 7.96 (m, 2H), 7.61 – 7.52 (m, 4H), 7.40 – 7.34 (m, 2H), 6.86 (s, 1H), 6.70 (t, 1H), 3.10 (s, 3H); **$^{19}$F NMR** (376 MHz, $CDCl_3$) δ -62.55, -111.83 (d, *J* = 56.3 Hz). The physical data were identical in all respects to those previously reported.[8]

**(8R,9S,13S,14S)-3-(Difluoromethoxy)-13-methyl-6,7,8,9,11,12,13,14,15,16-decahydro-17H-cyclopenta[a]phenanthren-17-one[10]**

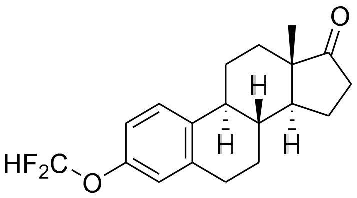


In a 10 mL round-bottomed flask was placed estrone (136 mg, 0.5 mmol, 1.0 equiv.), acetonitrile (1.0 mL) and 6M aqueous KOH (1.0 mL). The mixture was stirred rapidly at r.t and $HCF_2OTf$ (0.21 mL, 1.5 mmol, 3.0 equiv.) was added at once at 0 °C. The mixture was stirred vigorously for 2 minutes, warmed to r.t and stirred for a further 20 minutes. The reaction was diluted with $H_2O$ (10 mL) and extracted with ether (2 x 10 mL). The combined organic layers were dried over $MgSO_4$, concentrated, and purified by silica gel chromatography.

**Physical appearance:** White solid (yield: 43%). **$^1$H NMR** (400 MHz, $CDCl_3$) δ 7.29 (d, *J* = 8.4 Hz, 2H), 6.93 (d, *J* = 8.5, 1H), 6.88 (s , 1H), 6.50 (t, *J* = 74.3 Hz, 1H), 2.94 (dd, *J* = 9.0, 4.3 Hz, 2H), 2.58 – 2.47 (m, 1H), 2.42 – 2.37 (m, 1H), 2.27 (t, *J* = 10.6 Hz, 1H), 2.1 (ddd, *J* = 23.7, 13.3, 5.8 Hz, 2H), 1.98 (d, *J* = 10.4 Hz, 1H), 1.70 – 1.41 (m, 7H), 0.94 (s, 3H).**$^{19}$F NMR** (376 MHz, $CDCl_3$) δ -80.36 (d, *J* = 74.6 Hz). The physical data were identical in all respects to those previously reported.[10]

**Preparation of Boronic Acid Precursors**

Boronic acid precursors used in the synthesis towards 8a-8h, 8j and 8p-8v were purchased from commercial vendors.

Boronic acid precursors used in the synthesis towards 8i and 8k-8m were kindly donated by Pfizer inc.

Boronic acid precursors used in the synthesis of 8n and 8o were synthesized according to known literature procedures.

**(4-(4-((1-isopropoxy-2-methyl-1-oxopropan-2-yl)oxy)benzoyl)phenyl)boronic acid**

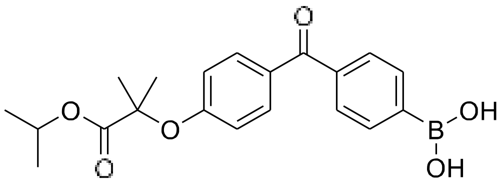


Prepared according to a known literature procedure[11]

**$^1$H NMR** (400 MHz, DMSO-$d_6$) δ 8.30 (s, 2H), 7.94 (d, *J* = 7.6 Hz, 2H), 7.76 – 7.69 (m, 2H), 7.67 – 7.60 (m, 2H), 6.94 – 6.87 (m, 2H), 5.04 – 4.93 (m, 1H), 1.61 (s, 6H), 1.16 (d, *J* = 6.3 Hz, 6H). **HRMS** (ESI, m/z) calcd [M - H]$^-$ for 369.15149, Found, 369.15117. The physical data were identical in all respects to those previously reported.[11] **Note: if $^1$H NMR is taken in $CDCl_3$ peaks corresponding to the boroxine trimer may be observed!**

**(4-(1-(p-tolyl)-3-(trifluoromethyl)-1H-pyrazol-5-yl)phenyl)boronic acid**

Prepared according to a known literature procedure[12]

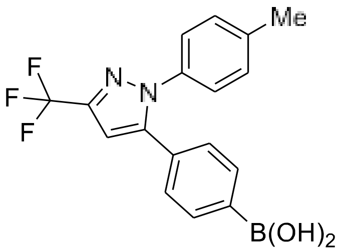


**$^1$H NMR** (400 MHz, DMSO-$d_6$) δ 7.76 (d, *J* = 8.4 Hz, 2H), 7.21 (d, *J* = 8.4 Hz, 2H), 7.07-7.15 (m, 5H), 2.21 (s, 3H); **LRMS**: (ESI$^+$, m/z) 347.1 ([M+H]$^+$, 100%). The physical data were identical in all respects to those previously reported.[12] **Note: if $^1$H NMR is taken in $CDCl_3$ peaks corresponding to the boroxine trimer may be observed!**

### *In Situ* Preparation of Iodine(III) dicarboxylates

In each radiochemical reaction, α-aryl-α-fluoroacetic acid and iodosylbenzene were pre-stirred in acetonitrile, solvent was removed under vacuum *a priori* to $^{18}F$ being dispensed into the reaction vial. To prove that iodine(III) dicarboxylate intermediate is formed during the pre-stirring step, the model radiochemistry substrate 2-fluoro-2-(4-phenoxyphenyl)acetic acid Iodine(III) dicarboxylate was fully characterised. For other substrates where appropriate, the Iodine (III) dicarboxylate intermediate complex formation was confirmed by $^{1}H$ NMR and $^{19}F$ NMR to prove a similar reaction mechanism. Notable shifts of both the benzylic alpha proton and fluorine were observed in $^{1}H$ and $^{19}F$ NMR respectively. Iodine(III) dicarboxylates diastereoisomer have peaks which stem from the stereogenic centre of the benzylic Ar-<u>**C**</u>FH$CO_2H$ carbon which were made in a racemic fashion **(Scheme SI-8).**

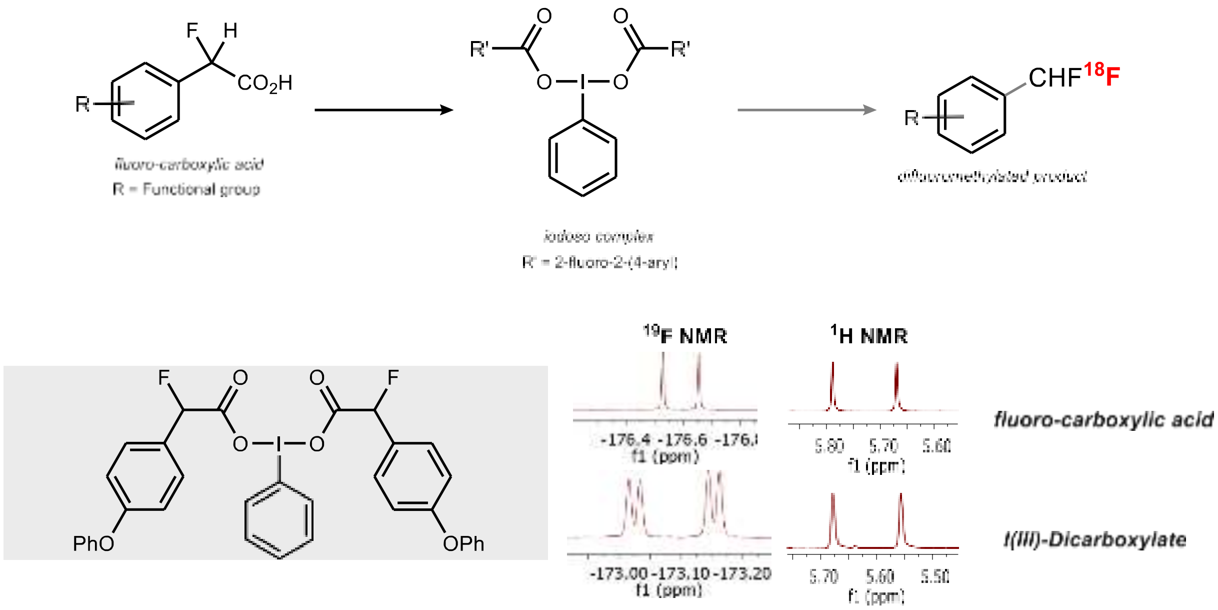


**Scheme SI-8** Synthesis of Iodine(III) dicarboxylates.

**$^{1}$H NMR** (400 MHz, $CDCl_3$) δ 7.96 (d, *J* = 6.7 Hz, 2H), 7.59 (t, *J* = 7.5 Hz, 1H), 7.40 (dt, *J* = 24.6, 8.2 Hz, 8H), 7.29 (s, 2H), 7.16 (t, *J* = 7.4 Hz, 2H), 7.04 (d, *J* = 1.1 Hz, 4H), 6.94 (d, *J* = 8.4 Hz, 4H), 5.70 (d, *J* = 48.0 Hz, 2H); **$^{13}$C NMR** (101 MHz, $CDCl_3$) δ 172.69 (d, *J* = 27.1 Hz), 158.59, 156.58, 134.80, 132.43, 131.33, 130.02, 129.36 (d, *J* = 20.9 Hz), 128.41, 124.01, 122.96, 119.52, 118.60, 88.33 (d, *J* = 189.0 Hz); **$^{19}$F NMR** (376 MHz, $CDCl_3$) -173.83 (d, *J* = 48.1 Hz, 1F), -173.85 (d, *J* = 48.1 Hz, 1F); **HRMS** (ESI) *m/z* calculated for $[M + Na]^+$, 717.05561, Found, 717.05588. **IR** (film, $cm^{-1}$) υ: 3062, 1755, 1677, 1232, 1025.

**Phenyl- λ3-iodanediyl bis(2-([1,1'-biphenyl]-4-yl)-2-fluoroacetate)**

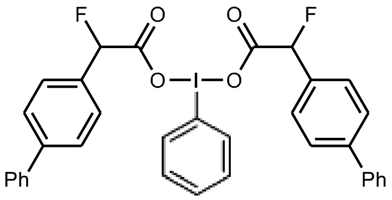


**$^1$H NMR** (400 MHz, $CDCl_3$) δ 7.97 – 7.89 (m, 2H), 7.56 (dd, *J* = 11.0, 7.9 Hz, 10H), 7.46 (t, *J* = 7.5 Hz, 5H), 7.42 – 7.36 (m, 6H), 5.77 (d, 49.0 Hz, 2H). **$^{19}$F NMR** (376 MHz, $CDCl_3$) δ -175.90 (d, *J* = 48.7 Hz, 2H).

**Phenyl- λ3-iodanediyl bis(2-fluoro-2-(4-(trifluoromethyl)phenyl)acetate)**

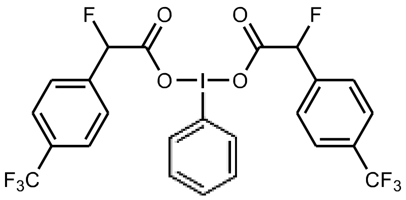


**$^1$H NMR** (400 MHz, Acetonitrile-$d_3$) δ 8.06 – 7.86 (m, 2H), 7.73 – 7.55 (m, 5H), 7.56 – 7.33 (m, 6H), 5.92 (d, *J* = 47.9 Hz, 2H). **$^{19}$F NMR** (376 MHz, Acetonitrile-$d_3$) δ -178.89 (d, *J* = 48.0 Hz).

**Phenyl- λ3-iodanediyl bis(2-fluoro-2-(3-methoxyphenyl)acetate)**

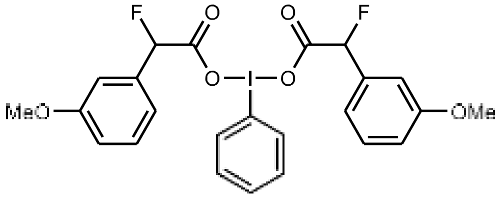


**$^1$H NMR** (400 MHz, $CDCl_3$) δ 7.84 – 7.73 (m, 2H), 7.51 – 7.38 (m, 1H), 7.37 – 7.27 (m, 2H), 7.23 – 7.12 (m, 2H), 6.93 – 6.65 (m, 6H), 5.61 (d, *J* = 48.4 Hz, 2H), 3.68 (s, 6H). **$^{19}$F NMR** (376 MHz, $CDCl_3$) δ -176.59 (d, *J* = 48.7 Hz).

**Phenyl- λ3-iodanediyl bis(2-(3,5-dimethylphenyl)-2-fluoroacetate)**

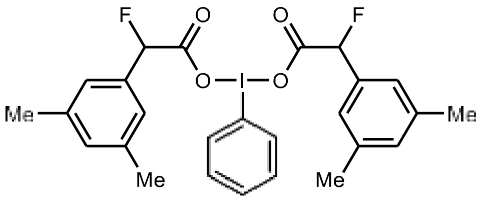


**¹H NMR** (400 MHz, $CDCl_3$) δ 7.83 (d, *J* = 11.2 Hz, 2H), 7.52 – 7.46 (m, 1H), 7.30 (d, *J* = 8.2 Hz, 2H), 6.88 (d, *J* = 20.8 Hz, 6H), 5.56 (d, *J* = 48.7 Hz, 2H), 2.20 (s, 12H). **¹⁹F NMR** (376 MHz, $CDCl_3$) δ -174.65 (d, *J* = 48.7 Hz, 1F) , -174.66 (d, *J* = 48.7 Hz, 1F).

**Phenyl- λ3-iodanediyl bis(2-fluoro-2-(2-methoxyphenyl)acetate)**

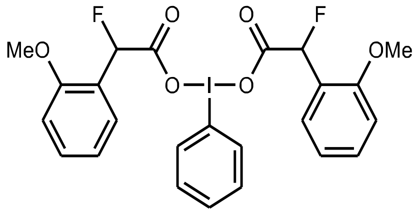


**¹H NMR** (400 MHz, $CDCl_3$) δ 7.96 (s, 2H), 7.56 (t, *J* = 7.4 Hz, 1H), 7.37 (dt, *J* = 21.5, 8.1 Hz, 4H), 7.24 – 7.13 (m, 2H), 7.00 – 6.84 (m, 4H), 6.06 (d, *J* = 48.0 Hz, 2H), 3.77 (s, 6H). **¹⁹F NMR** (376 MHz, $CDCl_3$) δ -173.78 (d, *J* = 47.4 Hz).

**Phenyl- λ3-iodanediyl bis(2-fluoro-2-(6-methoxynaphthalen-2-yl)acetate)**

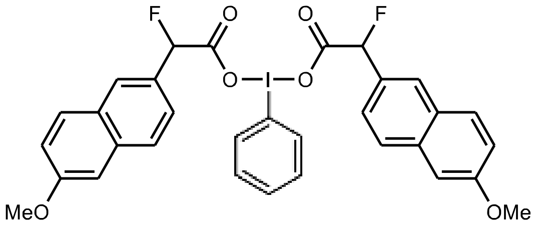


**¹H NMR** (400 MHz, $CDCl_3$) δ 7.70 (dq, *J* = 8.4, 1.9, 1.4 Hz, 2H), 7.60 (ddd, *J* = 16.7, 8.6, 2.3 Hz, 6H), 7.33 – 7.24 (m, 3H), 7.11 – 7.01 (m, 6H), 5.75 (d, *J* = 48.5 Hz, 2H), 3.84 (s, 6H). **¹⁹F NMR** (376 MHz, $CDCl_3$) δ -173.76 (d, *J* = 48.7), -173.75 (d, *J* = 48.6 Hz).

**Phenyl- λ3-iodanediyl bis(2-fluoro-2-(naphthalen-1-yl)acetate)**

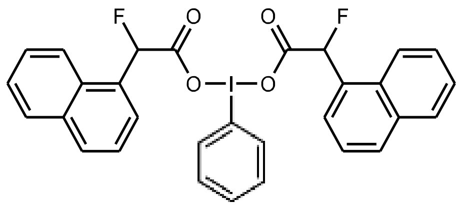


**$^{1}$H NMR** (400 MHz, $CDCl_3$) δ 8.03 (d, *J* = 8.4 Hz, 2H), 7.85 (d, *J* = 8.0 Hz, 4H), 7.46 (ddt, *J* = 33.1, 24.3, 9.9 Hz, 11H), 7.15 – 7.05 (m, 2H), 6.27 (d, *J* = 47.6 Hz, 2H). **$^{19}$F NMR** (376 MHz, $CDCl_3$) δ -174.22 (d, *J* = 47.6 Hz).

**Phenyl- λ3-iodanediyl bis(2-(2,5-dimethylphenyl)-2-fluoroacetate)**

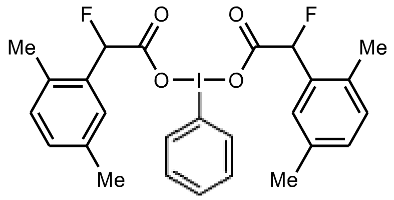


**$^{1}$H NMR** (400 MHz, Acetonitrile-$d_3$) δ 8.03 – 7.96 (m, 1H), 7.72 – 7.60 (m, 1H), 7.56 – 7.42 (m, 2H), 7.38 – 7.19 (m, 1H), 7.22 – 6.97 (m, 6H), 6.07 – 5.82 (d, *J* = 48.2 Hz, 2H), 2.26 (s, 6H), 2.22 (s, 6H), 2.26 (s, 6H). **$^{19}$F NMR** (376 MHz, Acetonitrile-$d_3$) δ -174.41 (d, *J* = 48.1 Hz).

**Phenyl-λ3-iodanediyl bis(2-(4-(2H-1,2,3-triazol-2-yl)phenyl)-2-fluoroacetate)**

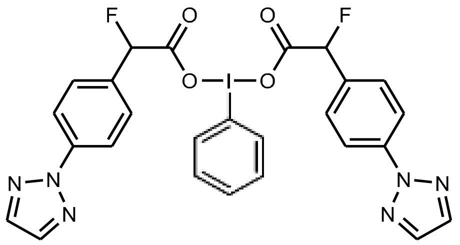


**$^{1}$H NMR** (400 MHz, $CDCl_3$) δ 8.03 (d, *J* = 8.7 Hz, 4H), 7.94 – 7.88 (m, 2H), 7.83 (s, 4H), 7.53 (d, *J* = 15.0 Hz, 1H), 7.46 – 7.34 (m, 6H), 5.77 (d, *J* = 48.3 Hz, 2H). **$^{19}$F NMR** (376 MHz, $CDCl_3$) δ -176.96 (d, *J* = 48.3 Hz, 1F), -176.98 (d, *J* = 48.3 Hz, 1F).

**Phenyl-λ3-iodanediyl bis(2-fluoro-2-(3-((6-(trifluoromethyl)pyridin-3-yl)oxy)phenyl)acetate)**

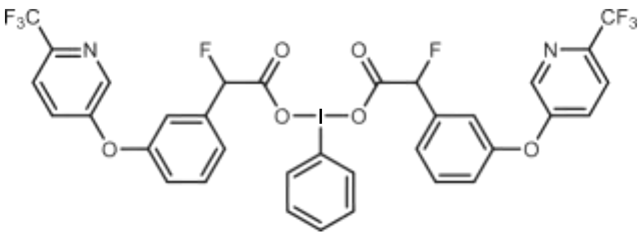

**$^{1}$H NMR** (400 MHz, $CDCl_3$) δ 8.34 (s, 2H), 7.91 – 7.65 (m, 4H), 7.65 – 7.22 (m, 6H), 7.17 – 7.05 (m, 5H), 6.94 (d, *J* = 8.6 Hz, 2H), 5.67 (d, *J* = 48.3 Hz, 2H). **$^{19}$F NMR** (376 MHz, $CDCl_3$) δ -61.64 (s), -178.45 (d, *J* = 48.4 Hz). -178.45 (d, *J* = 48.4 Hz).

**Phenyl-λ3-iodanediyl bis(2-(dibenzo[b,d]furan-4-yl)-2-fluoroacetate)**

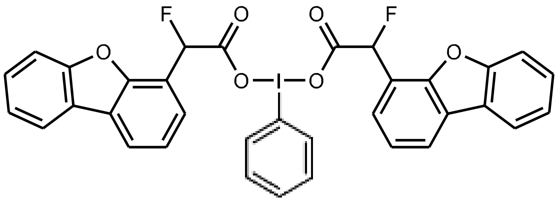

**$^{1}$H NMR** (400 MHz, $CDCl_3$) δ 7.84 (d, *J* = 7.6 Hz, 4H), 7.62 – 7.54 (m, 2H), 7.47 – 7.33 (m, 4H), 7.33 – 7.23 (m, 4H), 7.23 – 7.13 (m, 3H), 6.95 – 6.88 (m, 2H), 6.23 (d, *J* = 47.8 Hz, 2H). **$^{19}$F NMR** (376 MHz, $CDCl_3$) δ -176.02 (d, *J* = 47.8 Hz), -176.04 (d, *J* = 47.8 Hz).

**Diisopropyl 2,2'-(((4,4'-(((phenyl-λ3-iodanediyl)bis(oxy))bis(1-fluoro-2-oxoethane-2,1-diyl))bis(benzoyl))bis(4,1-phenylene))bis(oxy))bis(2-methylpropanoate)**

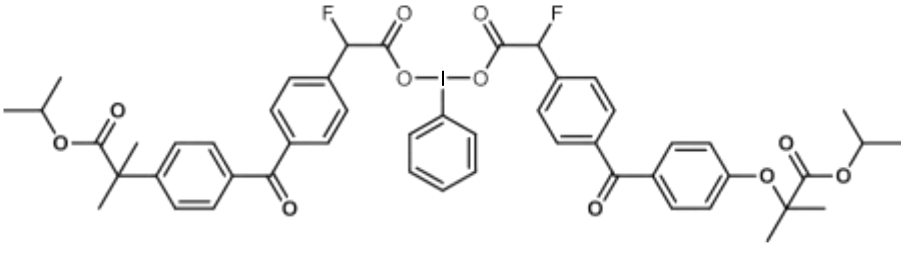

**$^{1}$H NMR** (400 MHz, $CDCl_3$) δ 7.84 (d, *J* = 7.9 Hz, 1H), 7.64 (dd, *J* = 23.7, 8.2 Hz, 8H), 7.51 (t, *J* = 7.0 Hz, 1H), 7.35 (d, *J* = 8.4 Hz, 5H), 6.79 (d, *J* = 8.6 Hz, 4H), 5.73 (d, *J* = 48.3 Hz, 2H), 5.02 (p, *J* = 6.4 Hz, 2H), 1.60 (s, 12H), 1.14 (d, *J* = 6.1 Hz, 12H). **$^{19}$F NMR** (376 MHz, $CDCl_3$) δ -179.56 (d, *J* = 48.3 Hz), -179.58 (d, *J* = 48.3 Hz ).

**Phenyl-λ3-iodanediyl bis(2-fluoro-2-(4-(6-methoxypyridin-3-yl)phenyl)acetate)**

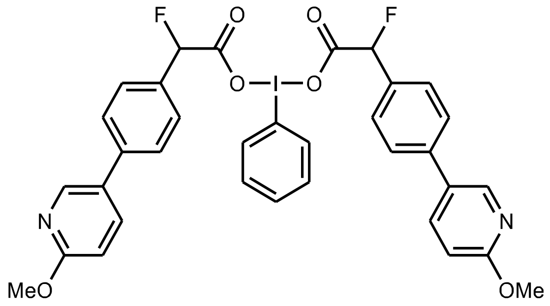

**$^{1}$H NMR** (400 MHz, $CDCl_3$) δ 8.39 (d, *J* = 2.3 Hz, 2H), 7.94 (d, *J* = 8.3 Hz, 2H), 7.78 (dd, *J* = 8.6, 2.5 Hz, 2H), 7.61 – 7.52 (m, 2H), 7.48 (d, *J* = 8.1 Hz, 4H), 7.39 (d, *J* = 8.0 Hz, 5H), 6.84 (d, *J* = 8.6 Hz, 2H), 5.76 (d, *J* = 48.4 Hz, 2H), 3.99 (s, 6H). **$^{19}$F NMR** (376 MHz, $CDCl_3$) δ -176.27 (d, *J* = 48.4 Hz).

**Phenyl-λ3-iodanediyl bis(2-fluoro-2-(((8R,9S,13S,14S)-13-methyl-17-oxo-7,8,9,11,12,13,14,15,16,17-decahydro-6H-cyclopenta[a]phenanthren-3-yl)oxy)acetate)**

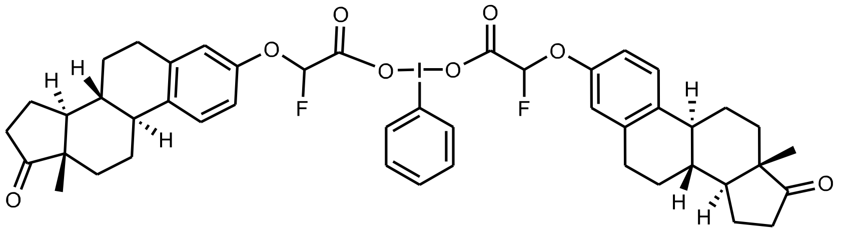

**$^{1}$H NMR** (400 MHz, $CDCl_3$) δ 8.13 – 8.03 (m, 2H), 7.63 – 7.54 (m, 1H), 7.47 (dd, *J* = 8.5, 7.2 Hz, 2H), 7.14 (d, *J* = 8.7 Hz, 2H), 6.81 – 6.62 (m, 4H), 5.80 (dd, *J* = 60.3, 1.1 Hz, 2H), 2.81 (dd, *J* = 8.9, 4.2 Hz, 4H), 2.44 (dd, *J* = 18.9, 8.6 Hz, 2H), 2.36 – 2.27 (m, 2H), 2.19 (d, *J* = 11.1 Hz, 2H), 2.13 – 1.84 (m, 8H), 1.63 – 1.29 (m, 12H), 0.84 (s, 6H). **$^{19}$F NMR** (376 MHz, $CDCl_3$) δ -125.93 (d, *J* = 60.6 Hz), -125.94 (d, *J* = 60.6 Hz)**.**

**Iodine(III) Dicarboxylates Formation Competition Experiment**

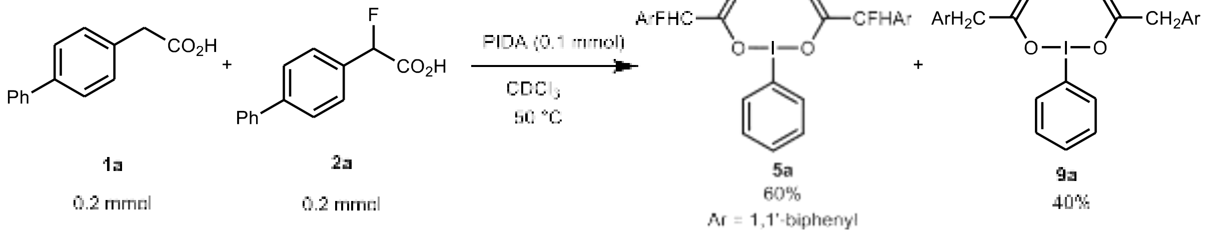


**1a** (0.2 mmol) and **2a** (0.2 mmol) were placed in 4 mL vial with PIDA (0.1 mmol). $CDCl_3$ (2 mL) was added and the reaction was stirred for 20 minutes at 50 °C prior to $^{19}$F NMR analysis.

## S6: Radiochemistry:

[$^{18}$F]Fluoride was produced by Alliance Medical (UK) via the $^{18}$O(p,n)$^{18}$F reaction and delivered as [$^{18}$F]fluoride in $^{18}$O-enriched-water. Radiosynthesis and azeotropic drying was performed on a NanoTek microfluidic device (Advion).

### Procedure for preparation of a solution of [$^{18}$F]TEAF in MeCN:

A solution of tetraethylammonium bicarbonate (9 mg) in 1 mL of MeCN/$H_2O$, 4:1 was freshly prepared. [$^{18}$F]Fluoride (3.0 - 4.0 GBq) was separated from $^{18}$O-enriched-water using a Chromafix PSHCO3 $^{18}$F separation cartridge (45 mg) and subsequently released with 900 µL (in 6 x 150 µL portions) of the tetraethylammonium bicarbonate solution into a 5 mL V-vial containing a magnetic stir bar in the concentrator. The solution was dried with five cycles of azeotropic drying with MeCN (5 x 200 µL) under a flow of $N_2$ at 105 °C. The dried [$^{18}$F]TEAF residue was re-dissolved in anhydrous MeCN (500 - 1000 µL).

### Optimisation

**Table SI-2** Optimisation studies for the [$^{18}$F]fluorodecarboxylation of **8b**

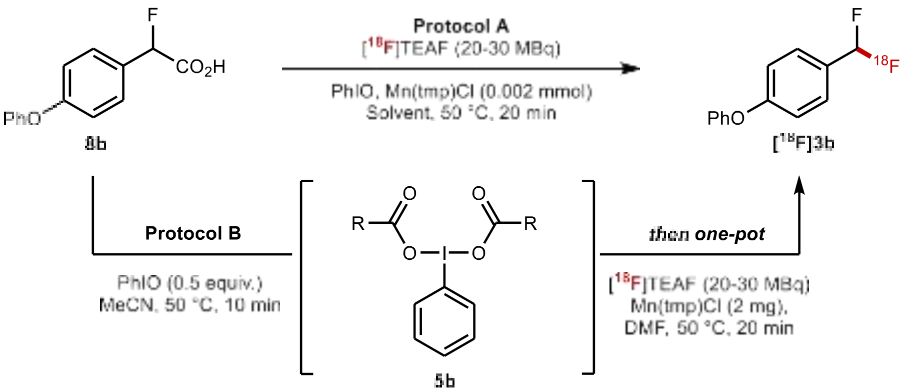


| Entry | Starting Material (mmol) | Protocol | Solvent | PhIO (mmol) | RCC[a] (*n* =2)[b] |
|---|---|---|---|---|---|
| 1 | **8b** (0.11) | **A** | MeCN[c] | 0.33 | 3% ± 1% |
| 2 | **8b** (0.11) | **A** | MeCN | 0.02 | 6% ± 1% |
| 3 | **8b** (0.11) | **A** | DMF | 0.02 | 7% ± 2% |
| 4 | **8b** (0.055) | **A** | DMF[d] | 0.02 | 22% ± 7% |
| **5** | **5b (0.014)** | **B** | **DMF[d]** | **-** | **40% ± 10%[e]** |
| 6 | **5b** (0.014) | **B** | DMF[d] | - | 0% ± 0%[f] |

| | | | | | |
|---|---|---|---|---|---|
| 7 | **8b** (0.014) | **A** | MeCN | 0.02 | 0%± 0%[g] |
| 8 | **5b** (0.014) | **B** | DMF[d] | - | 0% ± 0%[h] |

[a]Radiochemical conversion. [b]*n* = number of reactions. [c]600 µL of MeCN used. [d]MeCN removed at 100 °C after dispensing [$^{18}$F]TEAF. [e](*n* = 10). [f]Reaction Temperature = 100 °C. [g]catalyst: Mn(tmp)OTs. [h]No Mn Catalyst.

## $^{18}$F-fluorodecarboxylation competition experiment

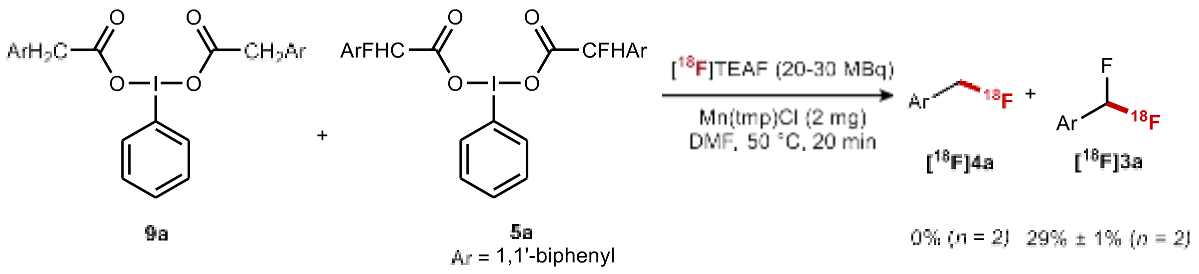


Into a 3 mL vial was weighed **9a** (0.014 mmol), **5a** (0.014 mmol) and manganese catalyst (2 mgs). To this vial was dispensed [$^{18}$F]TEAF (20 – 30 MBq) in a solution of anhydrous MeCN. The MeCN was removed at 100 °C under a flow of nitrogen. Upon cooling, DMF (300 µL) was added to the vial and the reaction was stirred at 50 °C for 20 minutes. The reaction was quenched with water (200 µL) and an aliquot was removed for analysis by radioTLC and radioHPLC for radiochemical conversion and product identity. Analysis was performed using a Waters Nova-Pak C18 column (4 µm, 3.9 x 150 mm) at a flow rate 1 mL/min. Radio-TLC was performed on Merck Kiesegel 60 F254 plates, using DCM/MeOH (9:1) as eluent. Analysis was performed using a plastic scintillator/PMT detector.

## General Procedure for the Small Scale $^{18}$F-Fluorination of substrates [$^{18}$F]3a – [$^{18}$F]3o, [$^{18}$F]3w – [$^{18}$F]3z, [$^{18}$F]11a:

Into a 3 mL vial was weighed substrate (0.014 mmol) and manganese catalyst (2 mgs). To this vial was dispensed [$^{18}$F]TEAF (20 – 30 MBq) in a solution of anhydrous MeCN. The MeCN was removed at 100 °C under a flow of nitrogen. Upon cooling, DMF (300 µL) was added to the vial and the reaction was stirred at 50 °C for 20 minutes. The reaction was quenched with water (200 µL) and an aliquot was removed for analysis by radioTLC and radioHPLC for radiochemical conversion and product identity. Analysis was performed using a Waters Nova-Pak C18 column (4 µm, 3.9 x 150 mm) at a flow rate 1 mL/min. Radio-TLC was performed on Merck Kiesegel 60 F254 plates, using DCM/MeOH (9:1) as eluent. Analysis was performed using a plastic scintillator/PMT detector.

**HPLC gradient A for small scale $^{18}$F-Fluorination of substrates [$^{18}$F]3a – [$^{18}$F]3o, [$^{18}$F]3w – [$^{18}$F]3z, [$^{18}$F]11a:**

Water/MeCN, 1 mL/min, Waters Nova-Pak C18 Column, 4 µm, 3.9 x 150 mm 0 - 1 min (5% MeCN) isocratic 1 - 10 min (5% MeCN to 95% MeCN) linear increase 10 - 14 min (95% MeCN) isocratic 14 - 15 min (95% MeCN to 5% MeCN) linear decrease 15 - 17 min (5% MeCN) isocratic.

**Radio-HPLC of substrates [$^{18}$F]3a – [$^{18}$F]3o, [$^{18}$F]3w – [$^{18}$F]3z, [$^{18}$F]11a**

Crude Radio-HPLC traces of the crude mixture following the general procedure, with authentic UV references overlaid are shown below. The solid black line indicates the UV trace for cold reference material and the solid red line is the crude radio-HPLC trace. All samples were run using HPLC gradient A.

[18F]3a

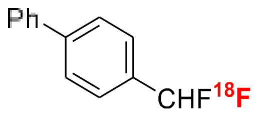


| Reaction | Radio-TLC | Radiochemical Conversion |
|---|---|---|
| 1 | 13% | 10% |
| 2 | 14% | 11% |
| 3 | 28% | 28% |
| 4 | 30% | 30% |
| Radiochemical Conversion + Standard Deviation | | **20% ± 10%** |

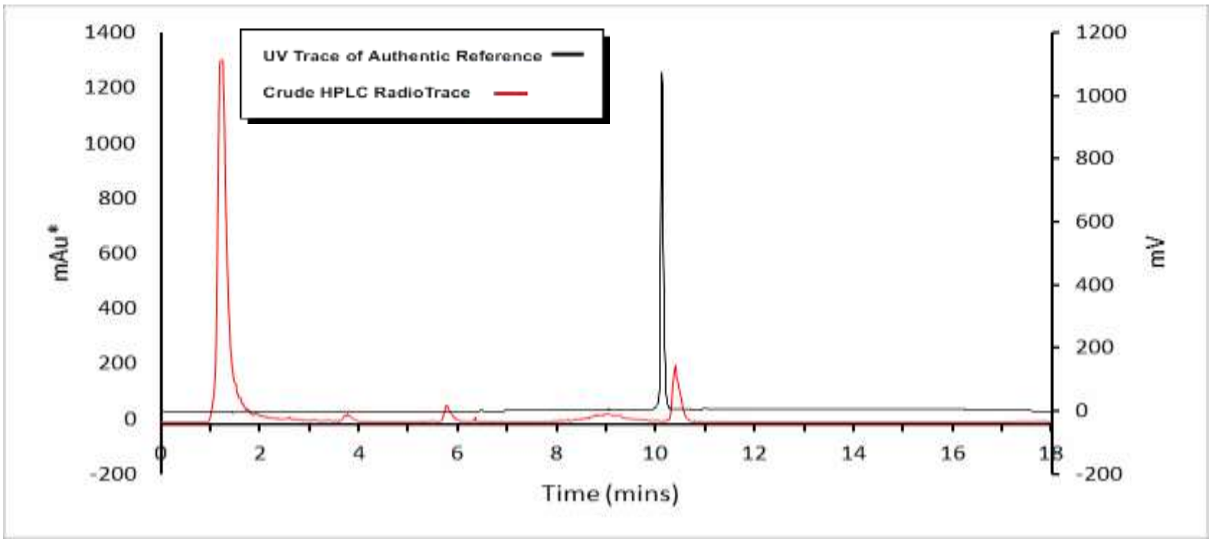


**Figure SI-1** HPLC radio-trace of **[$^{18}$F]3a** (red) overlaid with HPLC UV-trace ($\lambda$ = 220 nm) of $^{19}$F reference compound (black).

[18F]3b

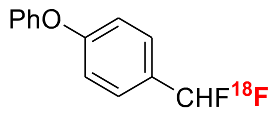


| Reaction | Radio-TLC | Radiochemical Conversion |
|---|---|---|
| 1 | 50% | 50% |
| 2 | 37% | 37% |
| 3 | 45% | 45% |
| 4 | 37% | 37% |
| 5 | 46% | 46% |
| 6 | 53% | 53% |
| 7 | 34% | 34% |
| 8 | 22% | 22% |
| 9 | 30% | 30% |
| 10 | 43% | 43% |
| Radiochemical Conversion + Standard Deviation | | **40% ± 9%** |

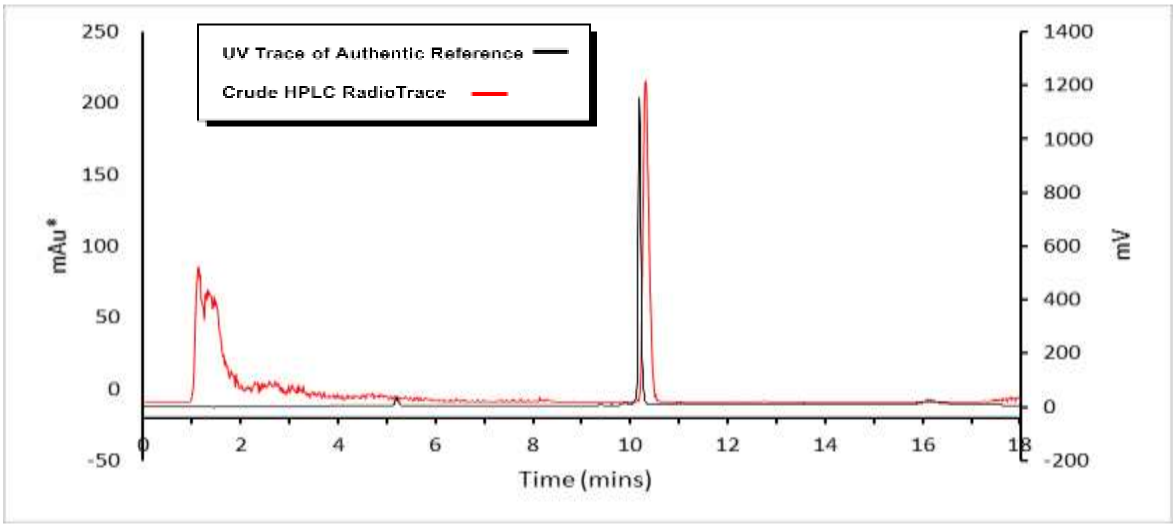


**Figure SI-2** HPLC radio-trace of **[$^{18}$F]3b** (red) overlaid with HPLC UV-trace ($\lambda$ = 220 nm) of $^{19}$F reference compound (black).

[$^{18}$F]3c

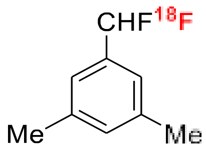


| Reaction | Radio-TLC | Radiochemical Conversion |
|---|---|---|
| 1 | 20% | 20% |
| 2 | 20% | 20% |
| 3 | 21% | 19% |
| 4 | 27% | 26% |
| Radiochemical Conversion + Standard Deviation | | **21% ± 3%** |

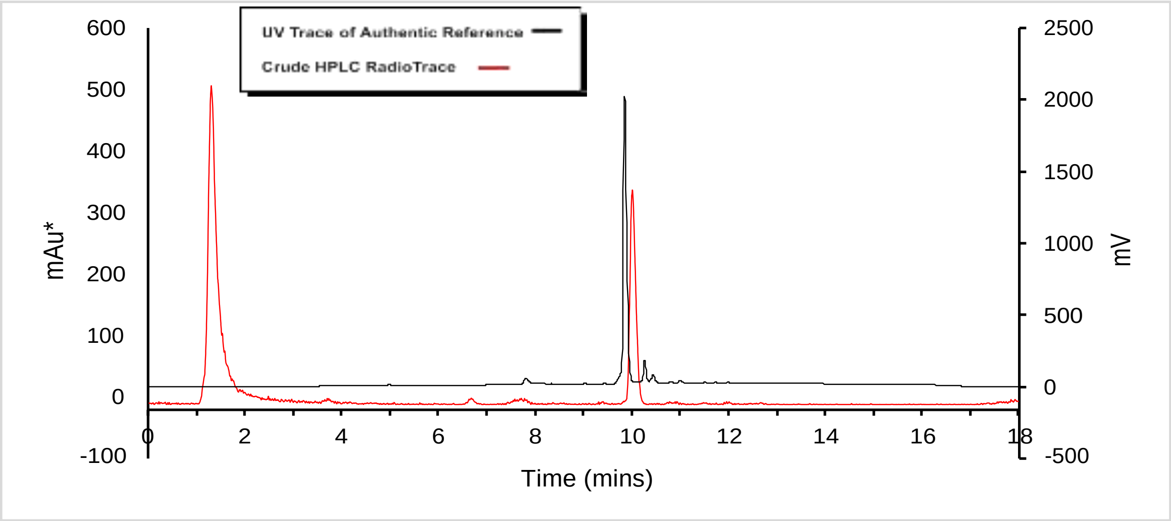


**Figure SI-3** HPLC radio-trace of **[$^{18}$F]3c** (red) overlaid with HPLC UV-trace (λ = 220 nm) of $^{19}$F reference compound (black).

[$^{18}$F]3d

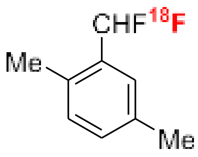


| Reaction | Radio-TLC | Radiochemical Conversion |
|---|---|---|
| 1 | 36% | 34% |
| 2 | 33% | 31% |
| 3 | 42% | 37% |
| 4 | 36% | 32% |
| Radiochemical Conversion + Standard Deviation | | **34% ± 3%** |

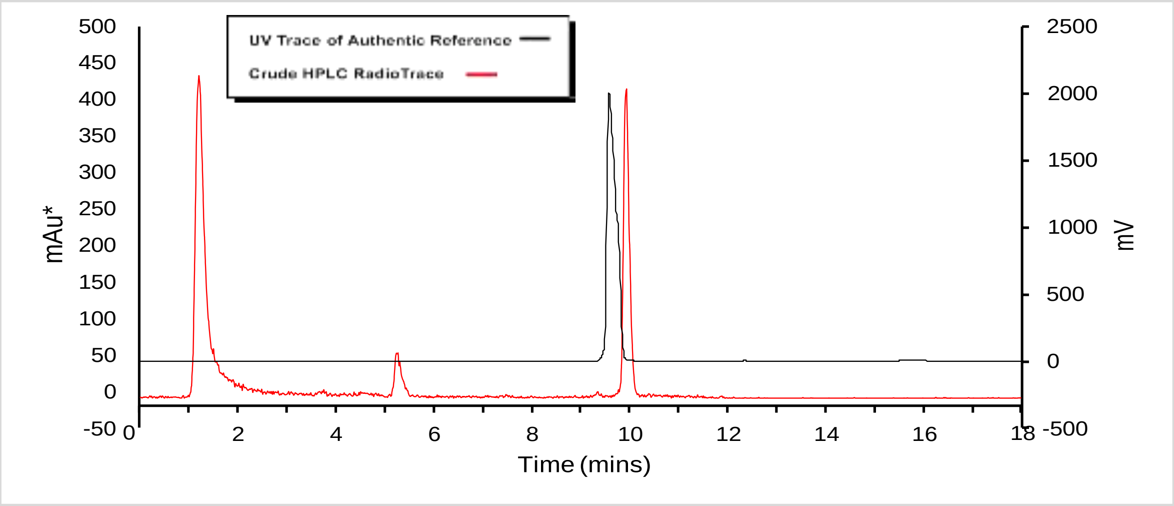


**Figure SI-4** HPLC radio-trace of **[$^{18}$F]3d** (red) overlaid with HPLC UV-trace ($\lambda$ = 220 nm) of $^{19}$F reference compound (black).

[$^{18}$F]3e

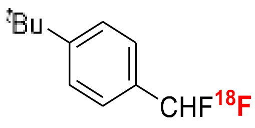


| Reaction | Radio-TLC | Radiochemical Conversion |
|---|---|---|
| 1 | 33% | 33% |
| 2 | 29% | 29% |
| 3 | 13% | 13% |
| 4 | 36% | 36% |
| Radiochemical Conversion + Standard Deviation | | **28% ± 10%** |

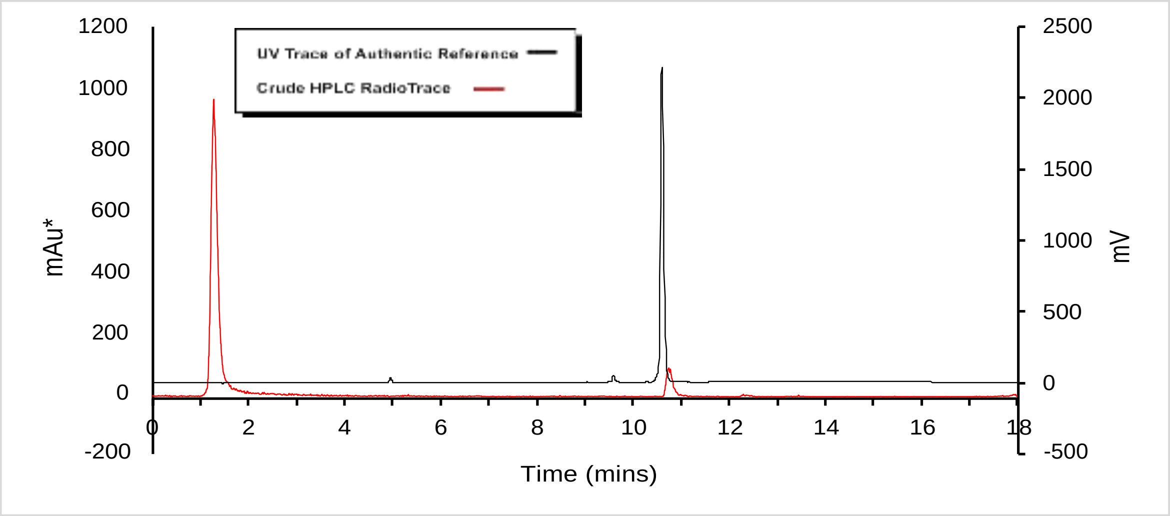


**Figure SI-5** HPLC radio-trace of **[$^{18}$F]3e** (red) overlaid with HPLC UV-trace ($\lambda$ = 220 nm) of $^{19}$F reference compound (black).

[$^{18}$F]3f

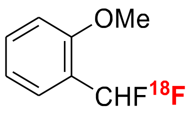


| **Reaction** | **Radio-TLC** | **Radiochemical Conversion** |
|---|---|---|
| 1 | 27% | 23% |
| 2 | 26% | 21% |
| 3 | 15% | 12% |
| 4 | 8% | 7% |
| Radiochemical Conversion + Standard Deviation | | **16% ± 7%** |

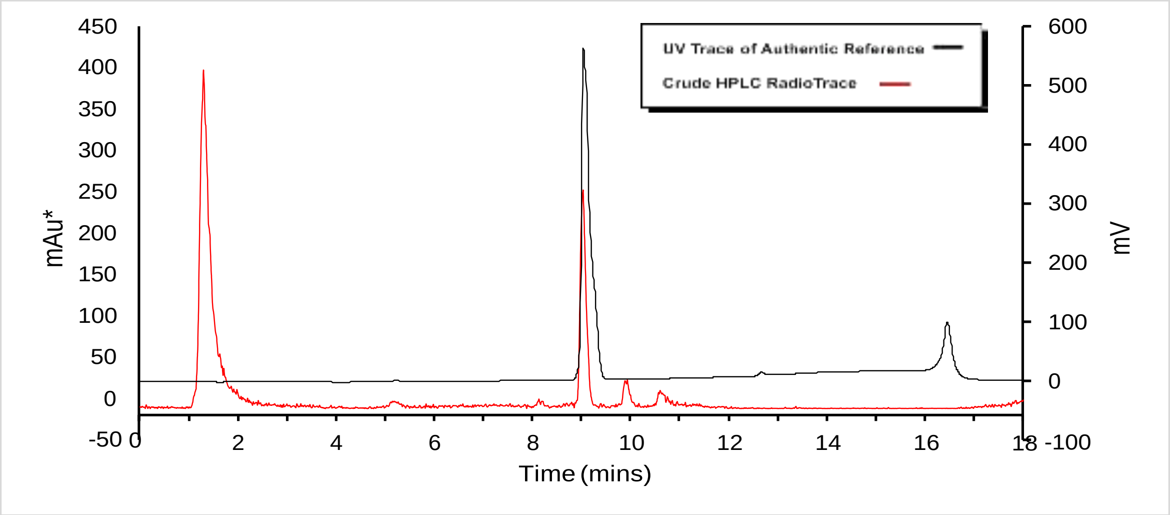


**Figure SI-6** HPLC radio-trace of **[$^{18}$F]3f** (red) overlaid with HPLC UV-trace (λ = 220 nm) of $^{19}$F reference compound (black).

[$^{18}$F]3g

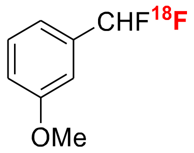


| Reaction | Radio-TLC | Radiochemical Conversion |
|---|---|---|
| 1 | 23% | 21 |
| 2 | 22% | 21 |
| 3 | 26% | 26% |
| 4 | 14% | 14% |
| Radiochemical Conversion + Standard Deviation | | **21% ± 5%** |

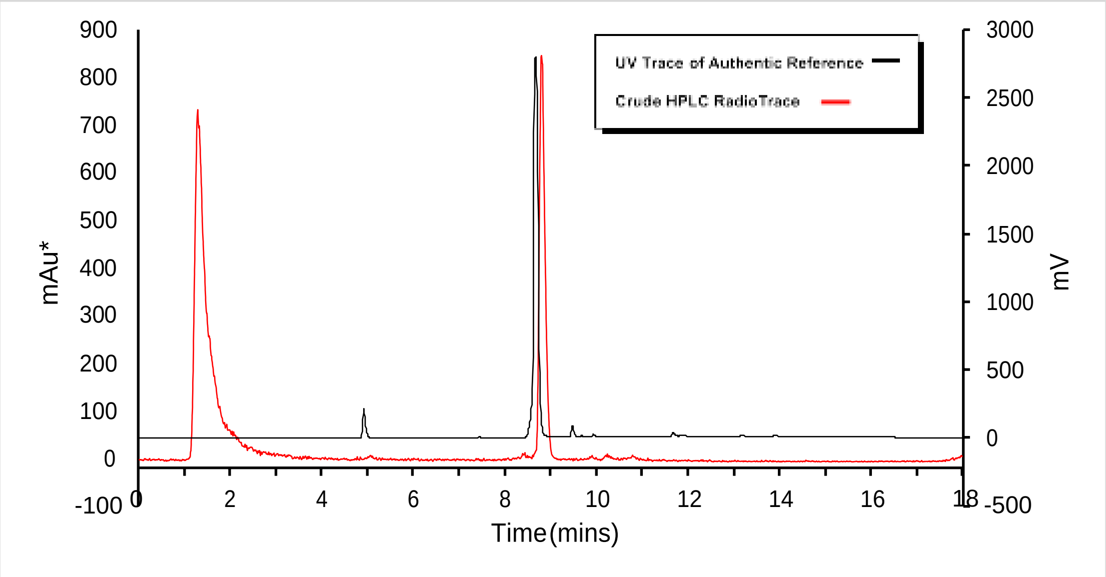


**Figure SI-7** HPLC radio-trace of **[$^{18}$F]3g** (red) overlaid with HPLC UV-trace (λ = 220 nm) of $^{19}$F reference compound (black).

[$^{18}$F]3h

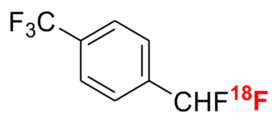


| Reaction | Radio-TLC | Radiochemical Conversion |
|---|---|---|
| 1 | 16% | 5% |
| 2 | 9% | 3% |
| Radiochemical Conversion + Standard Deviation | | **4% ± 1%** |

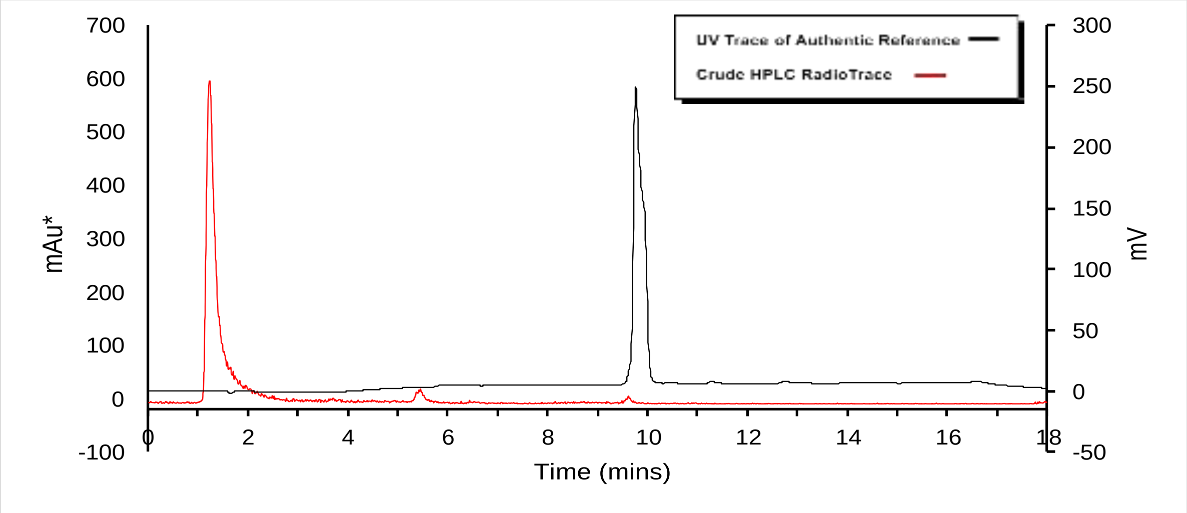


**Figure SI-8** HPLC radio-trace of **[$^{18}$F]3h** (red) overlaid with HPLC UV-trace ($\lambda$ = 220 nm) of $^{19}$F reference compound (black).

[$^{18}$F]3i

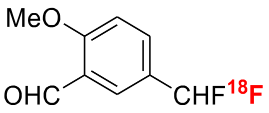


| Reaction | Radio-TLC | Radiochemical Conversion |
|---|---|---|
| 1 | 10% | 10% |
| 2 | 8% | 8% |
| 3 | 8% | 8% |
| 4 | 10% | 10% |
| Radiochemical Conversion + Standard Deviation | | **9% ± 1%** |

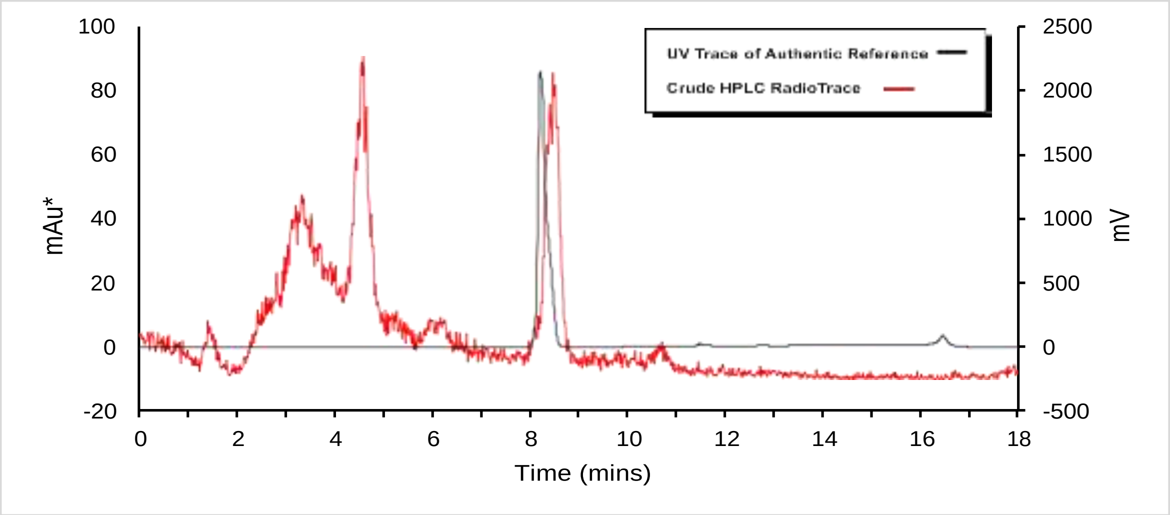


**Figure SI-9** HPLC radio-trace of **[$^{18}$F]3i** (red) overlaid with HPLC UV-trace ($\lambda$ = 220 nm) of $^{19}$F reference compound (black).

[$^{18}$F]3j

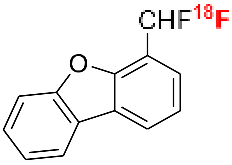


| Reaction | Radio-TLC | Radiochemical Conversion |
|---|---|---|
| 1 | 27% | 27% |
| 2 | 39% | 39% |
| 3 | 31% | 26% |
| 4 | 43% | 35% |
| Radiochemical Conversion + Standard Deviation | | **32% ± 6%** |

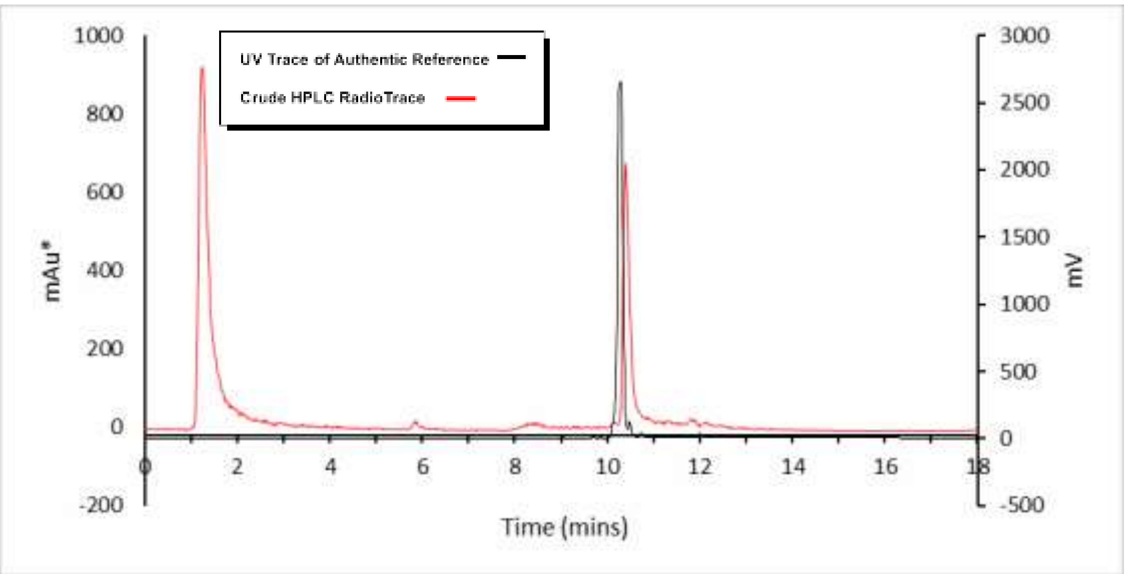


**Figure SI-10** HPLC radio-trace of **[$^{18}$F]3j** (red) overlaid with HPLC UV-trace (λ = 220 nm) of $^{19}$F reference compound (black).

**[$^{18}$F]3k**

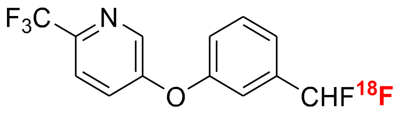


| Reaction | Radio-TLC | Radiochemical Conversion |
|---|---|---|
| 1 | 23% | 21% |
| 2 | 16% | 15% |
| 3 | 16% | 15% |
| 4 | 14% | 13% |
| Radiochemical Conversion + Standard Deviation | | **16% ± 4%** |

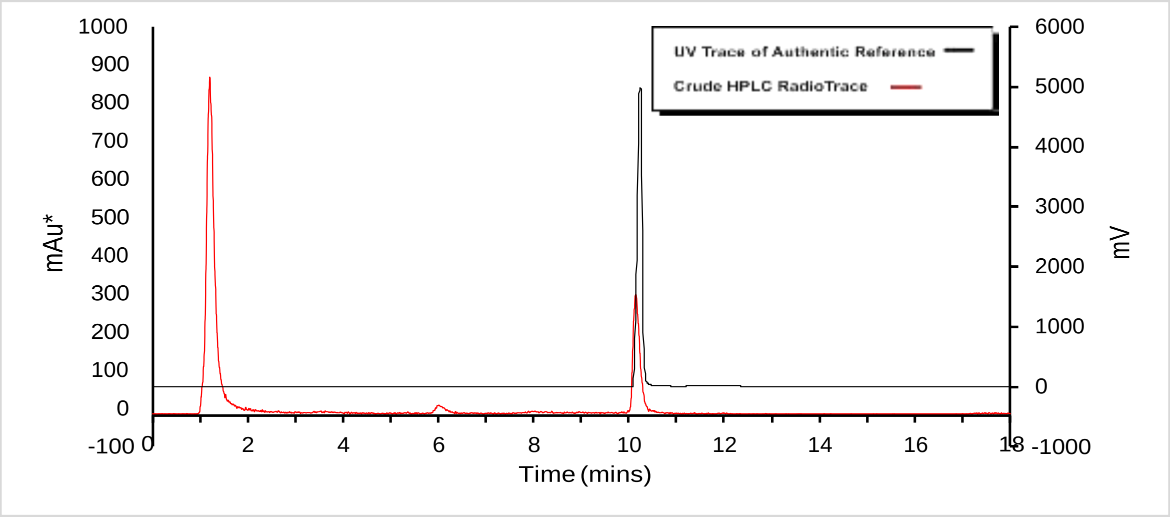


**Figure SI-11** HPLC radio-trace of **[$^{18}$F]3k** (red) overlaid with HPLC UV-trace ($\lambda$ = 220 nm) of $^{19}$F reference compound (black).

[$^{18}$F]3l

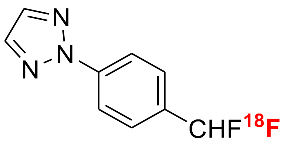


| **Reaction** | **Radio-TLC** | **Radiochemical Conversion** |
|---|---|---|
| 1 | 18% | 11% |
| 2 | 24% | 17% |
| 3 | 9% | 9% |
| 4 | 8% | 8% |
| Radiochemical Conversion + Standard Deviation | | **12% ± 4%** |

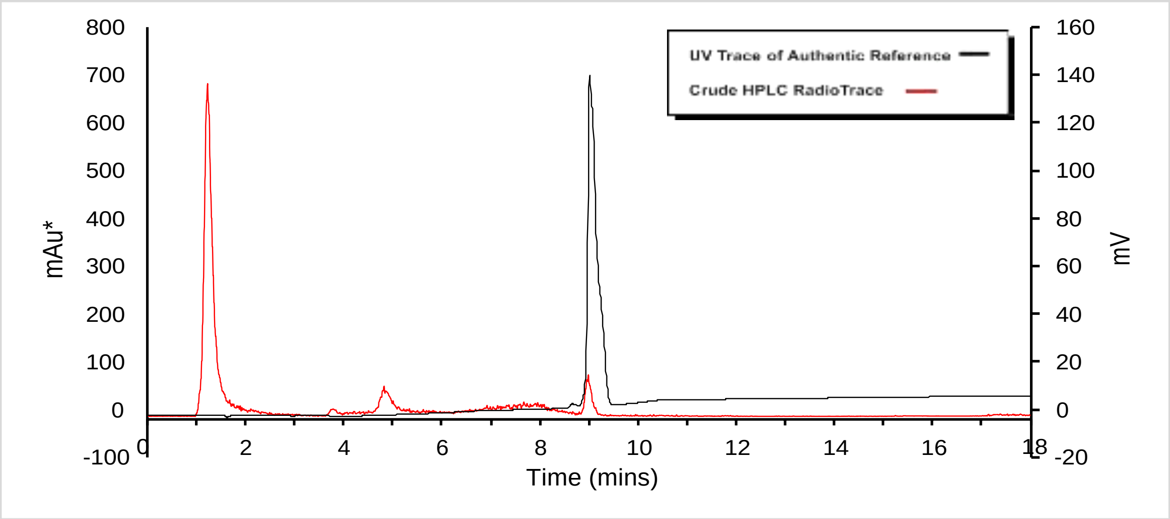


**Figure SI-12** HPLC radio-trace of **[$^{18}$F]3l** (red) overlaid with HPLC UV-trace ($\lambda$ = 220 nm) of $^{19}$F reference compound (black).

[$^{18}$F]3m

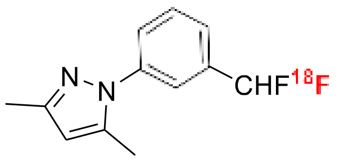


| Reaction | Radio-TLC | Radiochemical Conversion |
|---|---|---|
| 1 | 20% | 16% |
| 2 | 7% | 7% |
| 3 | 12% | 12% |
| 4 | 19% | 19% |
| Radiochemical Conversion + Standard Deviation | | **14% ± 5%** |

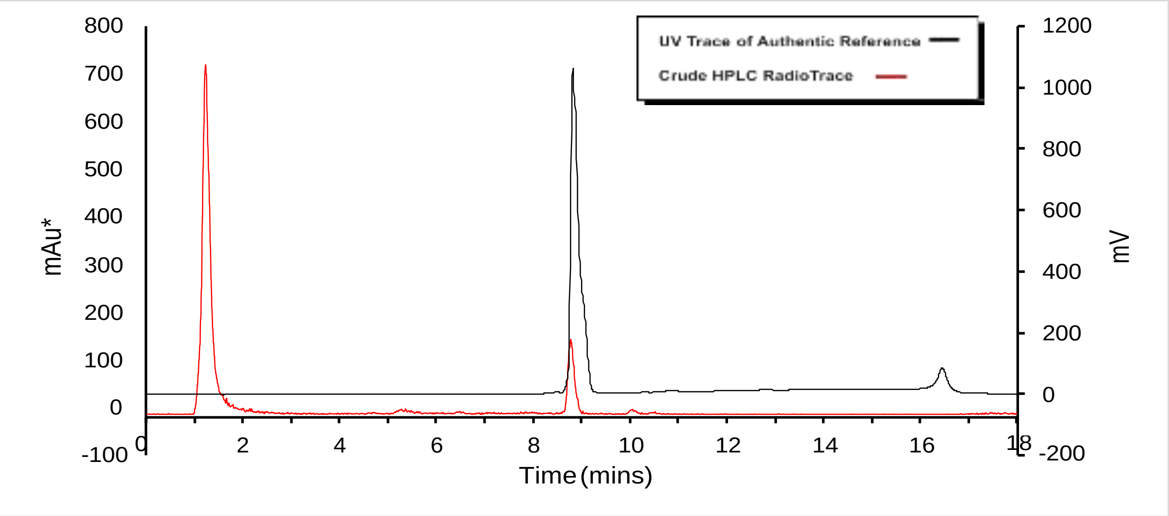


**Figure SI-13** HPLC radio-trace of **[$^{18}$F]3m** (red) overlaid with HPLC UV-trace (λ = 220 nm) of $^{19}$F reference compound (black).

[18F]3n

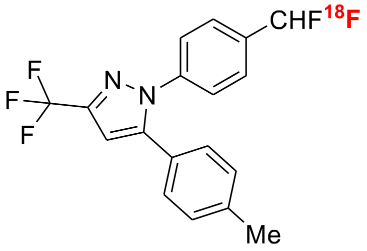


| Reaction | Radio-TLC | Radiochemical Conversion |
|---|---|---|
| 1 | 30% | 30% |
| 2 | 19% | 19% |
| 3 | 23% | 23% |
| 4 | 12% | 12% |
| Radiochemical Conversion + Standard Deviation | | **21% ± 6%** |

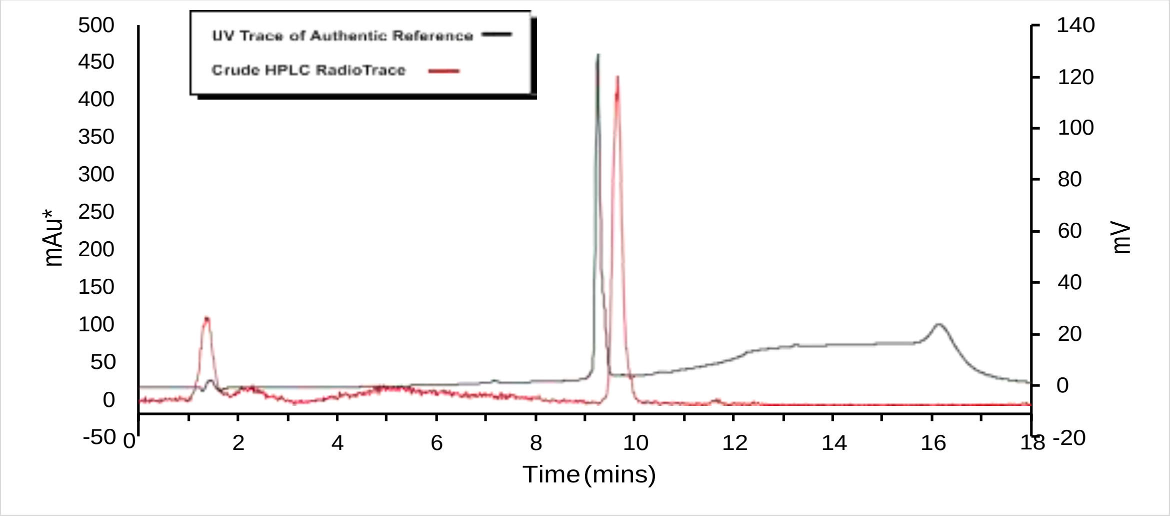


**Figure SI-14**. HPLC radio-trace of **[$^{18}$F]3n** (red) overlaid with HPLC UV-trace ($\lambda$ = 220 nm) of $^{19}$F reference compound (black).

[$^{18}$F]3o

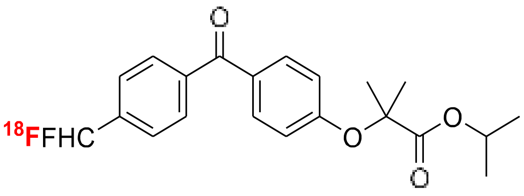


| Reaction | Radio-TLC | Radiochemical Conversion |
|---|---|---|
| 1 | 23% | 21% |
| 2 | 31% | 28% |
| 3 | 33% | 26% |
| 4 | 26% | 20% |
| Radiochemical Conversion + Standard Deviation | | **23% ± 3%** |

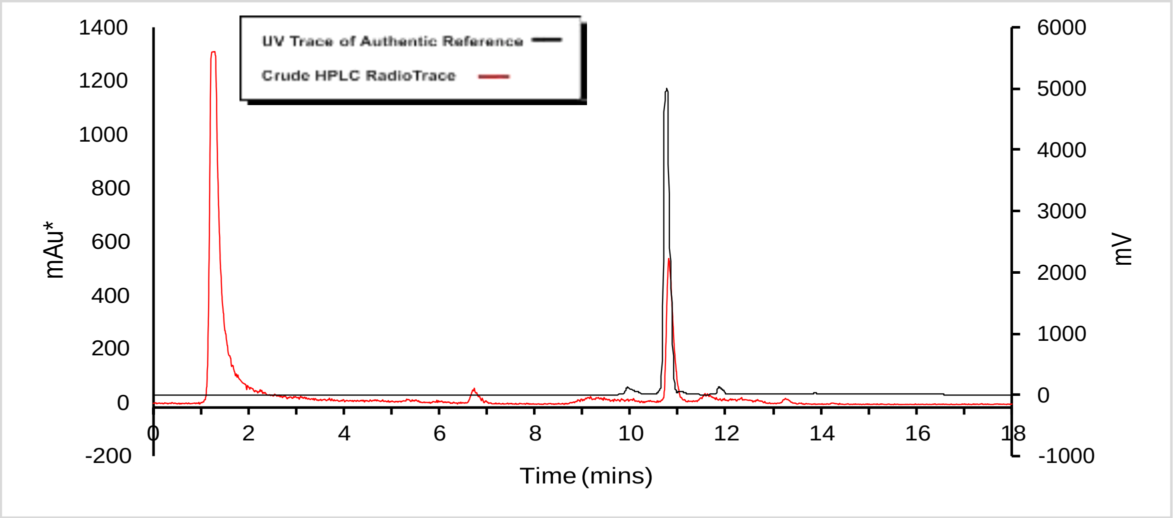


**Figure SI-15** HPLC radio-trace of **[$^{18}$F]3o** (red) overlaid with HPLC UV-trace ($\lambda$ = 220 nm) of $^{19}$F reference compound (black).

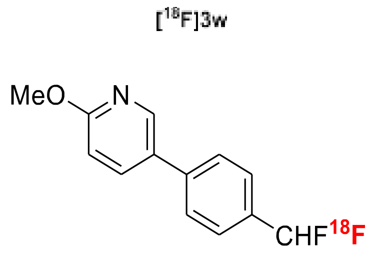


| Reaction | Radio-TLC | Radiochemical Conversion |
|---|---|---|
| 1 | 30% | 30% |
| 2 | 27% | 27% |
| 3 | 7% | 7% |
| 4 | 31% | 31% |
| Radiochemical Conversion + Standard Deviation | | **24% ± 11%** |

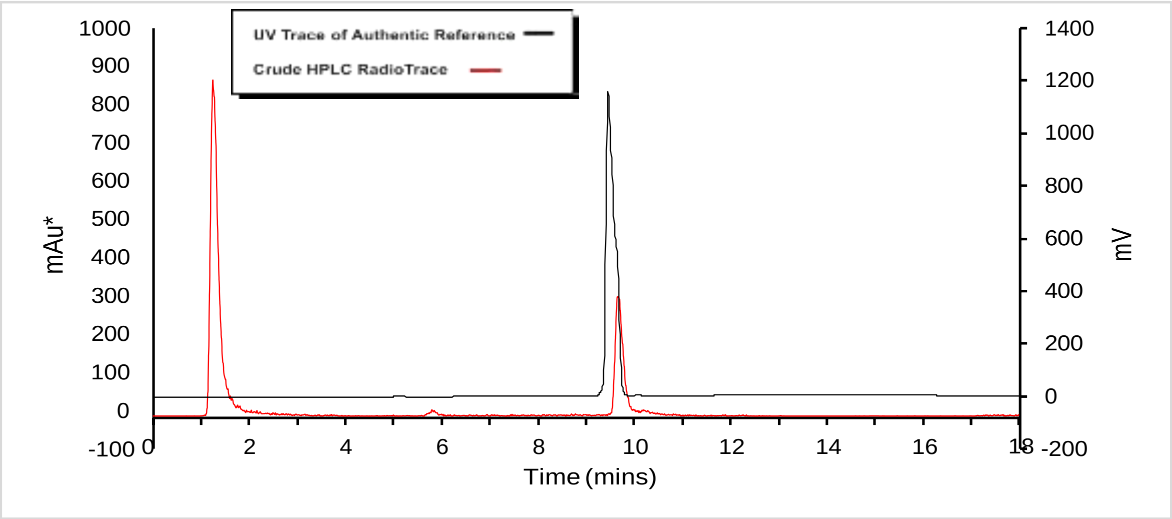


**Figure SI-16** HPLC radio-trace of **[18F]3w** (red) overlaid with HPLC UV-trace (λ = 220 nm) of 19F reference compound (black).

[$^{18}$F]3x

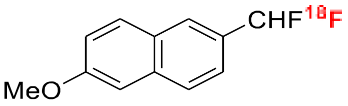


| Reaction | Radio-TLC | Radiochemical Conversion |
|---|---|---|
| 1 | 40% | 40% |
| 2 | 27% | 27% |
| 3 | 22% | 22% |
| 4 | 34% | 34% |
| Radiochemical Conversion + Standard Deviation | | **30% ± 8%** |

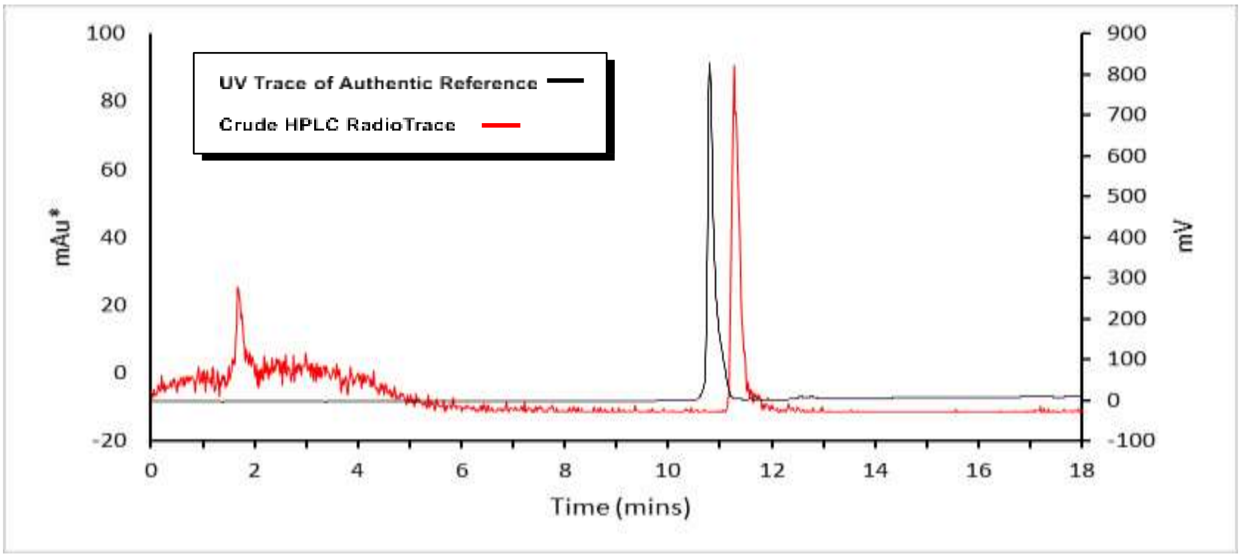


**Figure SI-17** HPLC radio-trace of **[$^{18}$F]3x** (red) overlaid with HPLC UV-trace ($\lambda$ = 220 nm) of $^{19}$F reference compound (black).

[$^{18}$F]3y

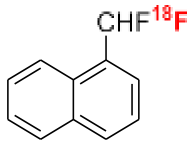


| Reaction | Radio-TLC | Radiochemical Conversion |
|---|---|---|
| 1 | 49% | 47% |
| 2 | 42% | 39% |
| 3 | 23% | 22% |
| 4 | 36% | 34% |
| Radiochemical Conversion + Standard Deviation | | **36% ± 10%** |

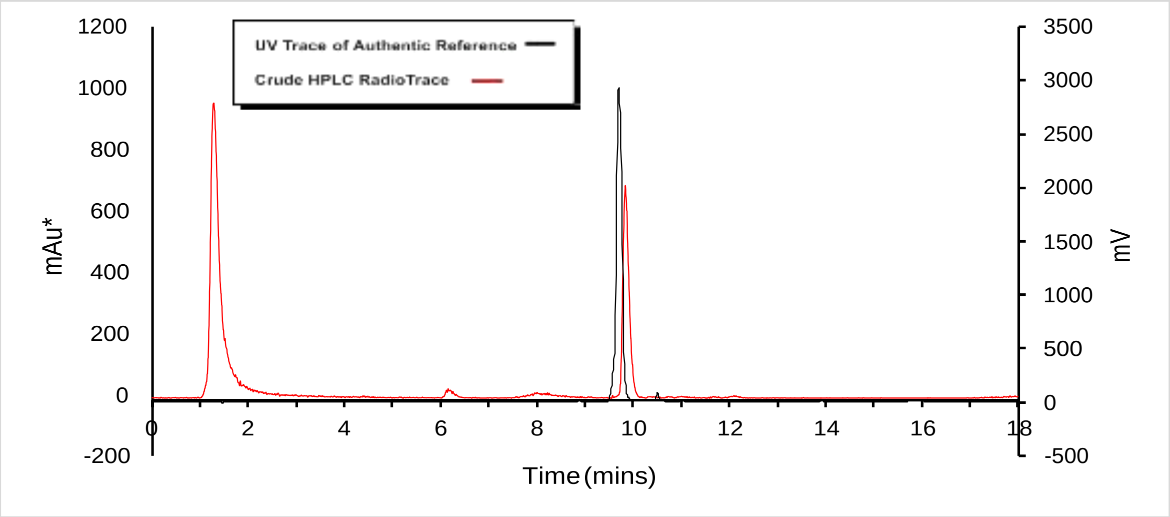


**Figure SI-18** HPLC radio-trace of **[$^{18}$F]3y** (red) overlaid with HPLC UV-trace ($\lambda$ = 220 nm) of $^{19}$F reference compound (black).

[$^{18}$F]3z

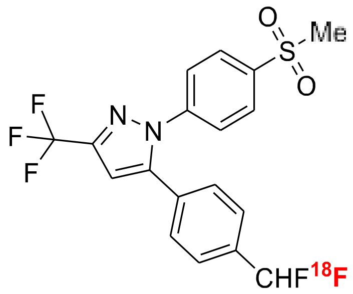


| Reaction | Radio-TLC | Radiochemical Conversion |
|---|---|---|
| 1 | 18% | 17% |
| 2 | 13% | 12% |
| 3 | 16% | 15% |
| Radiochemical Conversion + Standard Deviation | | **15% ± 2%** |

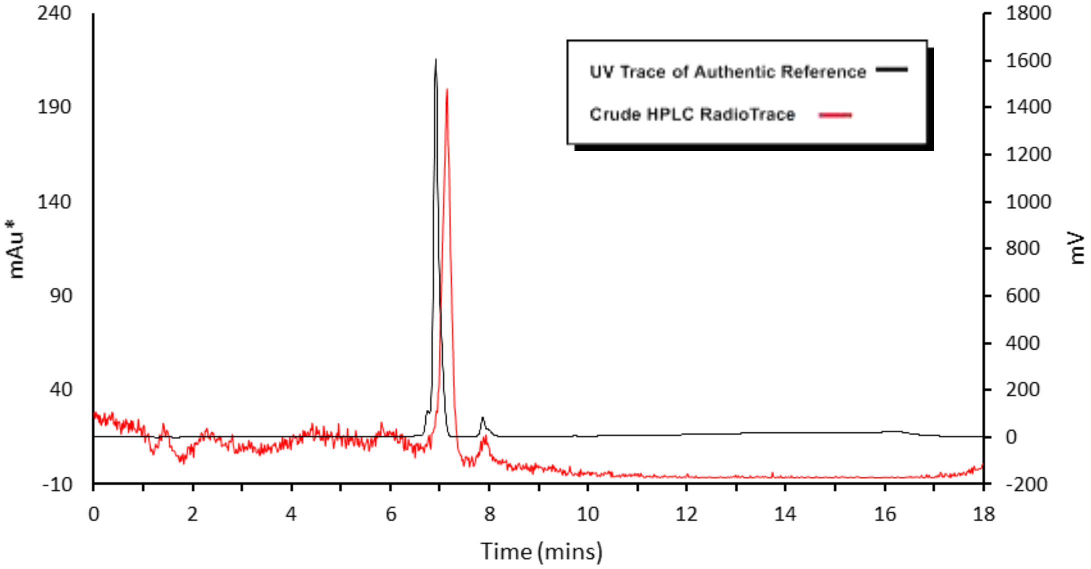


**Figure SI-19** HPLC radio-trace of **[$^{18}$F]3z** (red) overlaid with HPLC UV-trace (λ = 220 nm) of $^{19}$F reference compound (black).

[18F]11a

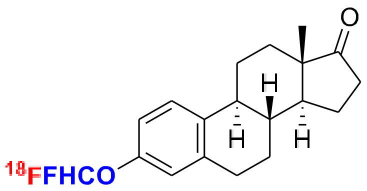


| Reaction | Radio-TLC | Radiochemical Conversion |
|---|---|---|
| 1 | 18% | 18% |
| 2 | 31% | 31% |
| 3 | 16% | 16% |
| Radiochemical Conversion + Standard Deviation | | **21% ± 6%** |

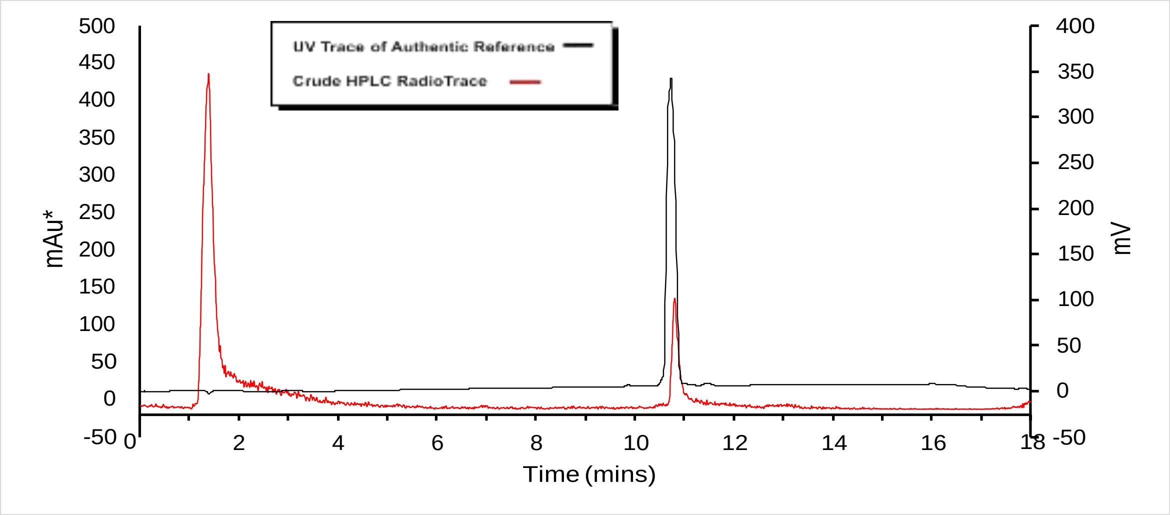


**Figure SI-20** HPLC radio-trace of **[$^{18}$F]11a** (red) overlaid with HPLC UV-trace (λ = 220 nm) of $^{19}$F reference compound (black).

**Re-optimisation for Large $^{18}F$ dose applications:**

**Elution Procedure:**

[$^{18}$F]Fluoride was separated from $^{18}$O-enriched-water using an anion exchange cartridge (Sep-Pak Accell Plus QMA Carbonate Plus Light Cartridge, 46 mg Sorbent per Cartridge, 40 µm particle size, Waters) and released with A solution of Mn(tmp)Cl (8 mg) in 600 µL of anhydrous MeOH into a 5 mL V-vial containing a magnetic stir bar in the concentrator.

**Optimisation under Mn(tmp)$^{18}$F Elution Conditions:**

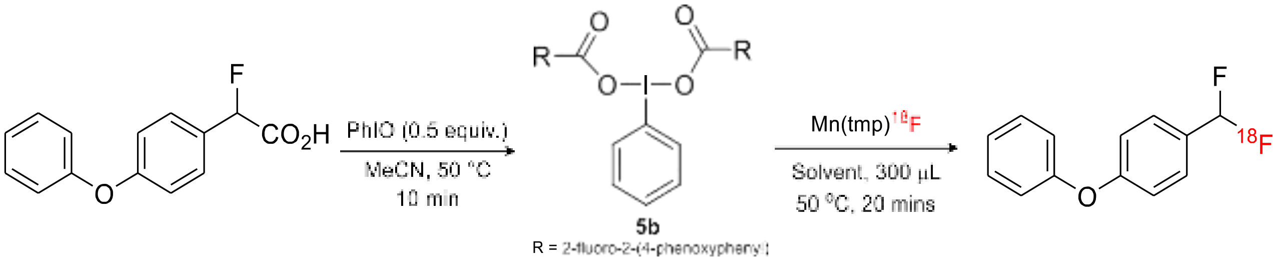


| Entry | Substrate (mmol) (5b) | Solvent | RCC (*n* = 2) |
|---|---|---|---|
| 1 | 0.014 | DMF | 0% |
| 2 | 0.014 | $CH_3CN$ | 4% ± 1% |
| 3 | 0.014 | DCM | 0% |
| 4 | 0.014 | DCE | 25% ± 6% |
| 5 | 0.056 | DCE | 3% ± 1% |
| 6[a] | 0.056 | DCE | 10% ± 1% |
| **7** | **0.007** | **DCE** | **37% ± 0%** |

[a]4 mg of Mn(tmp)Cl spiked into reaction mixture.

**Re-optimisation for clinically relevant doses of $^{18}F$:**

**Elution Procedure:**

[$^{18}F$]Fluoride was separated from $^{18}O$-enriched-water using an anion exchange cartridge (Sep-Pak Accell Plus QMA Carbonate Plus Light Cartridge, 46 mg Sorbent per Cartridge, 40 µm particle size, Waters) and released with A solution of Mn(tmp)Cl (8 mg) in 600 µL of anhydrous MeOH into a 5 mL V-vial containing a magnetic stir bar in the concentrator.

**Procedure for the $^{18}F$-fluorination of 5b under Batch Scale Isolation:**

[$^{18}F$]Fluoride was separated from $^{18}O$-enriched-water using an anion exchange cartridge (Sep-Pak Accell Plus QMA Carbonate Plus Light Cartridge, 46 mg Sorbent per Cartridge, 40 µm particle size, Waters) and released with A solution of Mn(tmp)Cl (8 mg) in 600 µL of anhydrous MeOH into a 5 mL V-vial containing a magnetic stir bar in the concentrator. The methanol was then removed at 80 °C under a flow of $N_2$. Once dry, the Mn(tmp)$^{18}F$ was dissolved in DCM (1 mL) and transferred to a 5 mL vial. Upon transfer, the DCM was removed at 80 °C under a flow of $N_2$. Once dry, substrate (0.007 mmol) dissolved in DCE (300 µL) was added and the reaction stirred at 60 °C for 20 minutes. Upon completion, the DCE was removed at 70 °C under a flow of $N_2$. The crude material was dissolved in DMF (300 µL). Upon cooling, the crude solution was diluted in $H_2O$ (6 mL) and eluted over a C18 SepPak cartridge (preconditioned with 2 mL MeOH followed by 10 mL $H_2O$). The 5 mL vial was rinsed again with 10% MeCN in $H_2O$ (2 mL) and passed over the C18 SepPak cartridge. The desired product was then eluted of the C18 SepPak cartridge with MeCN (2 mL) upon with RadioHPLC analysis was carried out to confirm the radiochemical purity of the product. Analysis was performed using a Waters Nova-Pak C18 column (4 µm, 3.9 x 150 mm) at a flow rate 1 mL/min. The overall synthesis time was 60 minutes.

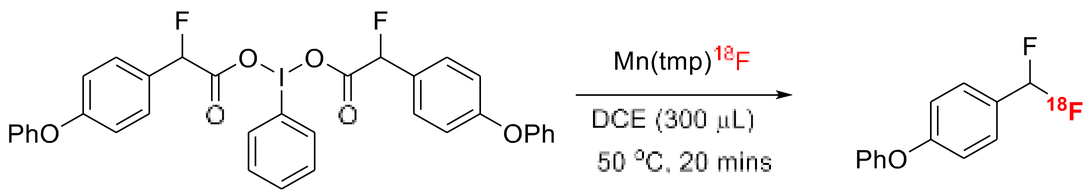


| Starting Activity (MBq) | Eluted (MBq) | Activity after drying MeOH (MBq) | Activity after transfer and DCM drying (MBq) | Activity after C18 (MBq) | RCY (decay corrected) |
|---|---|---|---|---|---|
| **841** | **720** | **704** | **594** | **67** | **12%** |

## Molar Activity Calculation:

The Molar Activity of [$^{18}$F]1-(difluoromethyl)-4-phenoxybenzene was assessed by radio-HPLC, using an analytical Analysis was performed using a Waters Nova-Pak C18 column (4 µm, 3.9 x 150 mm) at a flow rate 1 mL/min under HPLC gradient A.

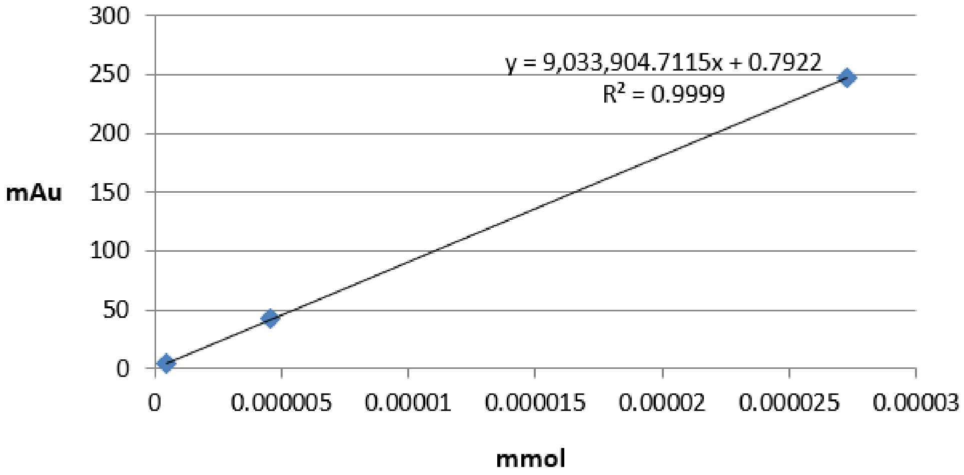


| Run | MA (GBq/µmol) | Activity (MBq) | mmol | Area (mAu) |
|---|---|---|---|---|
| **First injection** | **3.22** | 3.07 | 9.52412E-07 | 8.604 |
| **Second injection** | **2.77** | 2.76 | 9.95915 E-07 | 8.997 |

**First Injection**

y = 9,033,904.7115x + 0.7922 (y = mAu, x = mmol)

Area measured from isolated sample: 8.604 mAu

Activity of isolated sample: 3.07 MBq = 3.07E-03 GBq

mmol of isolated sample = 8.604/9,033,904= 9.52412E-07 mmol

9.52412E-07 mmol = 9.52412E-04 µmol

MA = 0.00307/9.52412E-04= 3.22 GBqµmol$^{-1}$

**Second Injection**

y = 9,033,904.7115x + 0.7922 (y = mAu, x = mmol)

Area measured from isolated sample: 8.997 mAu

Activity of isolated sample: 2.76 MBq = 2.76E-03 GBq

mmol of isolated sample = 8.997/9,033,904= 9.95915E-07 mmol

9.95915E-07 mmol = 9.95915E-04 µmol

MA = 0.00276/9.95915E-04= 2.77 GBqµmol$^{-1}$

**Average**

**M.A = 3.0 GBq/µmol**

**Attempt at one-pot procedure with the aryl boron reagent, ethyl 1-fluoro-2-bromoacetate and $^{18}$F-fluoride**

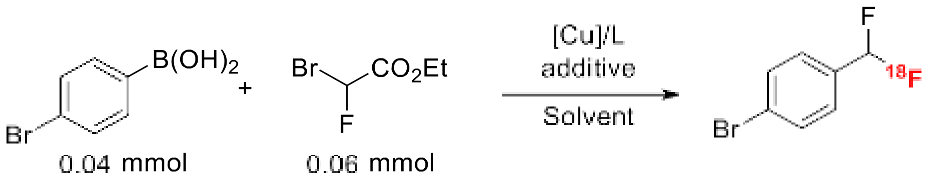


| Entry | Copper source (0.06 mmol) | Additive (mmol) | Temperature (°C) | RCC (*n* = 2) |
|---|---|---|---|---|
| 1 | CuI | TMEDA (0.1) | r.t | 0% |
| 2 | CuI | TMEDA (0.1) | r.t | 0% |
| 3 | Cu(II)OTf$_2$(py)$_4$ | n/a | r.t | 0% |
| 4 | Cu(II)OTf$_2$(py)$_4$ | n/a | r.t | 0% |
| 5 | [(iPr)CuCl] | n/a | r.t | 0% |
| 6[a] | [(iPr)CuCl] | n/a | r.t | 0% |
| 7 | CuI | TMEDA (0.1) | 90 | 0% |
| 8 | CuI | TMEDA (0.1) | 90 | 0% |

## References


1) W. Liu and J. T. Groves, *Angewandte Chemie*, 2013, **125**, 6140.
2) S. Mizuta, I. S. Stenhagen, M. O'Duill, J. Wolstenhulme, A. K. Kirjavainen, S. J. Forsback, M. Tredwell, G. Sandford, P. R. Moore and M. Huiban, *Org. Lett.,* 2013, **15**, 2648.
3) S. Verhoog, L. Pfeifer, T. Khotavivattana, S. Calderwood, T. L. Collier, K. Wheelhouse, M. Tredwell and V. Gouverneur, *Synlett,* 2016, **27**, 25.
4) F. Effenberger, S. Oßwald, *Tetrahedron: Asymmetry*, 2001, **12**, 279
5) F. Zhang, J. Z. Song, *Tetrahedron Lett.*, 2006, **47**, 7641.
6) Z. Feng, Q. Min, X. Zhang, *Org. Lett.*, 2015, **18**, 44.
7) K. Aikawa, H. Serizawa, K. Ishii, K. Mikami, *Org. Lett.*, 2016, **18**, 3690.
8) H. Shi, A. Braun, L. Wang, S. H. Liang, N. Vasdev and T. Ritter, *Angew. Chem., Int. Ed.,* 2016, **55**, 10786.
9) X. Deng, J. Lin, J. Xiao, *Org. Lett.,* 2016, **18**, 4384.
10) P. S. Fier and J. F. Hartwig, *Angew. Chem. Int. Ed.*, 2013, **52**, 2092
11) Y. Xiao, Q. Min, C. Xu, R. Wang and X. Zhang, *Angew. Chem. Int. Ed.,* 2016, **55**, 5837-5841.
12) Y. Chen, P. R. Murray, A. T. Davies and M. C. Willis, *J. Am. Chem. Soc.*, 2018, **140**, 8781.

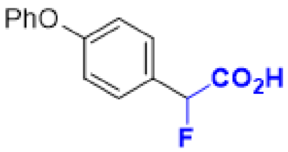

1

¹H NMR (400 MHz)
$CDCl_3$

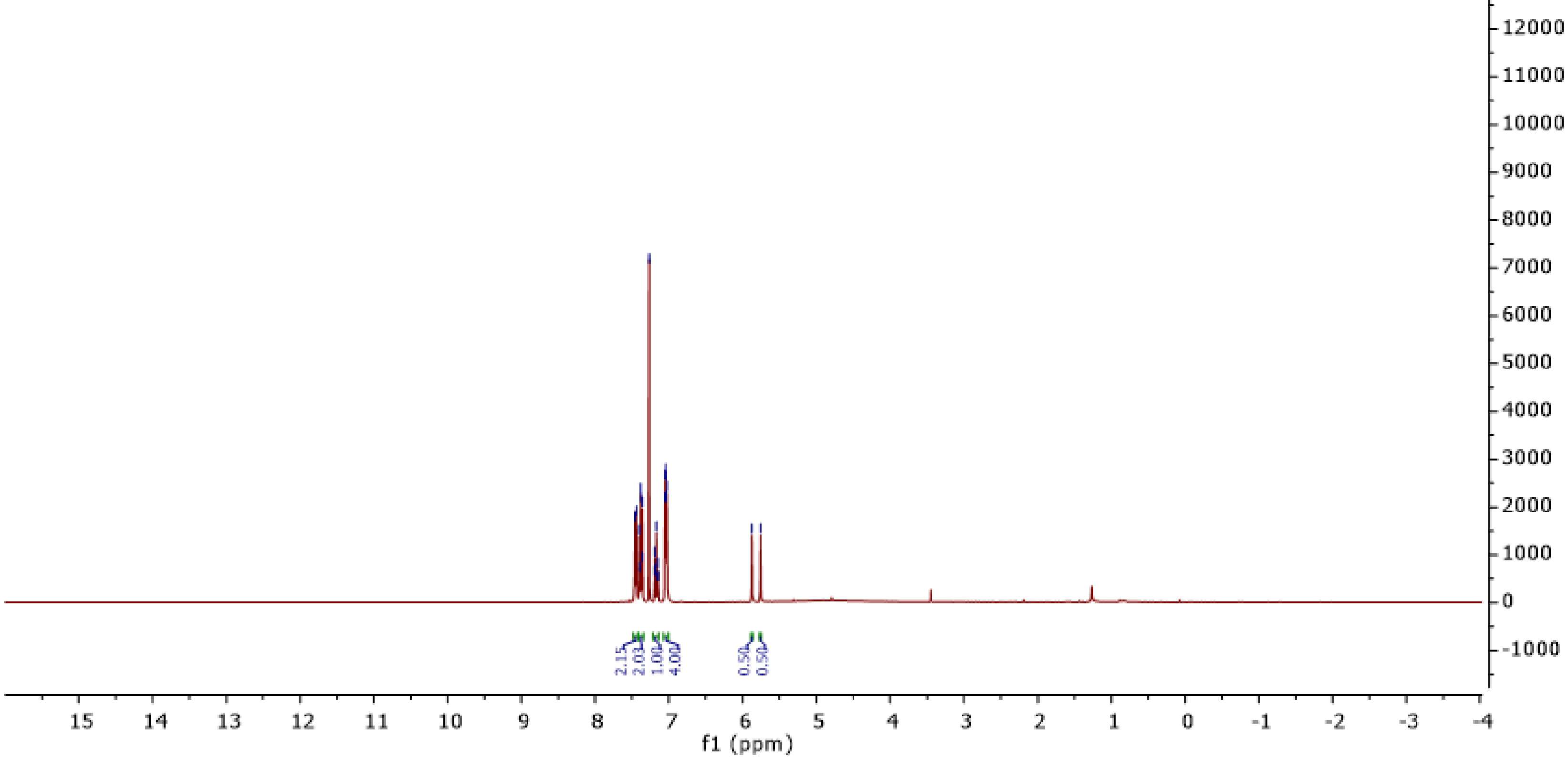

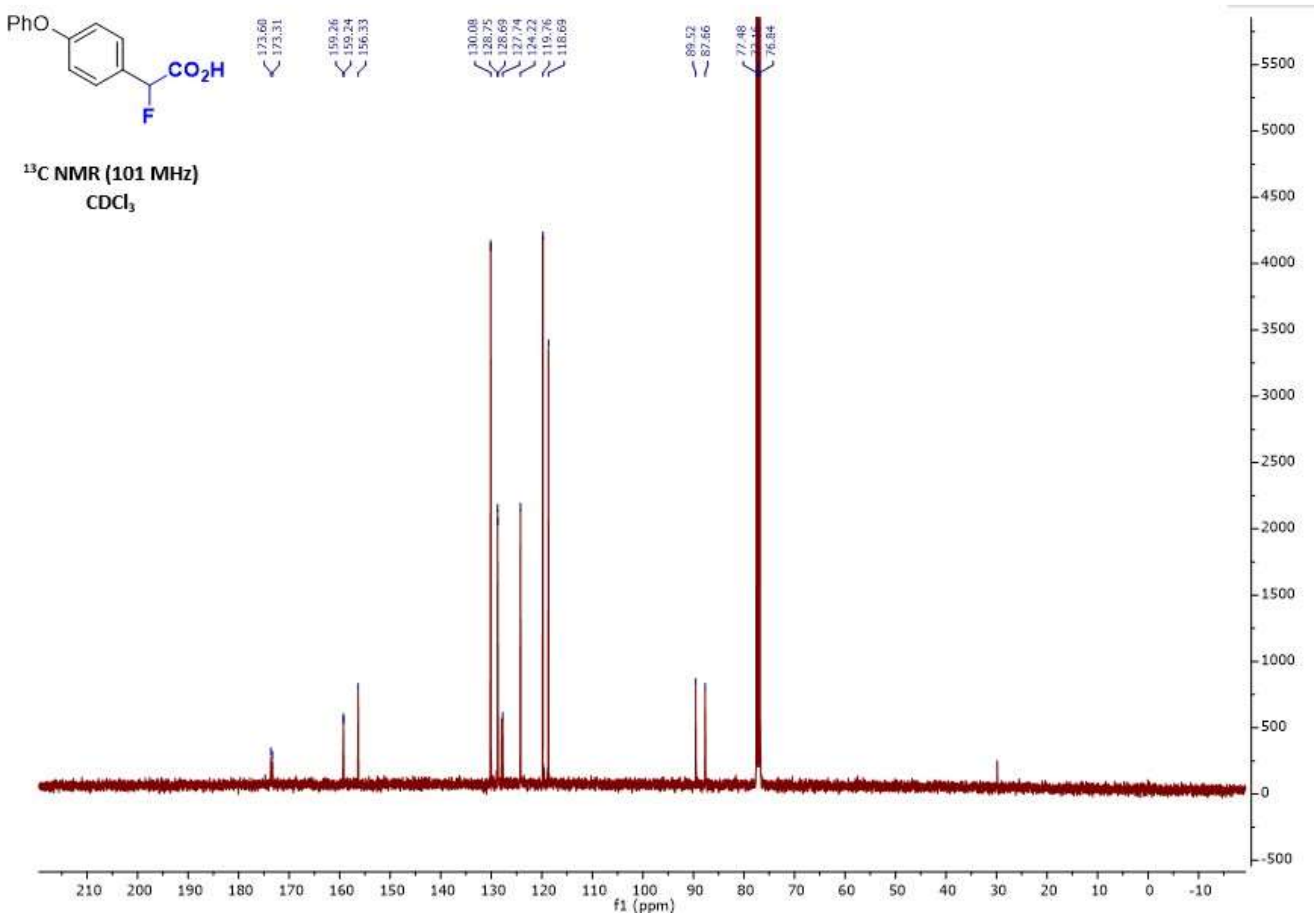
PhO
CO2H
F
13C NMR (101 MHz)
CDCl3
173.60
173.31
159.26
159.24
156.33
130.08
128.75
128.69
127.74
124.22
119.76
118.69
89.52
87.66
77.48
76.84
5500
5000
4500
4000
3500
3000
2500
2000
1500
1000
500
0
-500
210
200
190
180
170
160
150
140
130
120
110
100
90
80
70
60
50
40
30
20
10
0
-10
f1 (ppm)

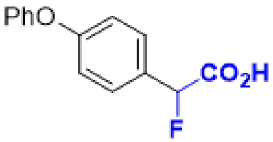


$^{13}C$ NMR (101 MHz)
$CDCl_3$

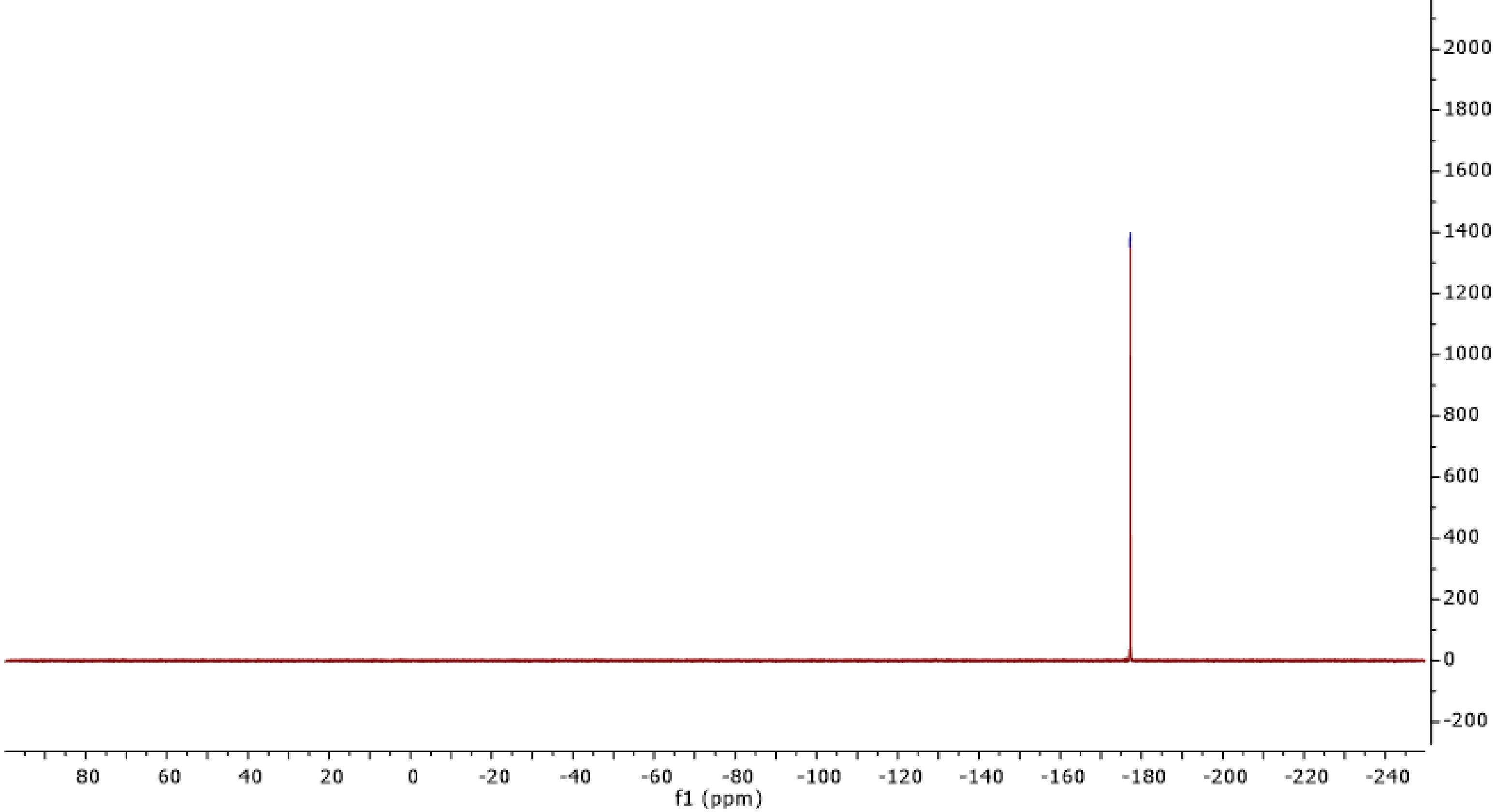

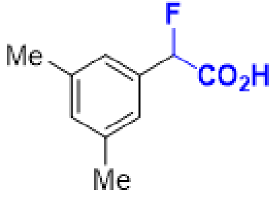

CDCl$_3$

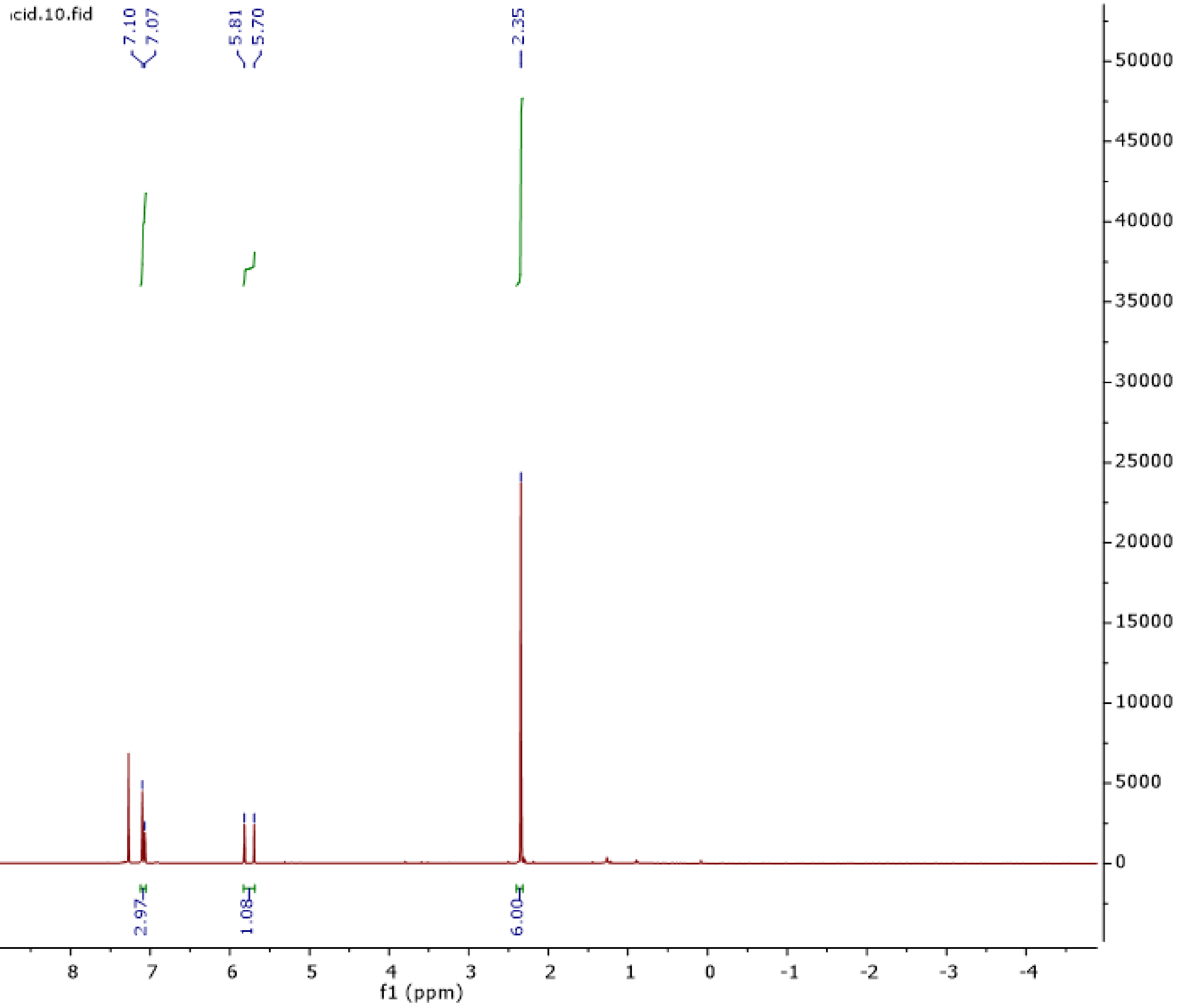

cid.10.fid
7.10
7.07
5.81
5.70
2.35
2.97
1.08
6.00
50000
45000
40000
35000
30000
25000
20000
15000
10000
5000
0
12
11
10
9
8
7
6
5
4
3
2
1
0
-1
-2
-3
-4
f1 (ppm)

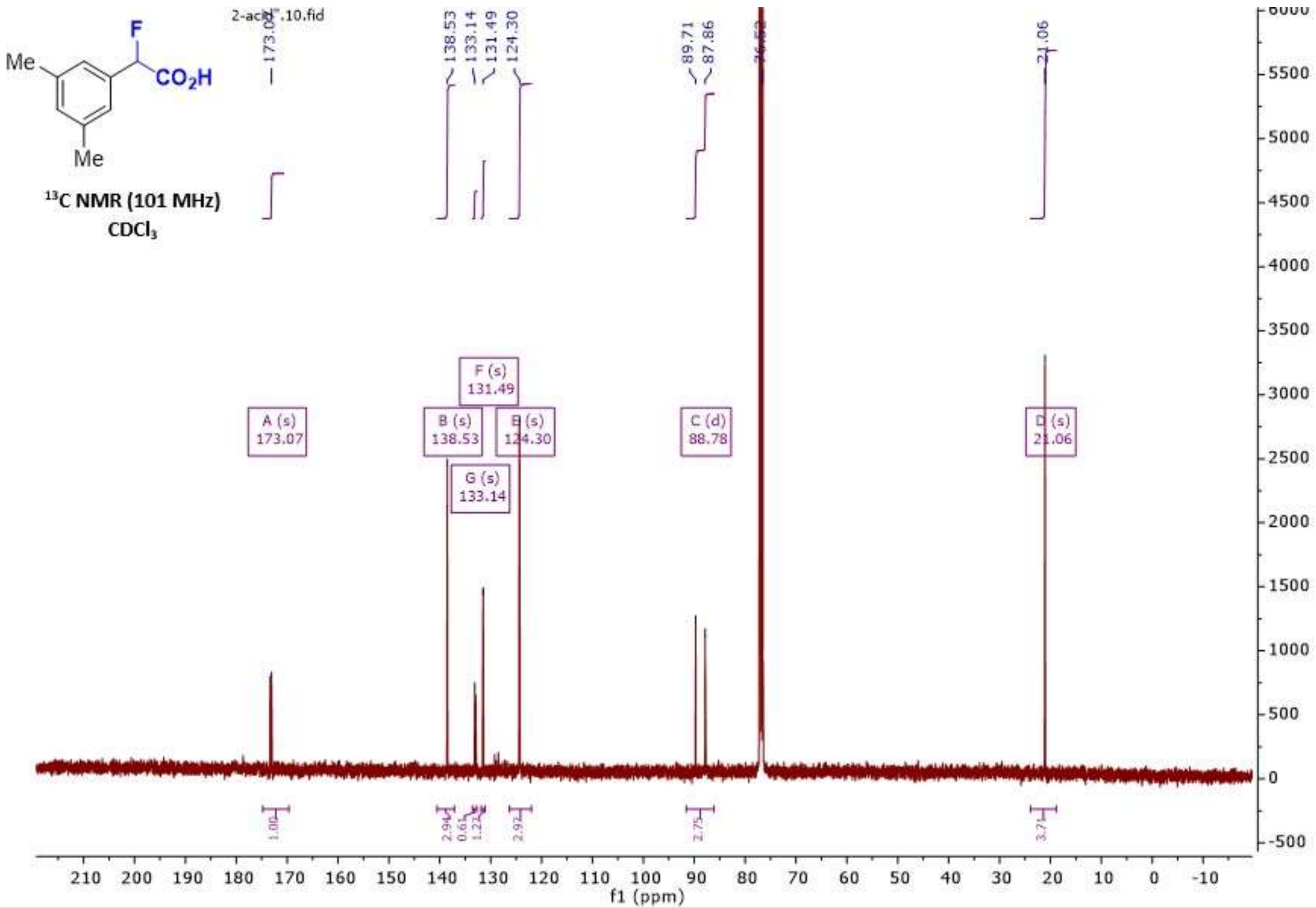

Me
F
CO2H
Me
13C NMR (101 MHz)
CDCl3
2-acid.10.fid
173.07
138.53
133.14
131.49
124.30
89.71
87.86
21.06
A (s) 173.07
B (s) 138.53
F (s) 131.49
G (s) 133.14
E (s) 124.30
C (d) 88.78
D (s) 21.06
f1 (ppm)

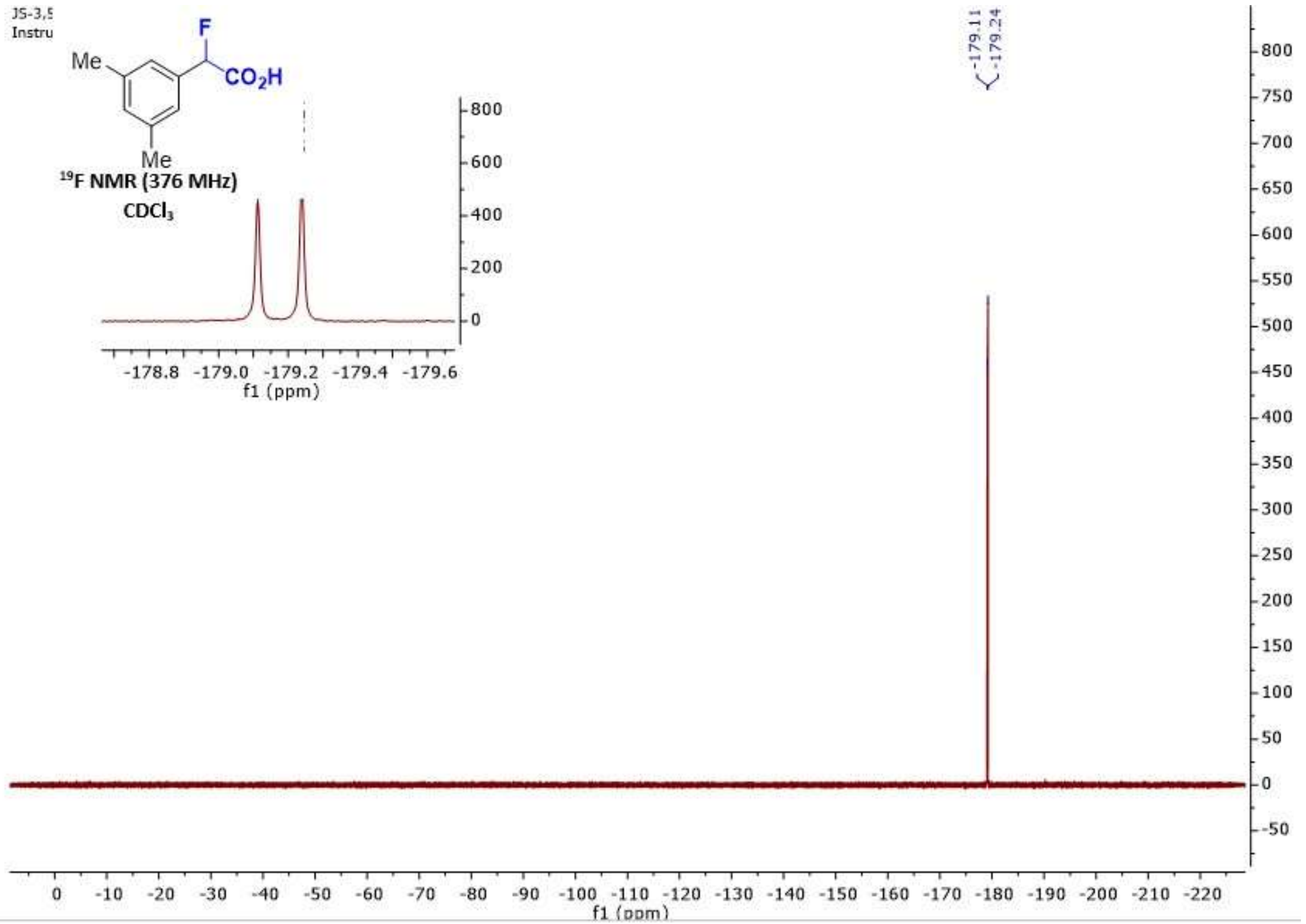
JS-3,5
Instru
F
Me
CO2H
Me
19F NMR (376 MHz)
CDCl3
-179.11
-179.24
800
600
400
200
0
-178.8 -179.0 -179.2 -179.4 -179.6
f1 (ppm)
800
750
700
650
600
550
500
450
400
350
300
250
200
150
100
50
0
-50
0 -10 -20 -30 -40 -50 -60 -70 -80 -90 -100 -110 -120 -130 -140 -150 -160 -170 -180 -190 -200 -210 -220
f1 (ppm)

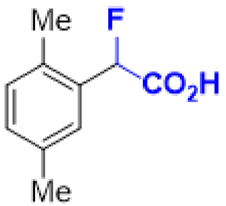


$^1$H NMR (400 MHz)
$CDCl_3$

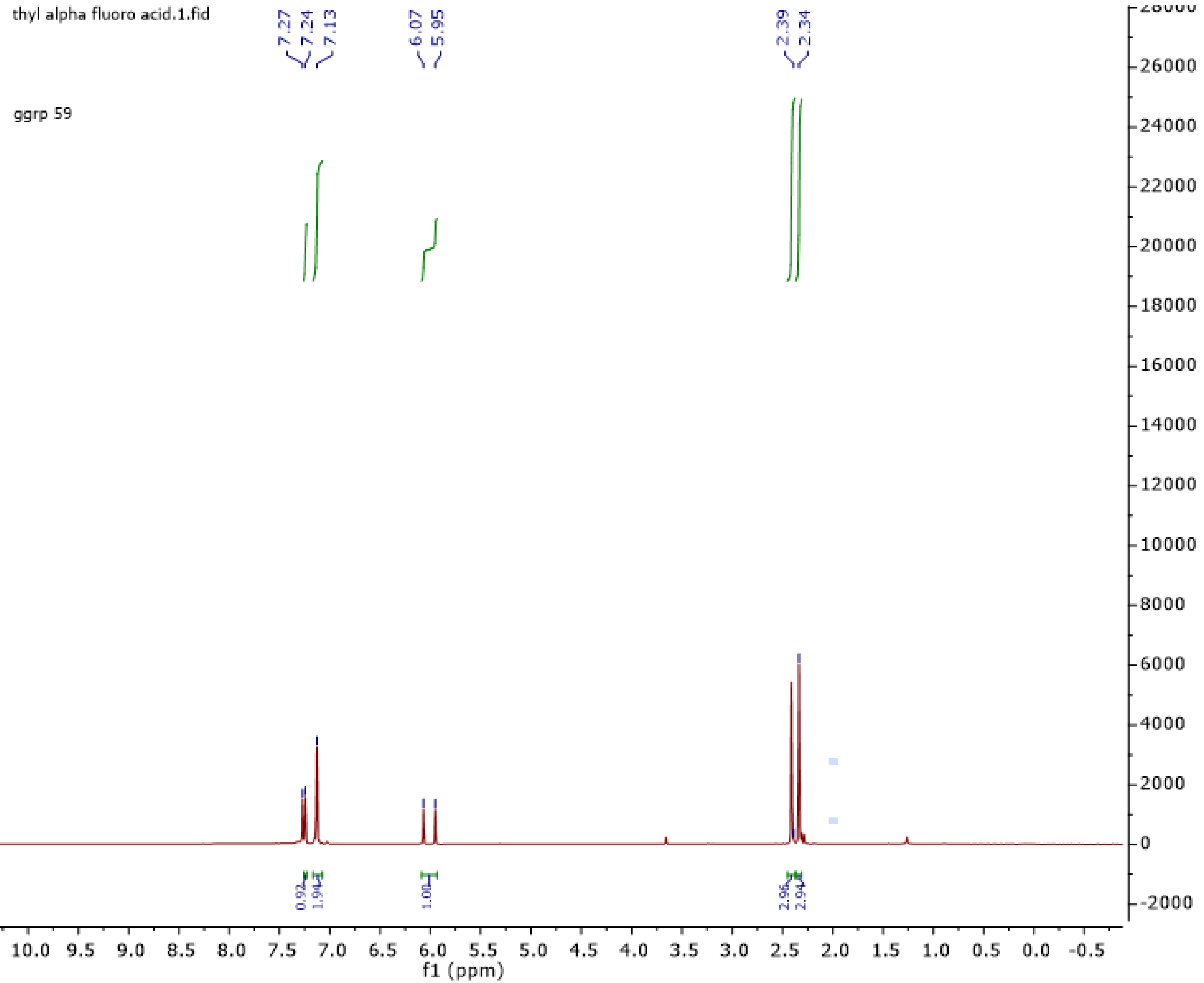


#

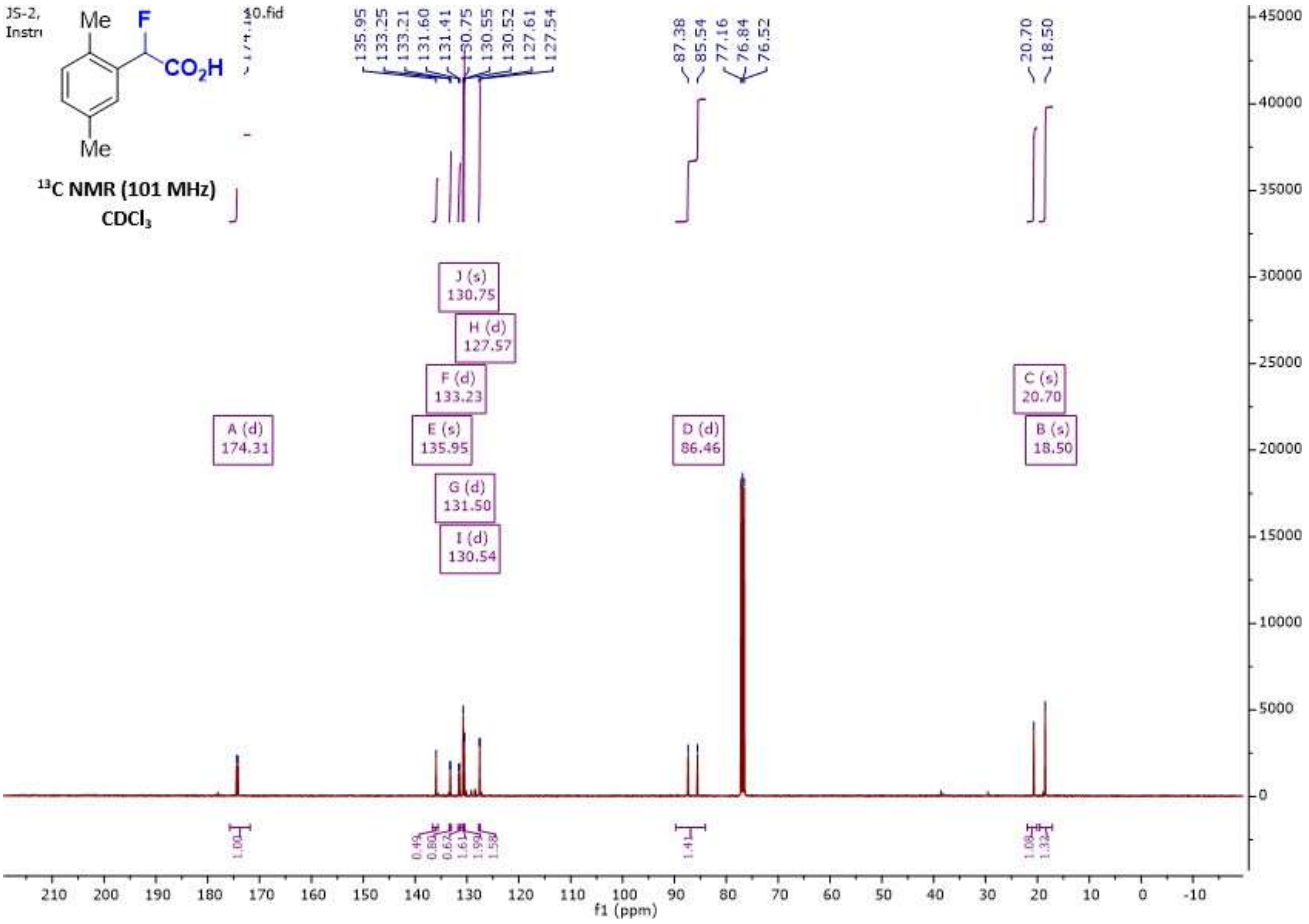

JS-2,
Instr
Me
F
$CO_2H$
Me
$^{13}$C NMR (101 MHz)
$CDCl_3$
135.95
133.25
133.21
131.60
131.41
130.75
130.55
130.52
127.61
127.54
87.38
85.54
77.16
76.84
76.52
20.70
18.50
A (d) 174.31
E (s) 135.95
F (d) 133.23
G (d) 131.50
I (d) 130.54
J (s) 130.75
H (d) 127.57
D (d) 86.46
C (s) 20.70
B (s) 18.50
1.00
0.49
0.80
0.62
1.61
1.99
1.58
1.41
1.08
1.32
f1 (ppm)

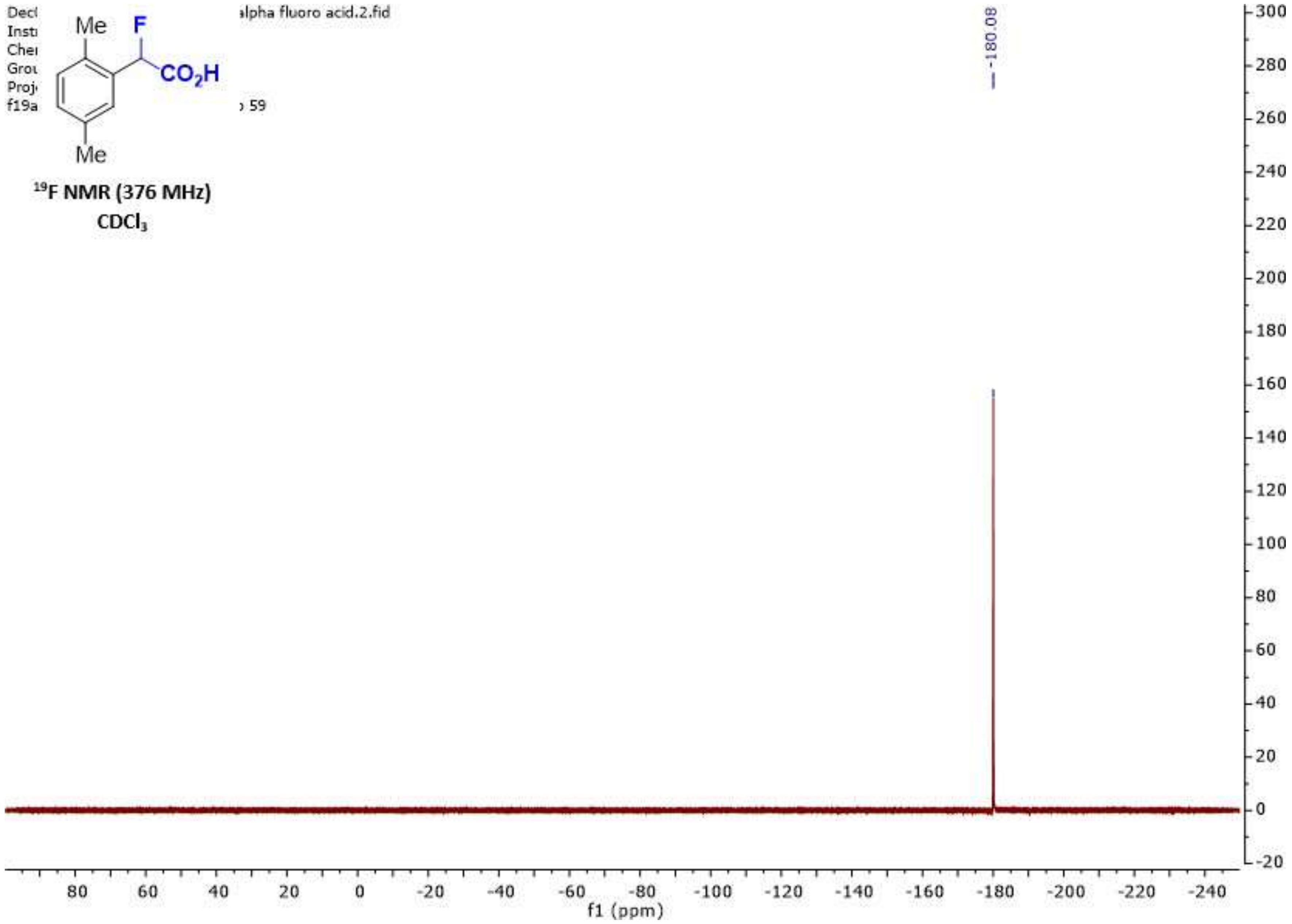

alpha fluoro acid.2.fid
Me
F
CO2H
Me
19F NMR (376 MHz)
CDCl3
-180.08
f1 (ppm)

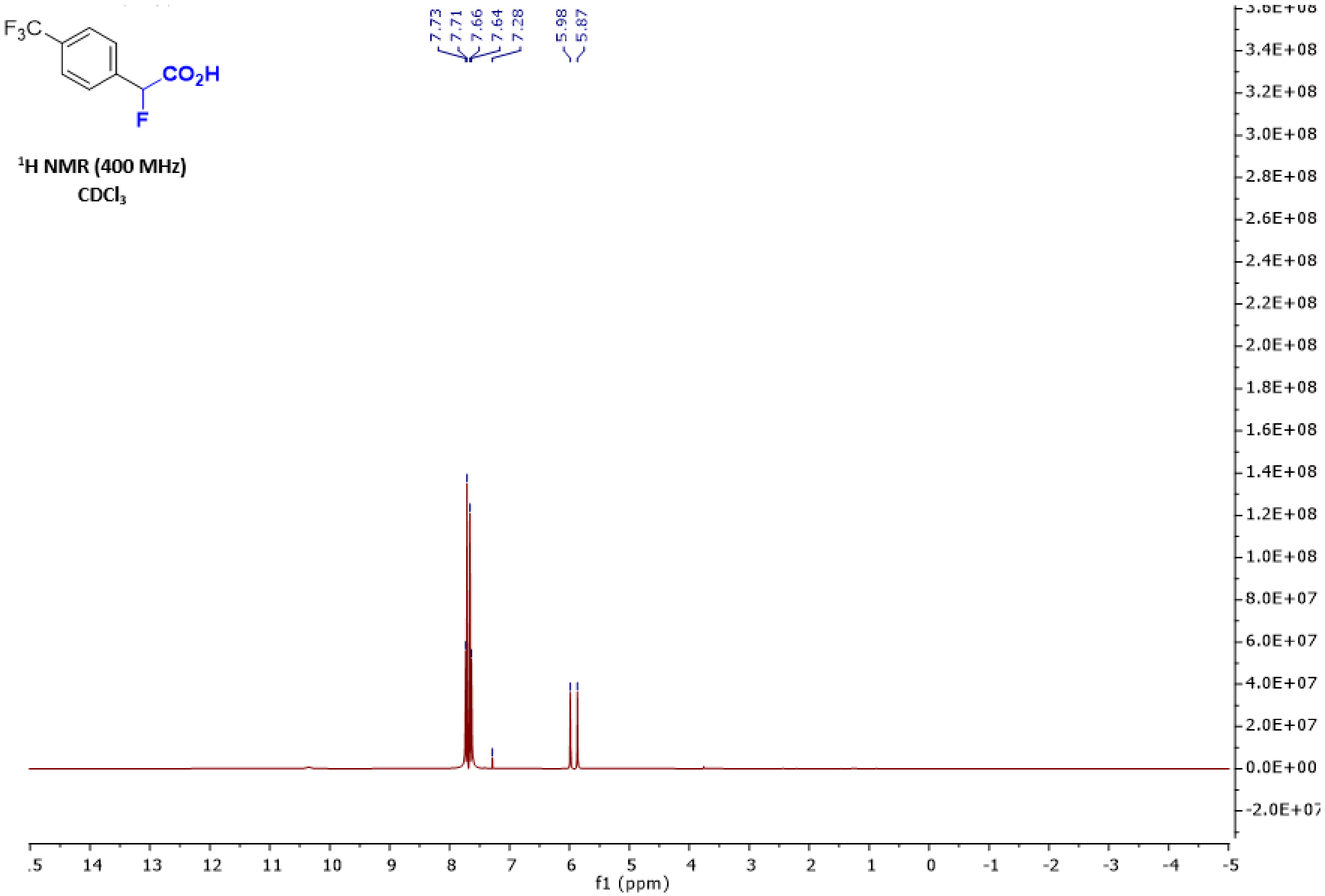

F3C
CO2H
F
1H NMR (400 MHz)
CDCl3
7.73
7.71
7.66
7.64
7.28
5.98
5.87
3.6E+08
3.4E+08
3.2E+08
3.0E+08
2.8E+08
2.6E+08
2.4E+08
2.2E+08
2.0E+08
1.8E+08
1.6E+08
1.4E+08
1.2E+08
1.0E+08
8.0E+07
6.0E+07
4.0E+07
2.0E+07
0.0E+00
-2.0E+07
.5 14 13 12 11 10 9 8 7 6 5 4 3 2 1 0 -1 -2 -3 -4 -5
f1 (ppm)

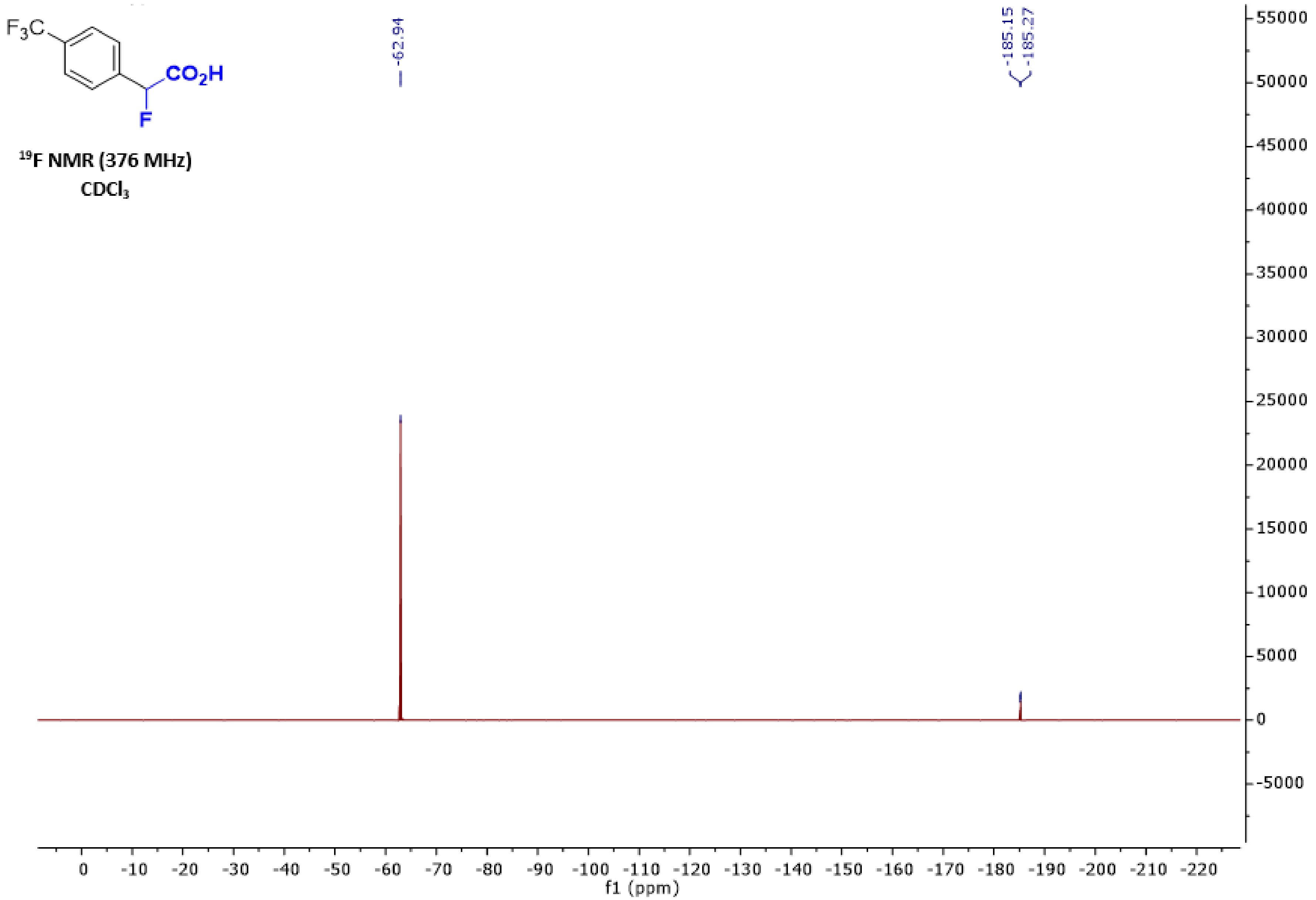


$^{19}F$ NMR (376 MHz)
$CDCl_3$

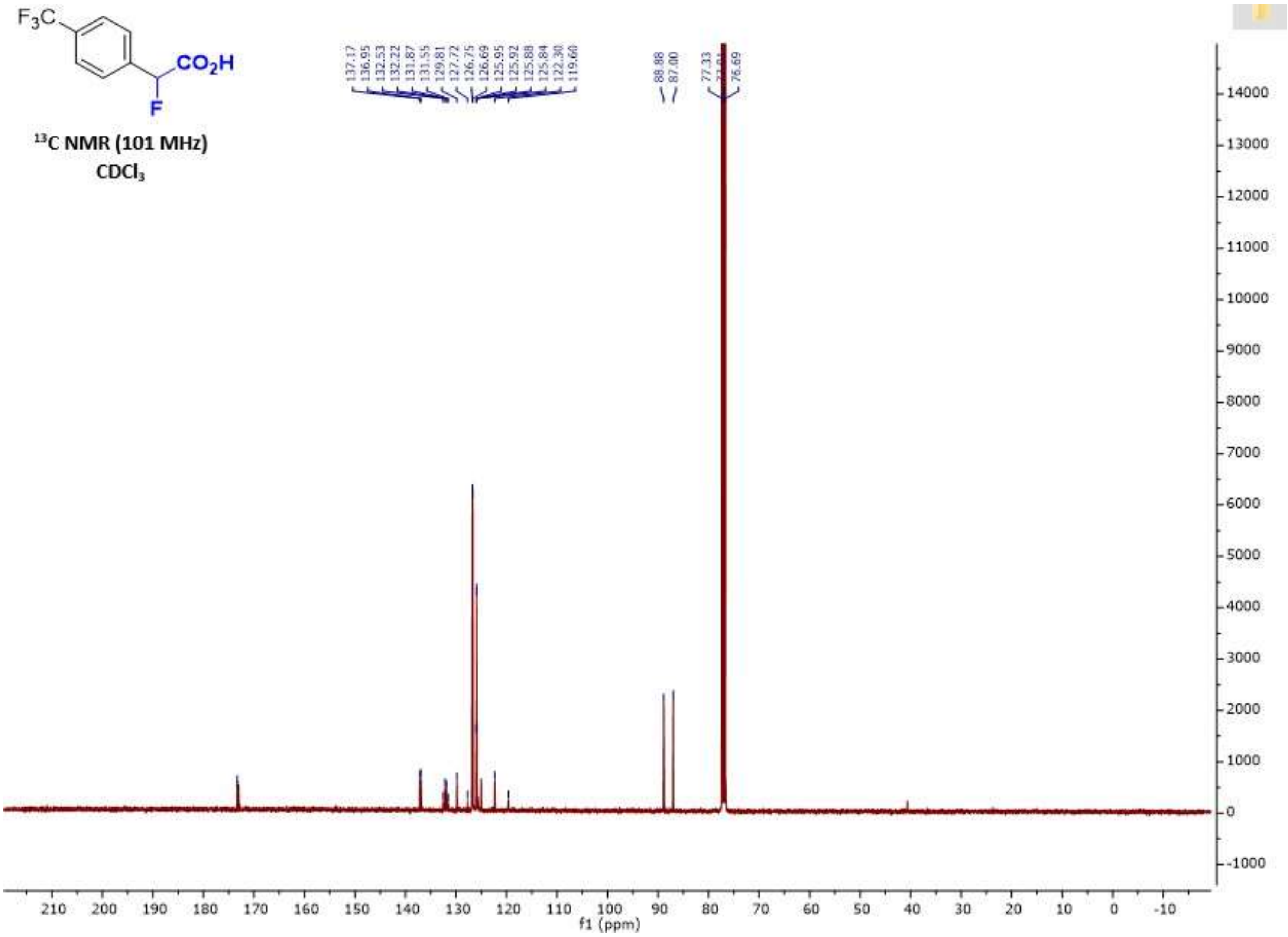
F3C
CO2H
F
13C NMR (101 MHz)
CDCl3
137.17
136.95
132.53
132.22
131.87
131.55
129.81
127.72
126.75
126.69
125.95
125.92
125.88
125.84
122.30
119.60
88.88
87.00
77.33
76.69
f1 (ppm)

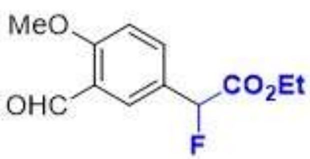


$^1$H NMR (400 MHz)
$CDCl_3$

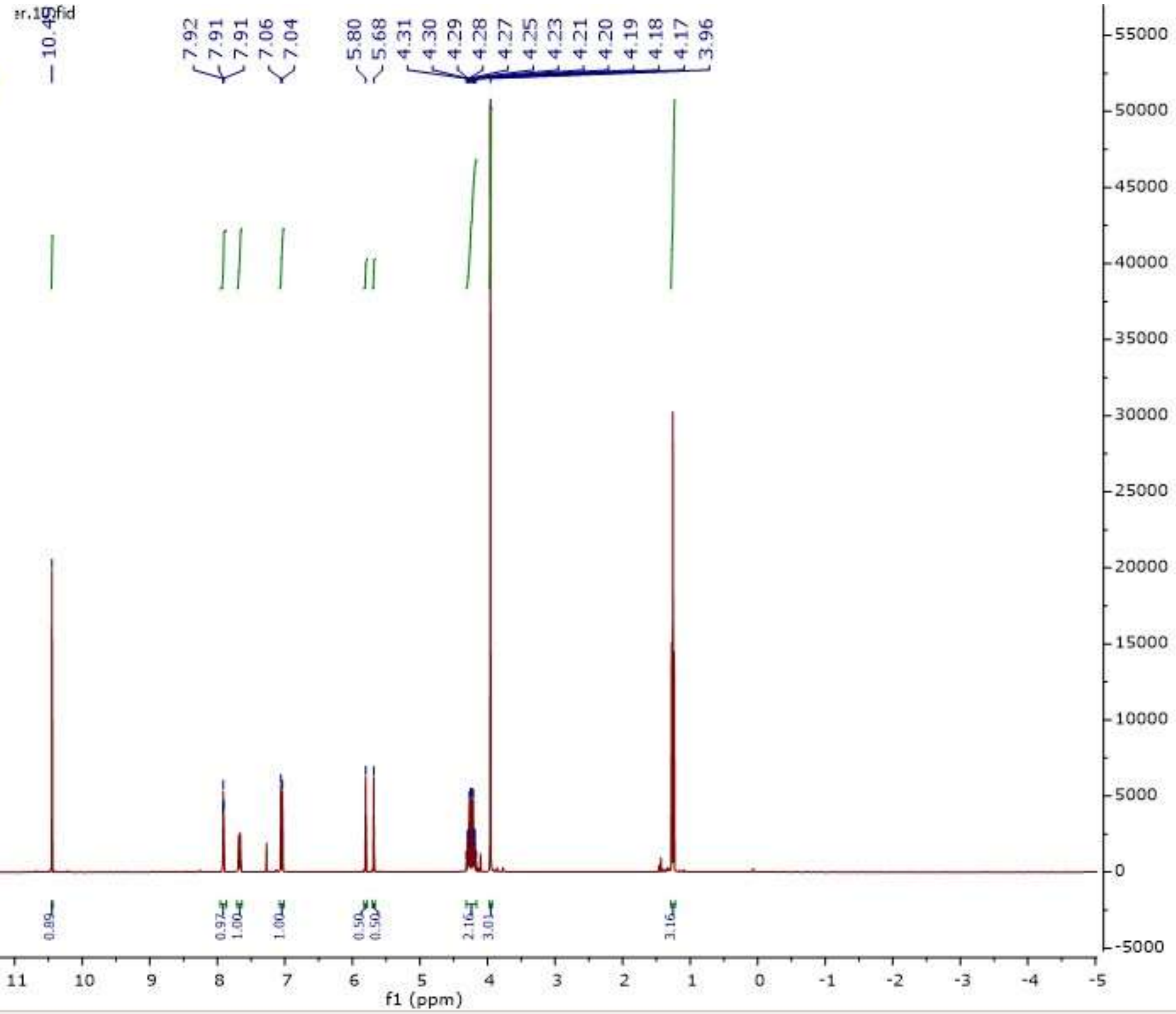

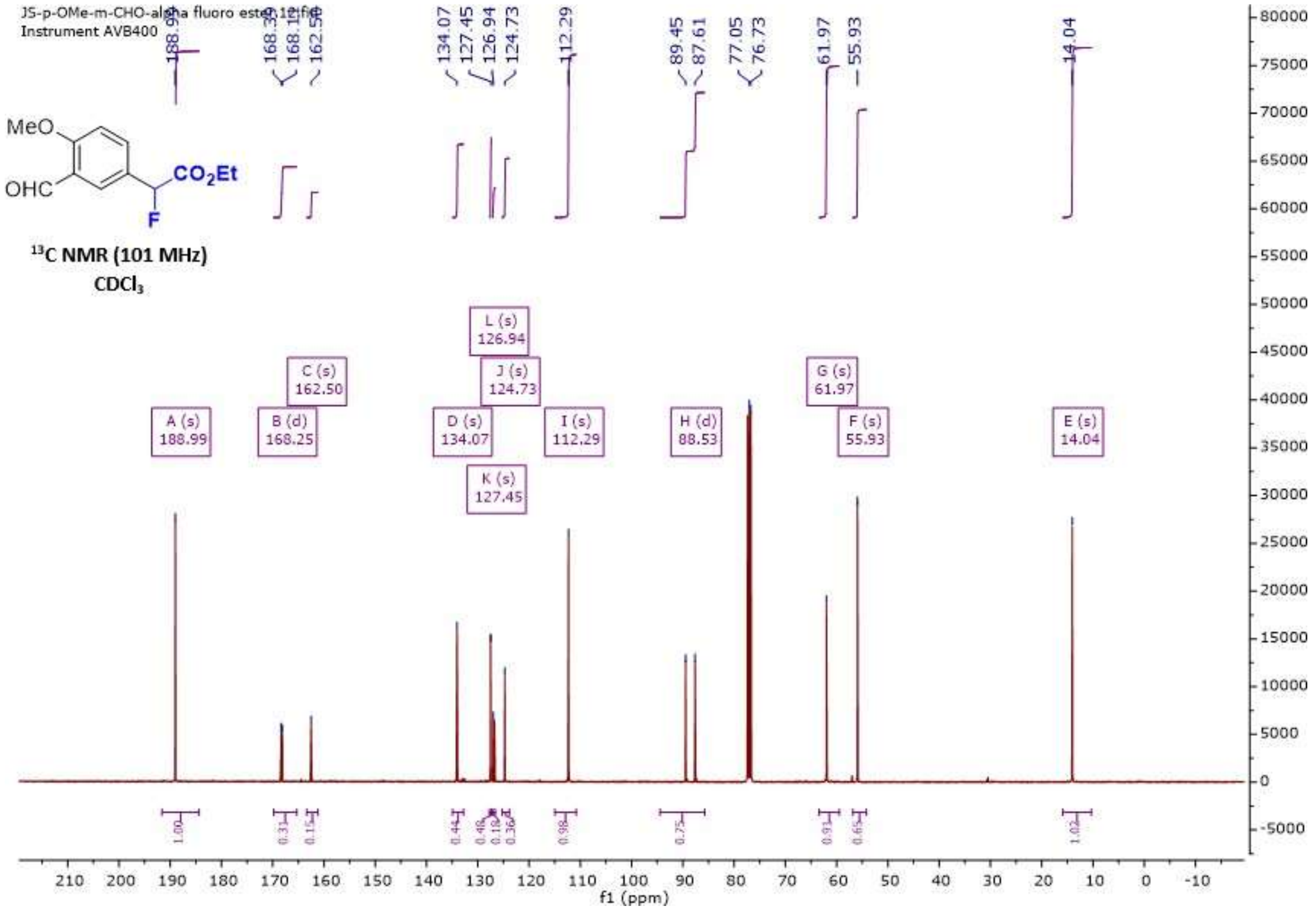

JS-p-OMe-m-CHO-alpha fluoro ester.1.fid
Instrument AVB400
MeO
OHC
CO2Et
F
13C NMR (101 MHz)
CDCl3
188.99
168.39
168.13
162.50
134.07
127.45
126.94
124.73
112.29
89.45
87.61
77.05
76.73
61.97
55.93
14.04
A (s) 188.99
B (d) 168.25
C (s) 162.50
D (s) 134.07
E (s) 14.04
F (s) 55.93
G (s) 61.97
H (d) 88.53
I (s) 112.29
J (s) 124.73
K (s) 127.45
L (s) 126.94
1.00
0.31
0.15
0.44
0.48
0.18
0.36
0.98
0.75
0.93
0.65
1.02
f1 (ppm)

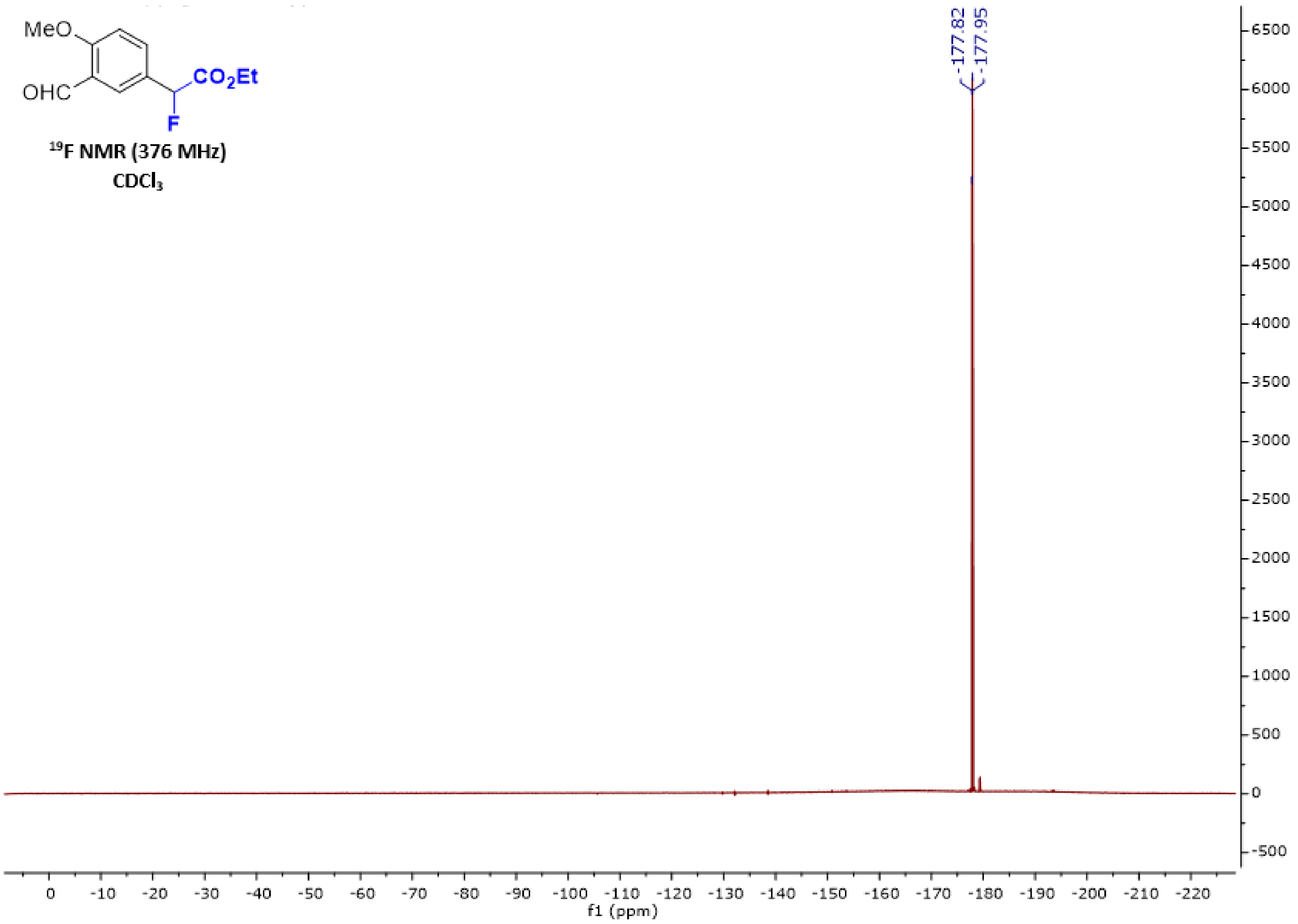
MeO
OHC
CO2Et
F
19F NMR (376 MHz)
CDCl3
-177.82
-177.95
6500
6000
5500
5000
4500
4000
3500
3000
2500
2000
1500
1000
500
0
-500
0 -10 -20 -30 -40 -50 -60 -70 -80 -90 -100 -110 -120 -130 -140 -150 -160 -170 -180 -190 -200 -210 -220
f1 (ppm)

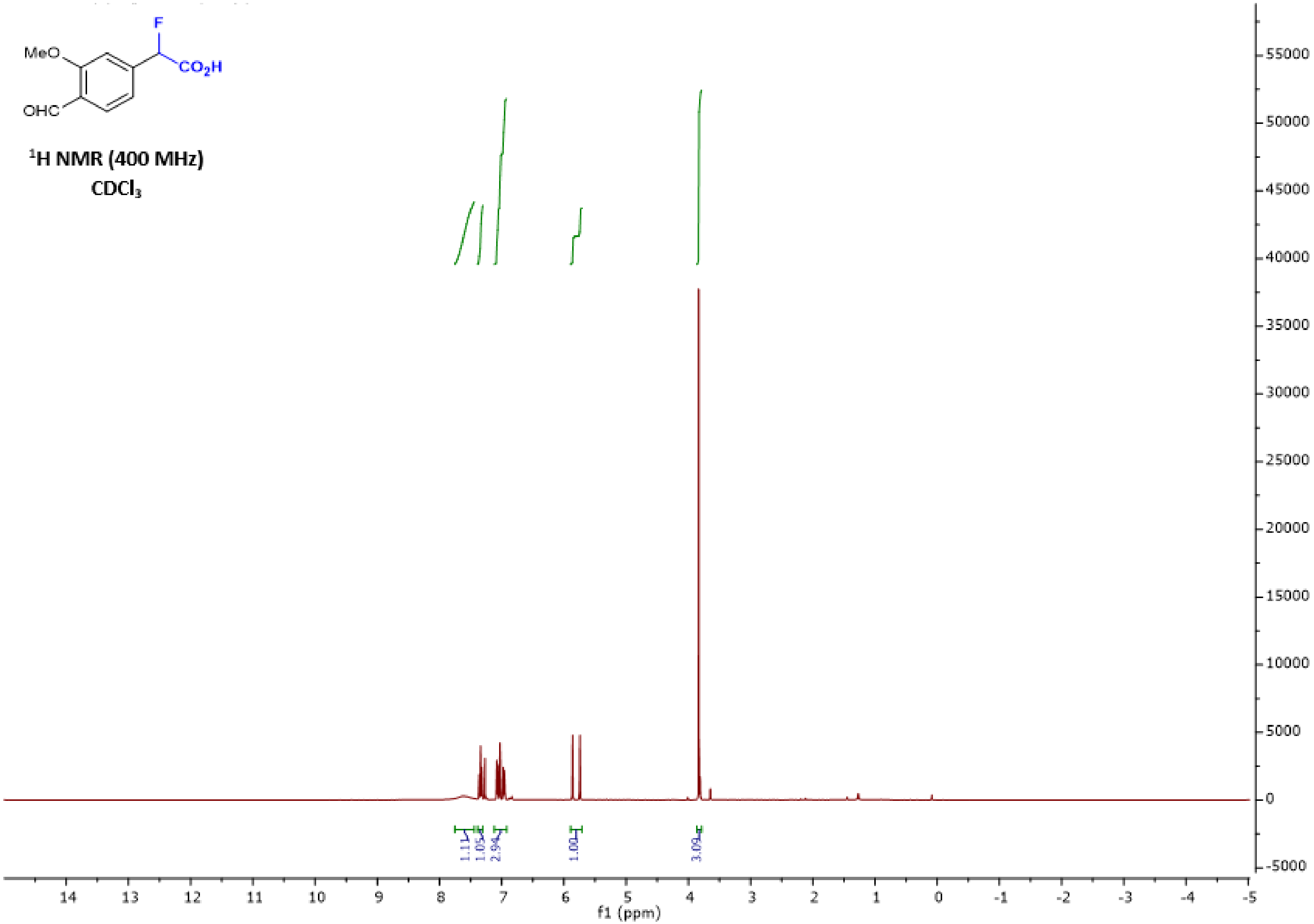
F
MeO
CO2H
OHC
1H NMR (400 MHz)
CDCl3
1.11
1.05
2.94
1.00
3.09
f1 (ppm)

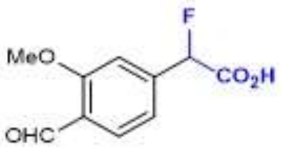


$^{19}$F NMR (376 MHz)
$CDCl_3$

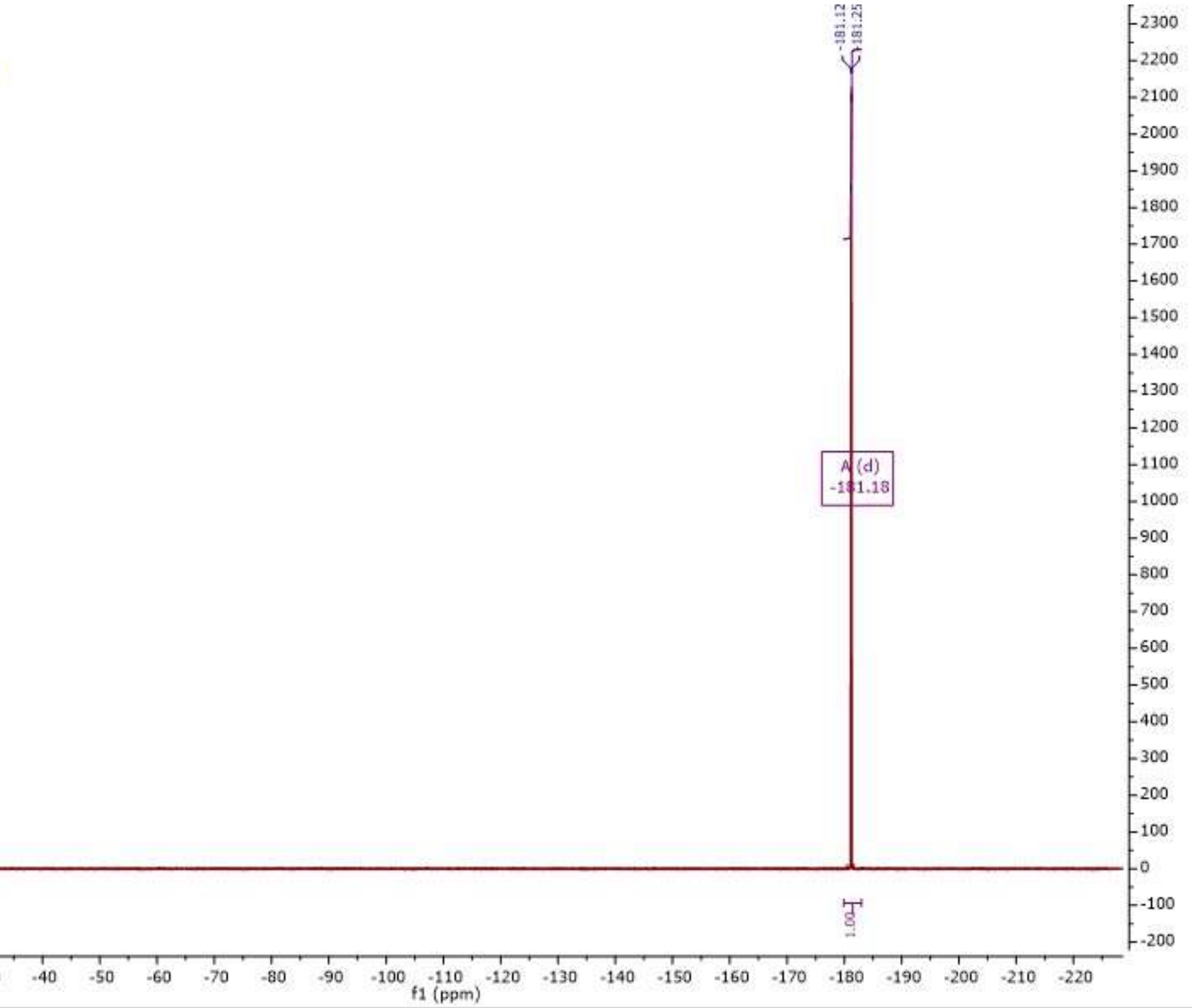

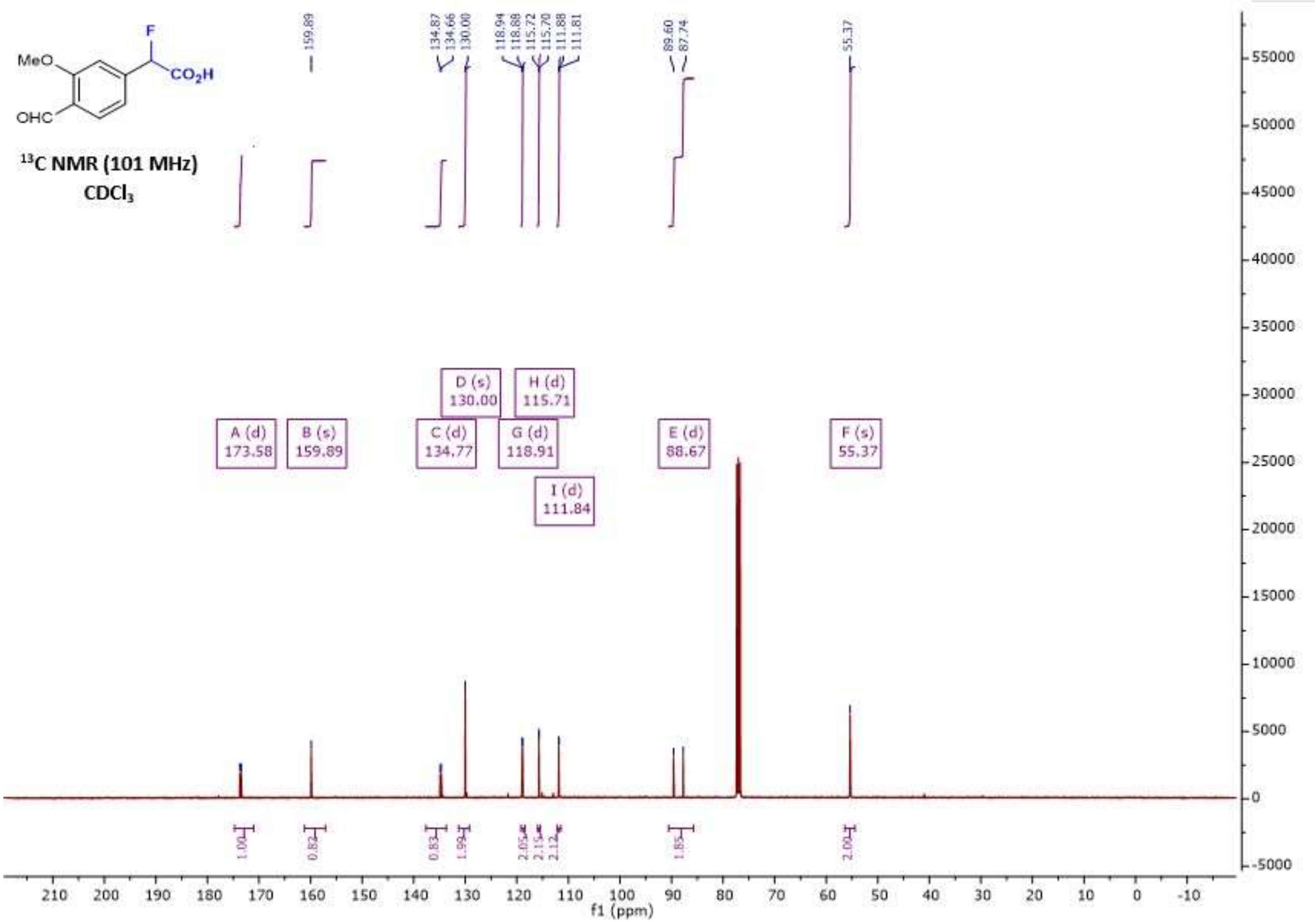

MeO
OHC
F
CO2H
13C NMR (101 MHz)
CDCl3
159.89
134.87
134.66
130.00
118.94
118.88
115.72
115.70
111.88
111.81
89.60
87.74
55.37
A (d) 173.58
B (s) 159.89
C (d) 134.77
D (s) 130.00
G (d) 118.91
H (d) 115.71
I (d) 111.84
E (d) 88.67
F (s) 55.37
1.00
0.82
0.83
1.99
2.05
2.15
2.12
1.85
2.00
f1 (ppm)

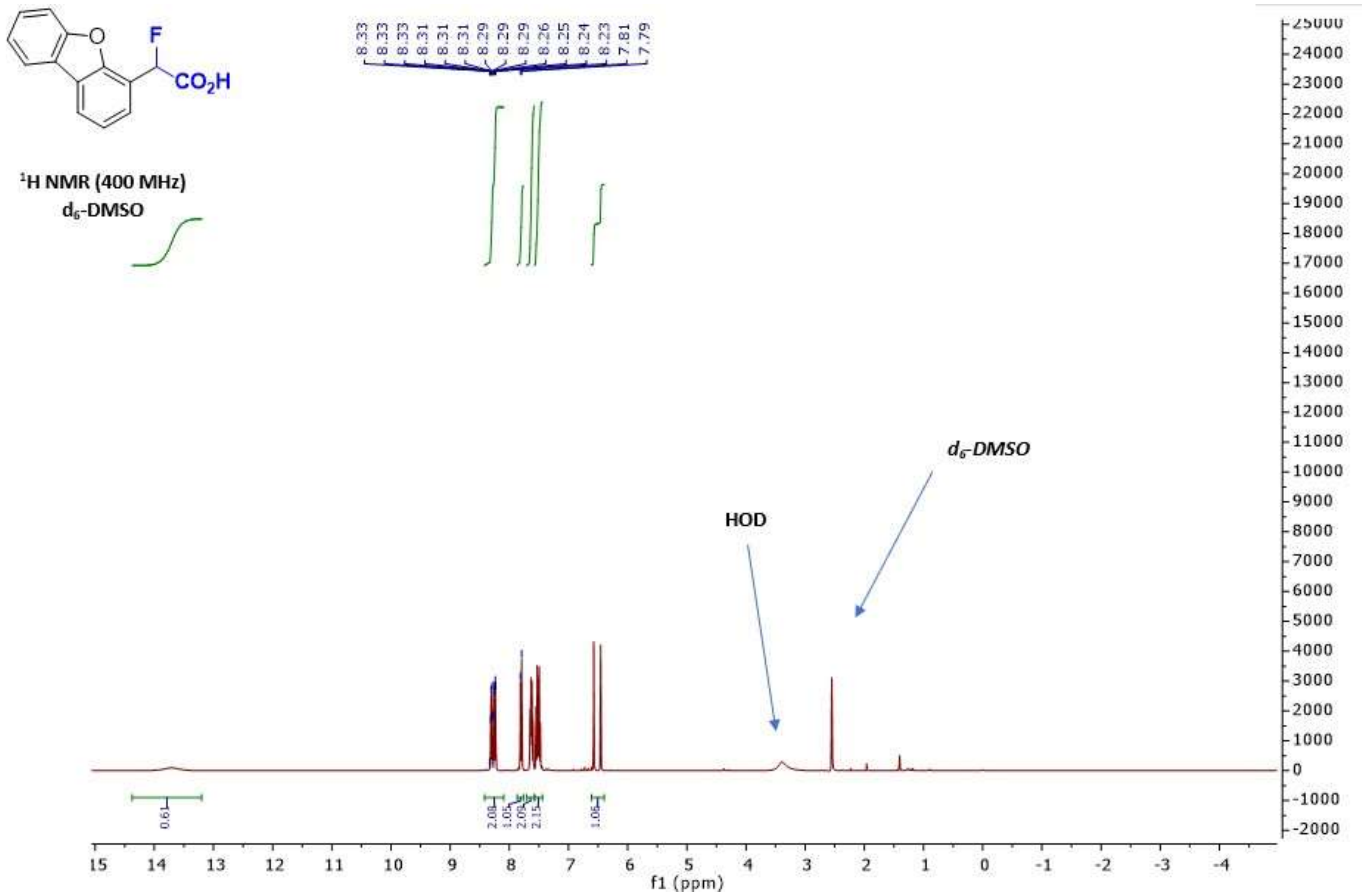

CO2H
F
1H NMR (400 MHz)
d6-DMSO
8.33
8.33
8.33
8.31
8.31
8.31
8.29
8.29
8.29
8.26
8.25
8.24
8.23
7.81
7.79
HOD
d6-DMSO
0.61
2.08
1.05
2.09
2.15
1.06
f1 (ppm)

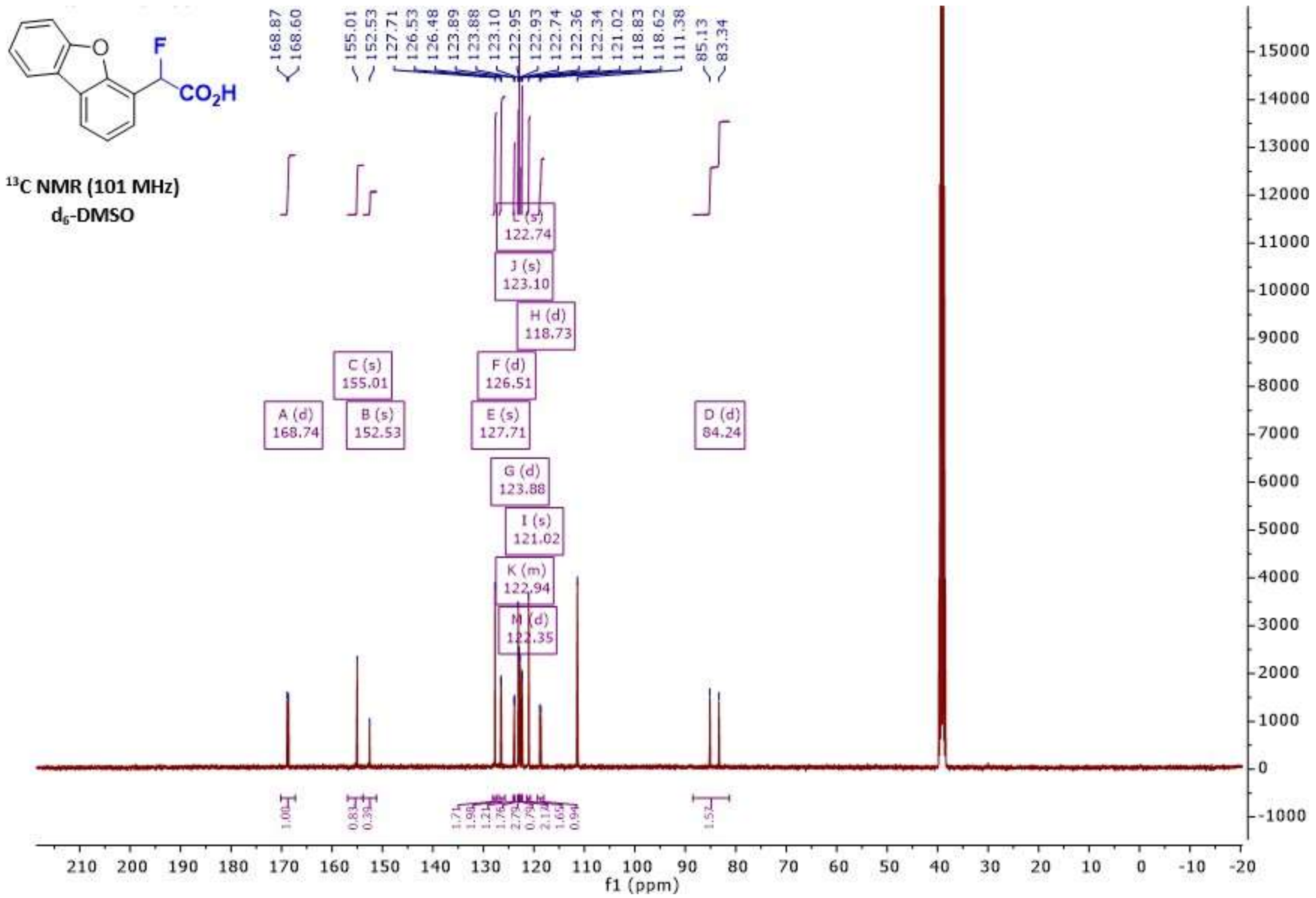

F
$CO_2H$
$^{13}C$ NMR (101 MHz)
$d_6$-DMSO
168.87
168.60
155.01
152.53
127.71
126.53
126.48
123.89
123.88
123.10
122.95
122.93
122.74
122.36
122.34
121.02
118.83
118.62
111.38
85.13
83.34
A (d) 168.74
B (s) 152.53
C (s) 155.01
D (d) 84.24
E (s) 127.71
F (d) 126.51
G (d) 123.88
H (d) 118.73
I (s) 121.02
J (s) 123.10
K (m) 122.94
L (s) 122.74
M (d) 122.35
1.00
0.83
0.39
1.71
1.98
1.21
1.76
2.79
0.79
2.14
1.69
0.94
1.52
f1 (ppm)

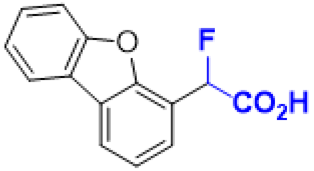

$^{19}F$ NMR (376 MHz)
$d_6$-DMSO

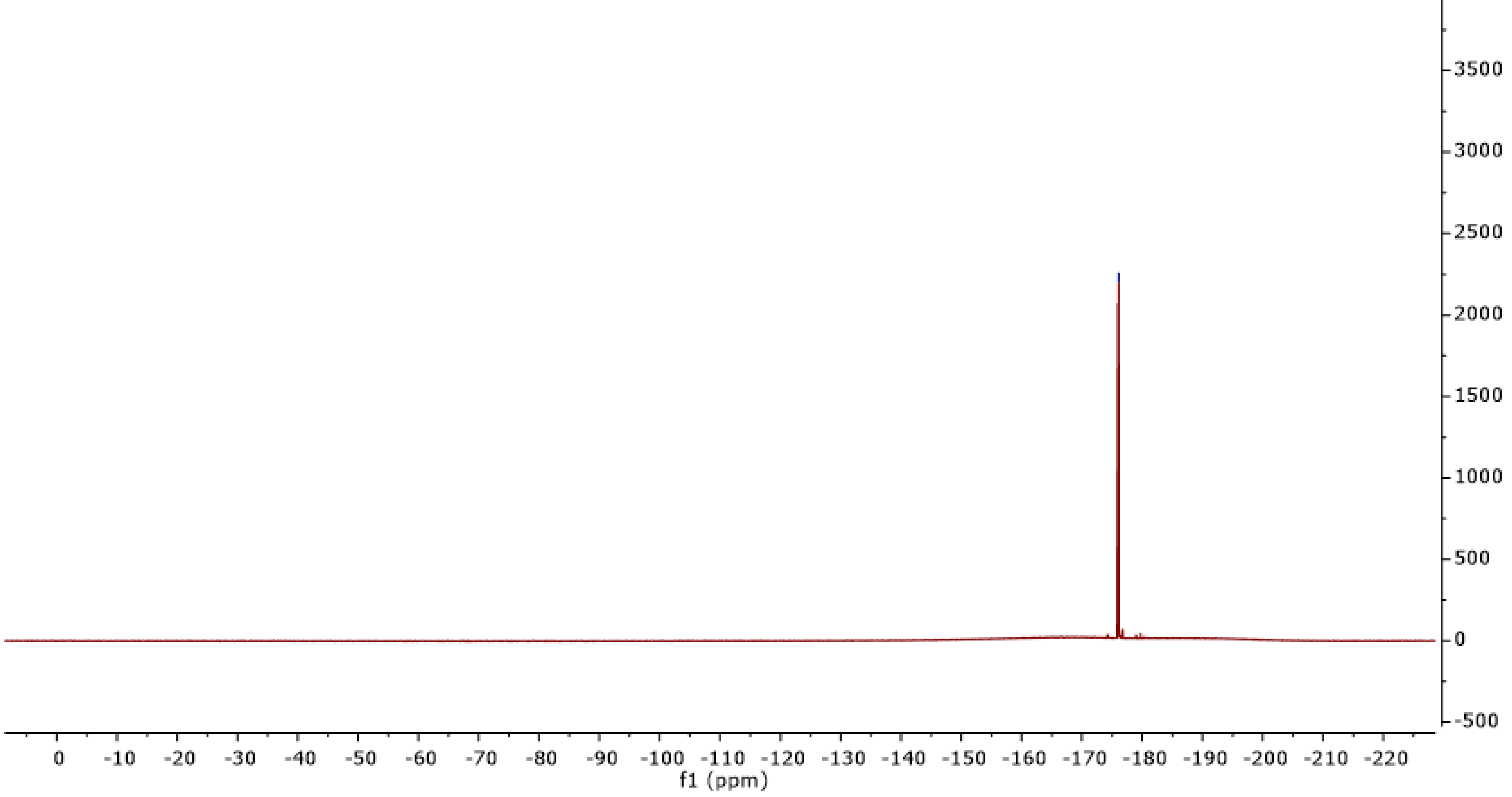

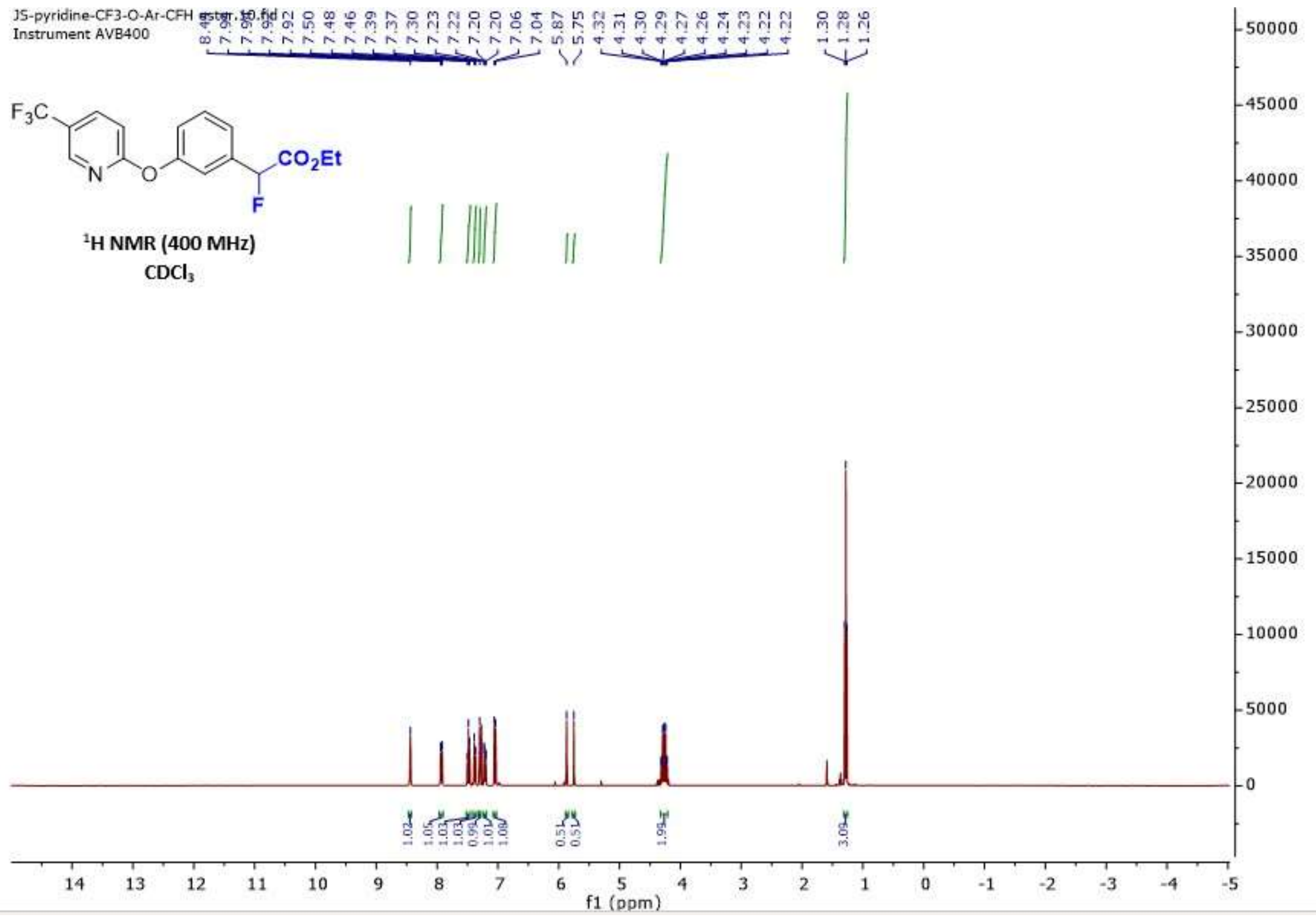
JS-pyridine-CF3-O-Ar-CFH ester.10.fid
Instrument AVB400
$^1$H NMR (400 MHz)
CDCl$_3$
f1 (ppm)

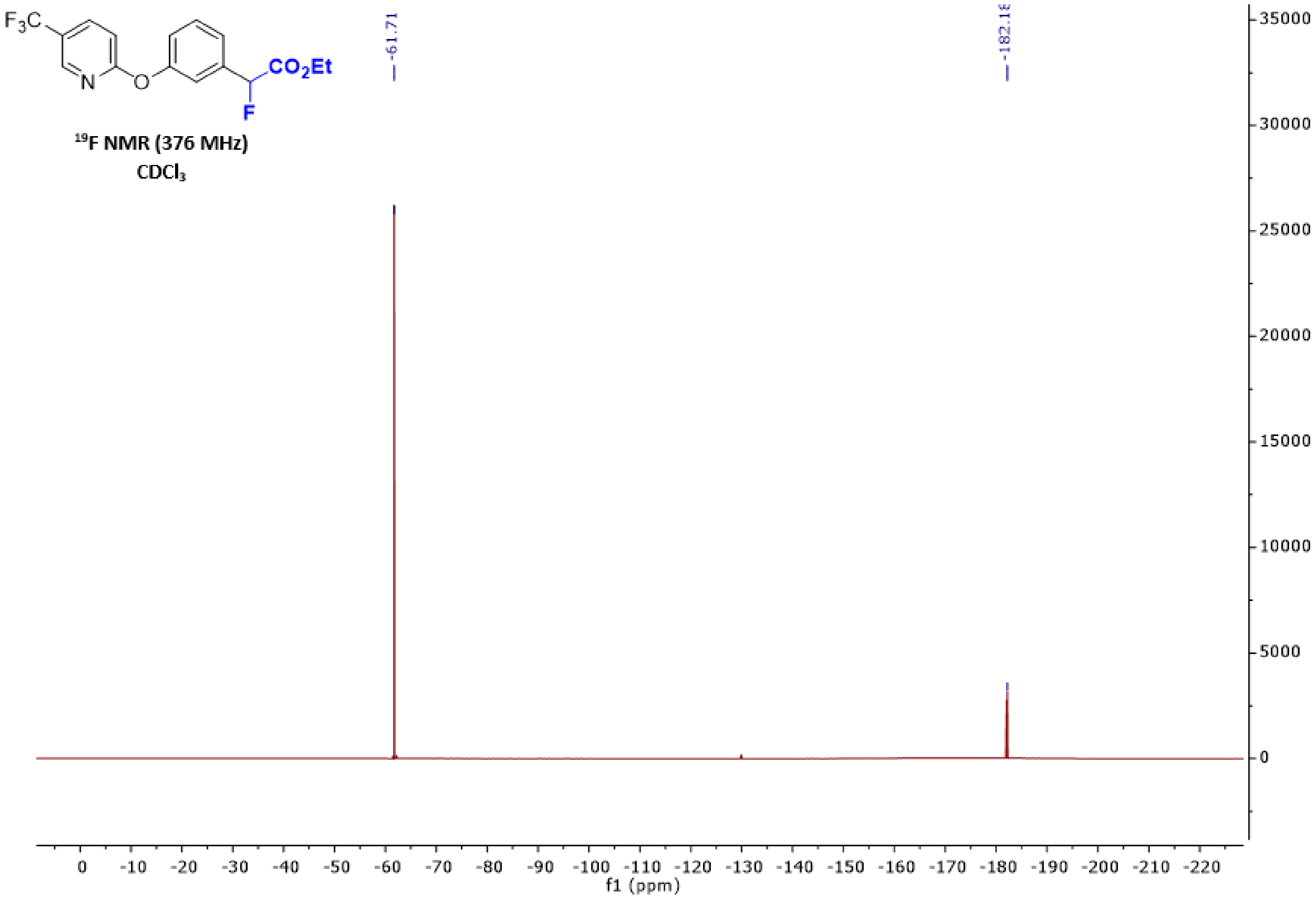

F3C
N
O
CO2Et
F
19F NMR (376 MHz)
CDCl3
-61.71
-182.1[illegible]
35000
30000
25000
20000
15000
10000
5000
0
0 -10 -20 -30 -40 -50 -60 -70 -80 -90 -100 -110 -120 -130 -140 -150 -160 -170 -180 -190 -200 -210 -220
f1 (ppm)

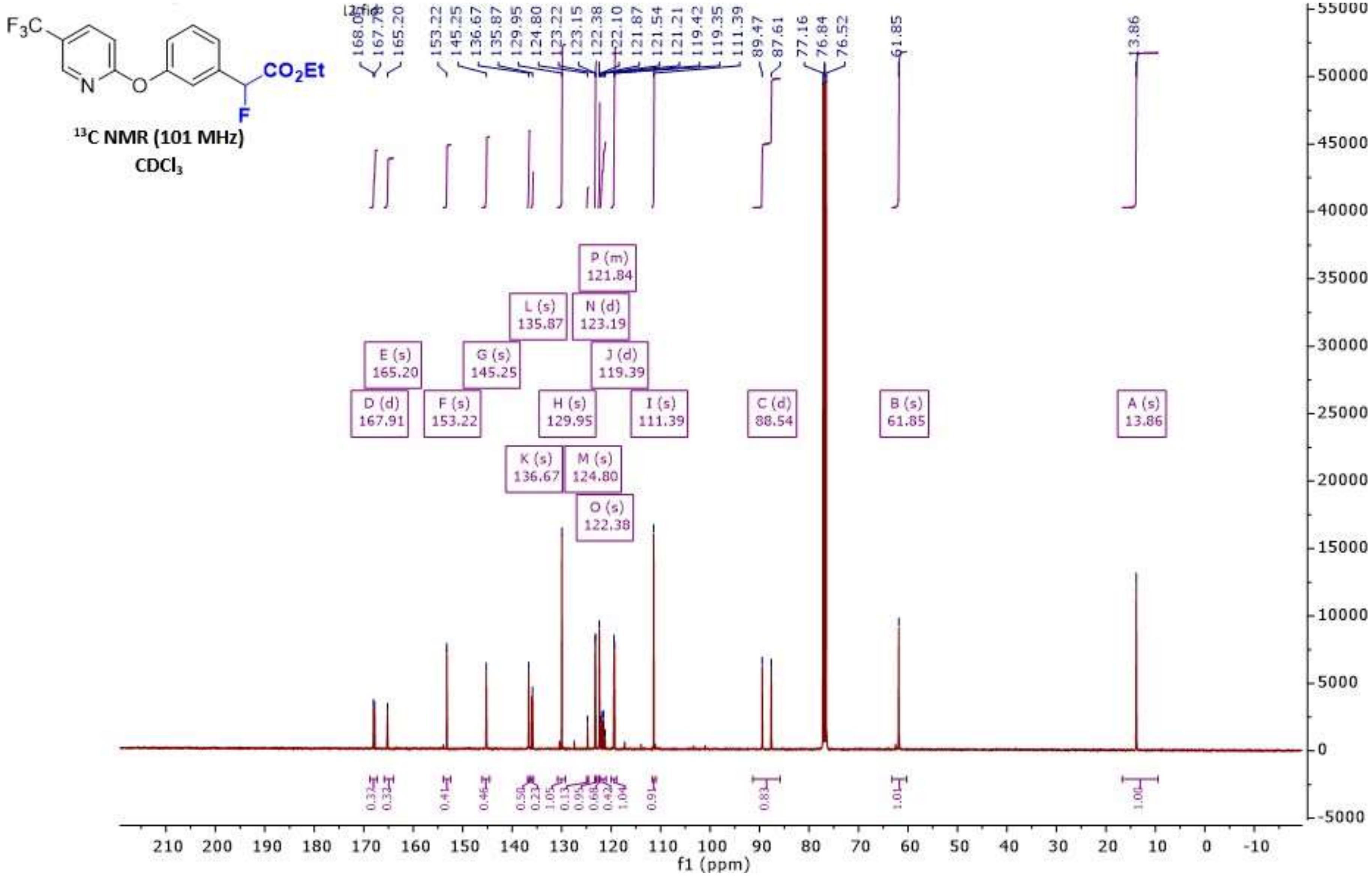

$^{13}$C NMR (101 MHz)
CDCl$_3$
168.03
167.78
165.20
153.22
145.25
136.67
135.87
129.95
124.80
123.22
123.15
122.38
122.10
121.87
121.54
121.21
119.42
119.35
111.39
89.47
87.61
77.16
76.84
76.52
61.85
13.86
P (m) 121.84
L (s) 135.87
N (d) 123.19
E (s) 165.20
G (s) 145.25
J (d) 119.39
D (d) 167.91
F (s) 153.22
H (s) 129.95
I (s) 111.39
C (d) 88.54
B (s) 61.85
A (s) 13.86
K (s) 136.67
M (s) 124.80
O (s) 122.38
f1 (ppm)

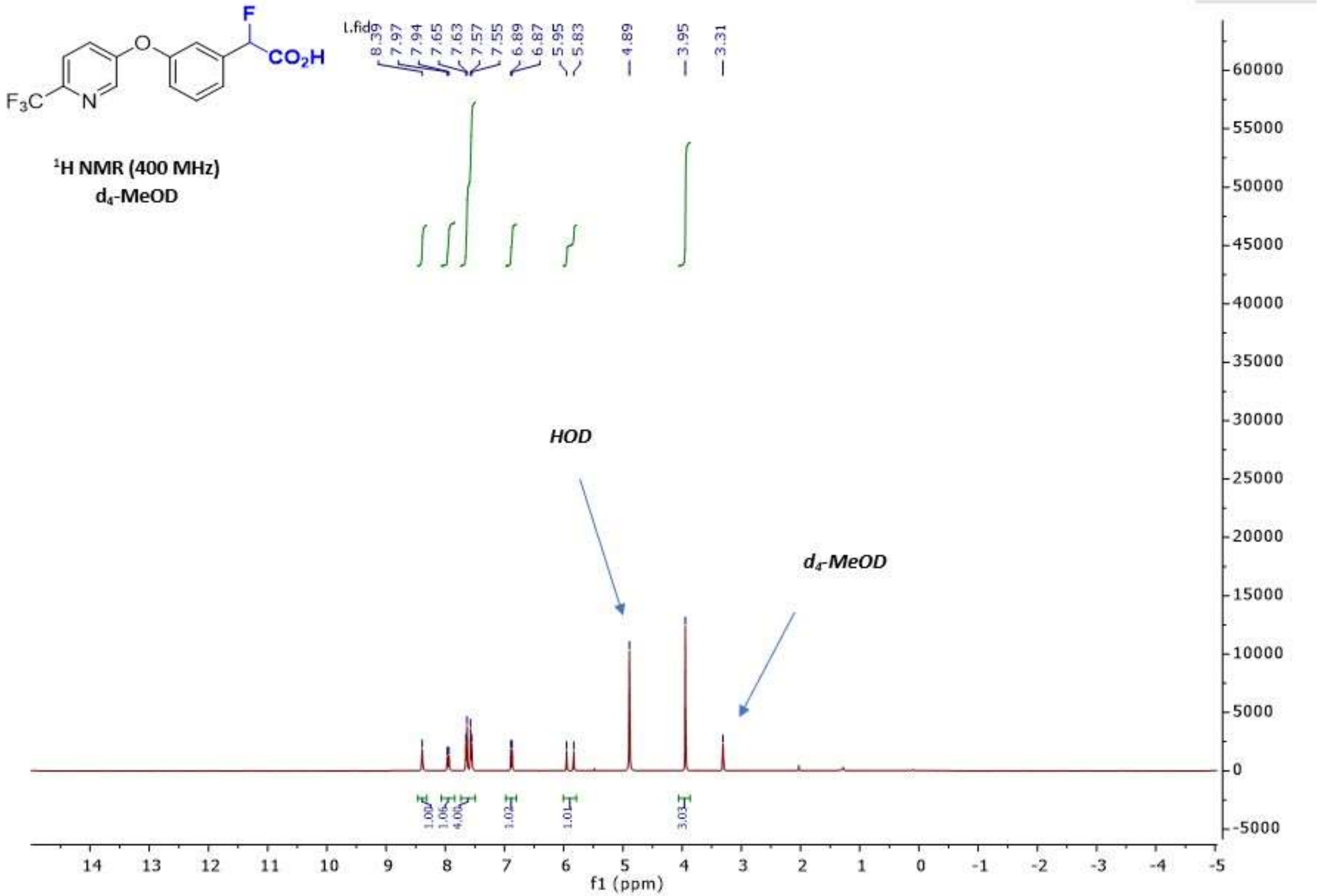
F
O
$CO_2H$
$F_3C$
N
$^{1}$H NMR (400 MHz)
$d_4$-MeOD
1.fid
8.39
7.97
7.94
7.65
7.63
7.57
7.55
6.89
6.87
5.95
5.83
4.89
3.95
3.31
HOD
$d_4$-MeOD
1.00
1.06
4.06
1.02
1.01
3.03
f1 (ppm)

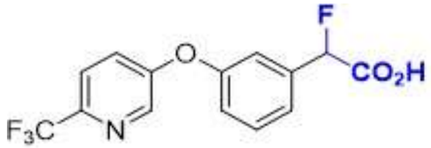


$^{19}$F NMR (376 MHz)
$CDCl_3$

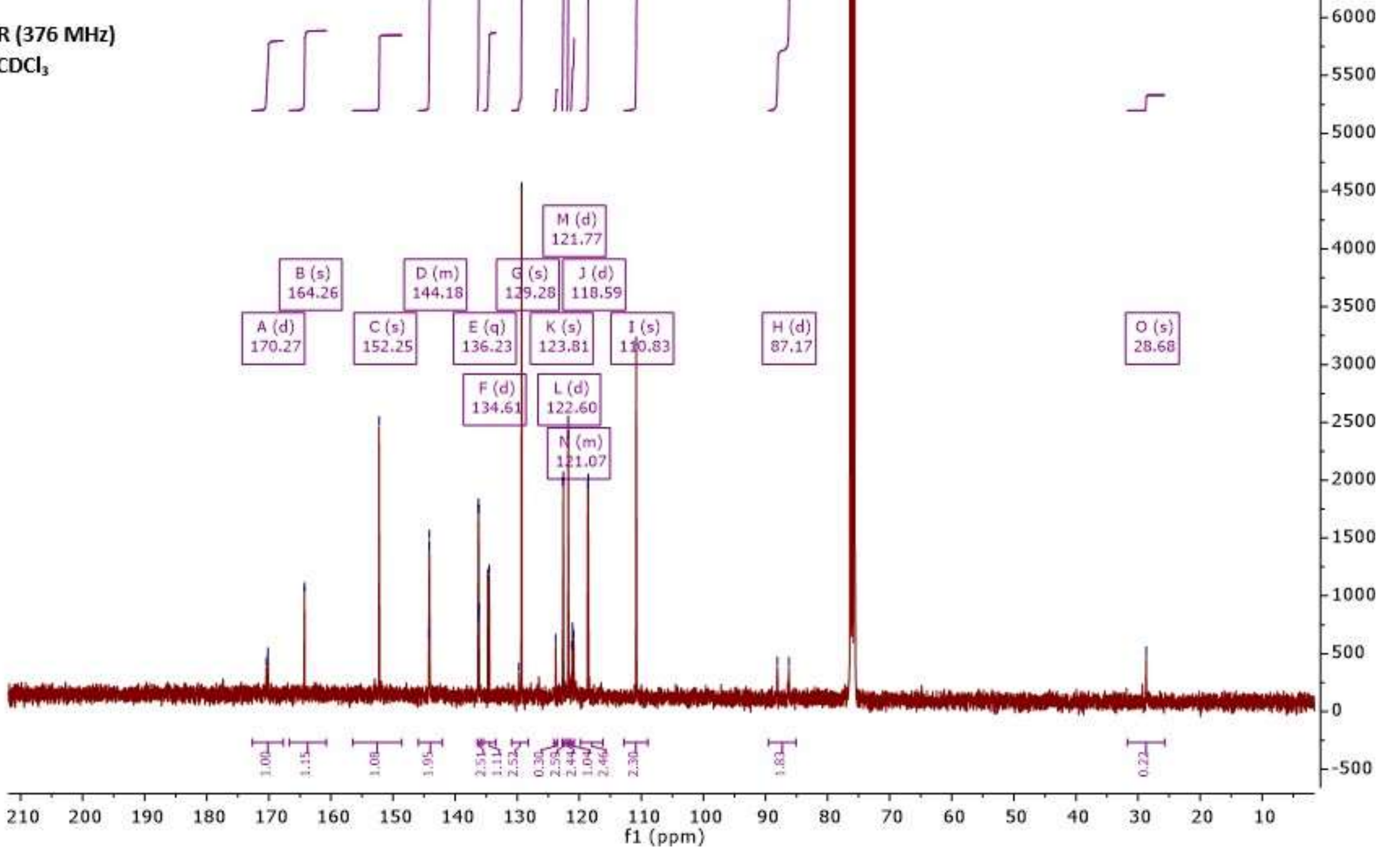

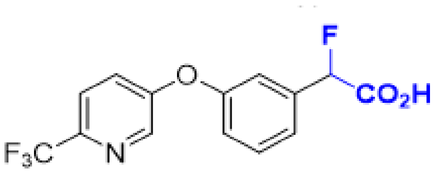

**$^{19}$F NMR (376 MHz)**
**$d_4$-MeOD**

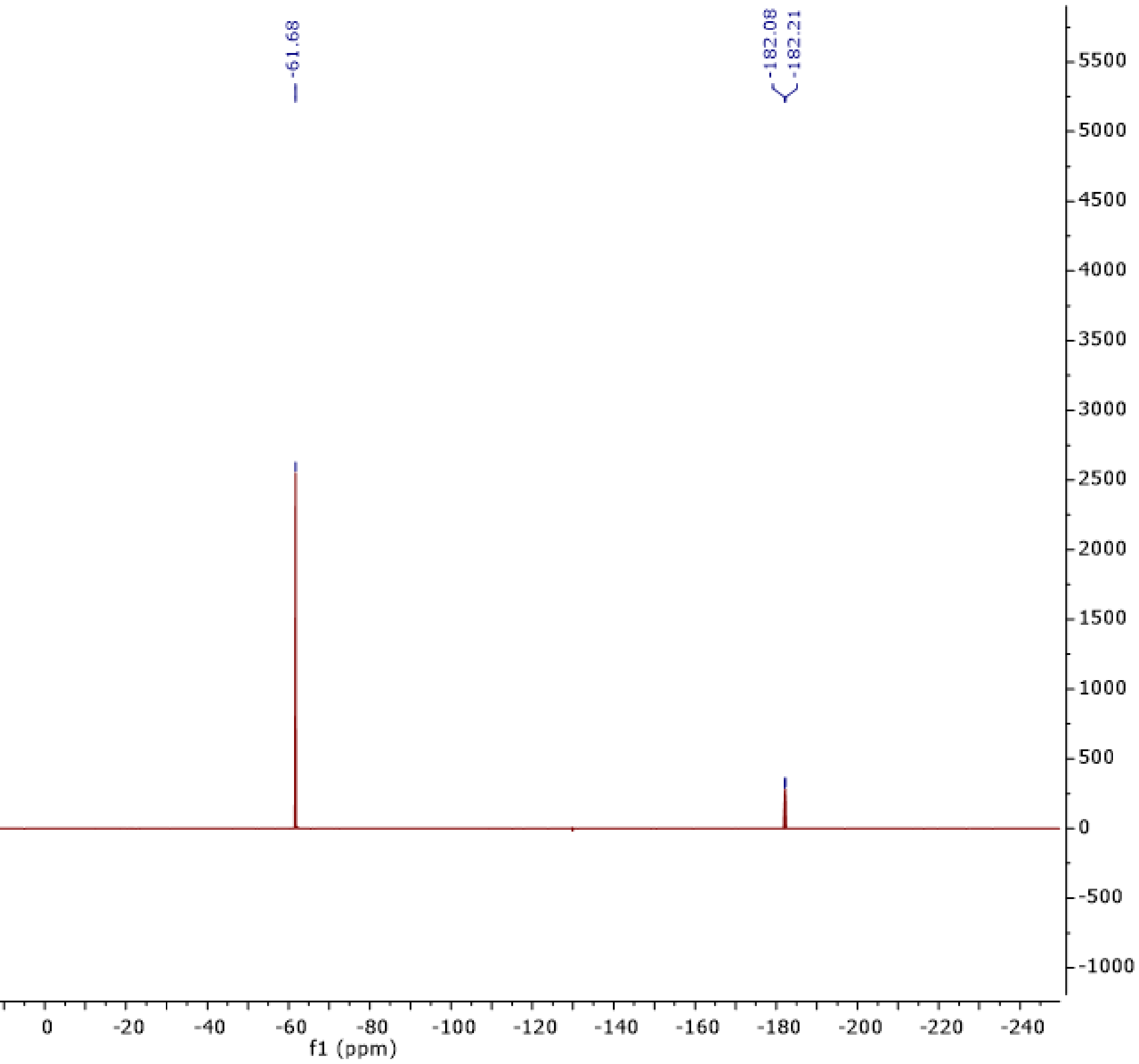

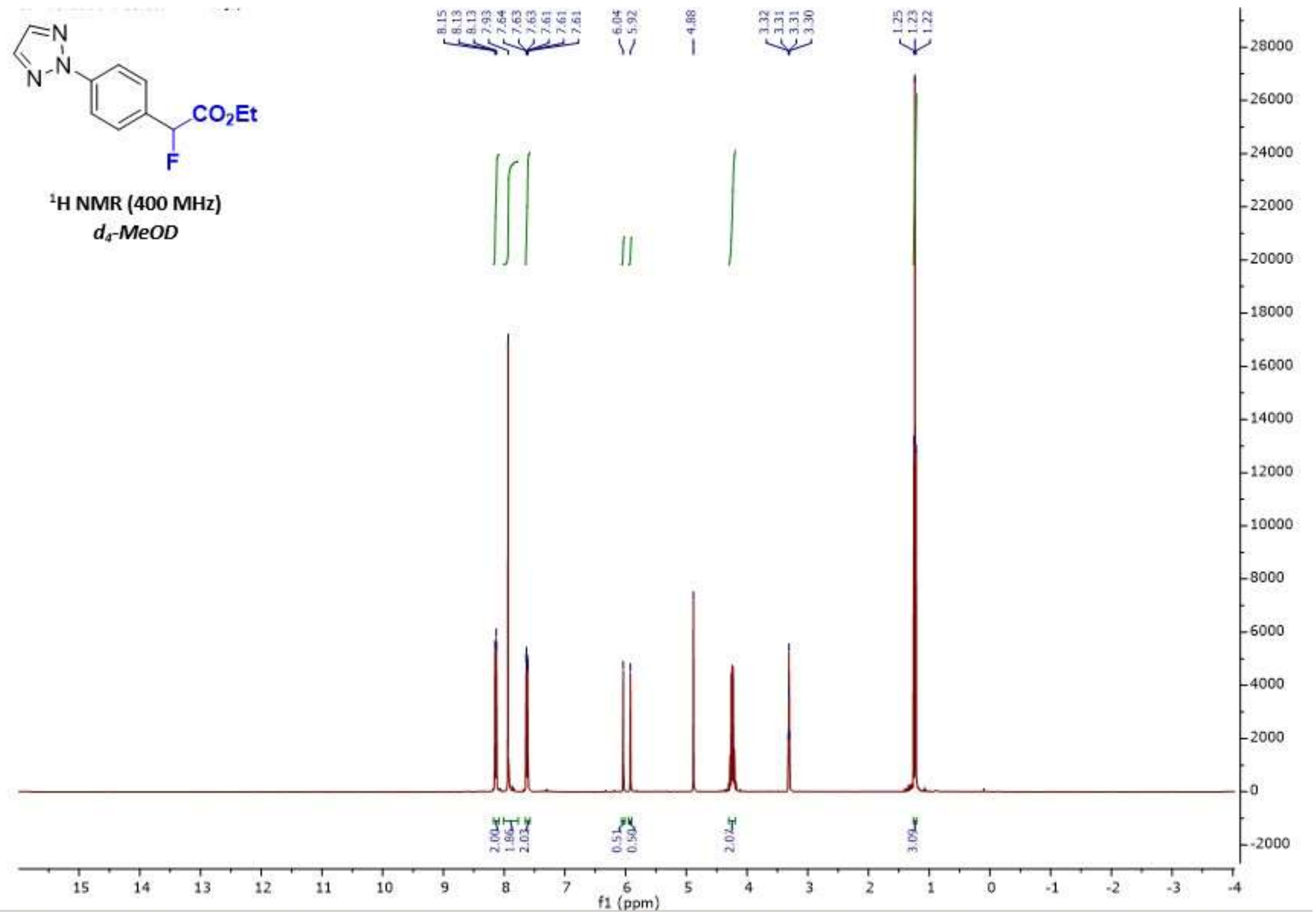
CO2Et
F
1H NMR (400 MHz)
d4-MeOD
8.15
8.13
8.13
7.93
7.64
7.63
7.63
7.61
7.61
7.61
6.04
5.92
4.88
3.32
3.31
3.31
3.30
1.25
1.23
1.22
2.00
1.86
2.03
0.51
0.50
2.07
3.09
f1 (ppm)

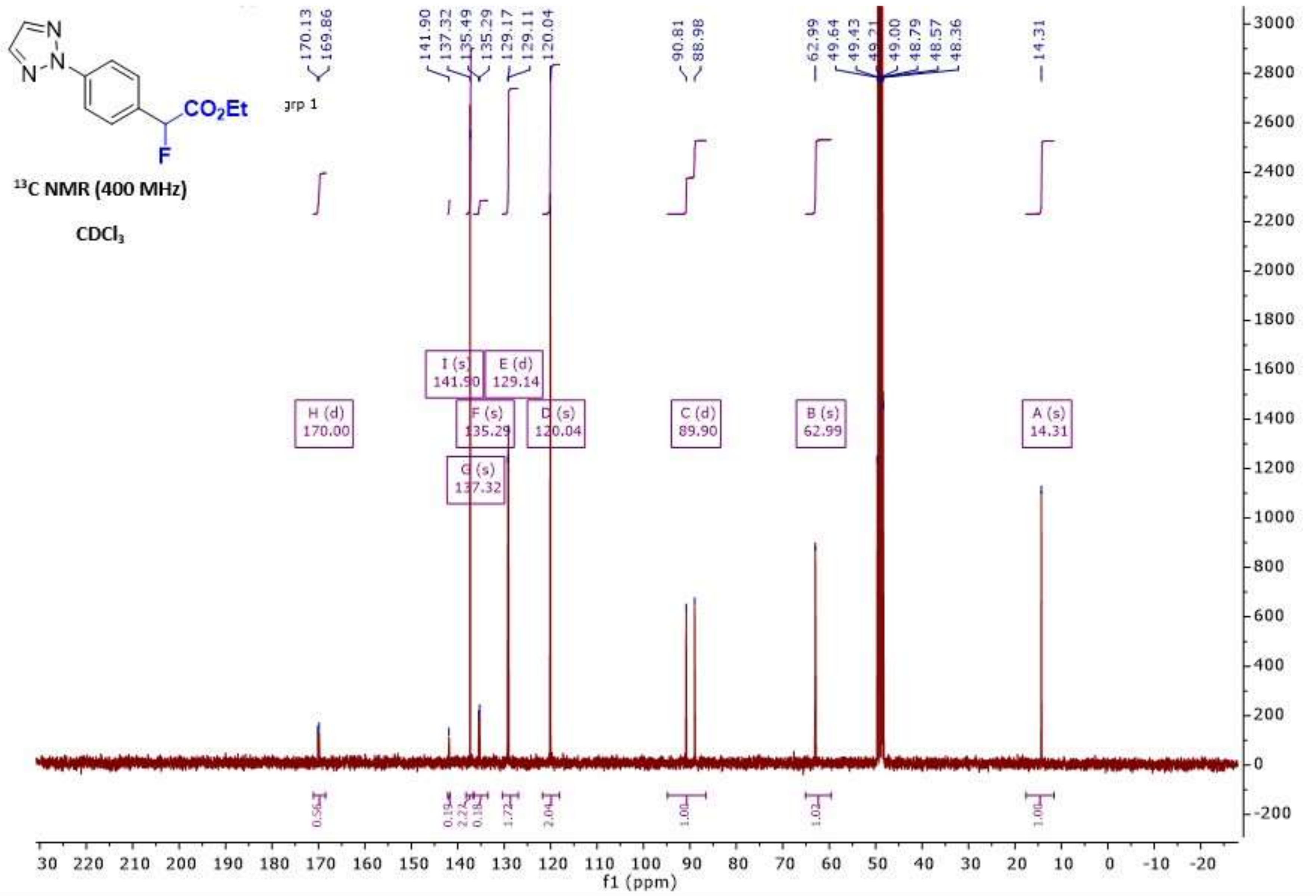

$^{13}$C NMR (400 MHz)
CDCl$_3$
CO$_2$Et
F
170.13
169.86
141.90
137.32
135.49
135.29
129.17
129.11
120.04
90.81
88.98
62.99
49.64
49.43
49.21
49.00
48.79
48.57
48.36
14.31
H (d) 170.00
I (s) 141.90
E (d) 129.14
F (s) 135.29
G (s) 137.32
D (s) 120.04
C (d) 89.90
B (s) 62.99
A (s) 14.31
f1 (ppm)

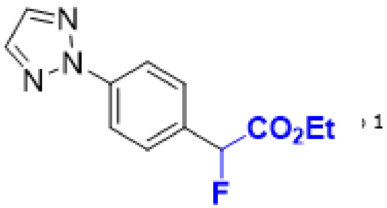

$^{19}$F NMR (376 MHz)

*$d_4$-MeOD*

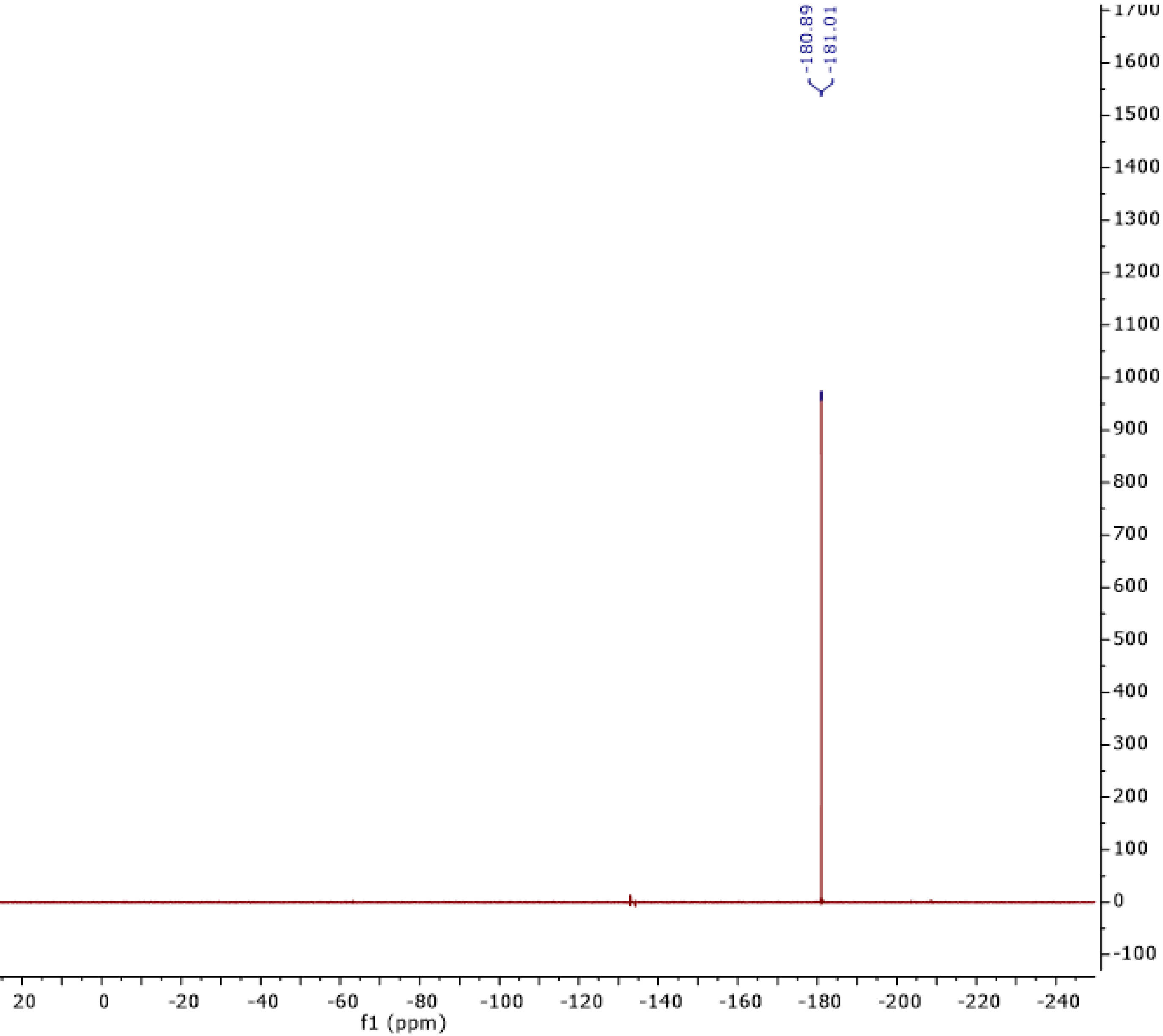

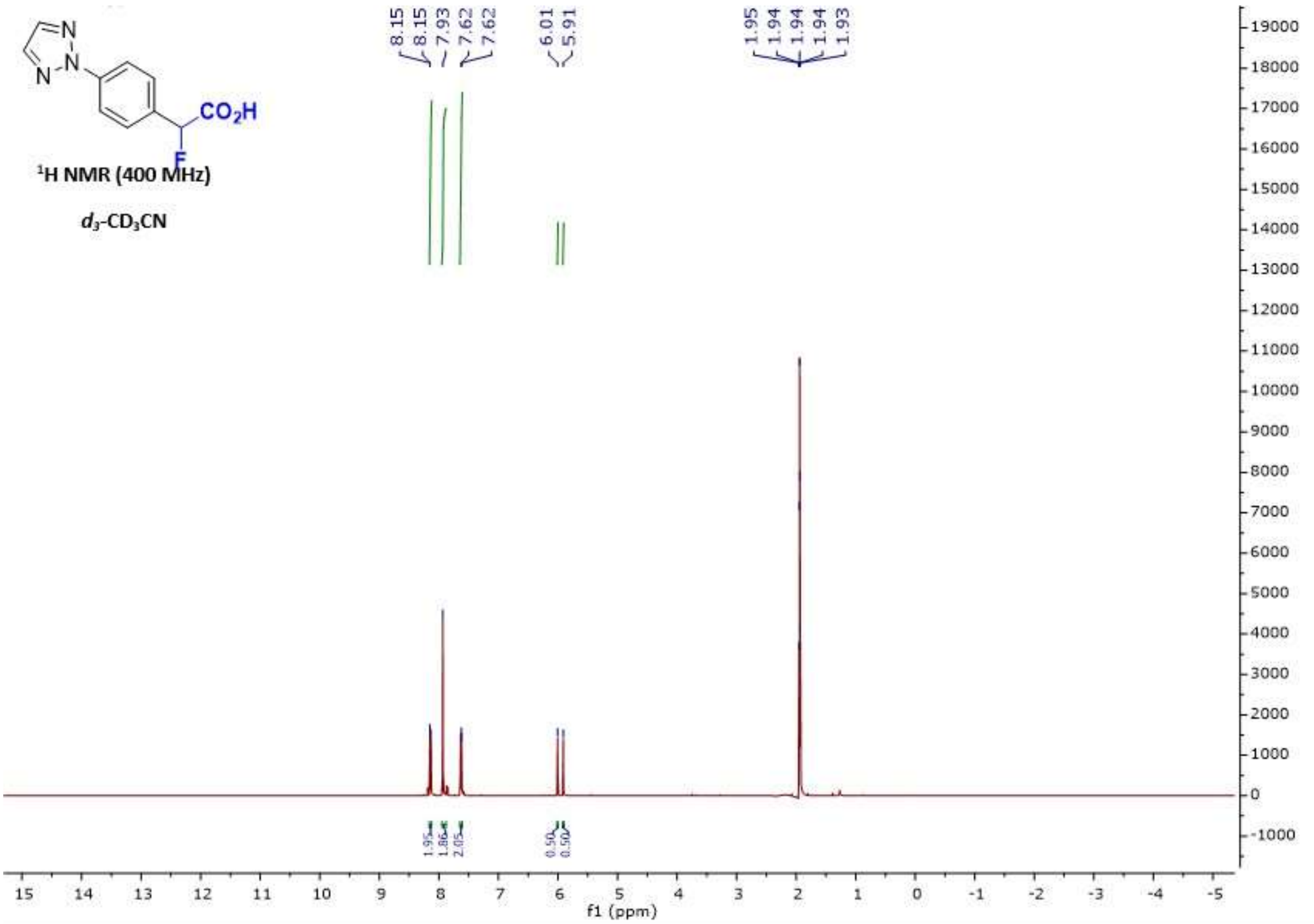

CO2H
F
1H NMR (400 MHz)
d3-CD3CN
8.15
8.15
7.93
7.62
7.62
6.01
5.91
1.95
1.94
1.94
1.94
1.93
1.95
1.86
2.05
0.50
0.50
f1 (ppm)

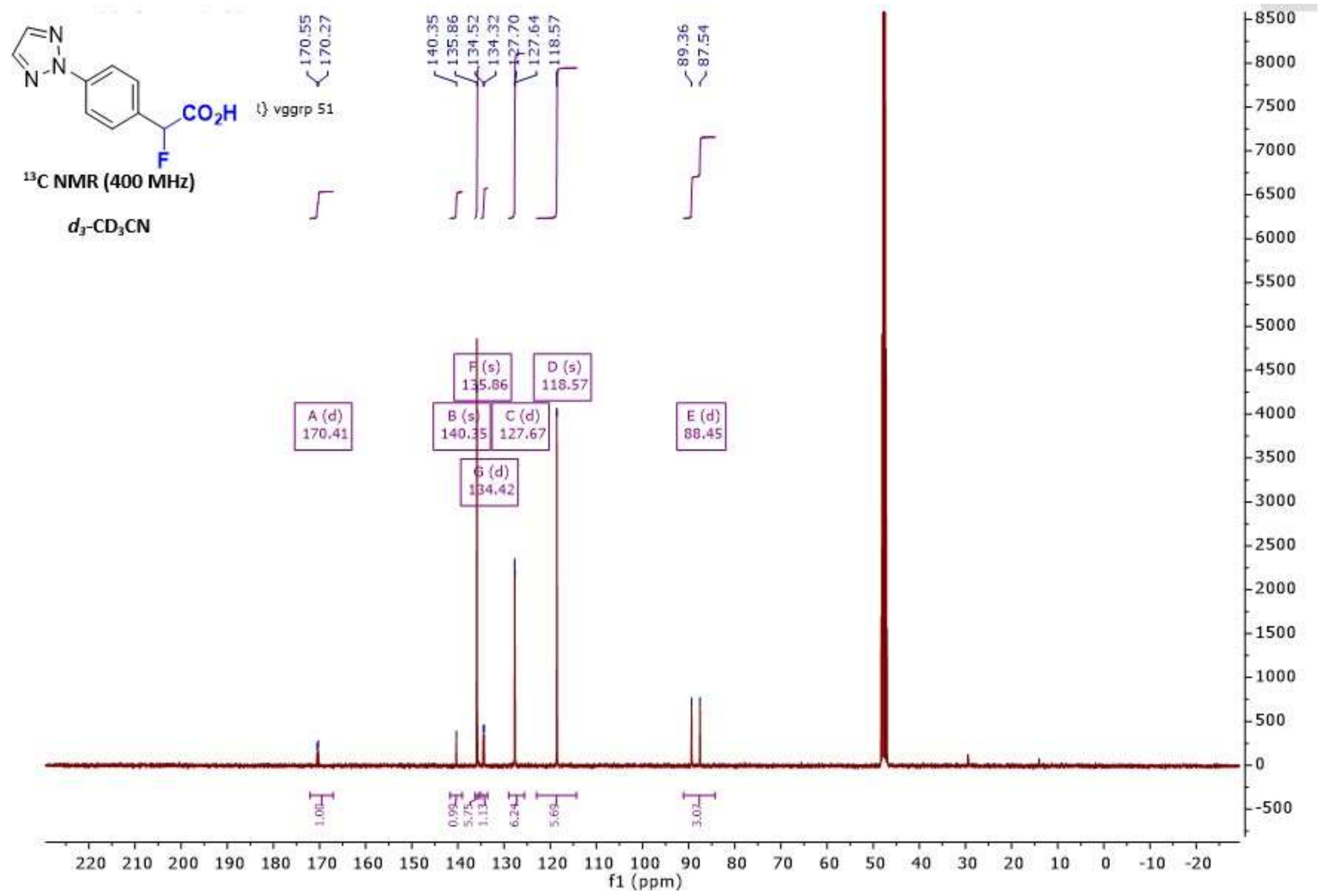

$^{13}$C NMR (400 MHz)
$d_3$-$CD_3CN$
$CO_2H$
F
170.55
170.27
140.35
135.86
134.52
134.32
127.70
127.64
118.57
89.36
87.54
A (d) 170.41
B (s) 140.35
F (s) 135.86
G (d) 134.42
C (d) 127.67
D (s) 118.57
E (d) 88.45
1.00
0.99
5.75
1.13
6.24
5.69
3.02
f1 (ppm)

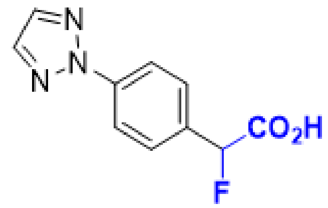


$^{19}$F NMR (376 MHz)

$d_3$-$CD_3CN$

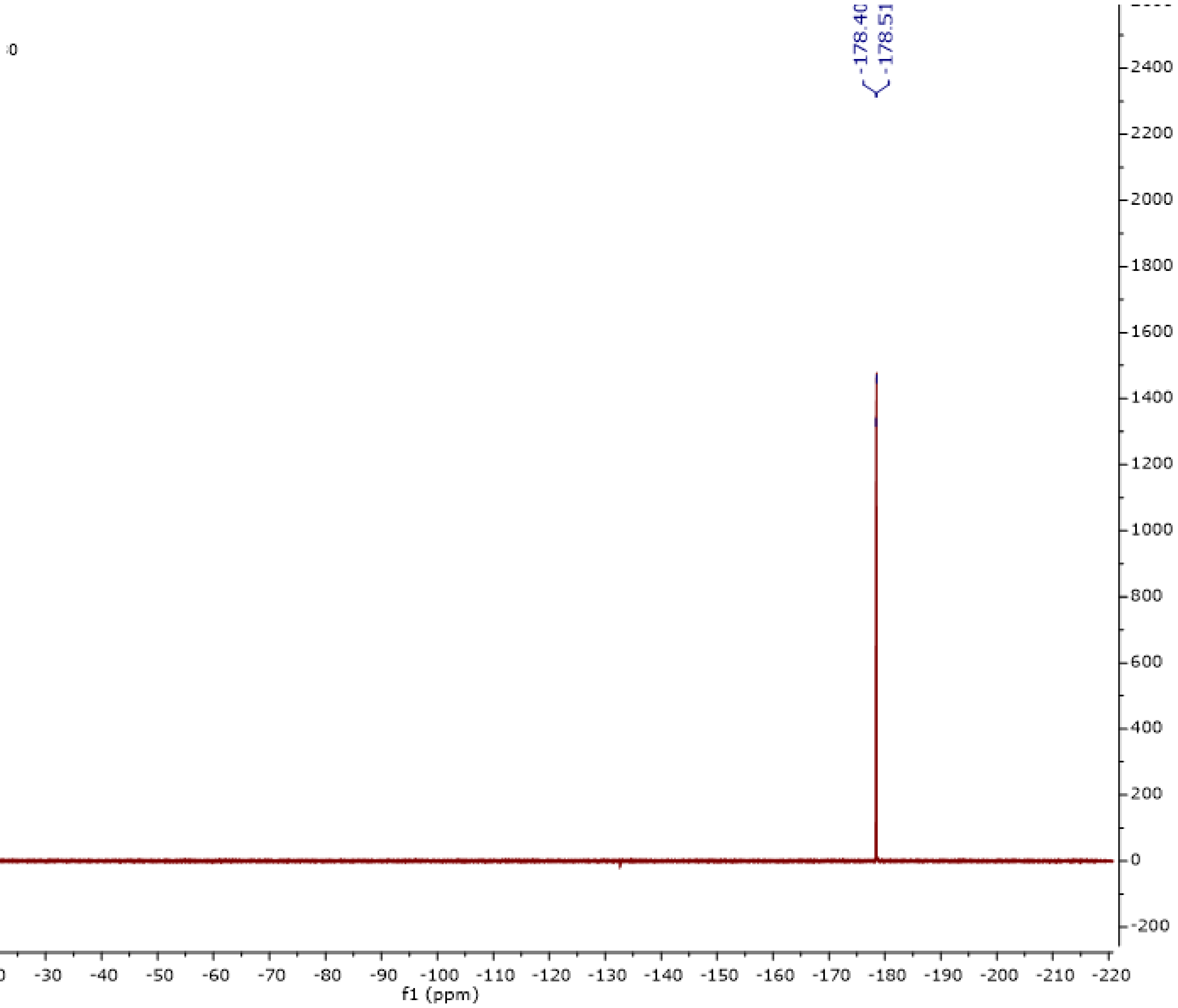

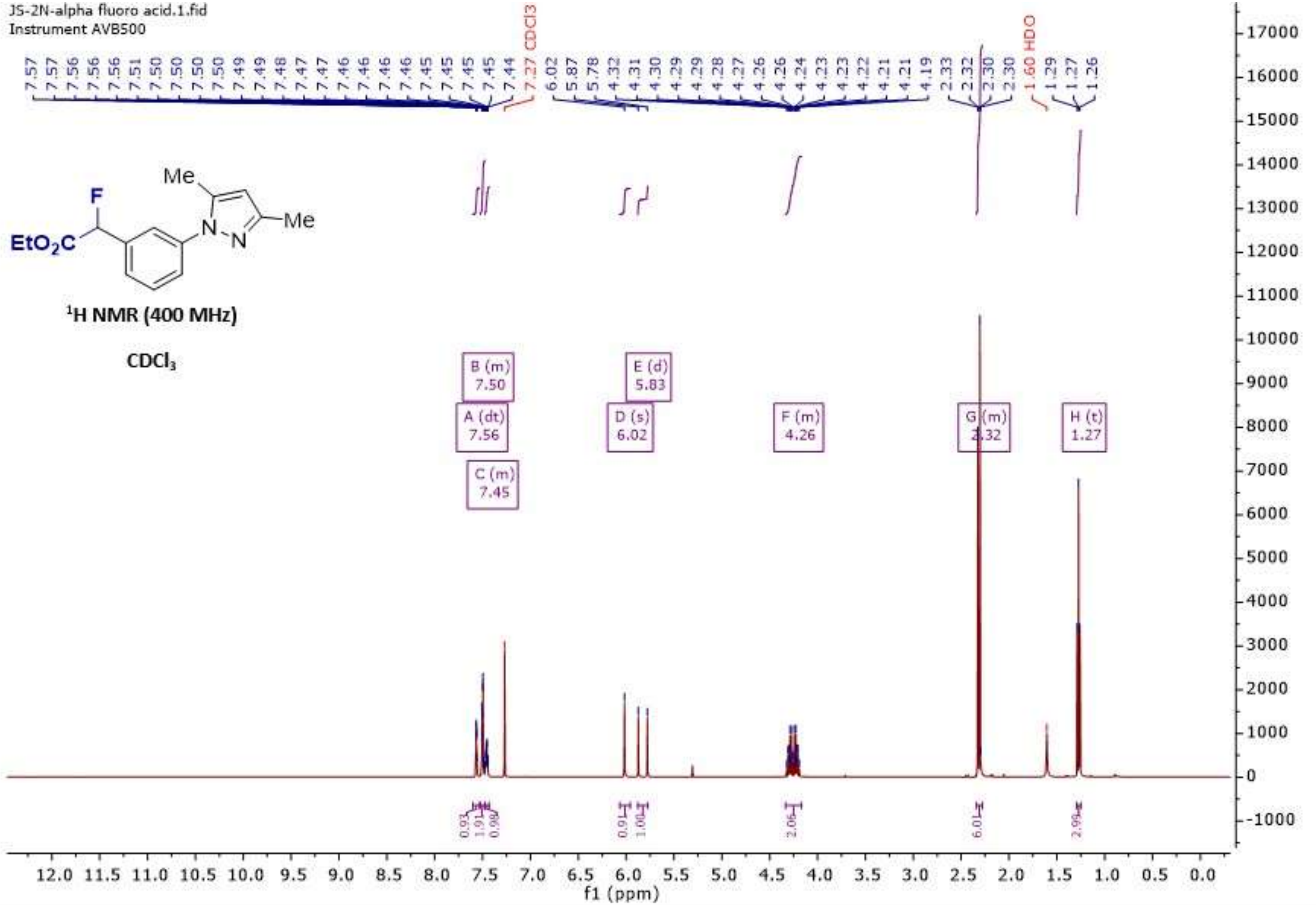
JS-2N-alpha fluoro acid.1.fid
Instrument AVB500
F
EtO2C
Me
Me
N
N
1H NMR (400 MHz)
CDCl3
B (m) 7.50
A (dt) 7.56
C (m) 7.45
E (d) 5.83
D (s) 6.02
F (m) 4.26
G (m) 2.32
H (t) 1.27
7.27 CDCl3
1.60 HDO
f1 (ppm)

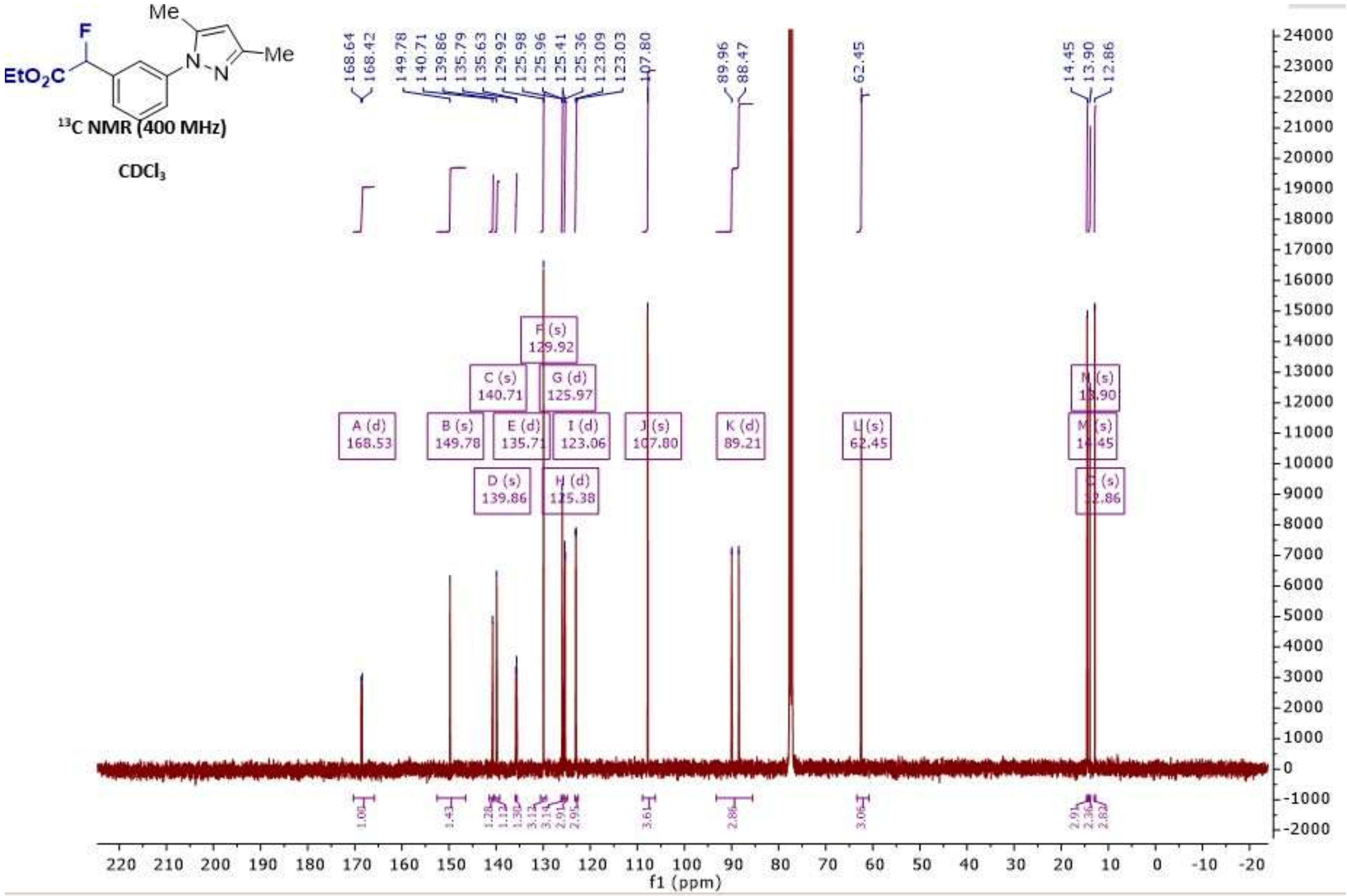

EtO2C
F
Me
Me
N
N
13C NMR (400 MHz)
CDCl3
168.64
168.42
149.78
140.71
139.86
135.79
135.63
129.92
125.98
125.96
125.41
125.36
123.09
123.03
107.80
89.96
88.47
62.45
14.45
13.90
12.86
A (d) 168.53
B (s) 149.78
C (s) 140.71
D (s) 139.86
E (d) 135.71
F (s) 129.92
G (d) 125.97
H (d) 125.38
I (d) 123.06
J (s) 107.80
K (d) 89.21
L (s) 62.45
M (s) 14.45
f1 (ppm)

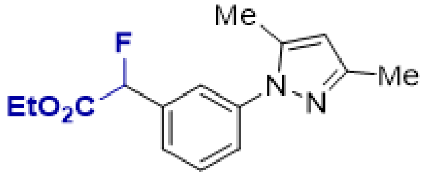

$^{19}$F NMR (376 MHz)

$CDCl_3$

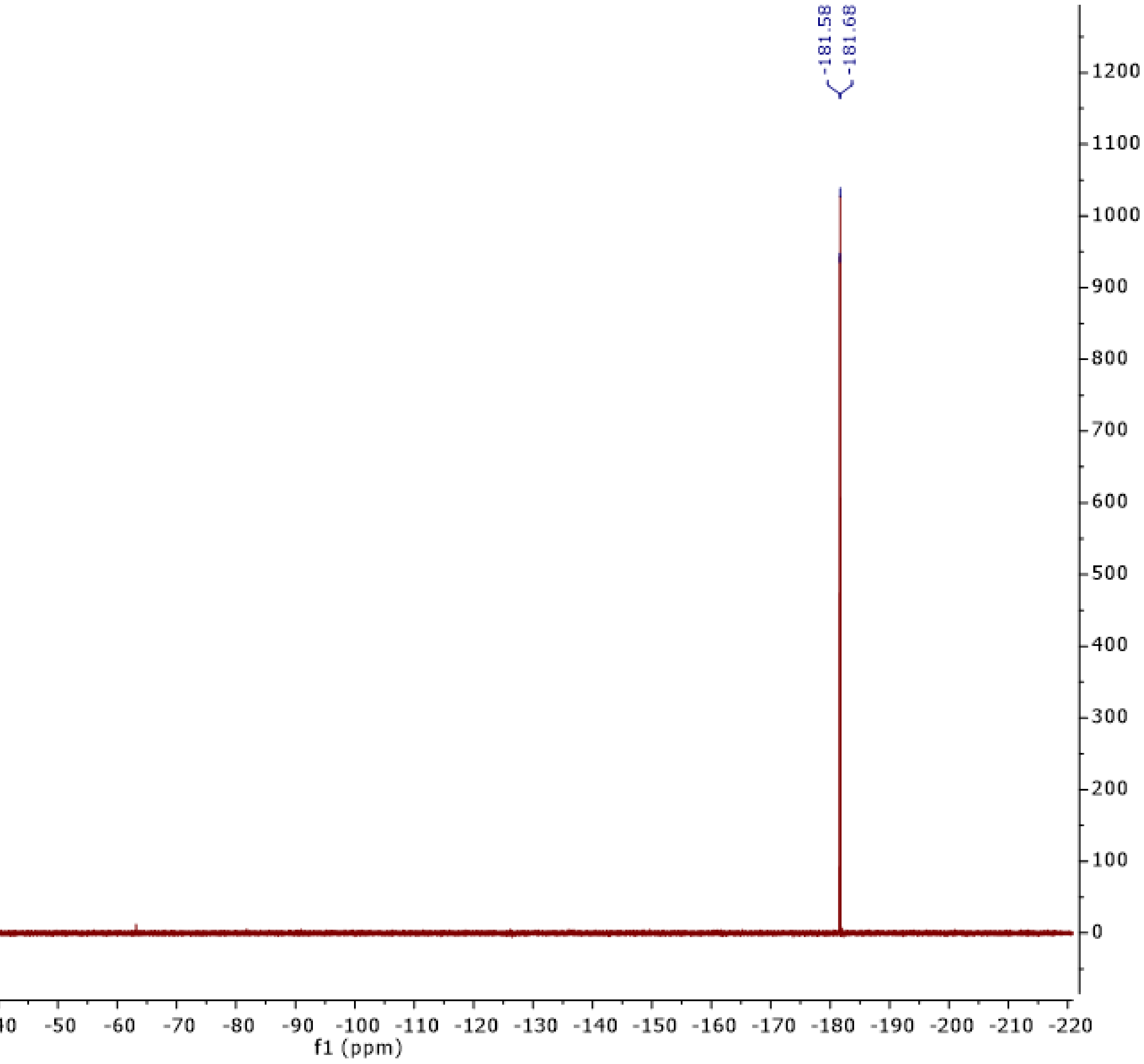

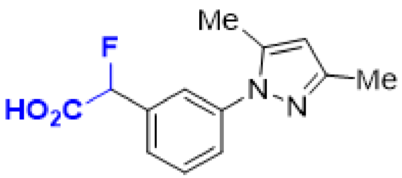


$^1$H NMR (400 MHz)

$d_4$-MeOD

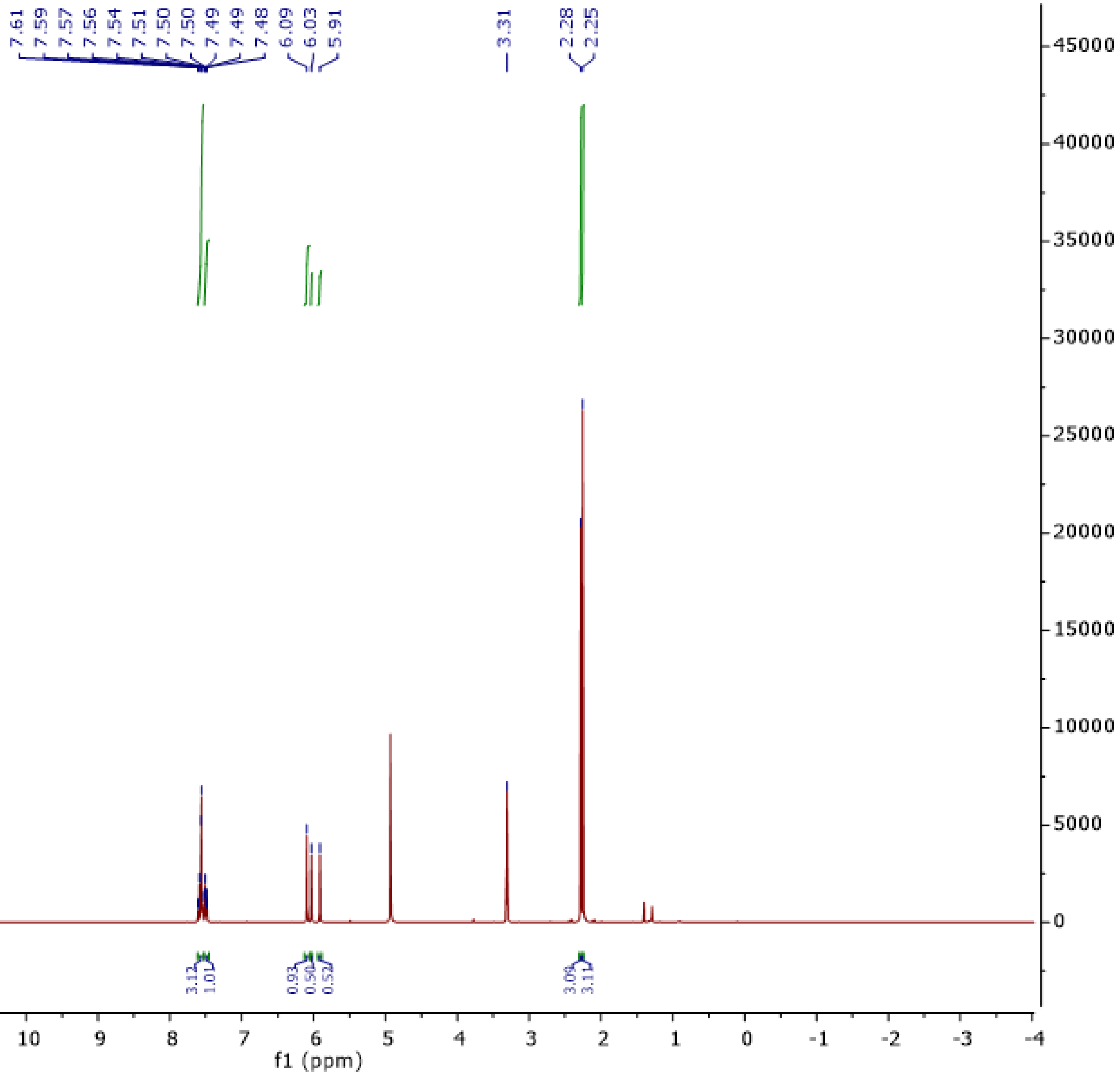

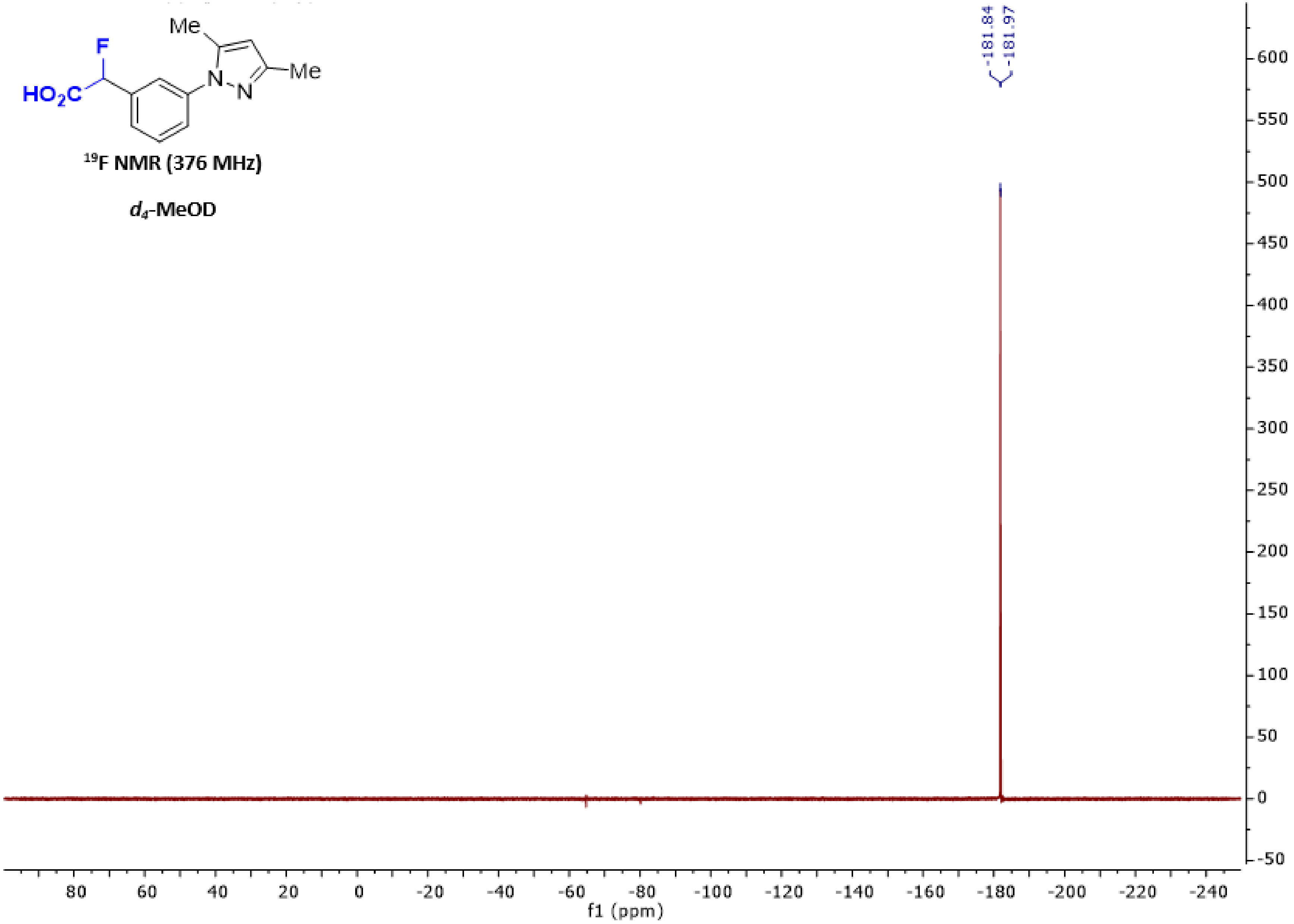


$^{19}$F NMR (376 MHz)

$d_4$-MeOD

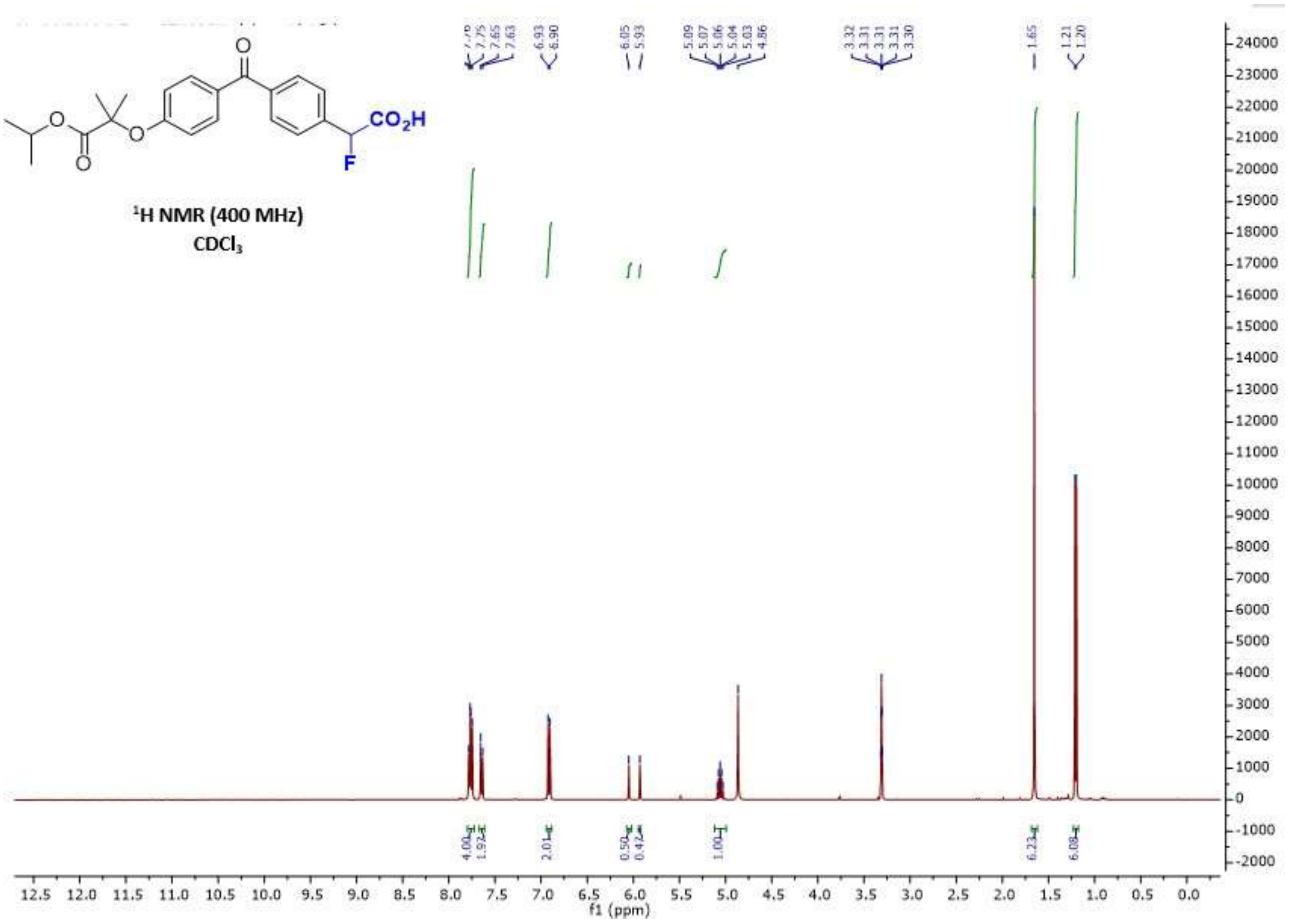
CO2H
F
1H NMR (400 MHz)
CDCl3
7.75
7.65
7.63
6.93
6.90
6.05
5.93
5.09
5.07
5.06
5.04
5.03
4.86
3.32
3.31
3.31
3.31
3.30
1.65
1.21
1.20
4.00
1.92
2.01
0.50
0.42
1.00
6.23
6.08
f1 (ppm)

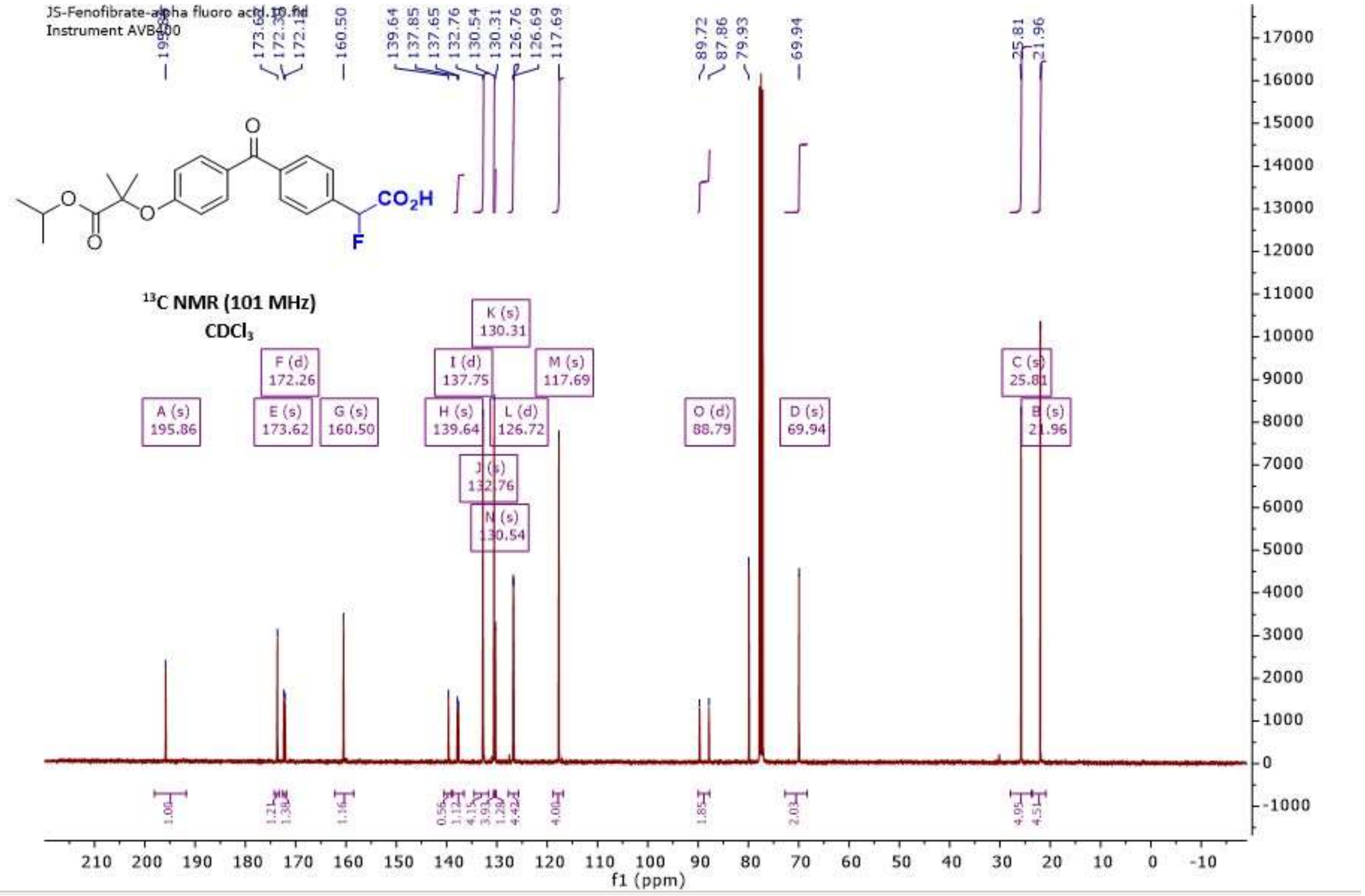

Instrument AVB400
13C NMR (101 MHz)
CDCl3
CO2H
F
173.62
172.30
172.13
160.50
139.64
137.85
137.65
132.76
130.54
130.31
126.76
126.69
117.69
89.72
87.86
79.93
69.94
25.81
21.96
A (s) 195.86
E (s) 173.62
F (d) 172.26
G (s) 160.50
H (s) 139.64
I (d) 137.75
J (s) 132.76
K (s) 130.31
N (s) 130.54
L (d) 126.72
M (s) 117.69
O (d) 88.79
D (s) 69.94
C (s) 25.81
B (s) 21.96
1.00
1.21
1.38
1.16
0.56
1.12
4.15
3.93
1.28
4.42
4.00
1.85
2.03
4.95
4.51
f1 (ppm)

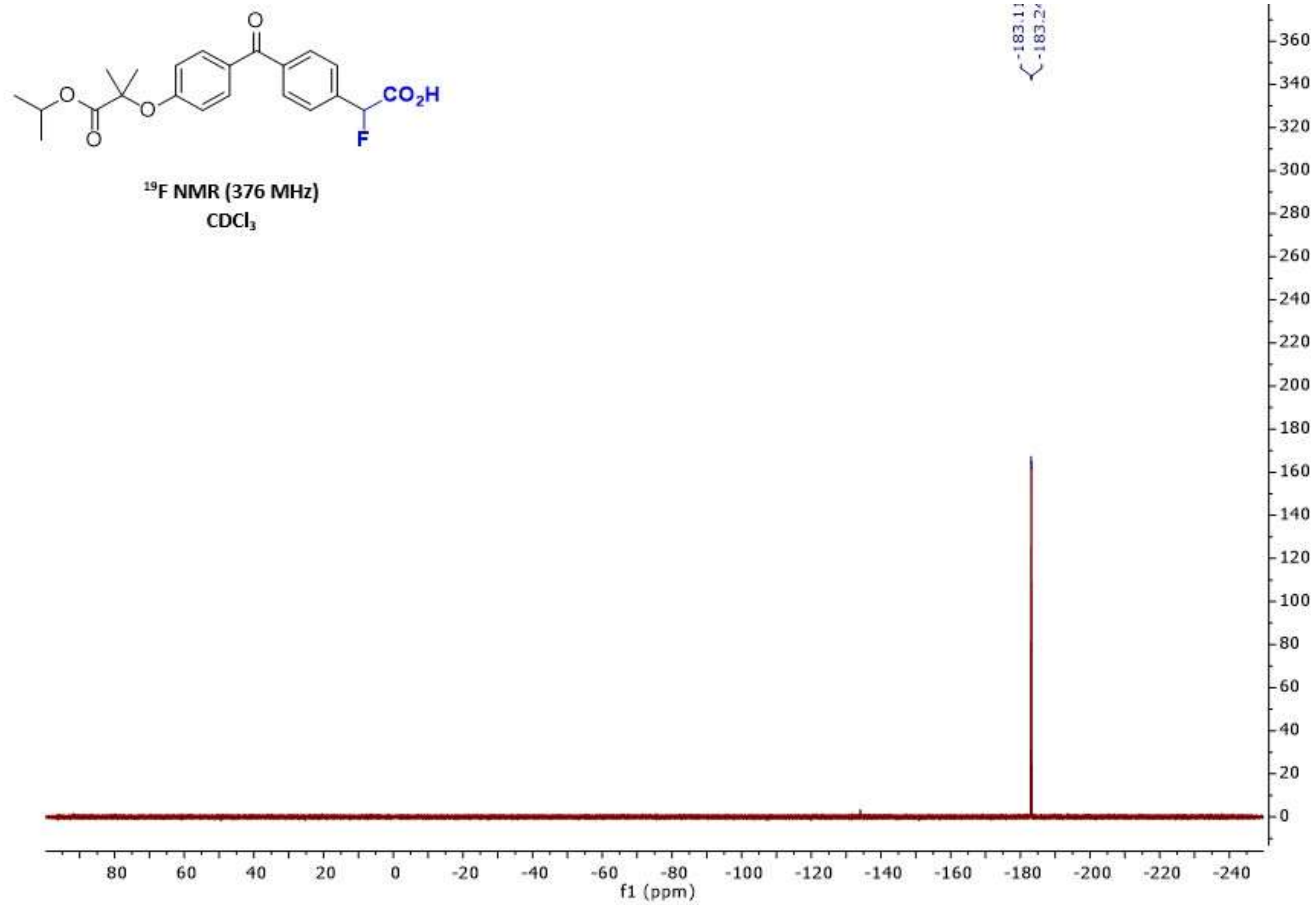
O
O
O
O
CO2H
F
19F NMR (376 MHz)
CDCl3
f1 (ppm)

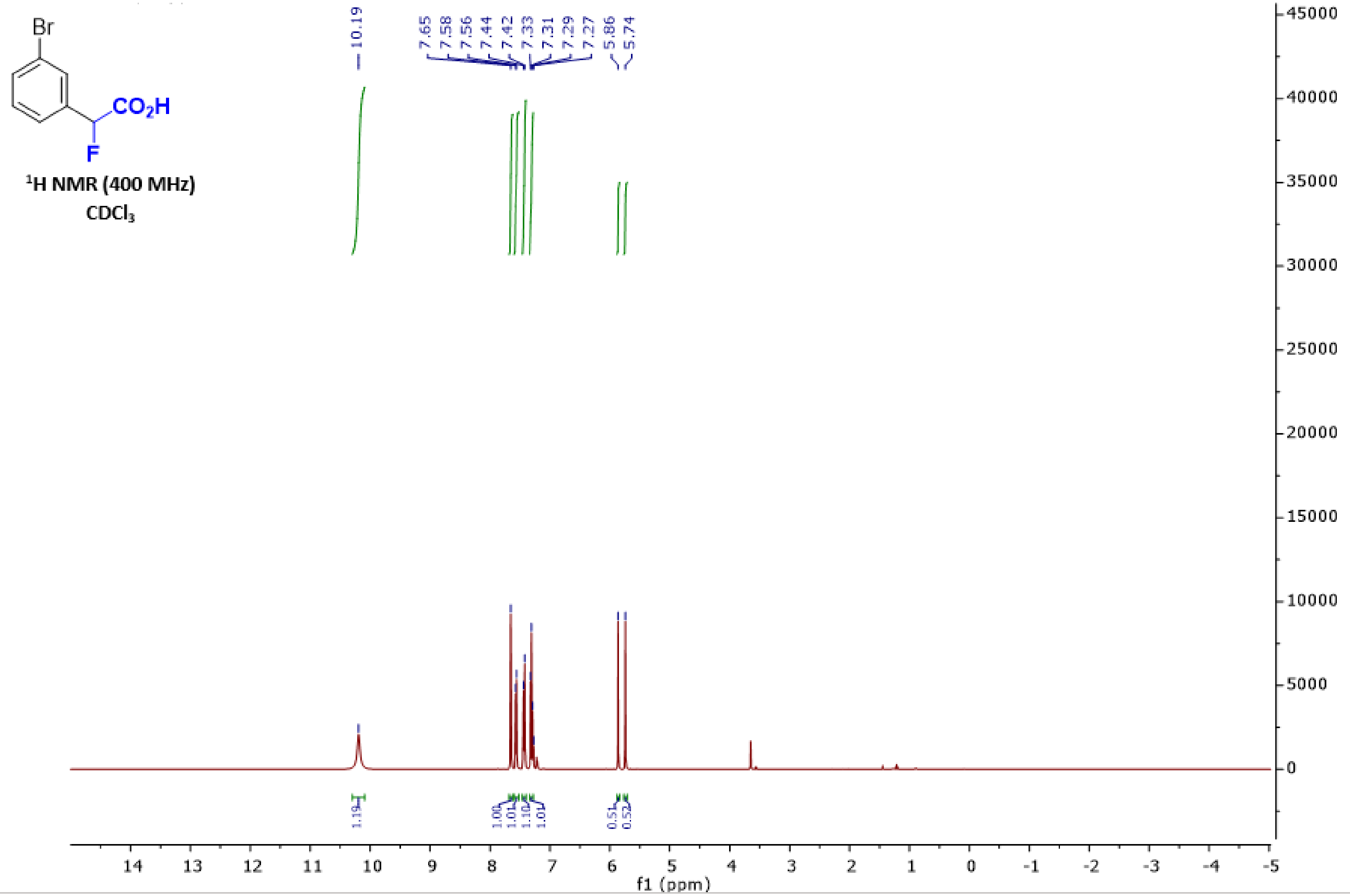
Br
CO2H
F
1H NMR (400 MHz)
CDCl3
10.19
7.65
7.58
7.56
7.44
7.42
7.33
7.31
7.29
7.27
5.86
5.74
1.19
1.00
1.01
1.10
1.01
0.51
0.52
f1 (ppm)

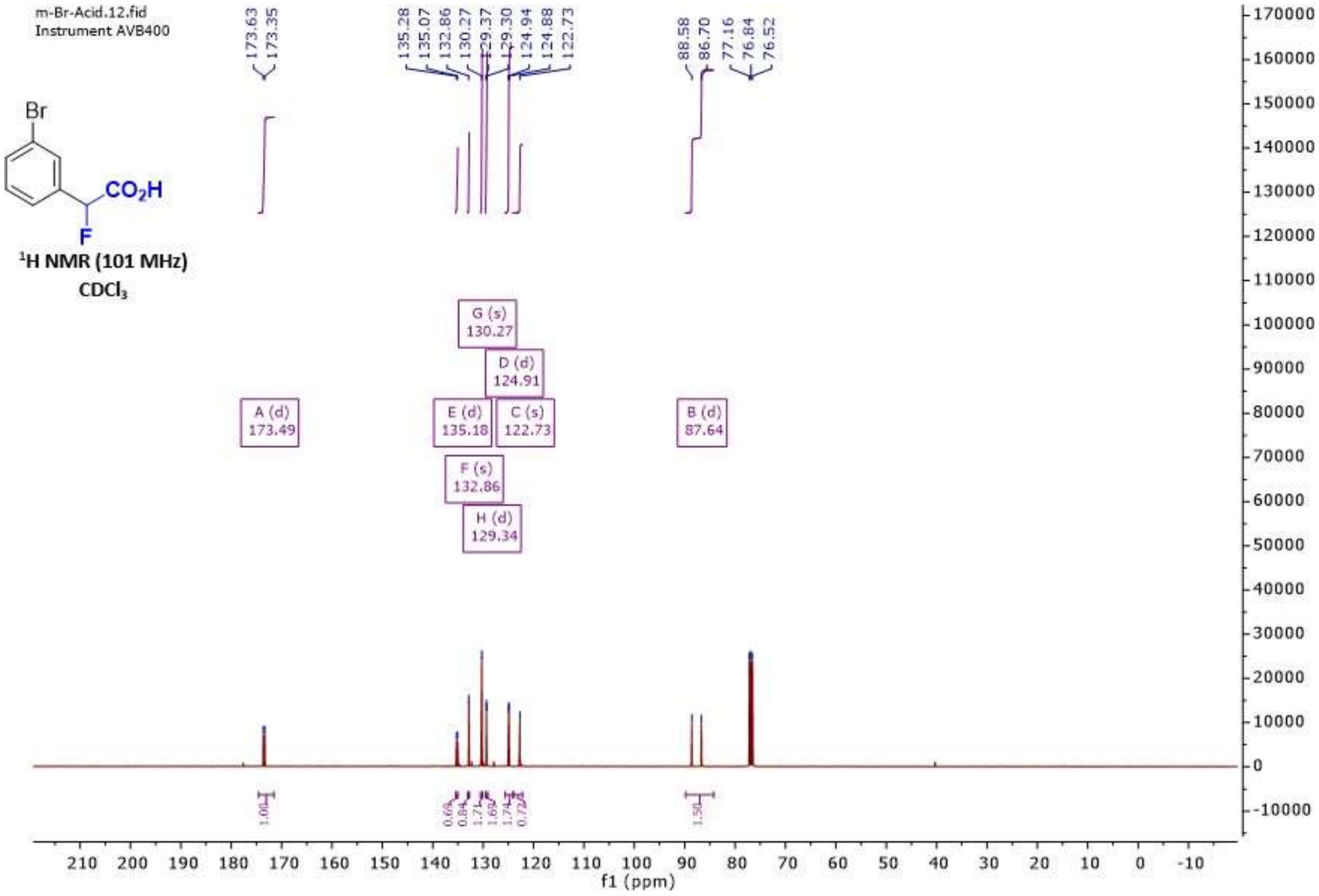

m-Br-Acid.12.fid
Instrument AVB400
173.63
173.35
135.28
135.07
132.86
130.27
129.37
129.30
124.94
124.88
122.73
88.58
86.70
77.16
76.84
76.52
Br
CO2H
F
1H NMR (101 MHz)
CDCl3
G (s) 130.27
D (d) 124.91
A (d) 173.49
E (d) 135.18
C (s) 122.73
B (d) 87.64
F (s) 132.86
H (d) 129.34
1.00
0.69
0.84
1.71
1.69
1.74
0.72
1.50
f1 (ppm)

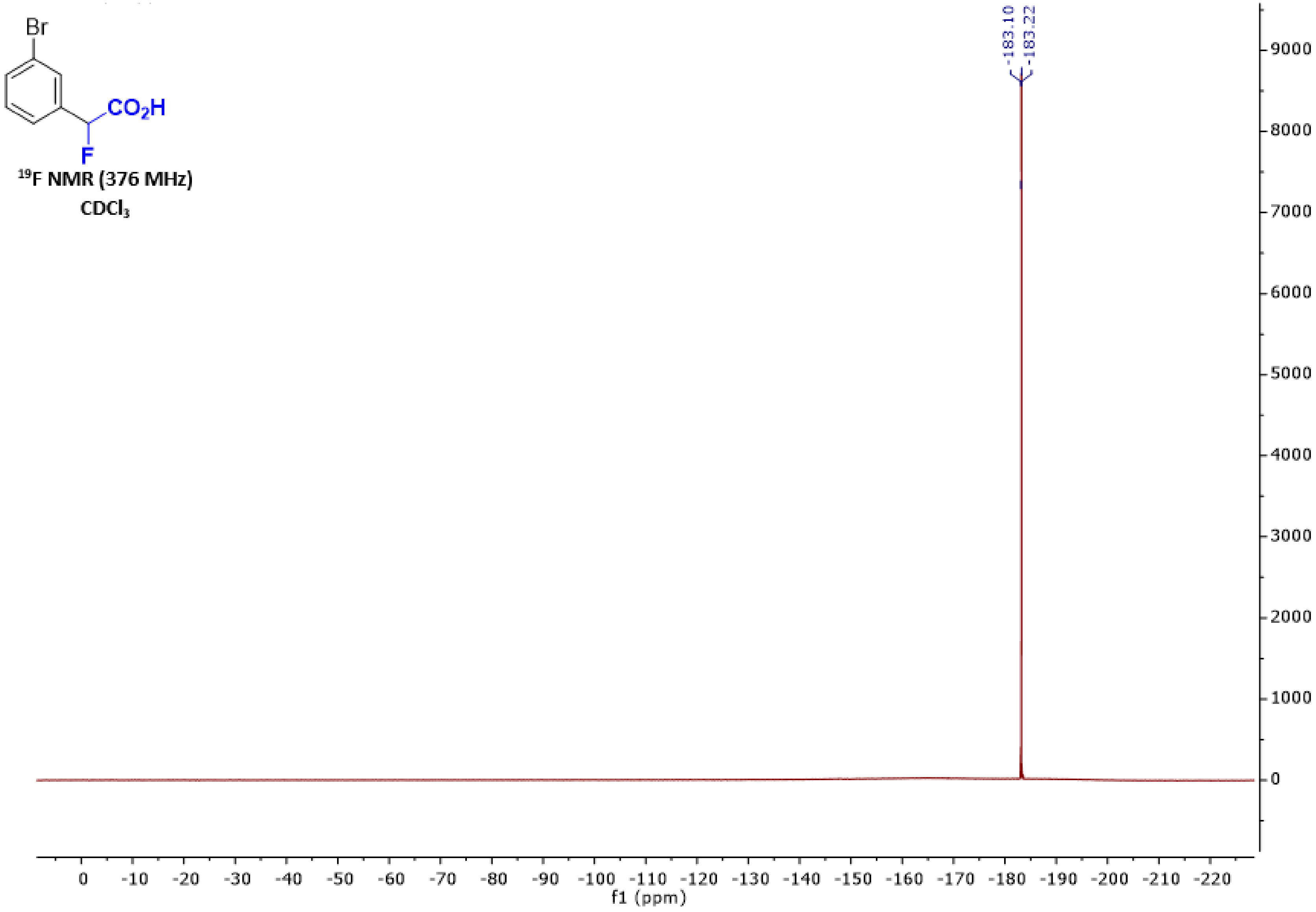
Br
CO2H
F
19F NMR (376 MHz)
CDCl3
-183.10
-183.22
9000
8000
7000
6000
5000
4000
3000
2000
1000
0
0 -10 -20 -30 -40 -50 -60 -70 -80 -90 -100 -110 -120 -130 -140 -150 -160 -170 -180 -190 -200 -210 -220
f1 (ppm)

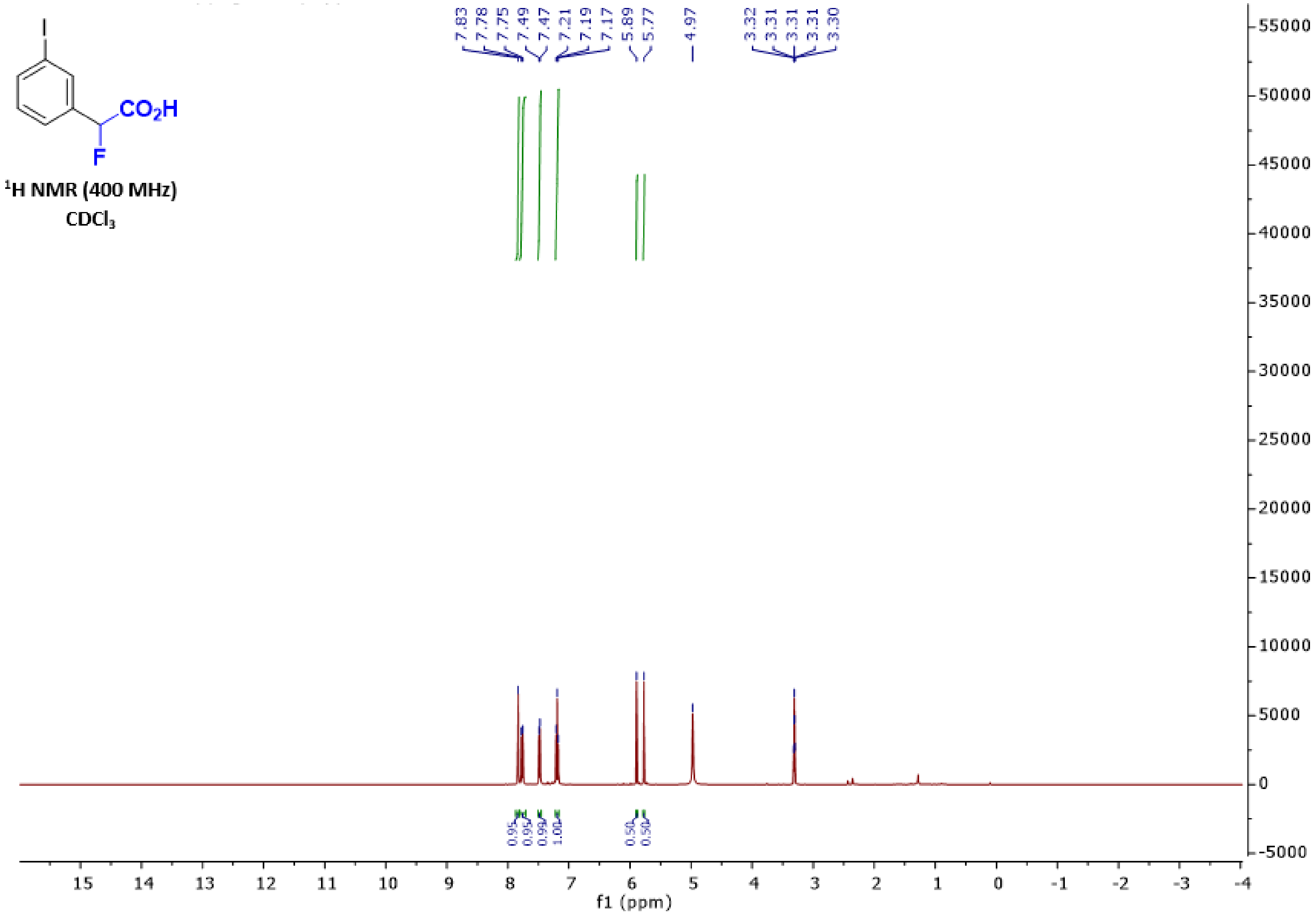
CO2H
F
1H NMR (400 MHz)
CDCl3
7.83
7.78
7.75
7.49
7.47
7.21
7.19
7.17
5.89
5.77
4.97
3.32
3.31
3.31
3.31
3.30
0.95
0.95
0.99
1.00
0.50
0.50
f1 (ppm)

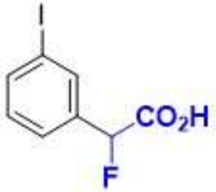


$^{13}$C NMR (101 MHz)
$CDCl_3$

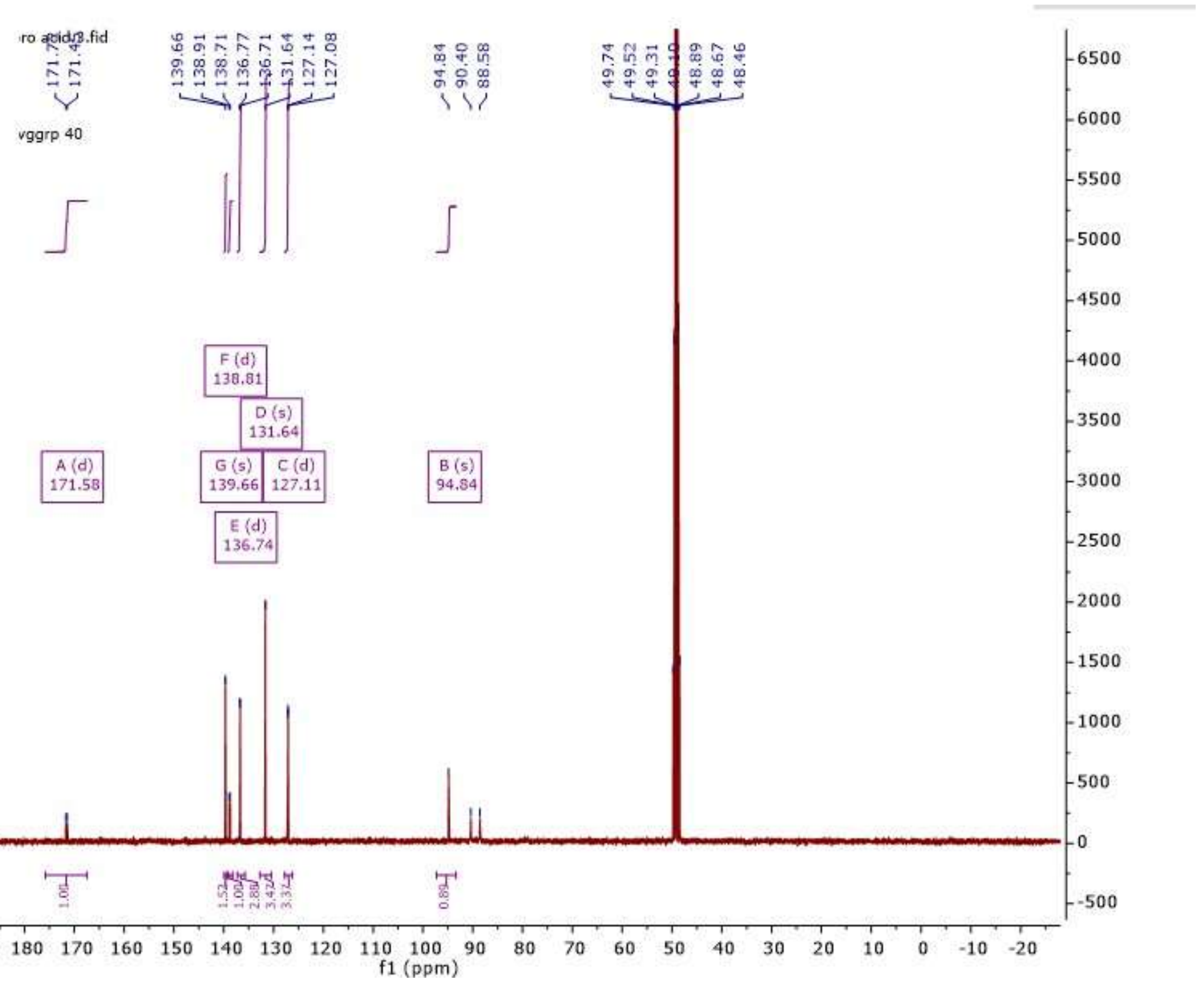

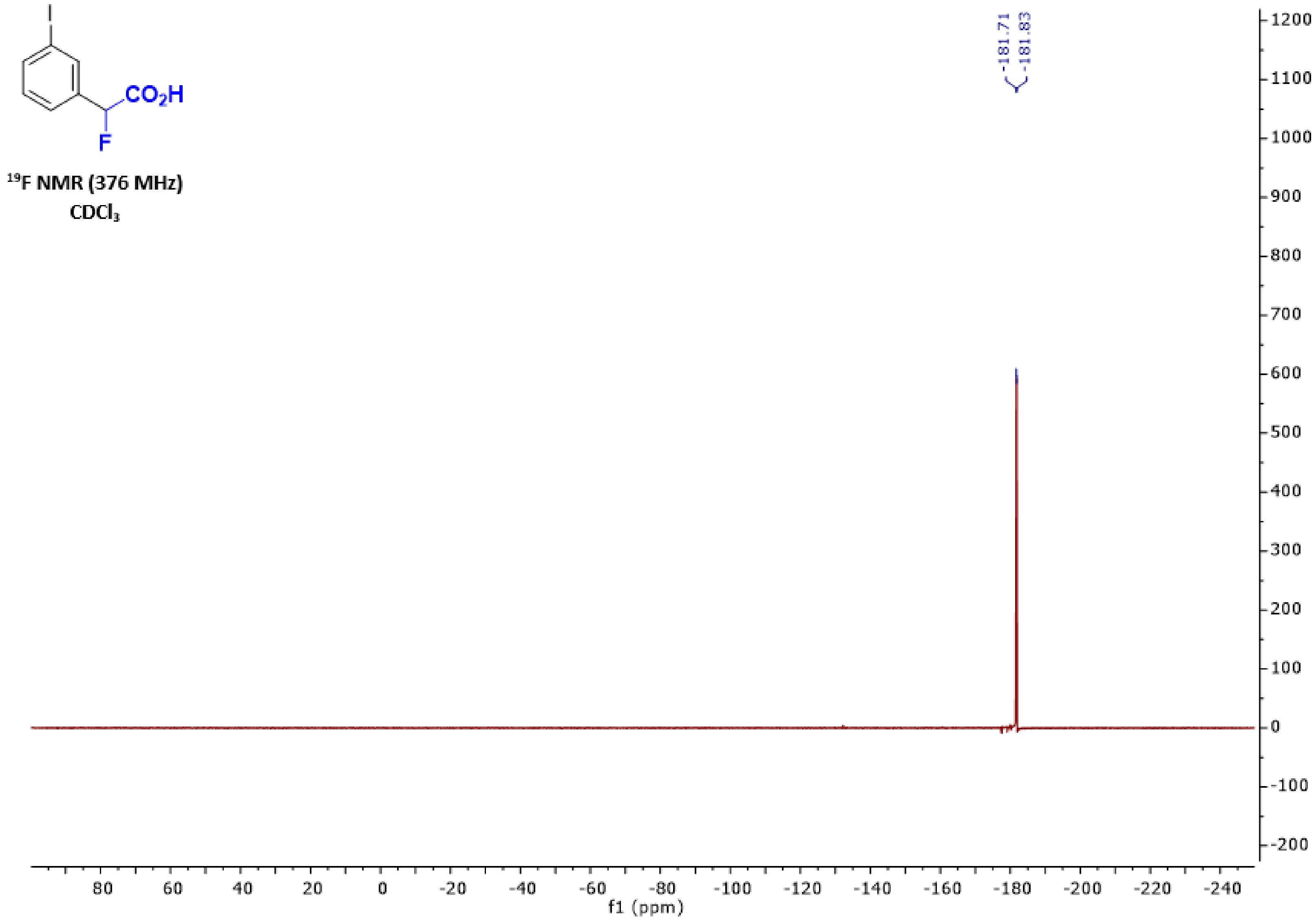

CO2H
F
I
19F NMR (376 MHz)
CDCl3
-181.71
-181.83
f1 (ppm)

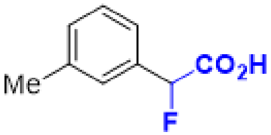


$^{19}$F NMR (400 MHz)

$CDCl_3$

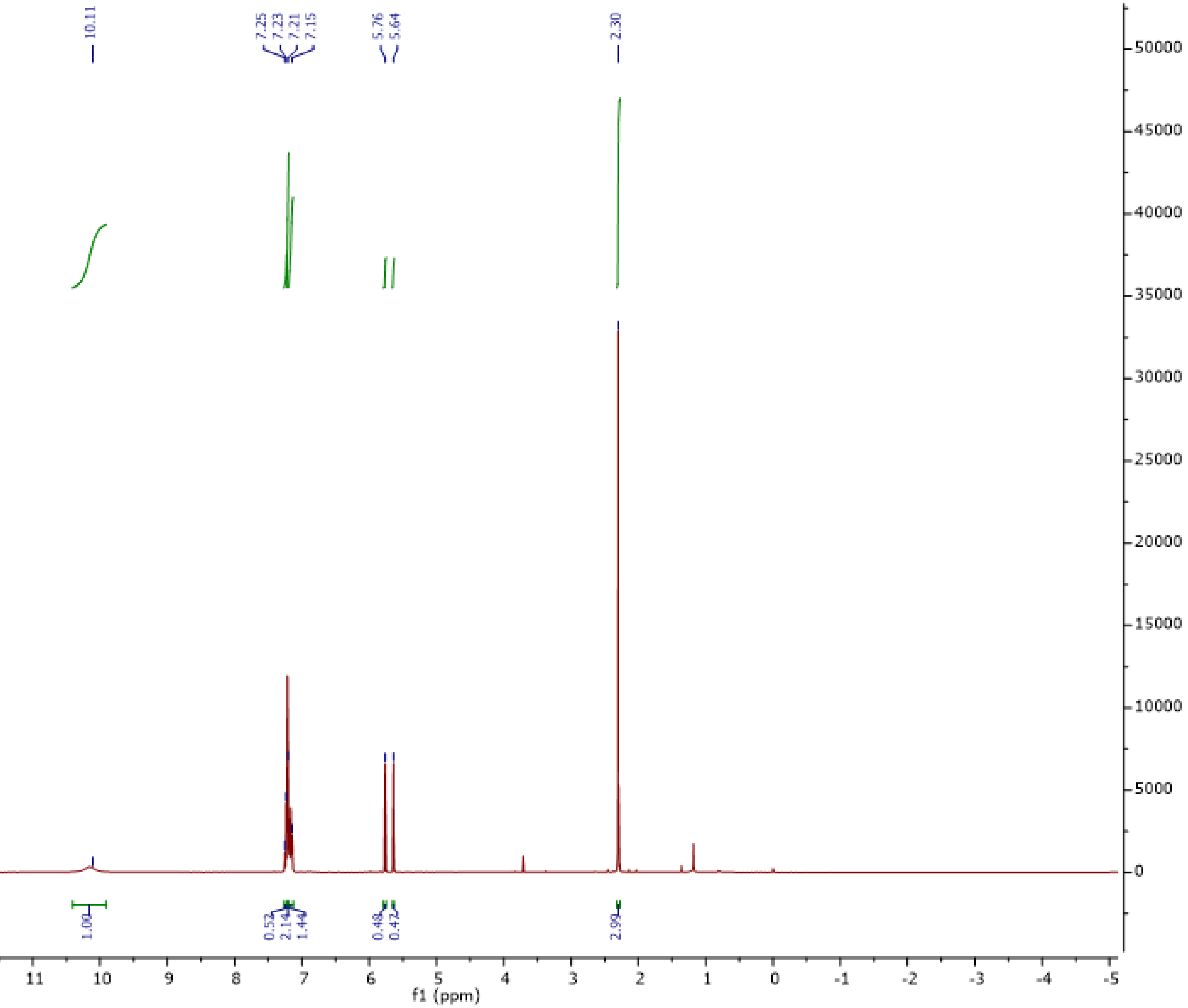

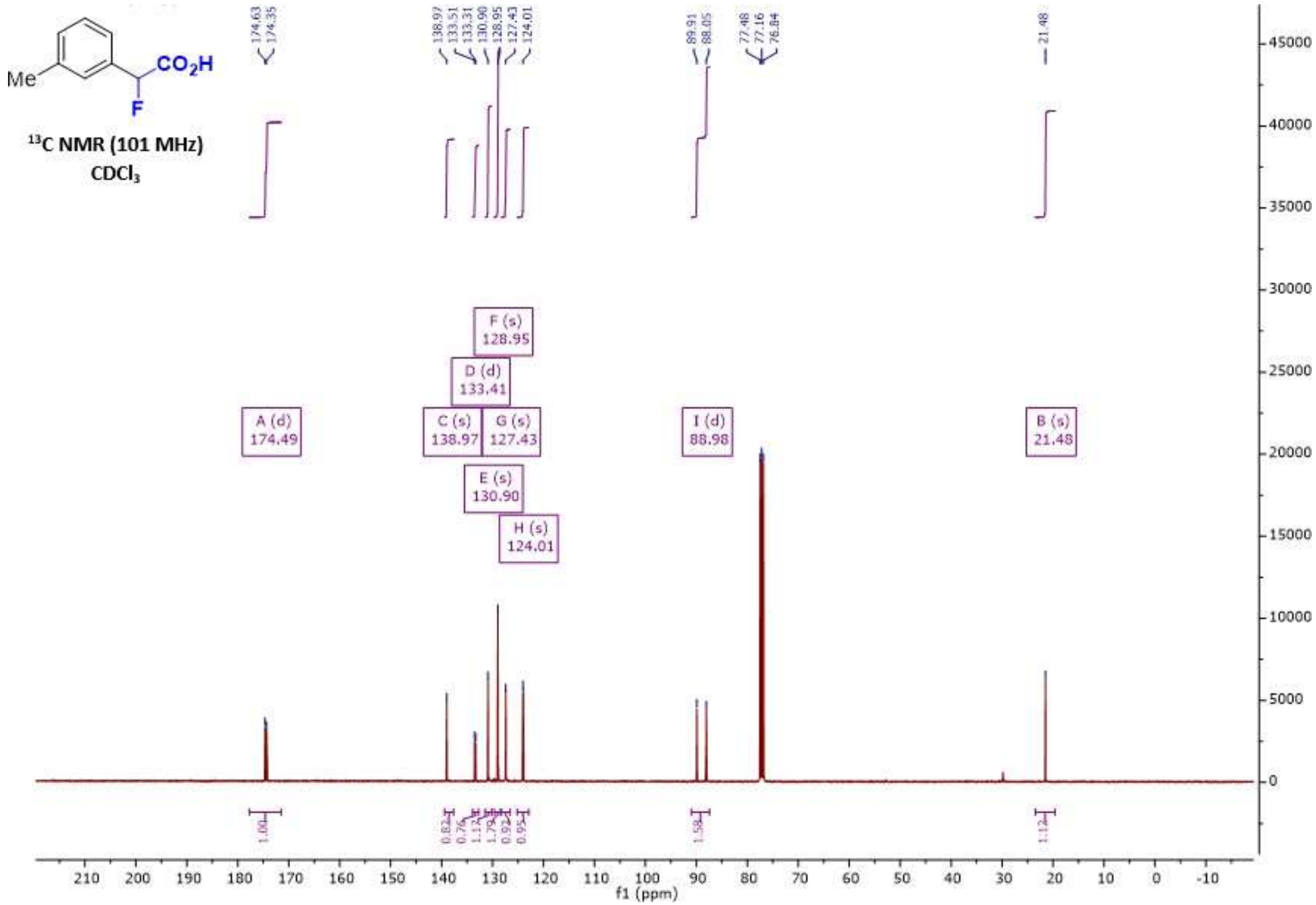

Me
$CO_2H$
F
$^{13}C$ NMR (101 MHz)
$CDCl_3$
174.63
174.35
138.97
133.51
133.31
130.90
128.95
127.43
124.01
89.91
88.05
77.48
77.16
76.84
21.48
F (s) 128.95
D (d) 133.41
A (d) 174.49
C (s) 138.97
G (s) 127.43
I (d) 88.98
B (s) 21.48
E (s) 130.90
H (s) 124.01
1.00
0.82
0.76
1.17
1.79
0.92
0.95
1.58
1.12
45000
40000
35000
30000
25000
20000
15000
10000
5000
0
210
200
190
180
170
160
150
140
130
120
110
100
90
80
70
60
50
40
30
20
10
0
-10
f1 (ppm)

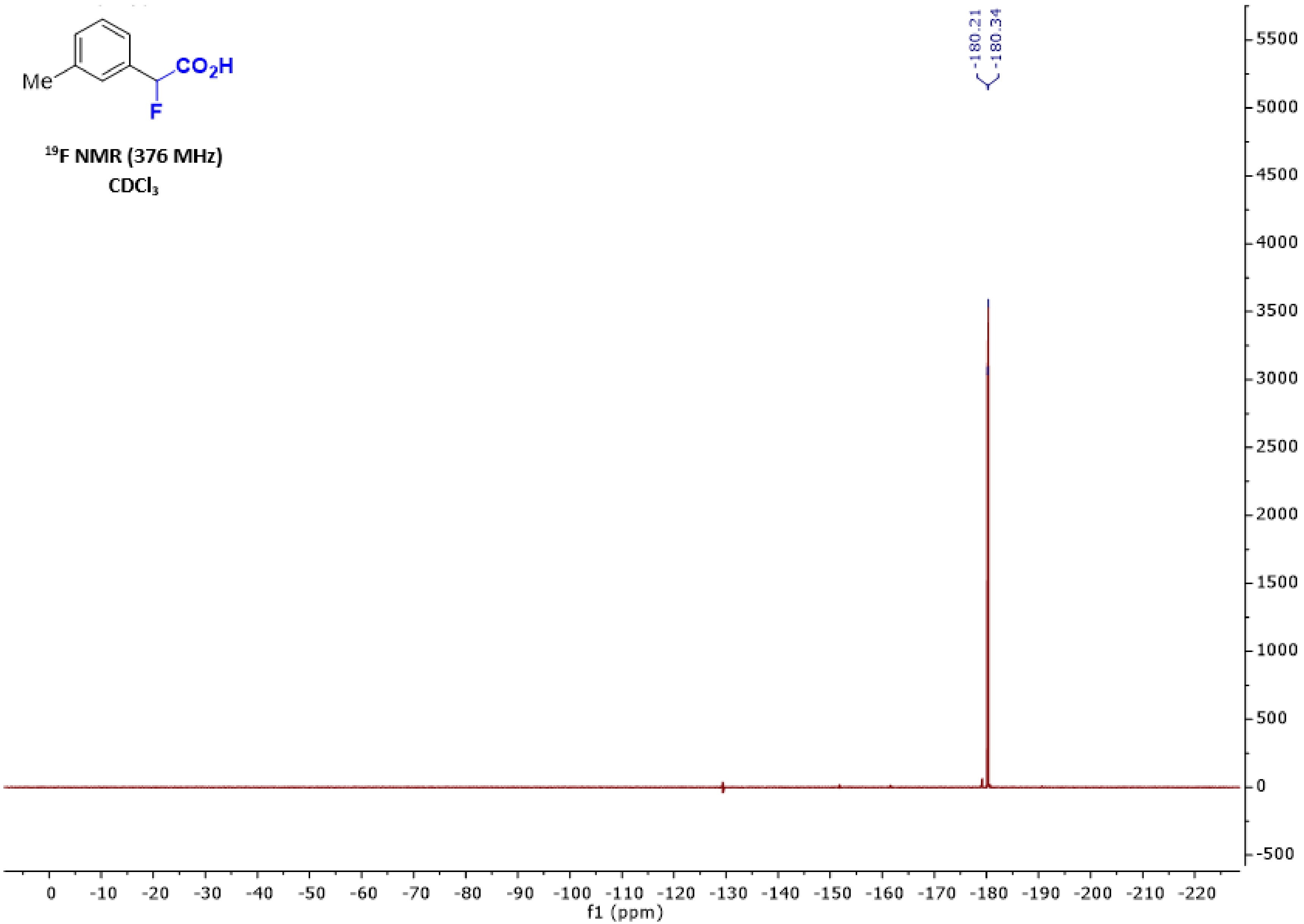
Me
$CO_2H$
F
$^{19}$F NMR (376 MHz)
$CDCl_3$
-180.21
-180.34
5500
5000
4500
4000
3500
3000
2500
2000
1500
1000
500
0
-500
0
-10
-20
-30
-40
-50
-60
-70
-80
-90
-100
-110
-120
-130
-140
-150
-160
-170
-180
-190
-200
-210
-220
f1 (ppm)

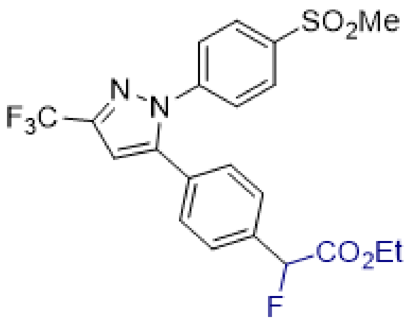


**$^{1}$H NMR (400 MHz)**
**$CDCl_3$**

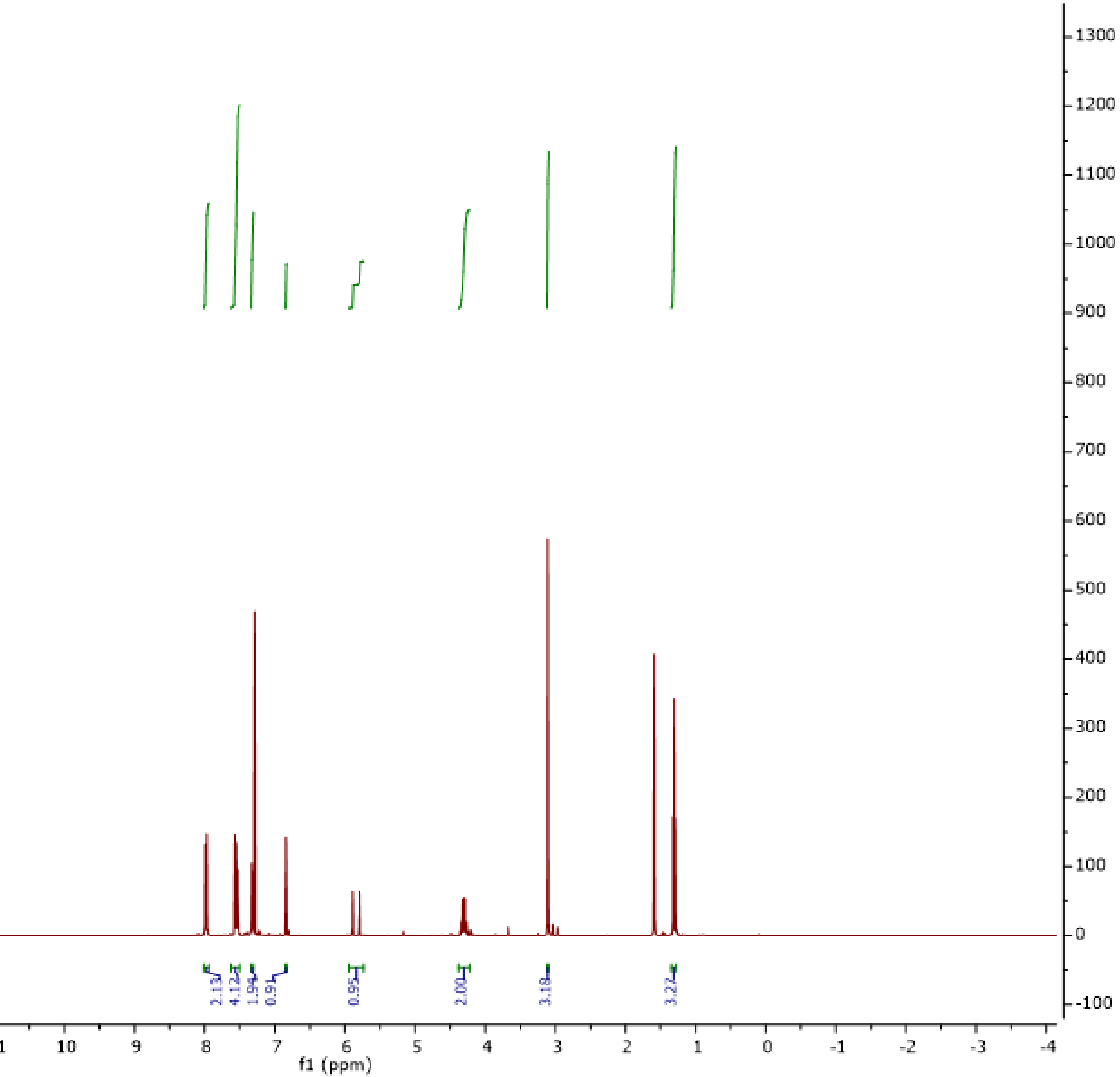

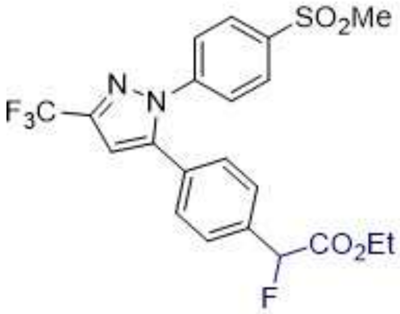

$^{13}$C NMR (101 MHz)
$CDCl_3$

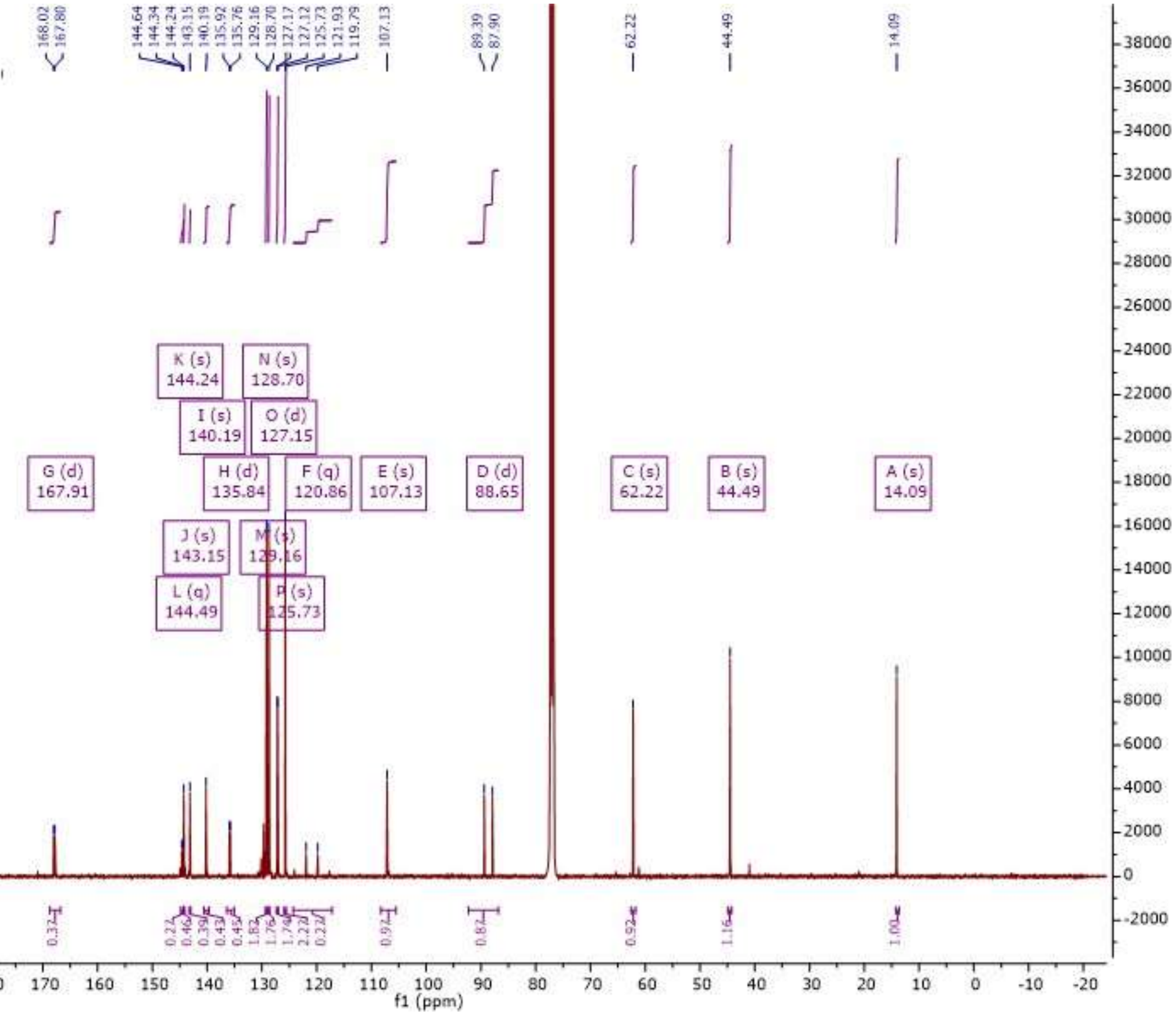

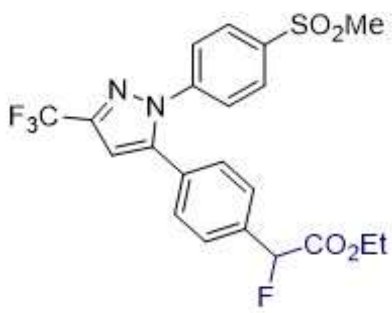


**$^{19}$F NMR (376 MHz)**
**CDCl₃**

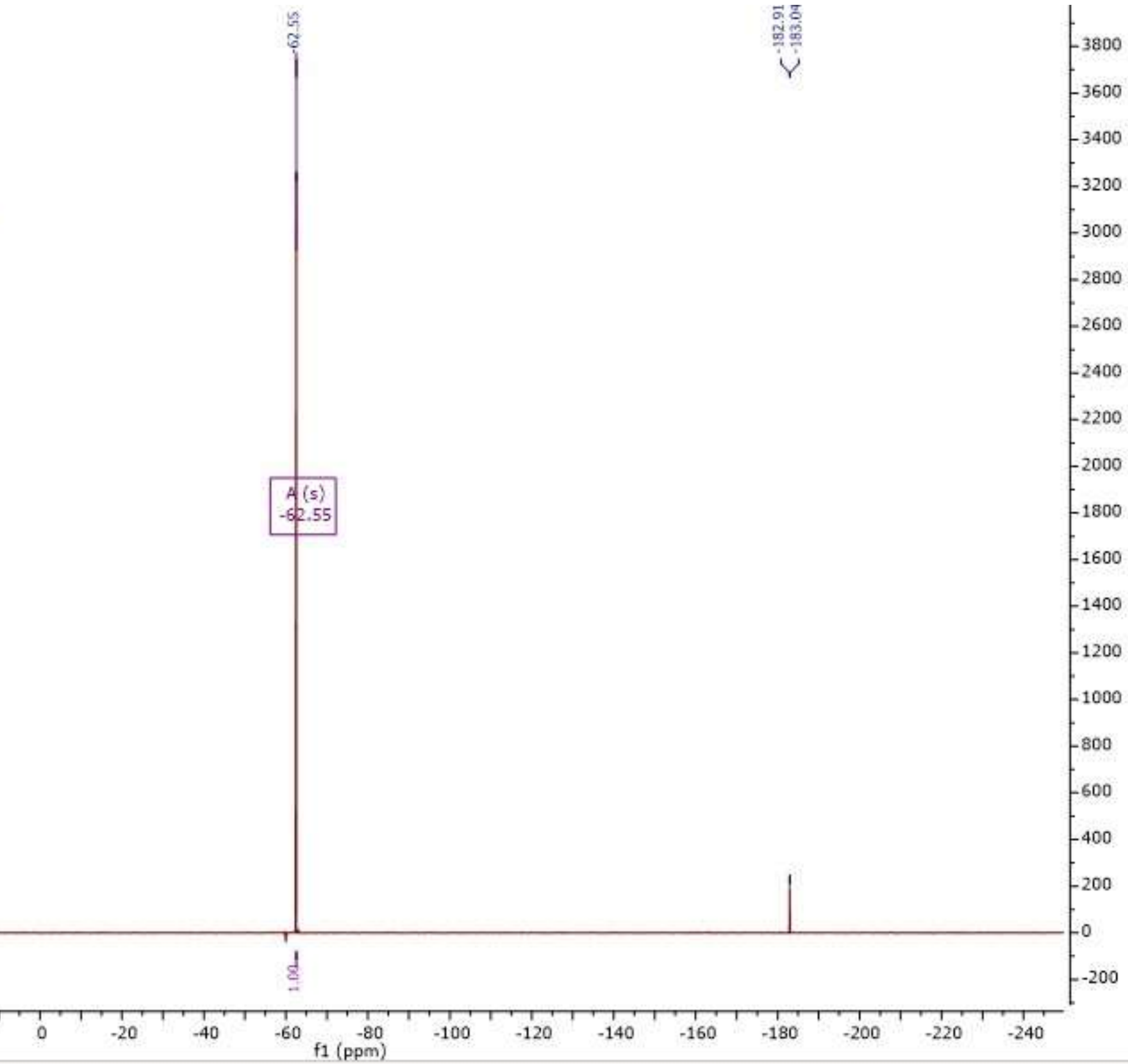

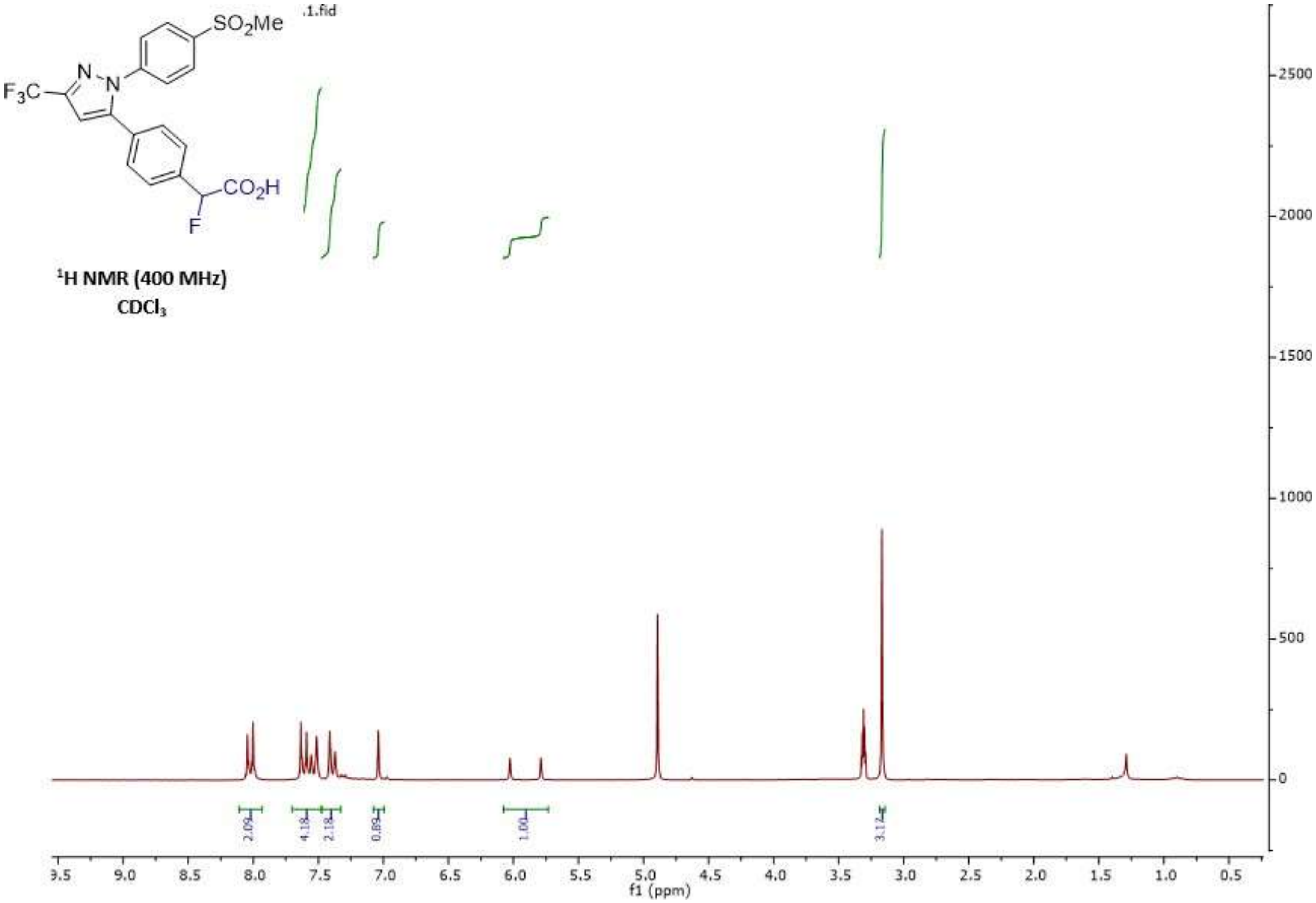

SO2Me
F3C
N
N
CO2H
F
.1.fid
¹H NMR (400 MHz)
CDCl3
2.09
4.18
2.18
0.89
1.00
3.17
f1 (ppm)

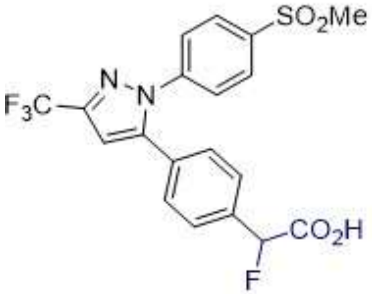


**$^{13}$C NMR (101 MHz)**
**$CDCl_3$**

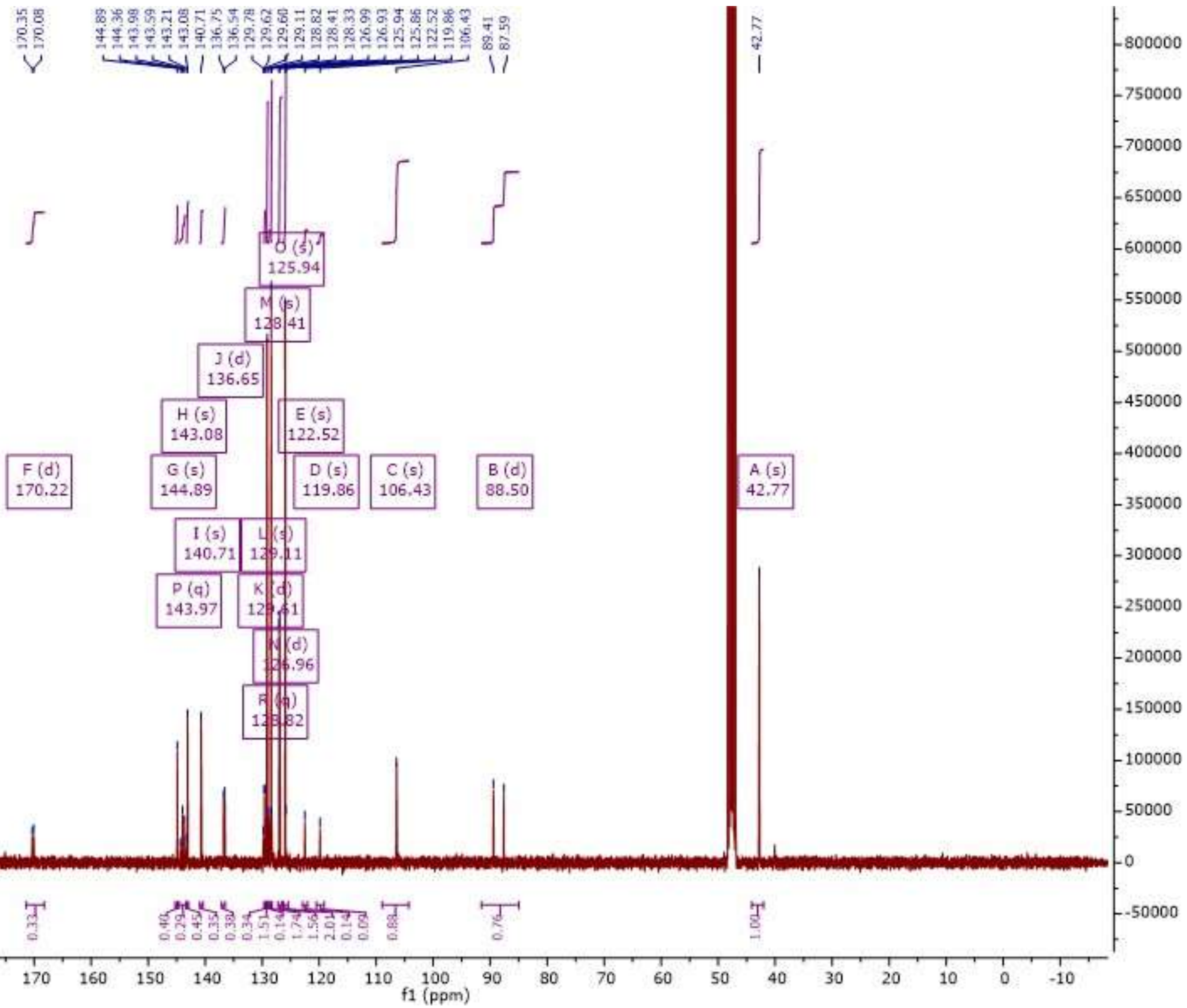

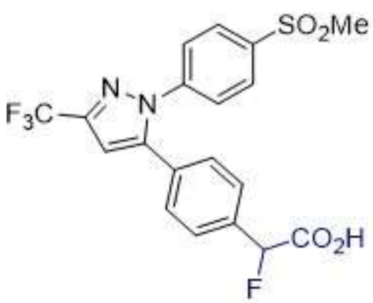

$^{19}F$ NMR (376 MHz)
$CDCl_3$

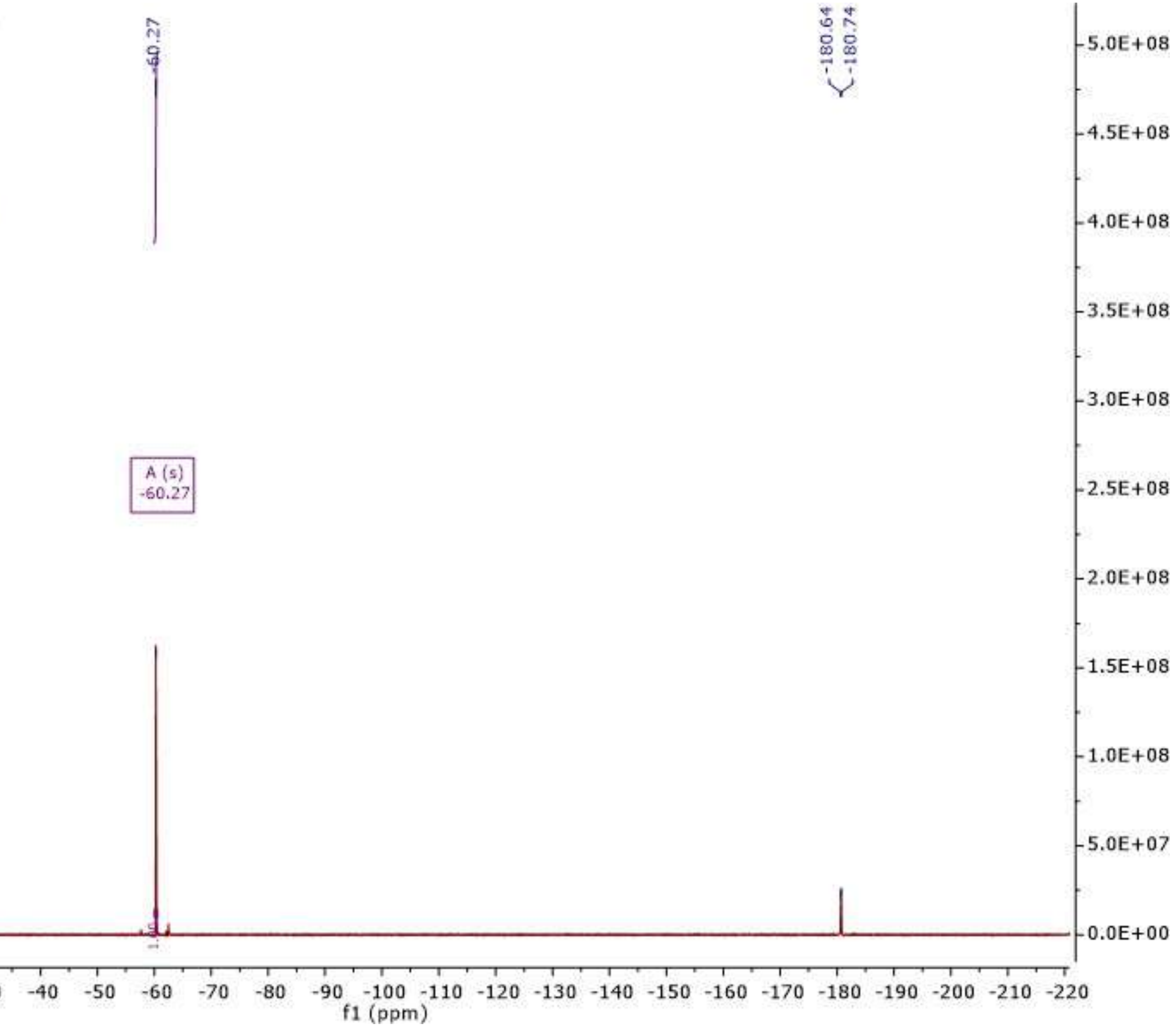

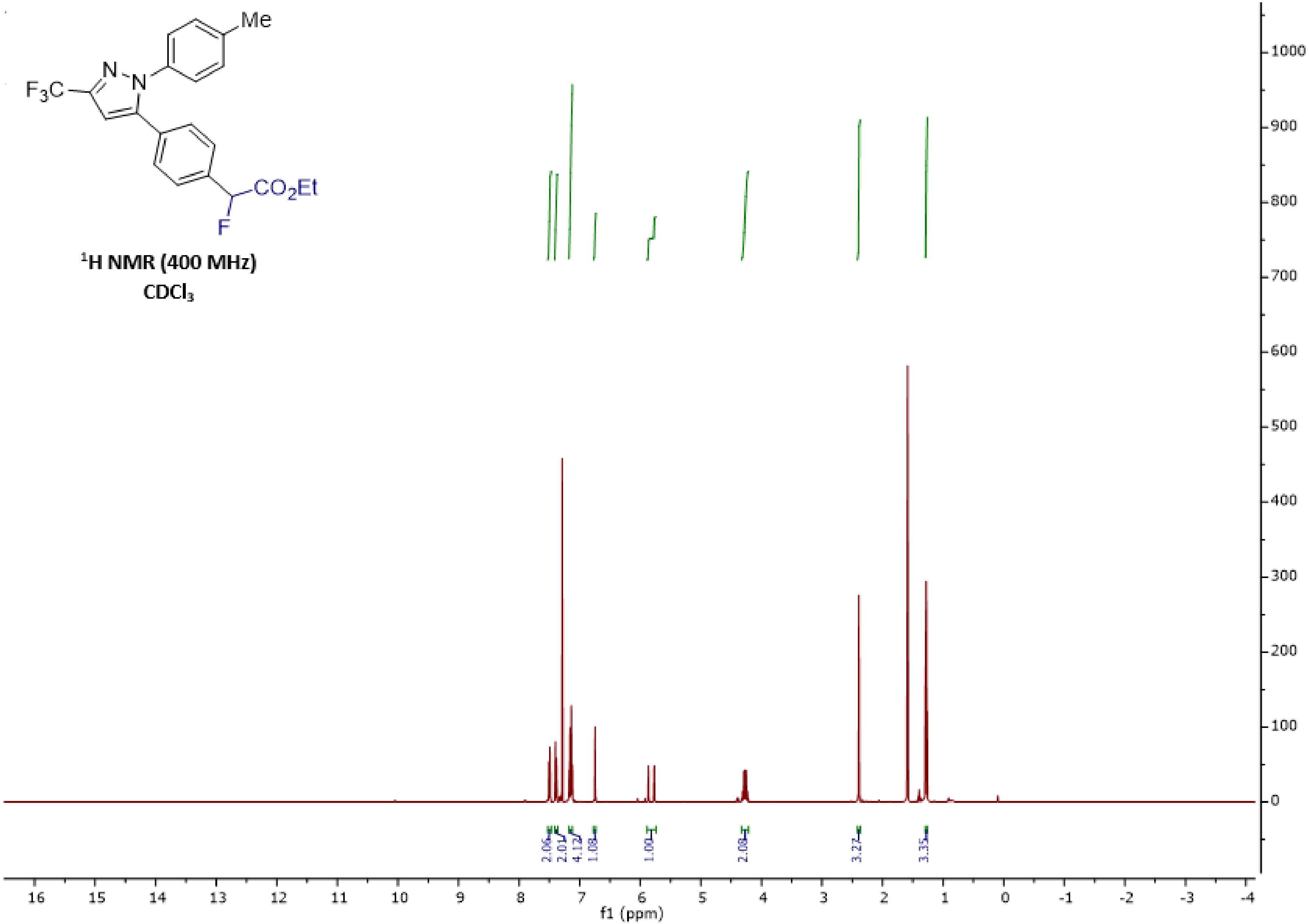
Me
N
N
F3C
CO2Et
F
1H NMR (400 MHz)
CDCl3
2.06
2.01
4.12
1.08
1.00
2.08
3.27
3.35
f1 (ppm)

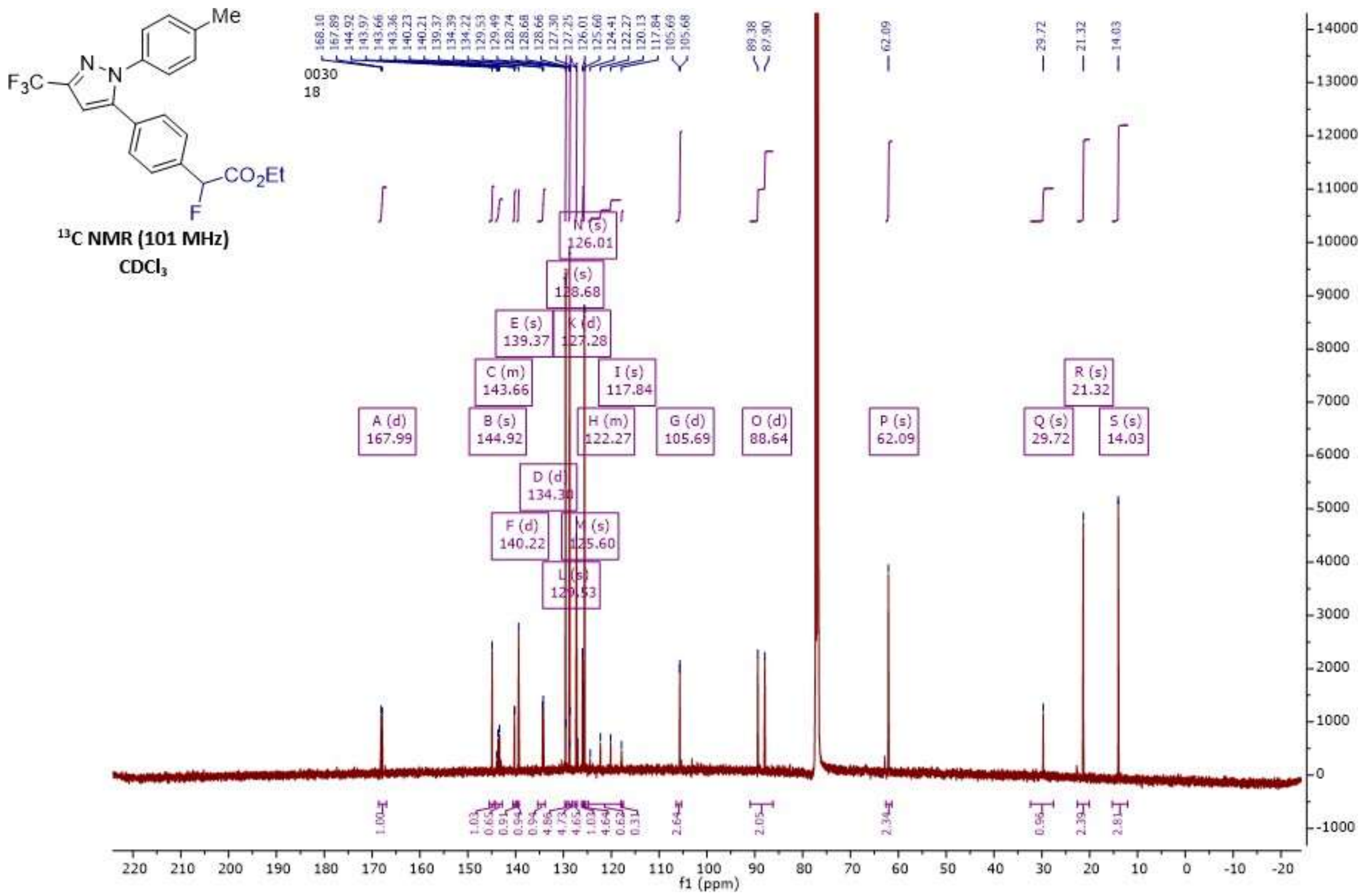


**$^{13}$C NMR (101 MHz)**
**$CDCl_3$**

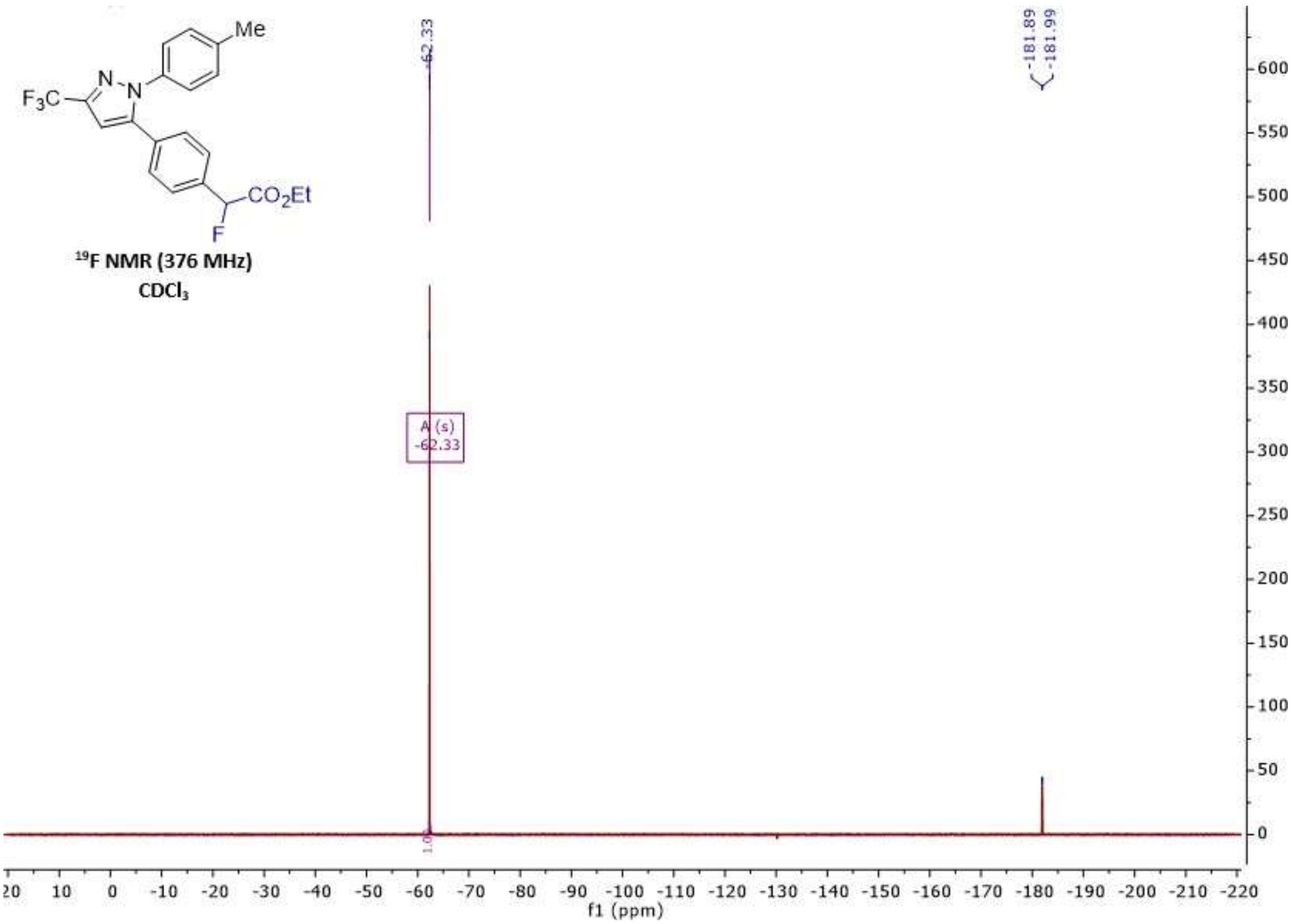

Me
N
N
$F_3C$
$CO_2Et$
F
$^{19}$F NMR (376 MHz)
$CDCl_3$
-62.33
-181.89
-181.99
A (s)
-62.33
1.00
f1 (ppm)

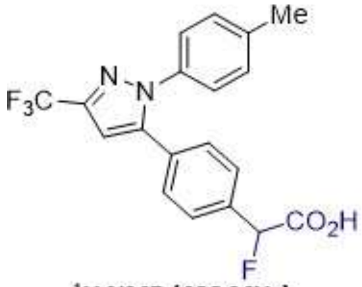

$^1$H NMR (400 MHz)
MeOD

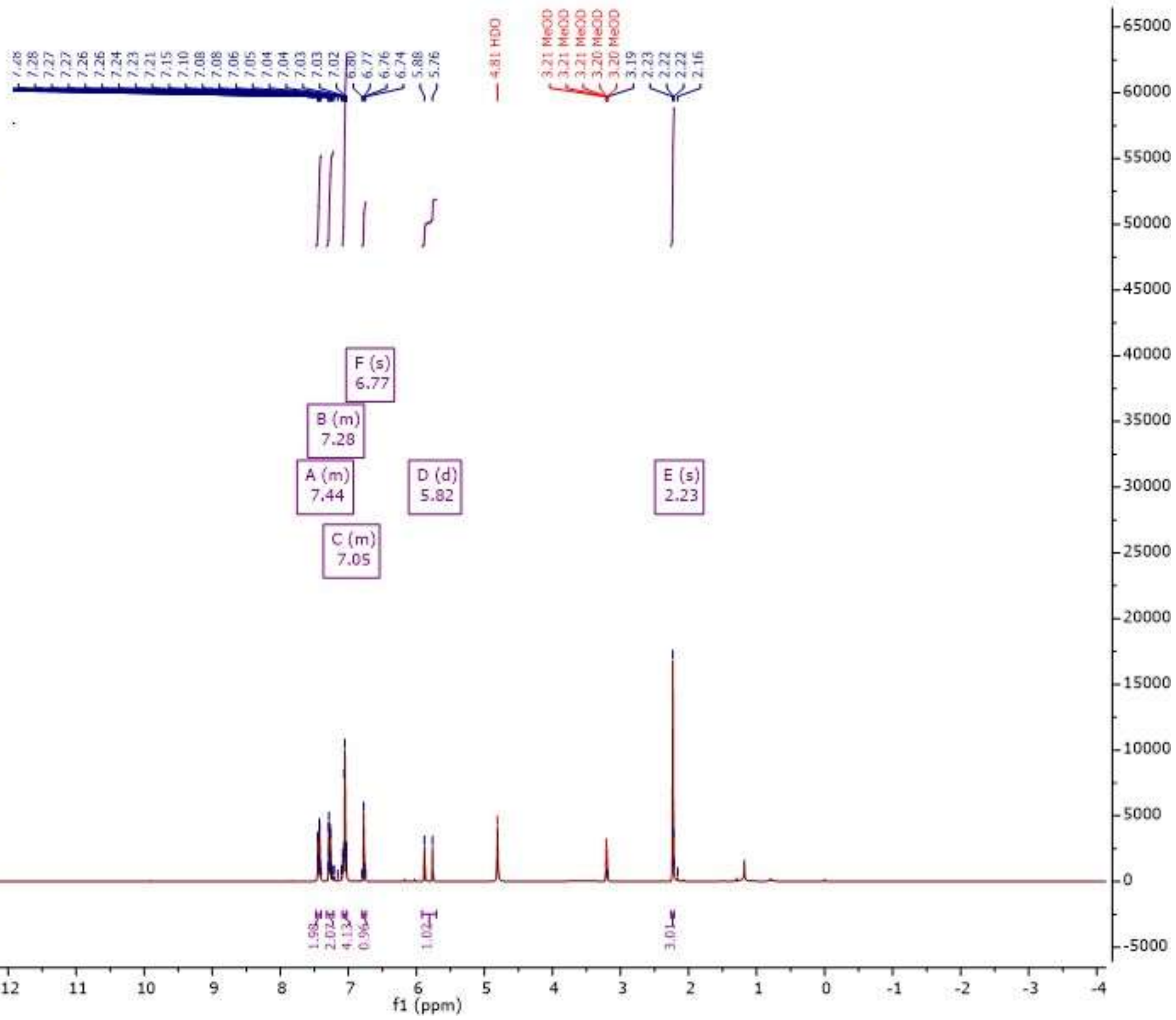

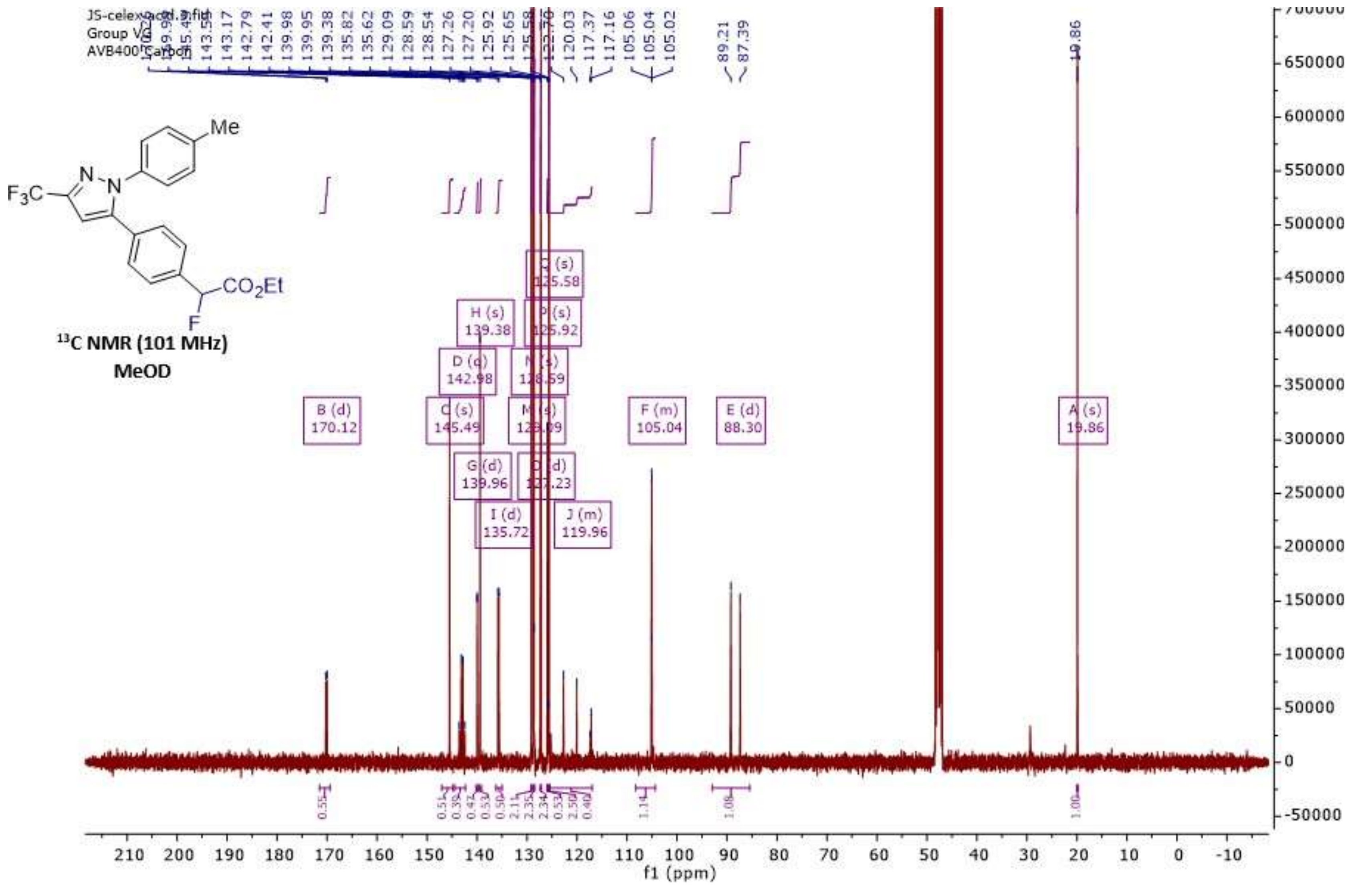

$^{13}$C NMR (101 MHz)
MeOD

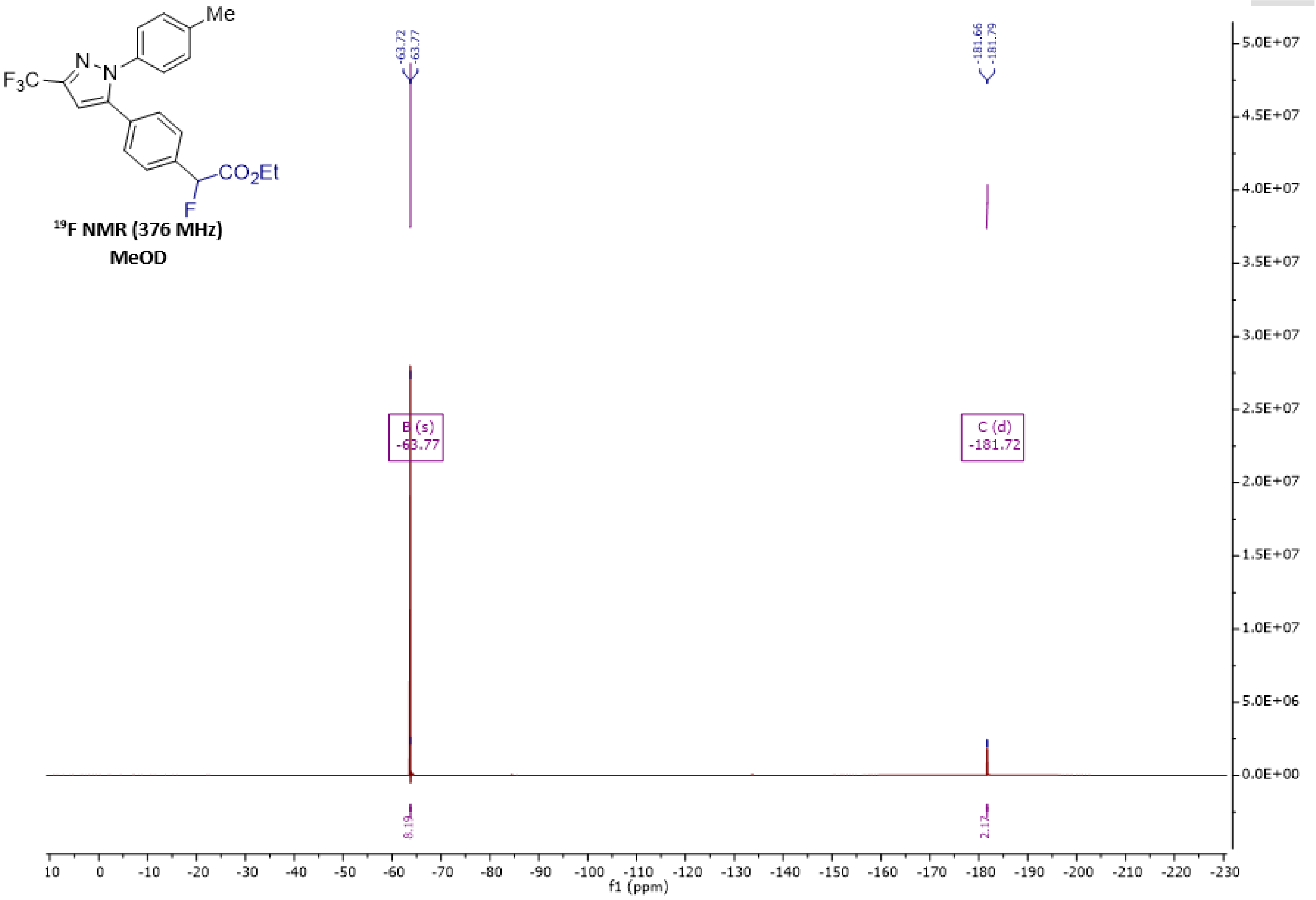

Me
N
N
F3C
CO2Et
F
19F NMR (376 MHz)
MeOD
-63.72
-63.77
-181.66
-181.79
B (s)
-63.77
C (d)
-181.72
8.19
2.17
f1 (ppm)

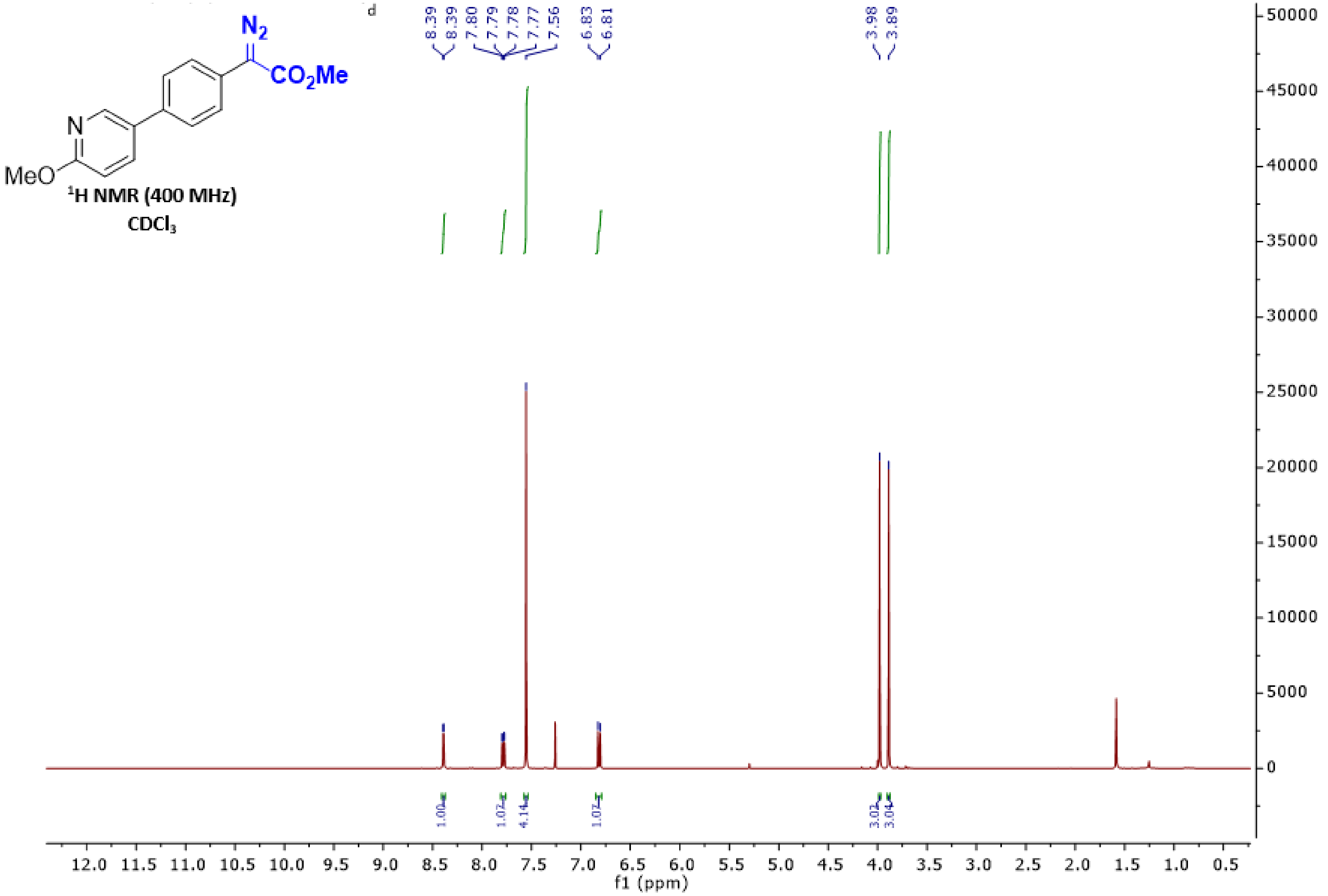

N2
CO2Me
N
MeO
1H NMR (400 MHz)
CDCl3
8.39
8.39
7.80
7.79
7.78
7.77
7.56
6.83
6.81
3.98
3.89
1.00
1.07
4.14
1.07
3.02
3.04
f1 (ppm)

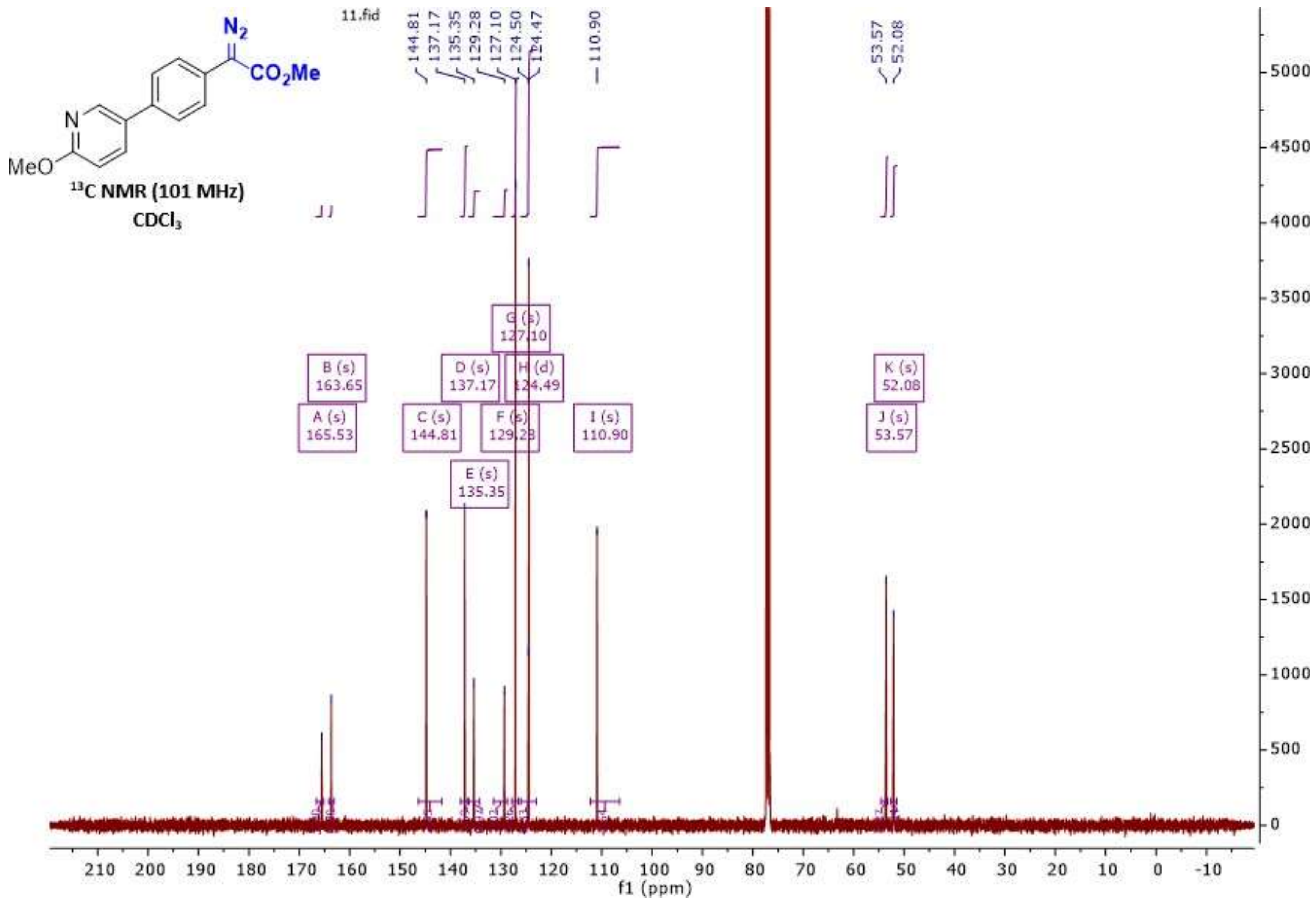
N2
CO2Me
N
MeO
13C NMR (101 MHz)
CDCl3
11.fid
144.81
137.17
135.35
129.28
127.10
124.50
124.47
110.90
53.57
52.08
G (s) 127.10
B (s) 163.65
D (s) 137.17
H (d) 124.49
K (s) 52.08
A (s) 165.53
C (s) 144.81
F (s) 129.28
I (s) 110.90
J (s) 53.57
E (s) 135.35
5000
4500
4000
3500
3000
2500
2000
1500
1000
500
0
210 200 190 180 170 160 150 140 130 120 110 100 90 80 70 60 50 40 30 20 10 0 -10
f1 (ppm)

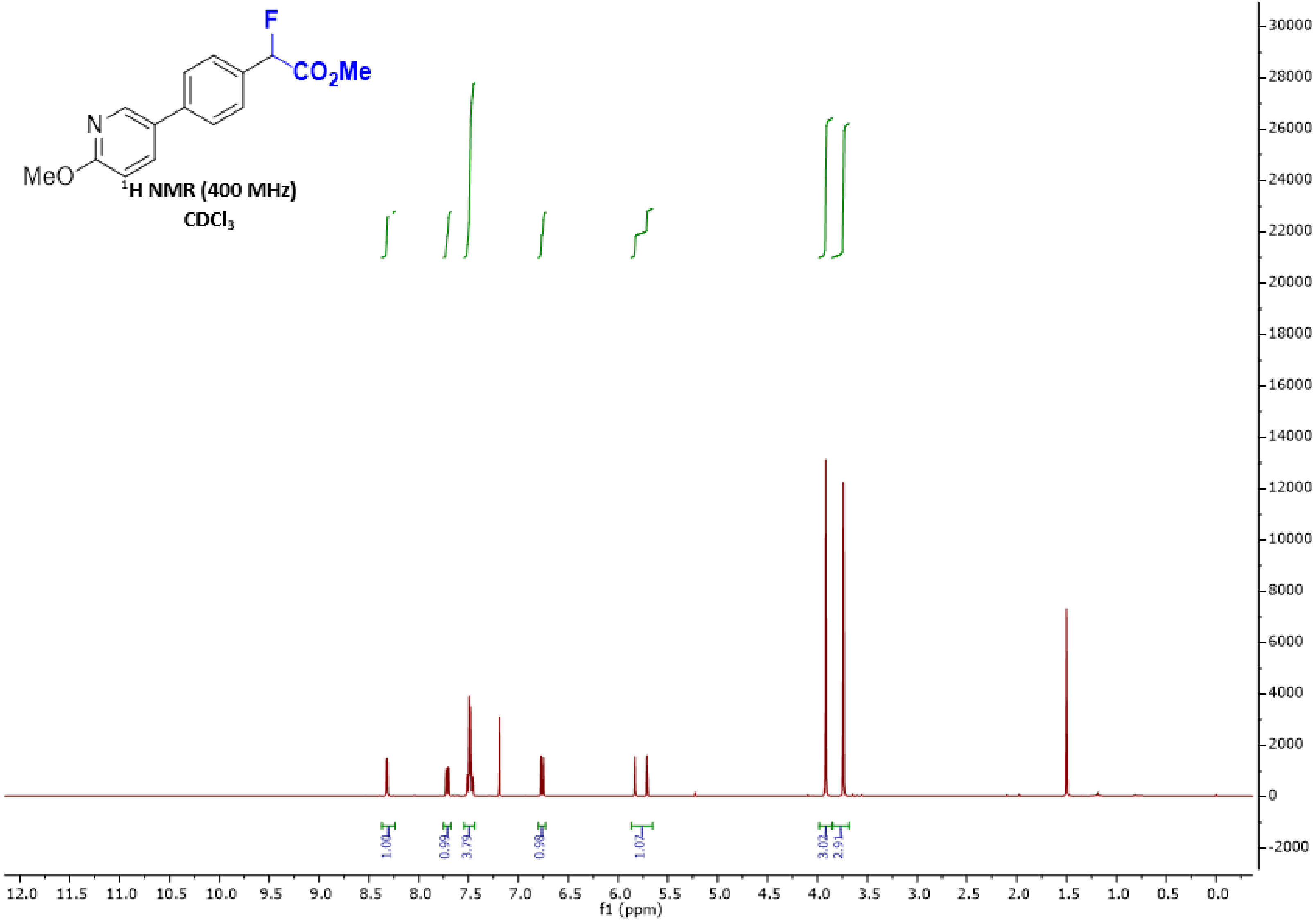
F
CO2Me
N
MeO
1H NMR (400 MHz)
CDCl3
1.00
0.99
3.79
0.98
1.02
3.02
2.91
f1 (ppm)

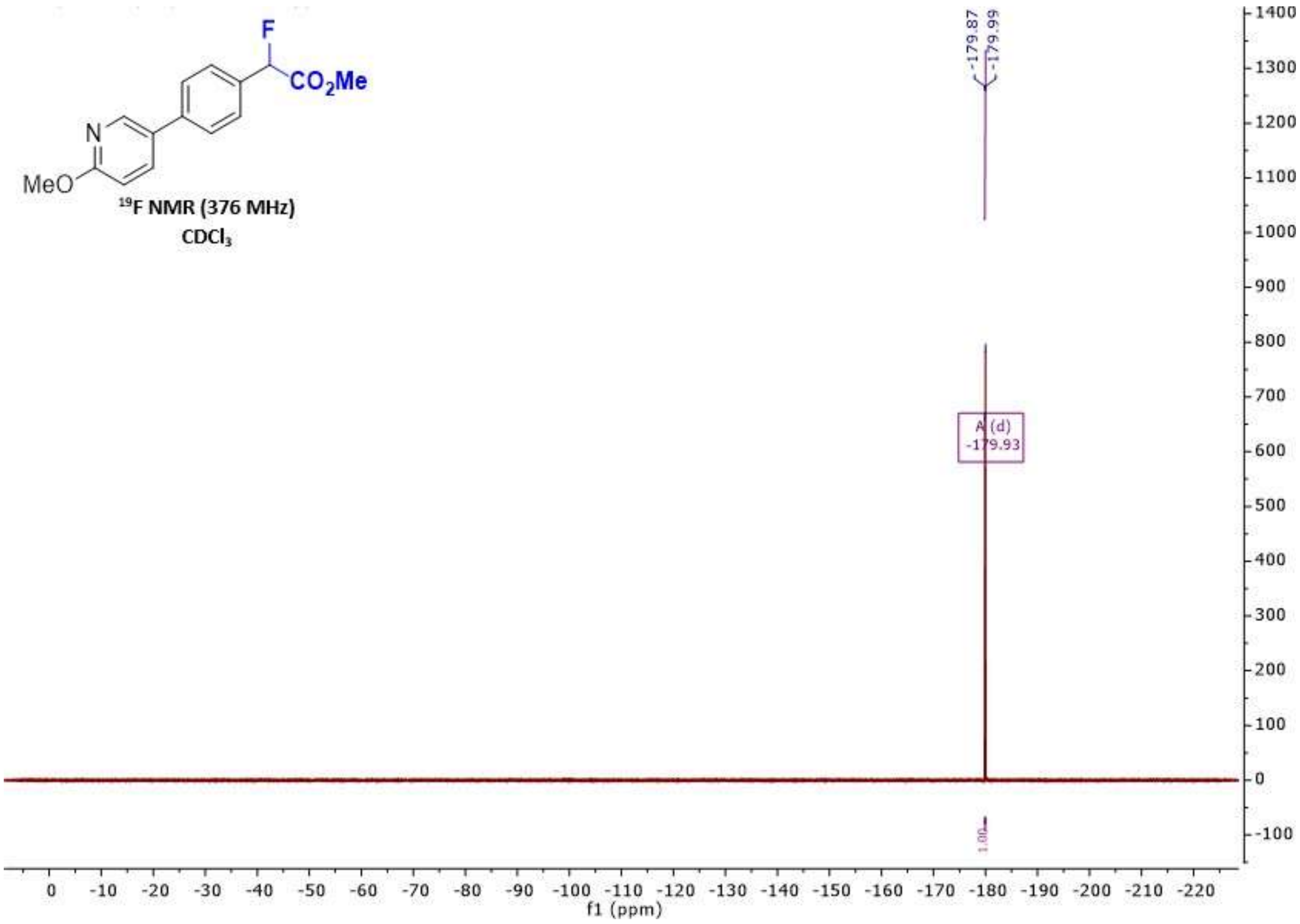
F
CO2Me
N
MeO
19F NMR (376 MHz)
CDCl3
-179.87
-179.99
A (d)
-179.93
1.00
f1 (ppm)

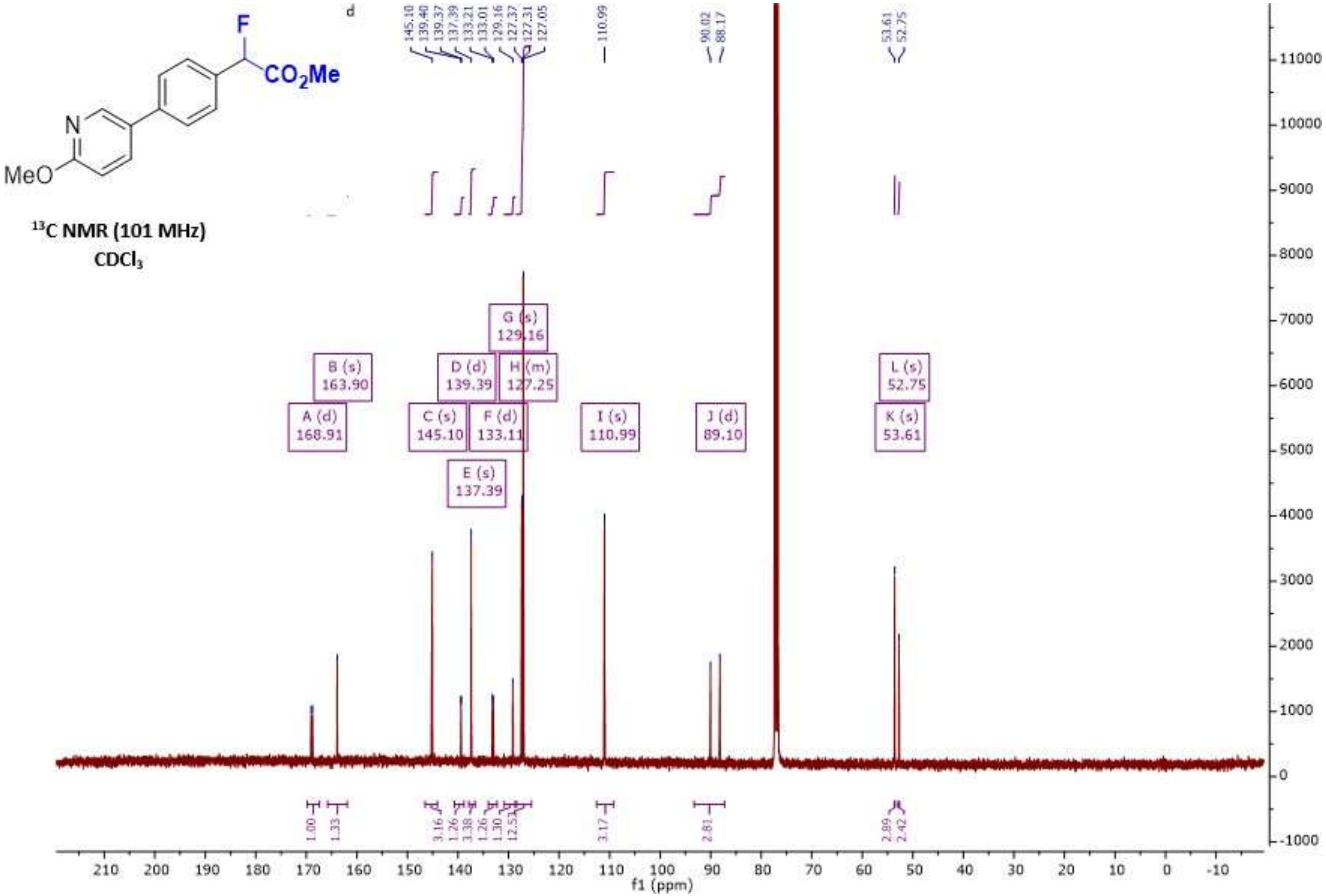
F
CO2Me
N
MeO
13C NMR (101 MHz)
CDCl3
145.10
139.40
139.37
137.39
133.21
133.01
129.16
127.37
127.31
127.05
110.99
90.02
88.17
53.61
52.75
A (d) 168.91
B (s) 163.90
C (s) 145.10
D (d) 139.39
E (s) 137.39
F (d) 133.11
G (s) 129.16
H (m) 127.25
I (s) 110.99
J (d) 89.10
K (s) 53.61
L (s) 52.75
1.00
1.33
3.16
1.26
3.38
1.26
1.30
12.53
3.17
2.81
2.89
2.42
f1 (ppm)

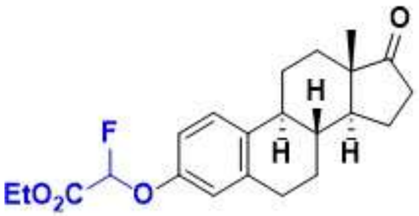


$^1$H NMR (400 MHz)
$CDCl_3$

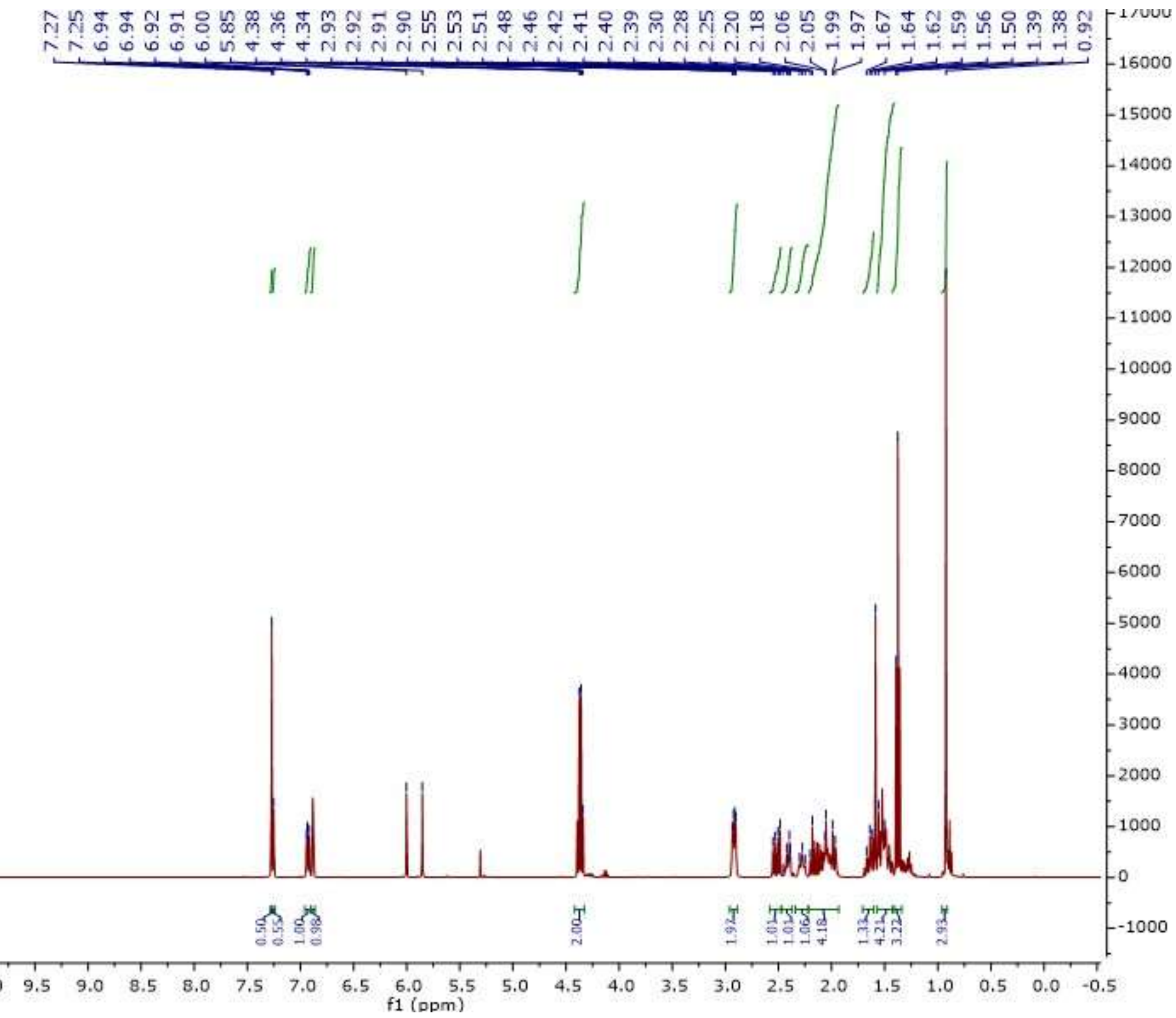


c

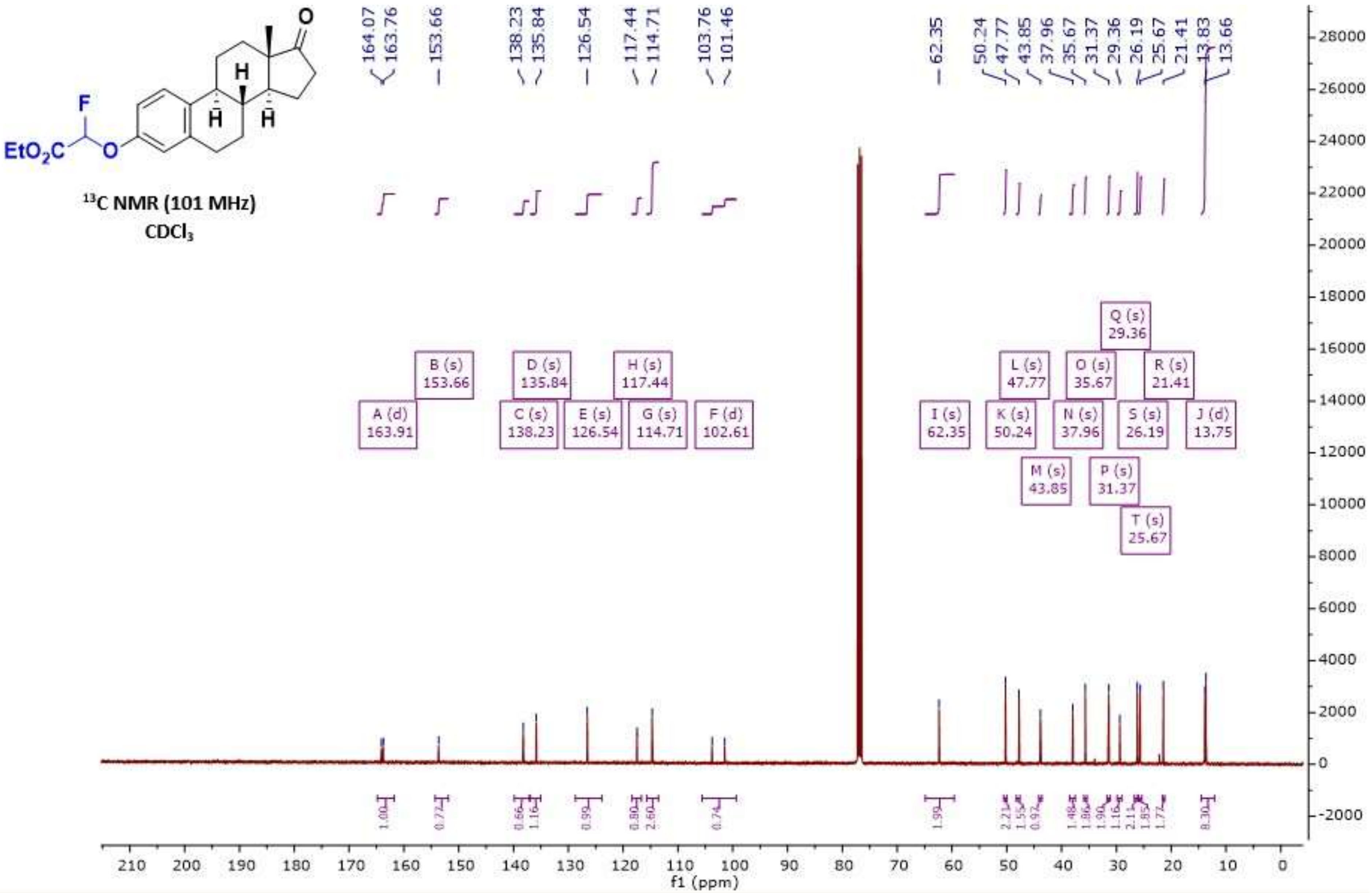

EtO2C
F
O
H
H
H
O
13C NMR (101 MHz)
CDCl3
164.07
163.76
153.66
138.23
135.84
126.54
117.44
114.71
103.76
101.46
62.35
50.24
47.77
43.85
37.96
35.67
31.37
29.36
26.19
25.67
21.41
13.83
13.66
A (d) 163.91
B (s) 153.66
C (s) 138.23
D (s) 135.84
E (s) 126.54
F (d) 102.61
G (s) 114.71
H (s) 117.44
I (s) 62.35
J (d) 13.75
K (s) 50.24
L (s) 47.77
M (s) 43.85
N (s) 37.96
O (s) 35.67
P (s) 31.37
Q (s) 29.36
R (s) 21.41
S (s) 26.19
T (s) 25.67
1.00
0.72
0.66
1.16
0.99
0.80
2.60
0.74
1.99
2.21
1.55
0.92
1.48
1.86
1.90
1.16
2.11
1.85
1.77
8.30
f1 (ppm)

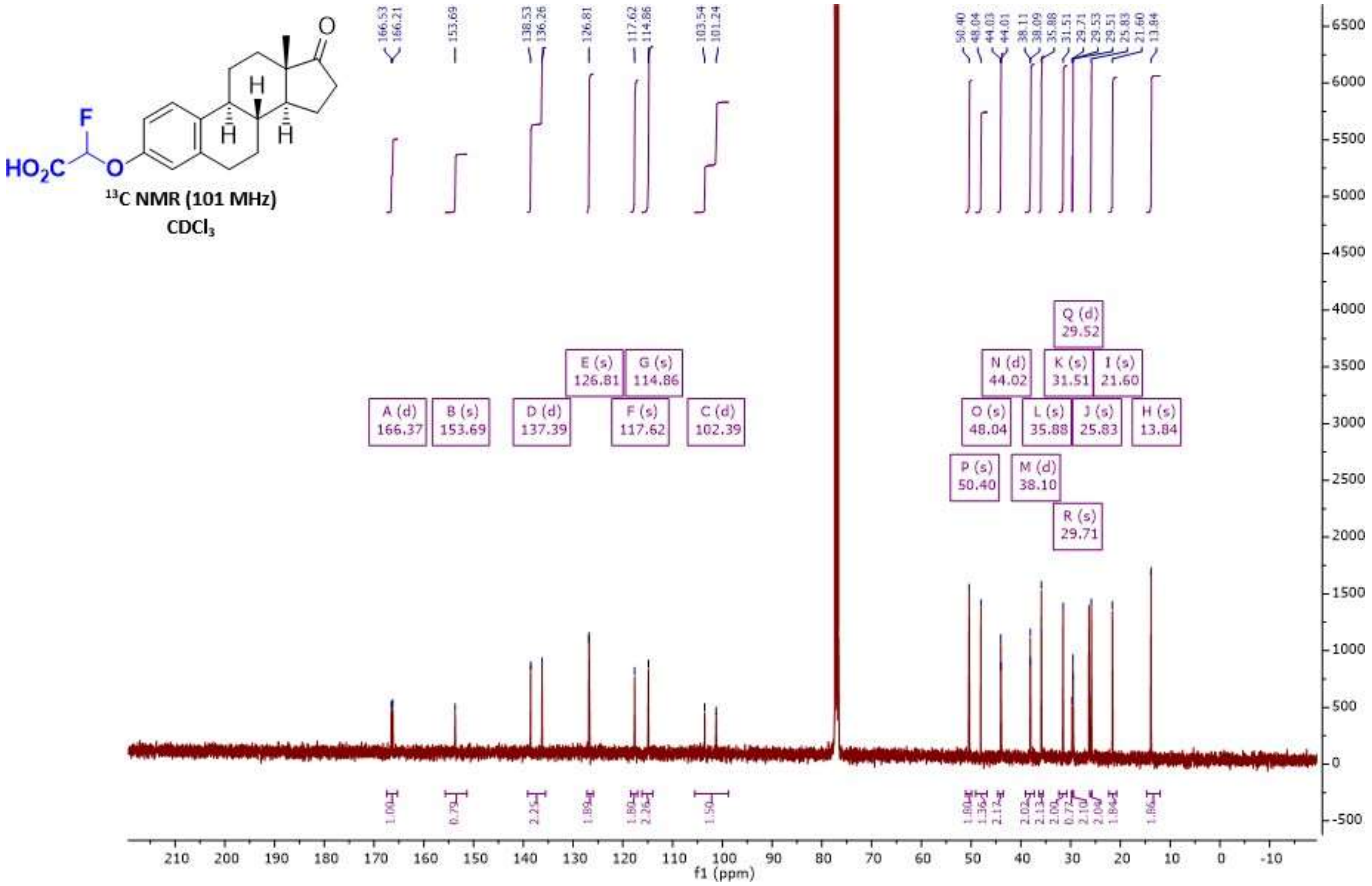

HO2C
F
O
H
H
H
O
13C NMR (101 MHz)
CDCl3
166.53
166.21
153.69
138.53
136.26
126.81
117.62
114.86
103.54
101.24
50.40
48.04
44.03
44.01
38.11
38.09
35.88
31.51
29.71
29.53
29.51
25.83
21.60
13.84
A (d) 166.37
B (s) 153.69
D (d) 137.39
E (s) 126.81
G (s) 114.86
F (s) 117.62
C (d) 102.39
Q (d) 29.52
N (d) 44.02
K (s) 31.51
I (s) 21.60
O (s) 48.04
L (s) 35.88
J (s) 25.83
H (s) 13.84
P (s) 50.40
M (d) 38.10
R (s) 29.71
1.00
0.79
2.25
1.89
1.80
2.26
1.50
1.80
1.36
2.17
2.02
2.13
2.00
0.77
2.10
2.04
1.84
1.86
f1 (ppm)

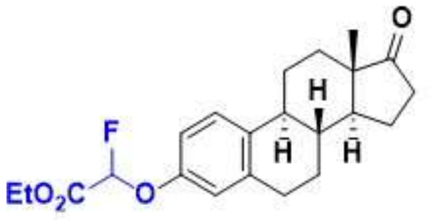

$^{19}$F NMR (376 MHz)
$CDCl_3$

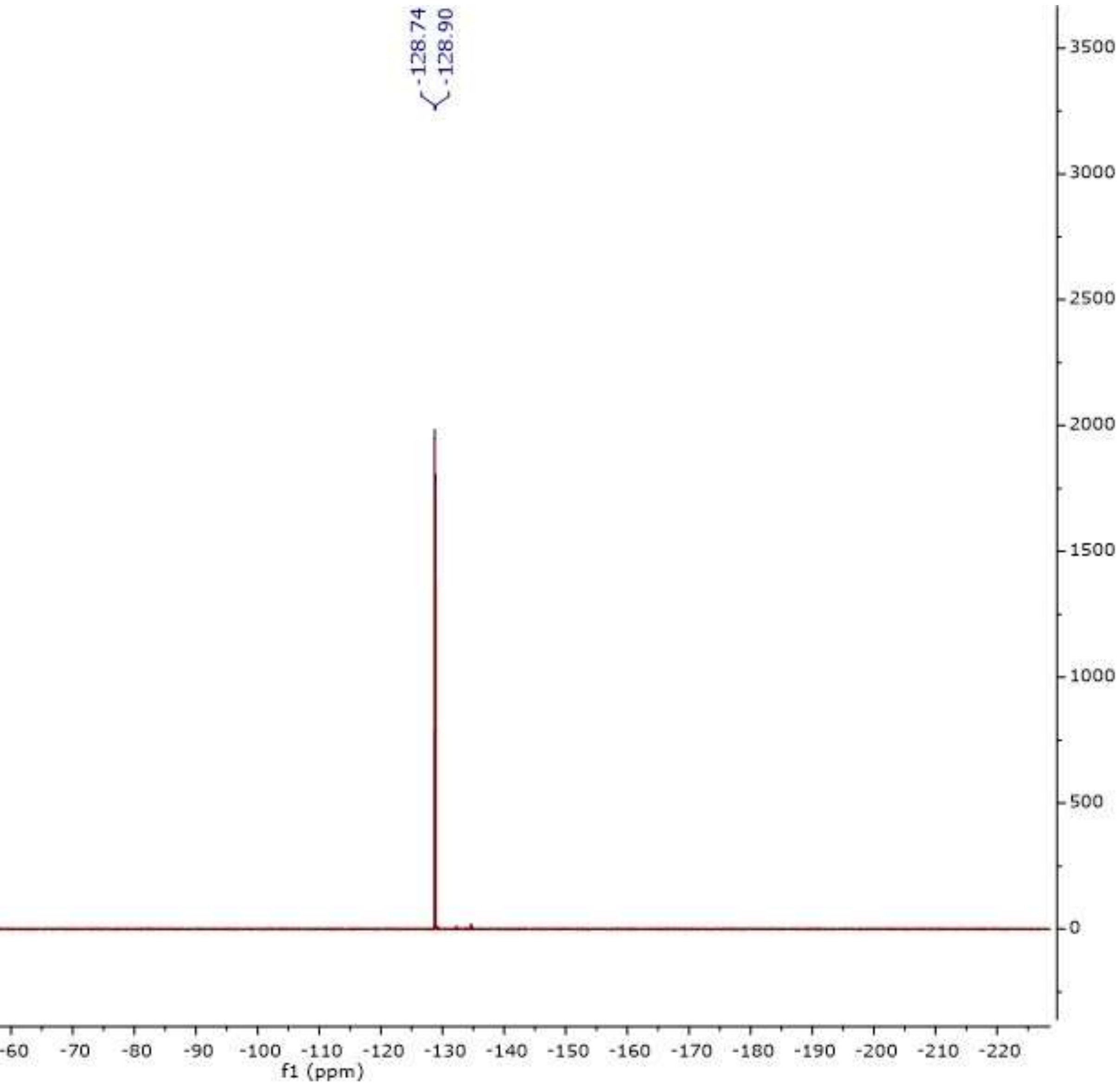

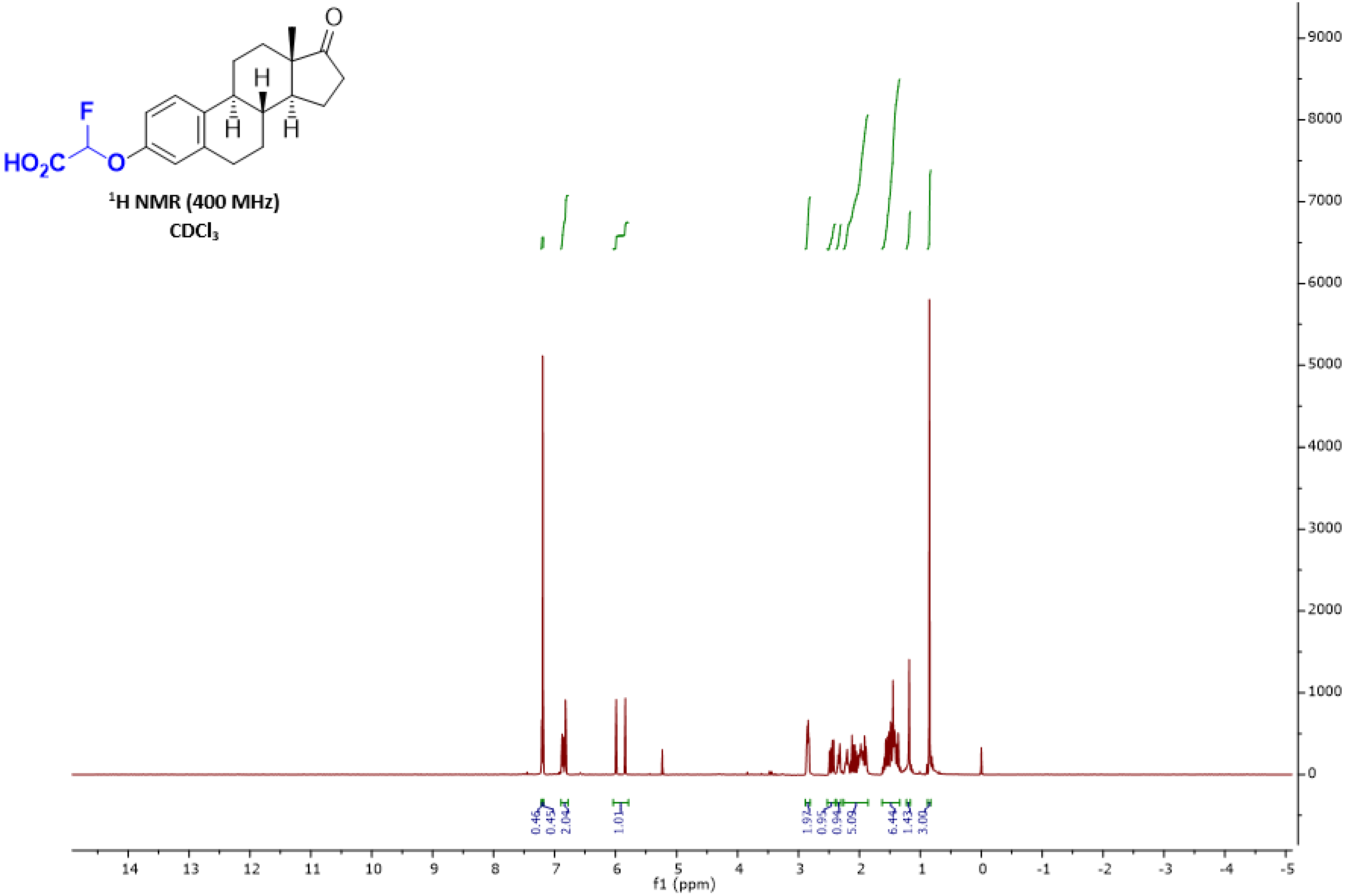

HO2C
F
O
O
H
H
H
1H NMR (400 MHz)
CDCl3
0.46
0.45
2.04
1.01
1.92
0.95
0.94
5.09
6.44
1.43
3.00
f1 (ppm)

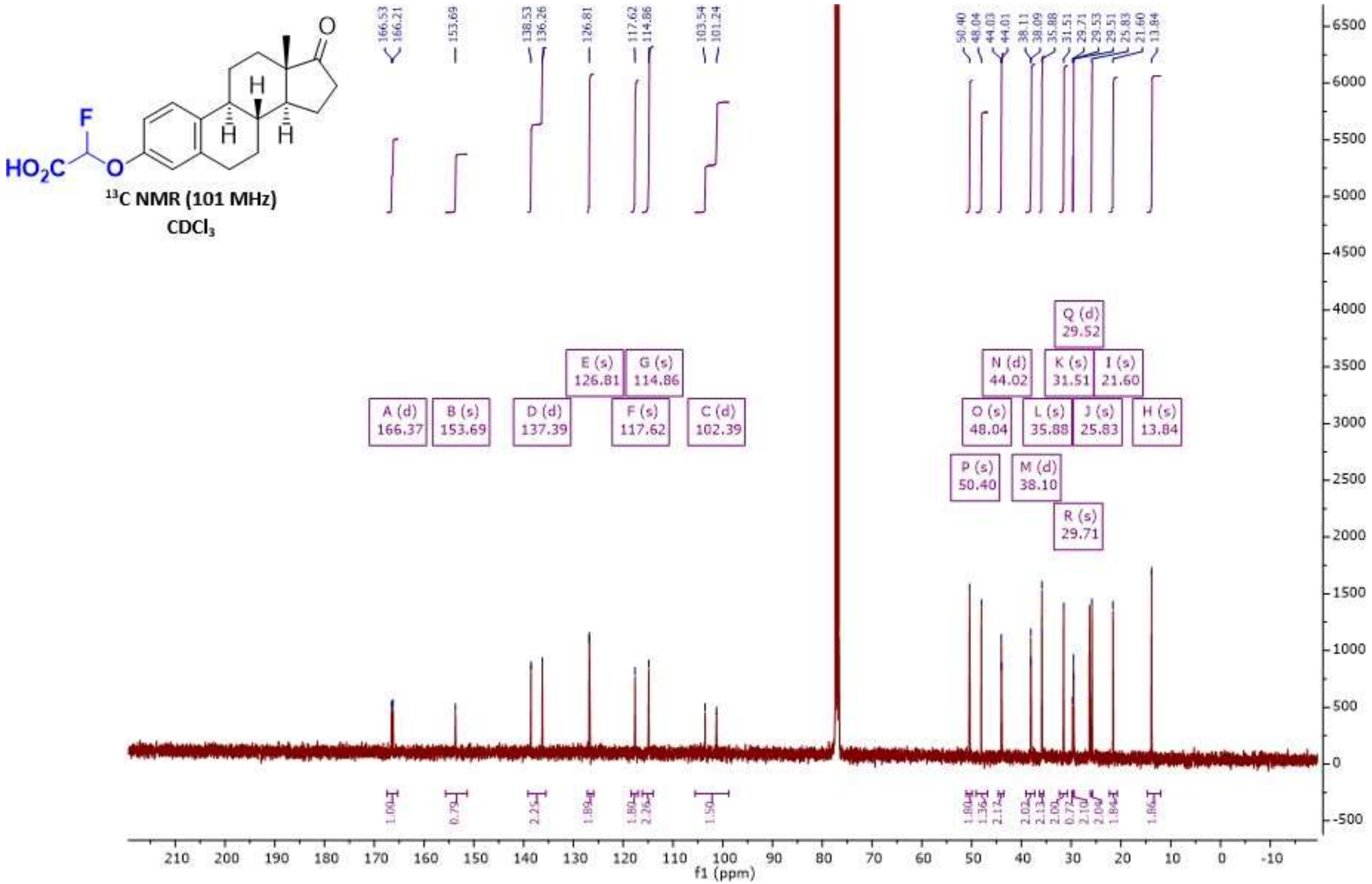

F
HO2C
O
H
13C NMR (101 MHz)
CDCl3
166.53
166.21
153.69
138.53
136.26
126.81
117.62
114.86
103.54
101.24
50.40
48.04
44.03
44.01
38.11
38.09
35.88
31.51
29.71
29.53
29.51
25.83
21.60
13.84
A (d) 166.37
B (s) 153.69
D (d) 137.39
E (s) 126.81
G (s) 114.86
F (s) 117.62
C (d) 102.39
Q (d) 29.52
N (d) 44.02
K (s) 31.51
I (s) 21.60
O (s) 48.04
L (s) 35.88
J (s) 25.83
H (s) 13.84
P (s) 50.40
M (d) 38.10
R (s) 29.71
1.00
0.79
2.25
1.89
1.80
2.26
1.50
1.80
1.36
2.17
2.02
2.13
2.00
0.72
2.10
2.04
1.84
1.86
f1 (ppm)

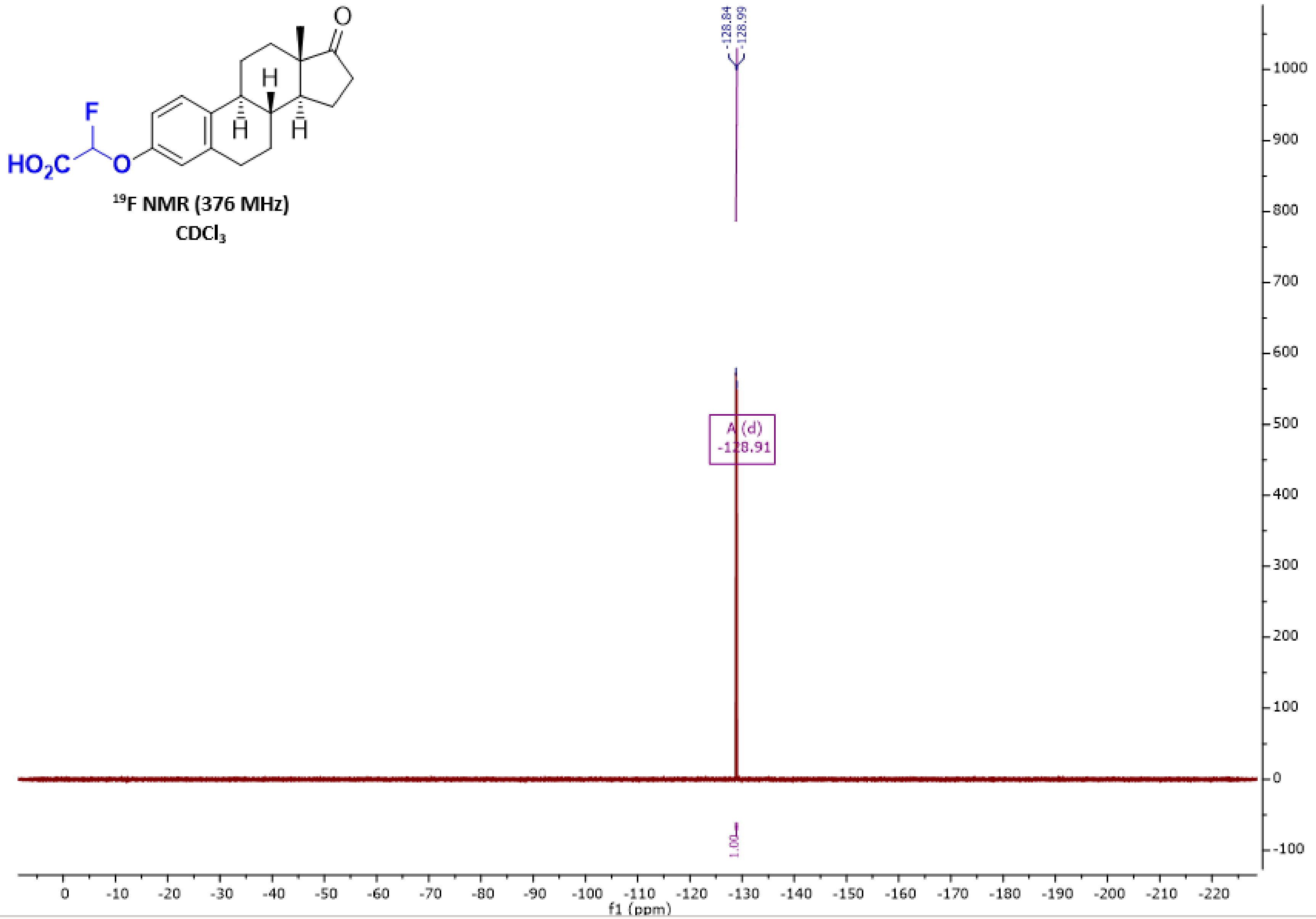

HO2C
F
O
O
H
H
H
19F NMR (376 MHz)
CDCl3
-128.84
-128.99
A (d)
-128.91
1.00
f1 (ppm)

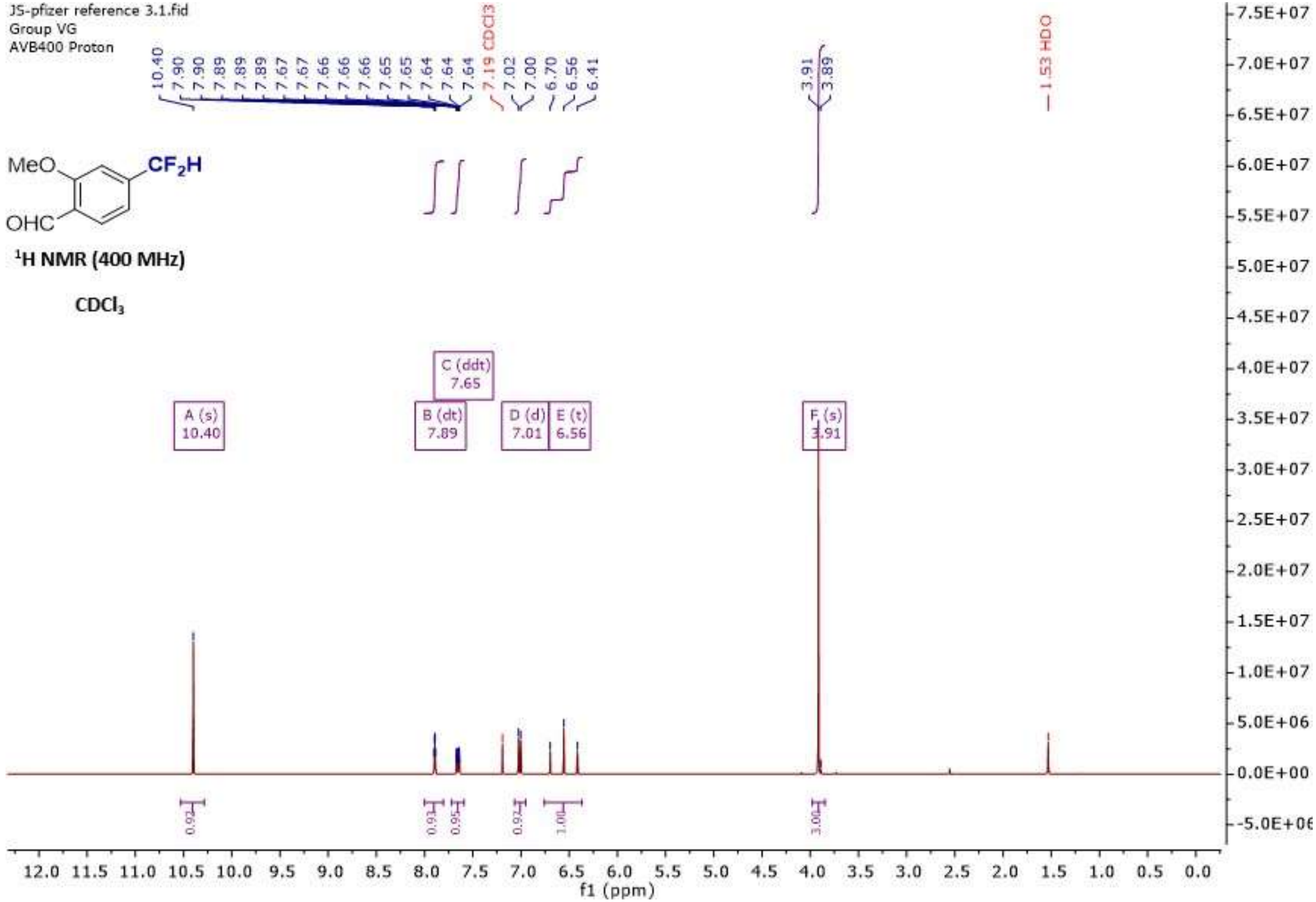

JS-pfizer reference 3.1.fid
Group VG
AVB400 Proton
10.40
7.90
7.90
7.89
7.89
7.89
7.67
7.67
7.66
7.66
7.66
7.65
7.65
7.64
7.64
7.64
7.19 CDCl3
7.02
7.00
6.70
6.56
6.41
3.91
3.89
1.53 HDO
MeO
CF2H
OHC
1H NMR (400 MHz)
CDCl3
A (s) 10.40
B (dt) 7.89
C (ddt) 7.65
D (d) 7.01
E (t) 6.56
F (s) 3.91
0.93
0.91
0.95
0.92
1.00
3.00
f1 (ppm)

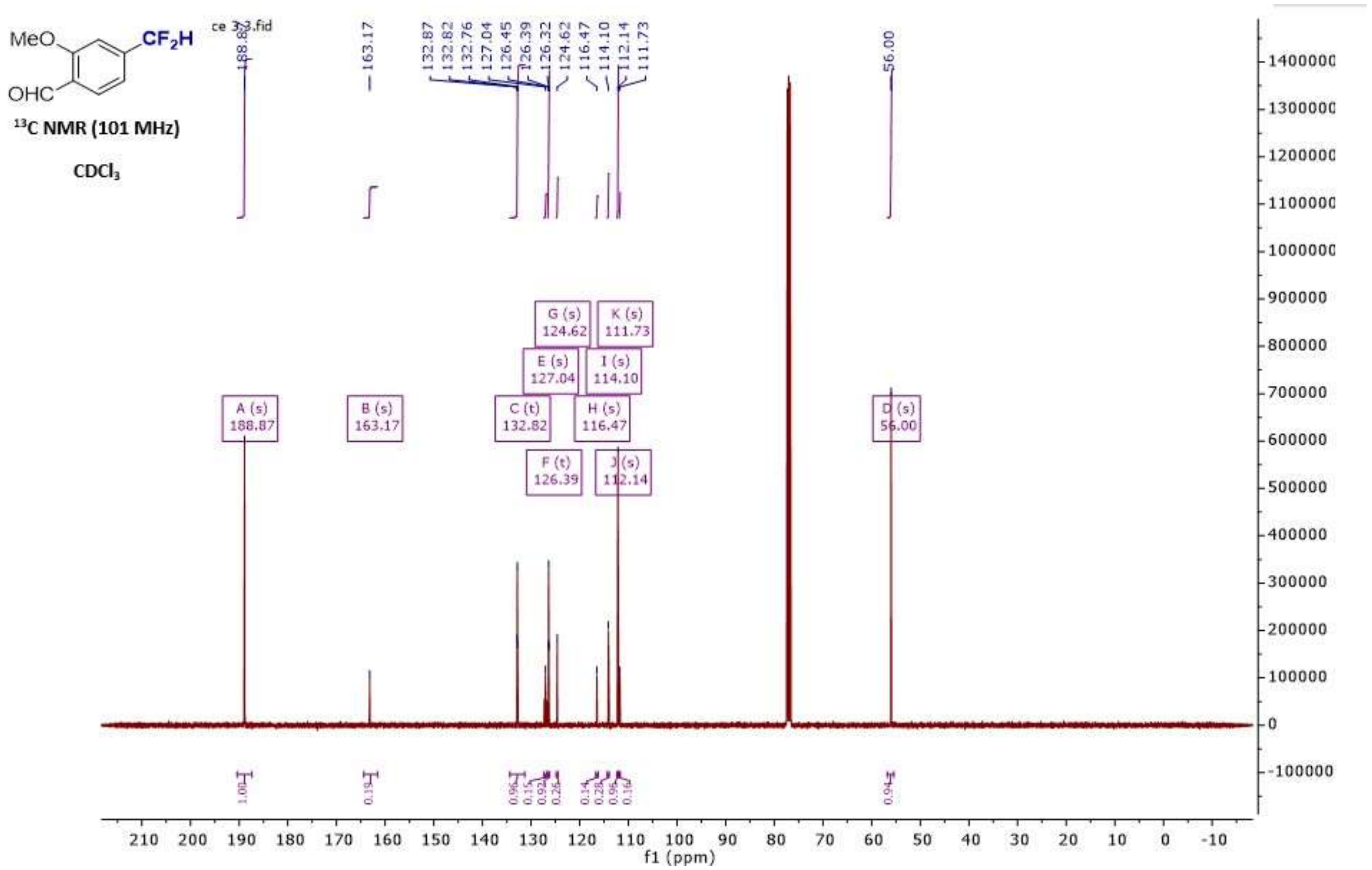
MeO
CF2H
OHC
13C NMR (101 MHz)
CDCl3
ce 3-3.fid
188.87
163.17
132.87
132.82
132.76
127.04
126.45
126.39
126.32
124.62
116.47
114.10
112.14
111.73
56.00
A (s) 188.87
B (s) 163.17
C (t) 132.82
D (s) 56.00
E (s) 127.04
F (t) 126.39
G (s) 124.62
H (s) 116.47
I (s) 114.10
J (s) 112.14
K (s) 111.73
1.00
0.19
0.96
0.15
0.92
0.26
0.14
0.28
0.95
0.16
0.94
f1 (ppm)

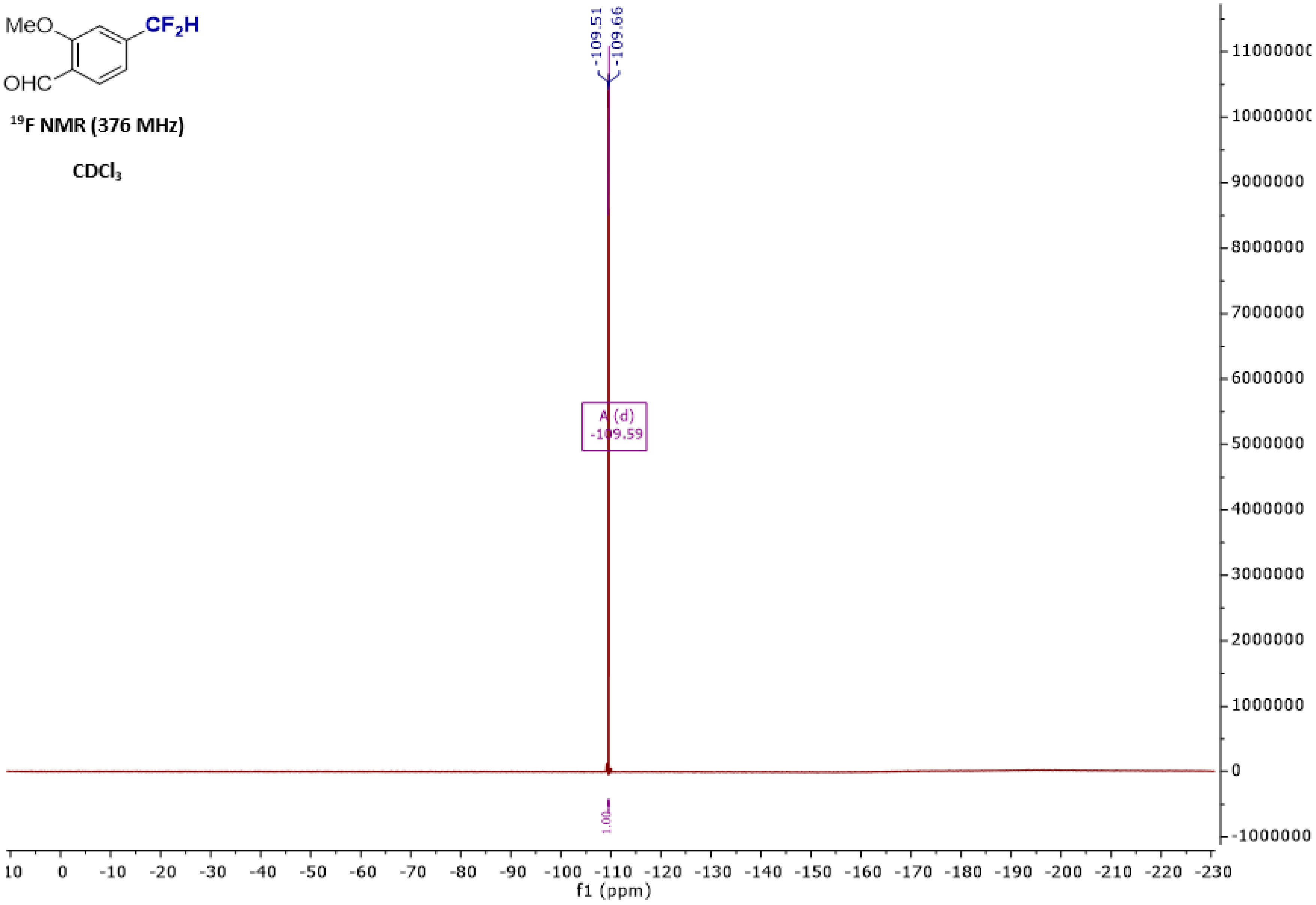
MeO
CF2H
OHC
19F NMR (376 MHz)
CDCl3
-109.51
-109.66
A (d)
-109.59
1.00
f1 (ppm)

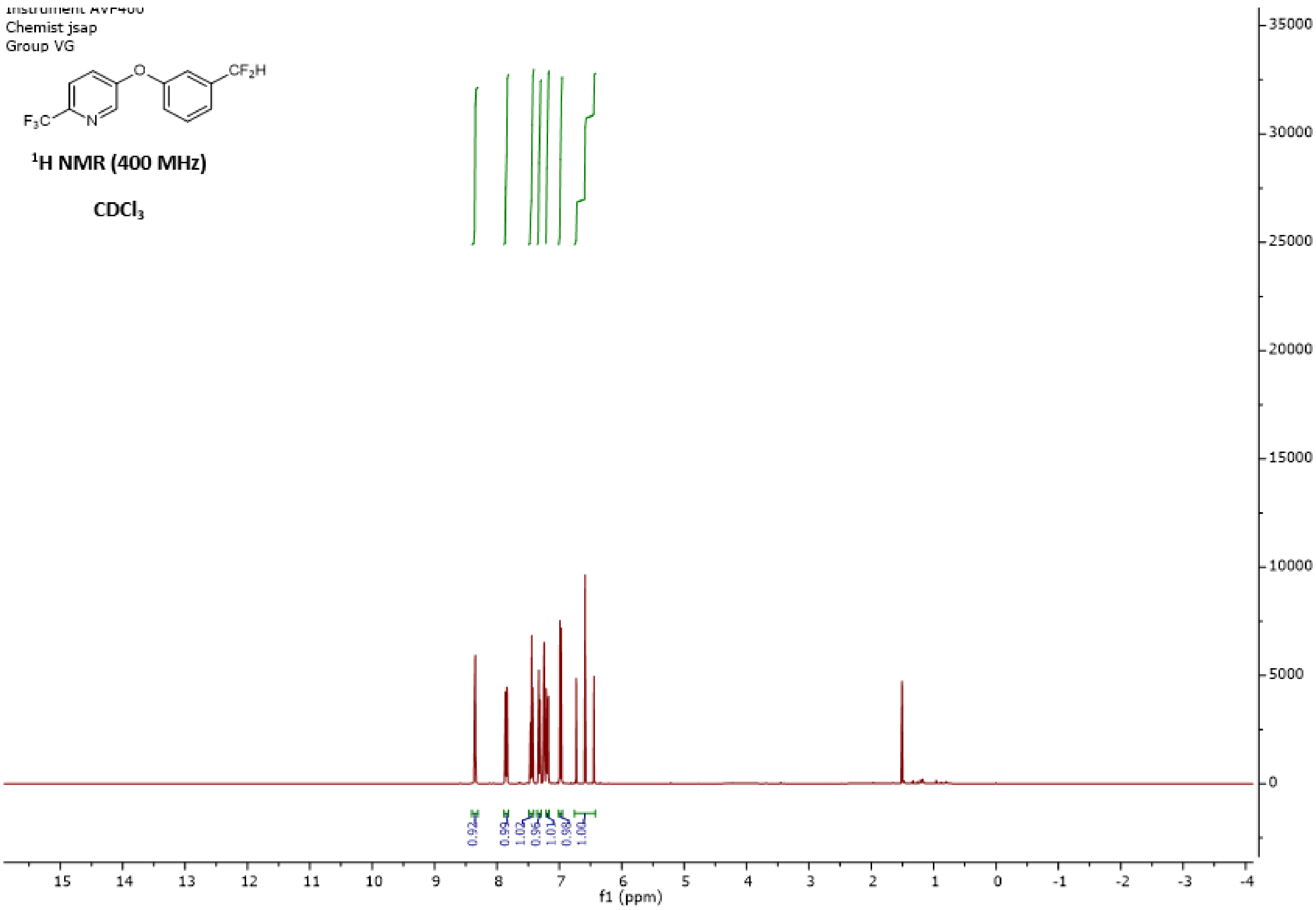

Chemist jsap
Group VG
CF2H
O
F3C
N
1H NMR (400 MHz)
CDCl3
0.92
0.99
1.03
0.96
1.01
0.98
1.00
f1 (ppm)
15 14 13 12 11 10 9 8 7 6 5 4 3 2 1 0 -1 -2 -3 -4
35000 30000 25000 20000 15000 10000 5000 0

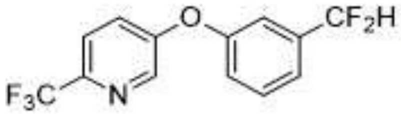


$^1$H NMR (101 MHz)
$CDCl_3$

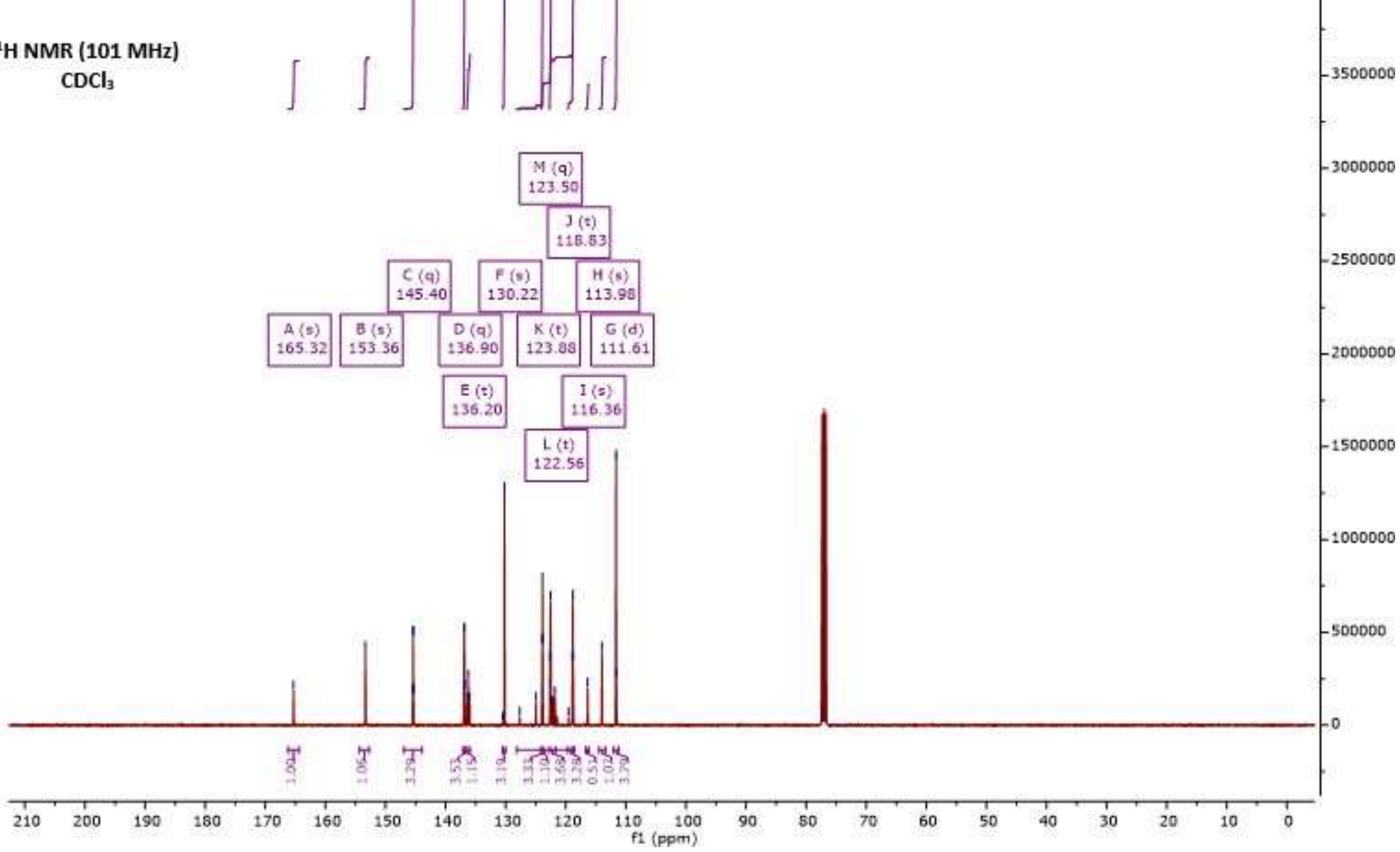

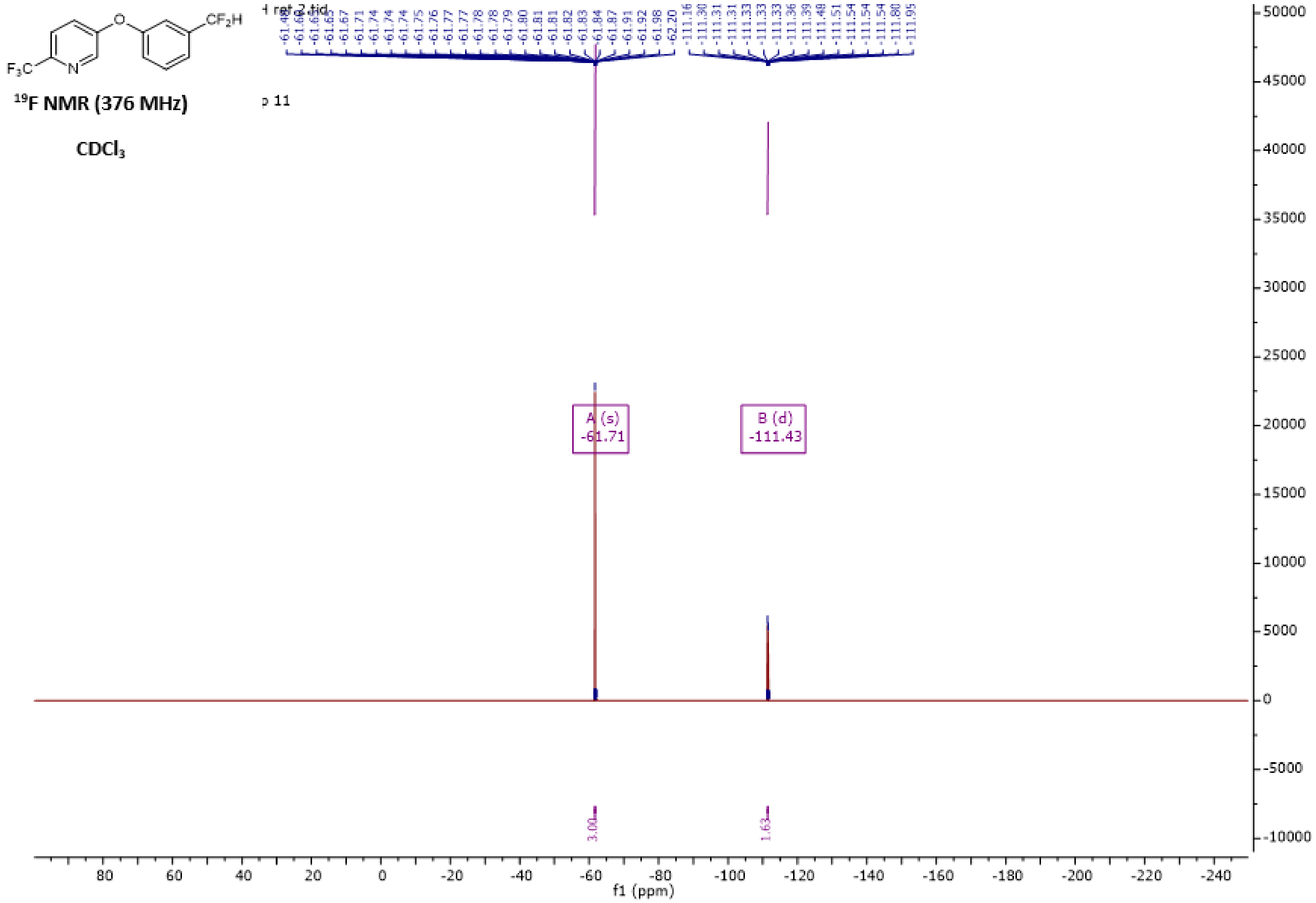

CF2H
F3C
N
O
19F NMR (376 MHz)
CDCl3
p 11
-61.65
-61.67
-61.71
-61.74
-61.74
-61.74
-61.75
-61.76
-61.77
-61.77
-61.78
-61.78
-61.79
-61.80
-61.81
-61.81
-61.82
-61.83
-61.84
-61.87
-61.91
-61.92
-61.98
-62.20
-111.16
-111.30
-111.31
-111.31
-111.33
-111.33
-111.33
-111.36
-111.39
-111.48
-111.51
-111.54
-111.54
-111.54
-111.80
-111.95
A (s)
-61.71
B (d)
-111.43
3.00
1.63
f1 (ppm)

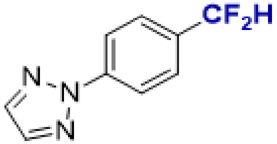


$^{19}$F NMR (376 MHz)

$CDCl_3$

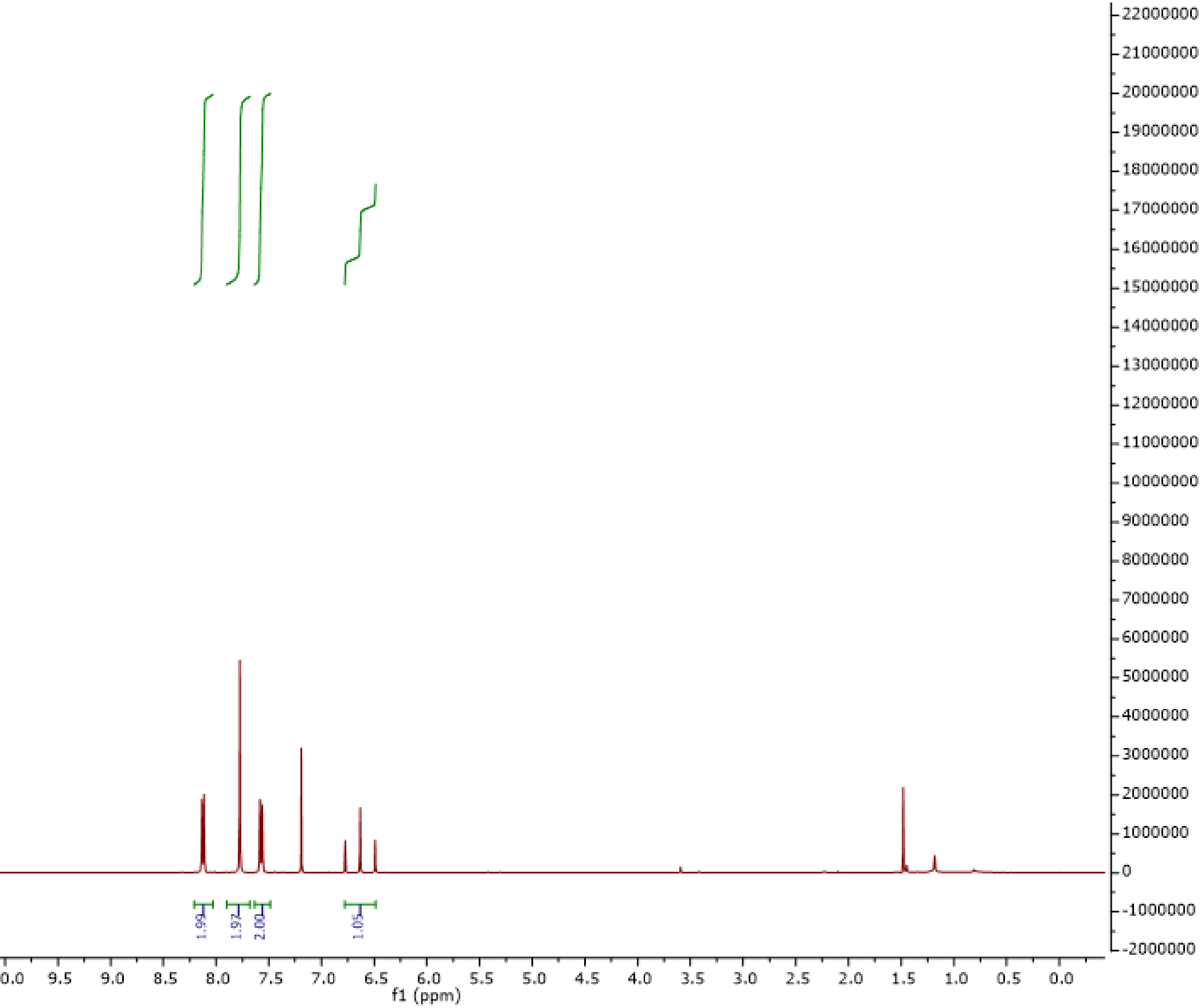

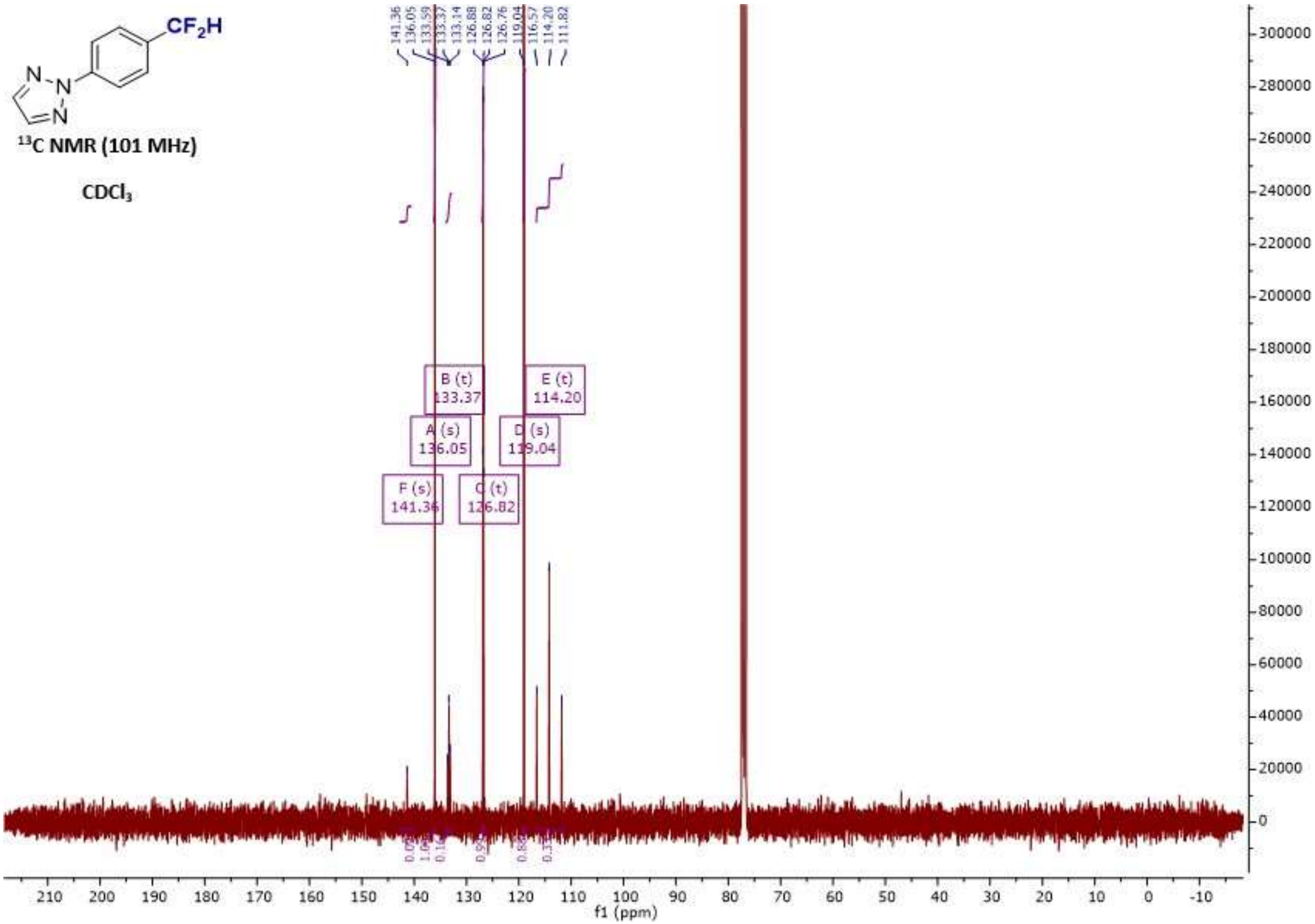
CF2H
N
N
N
13C NMR (101 MHz)
CDCl3
141.36
136.05
133.59
133.37
133.14
126.88
126.82
126.76
119.04
116.57
114.20
111.82
B (t)
133.37
E (t)
114.20
A (s)
136.05
D (s)
119.04
F (s)
141.36
C (t)
126.82
f1 (ppm)

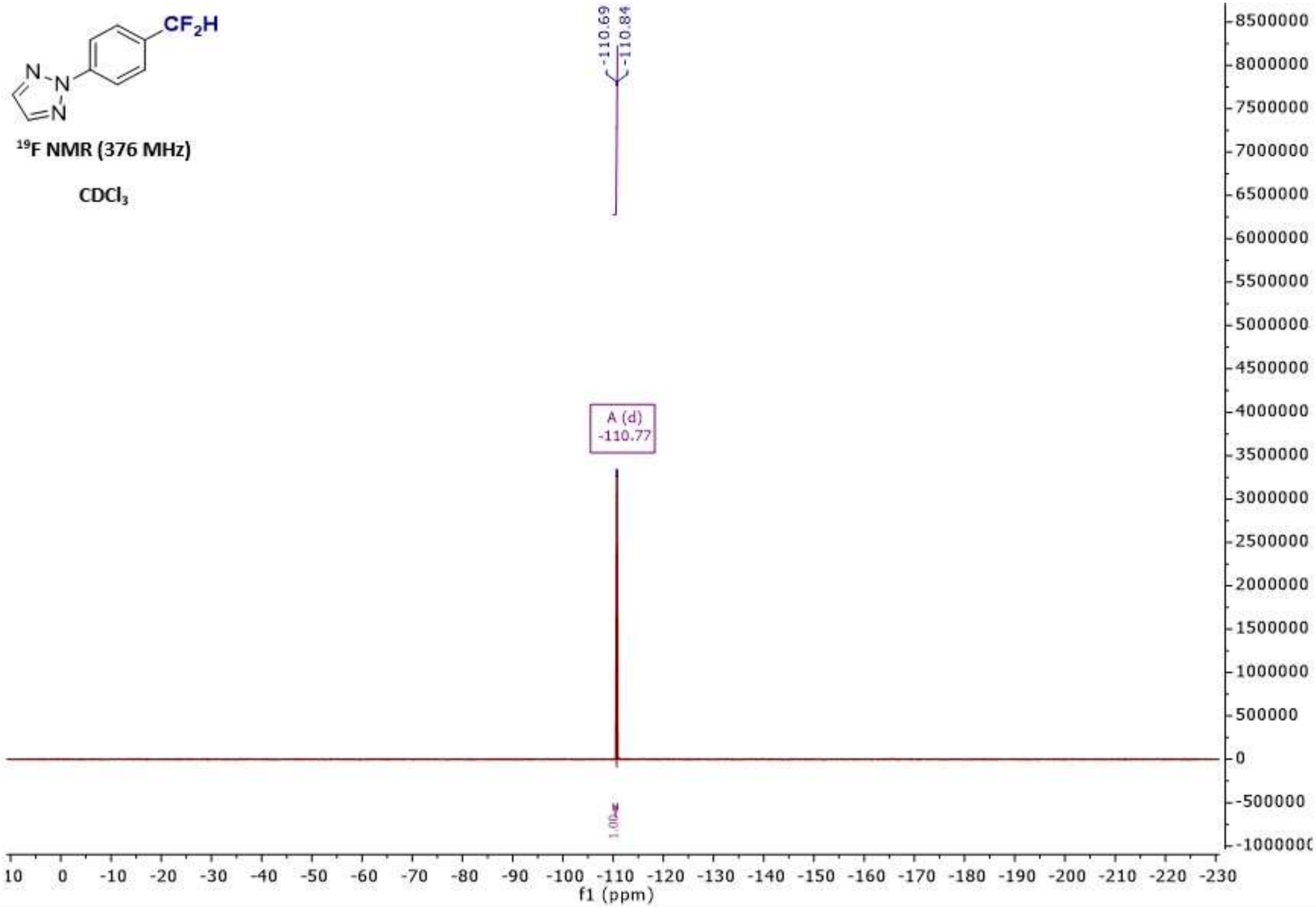

CF$_2$H
N
N
N
$^{19}$F NMR (376 MHz)
CDCl$_3$
-110.69
-110.84
A (d)
-110.77
1.00
f1 (ppm)

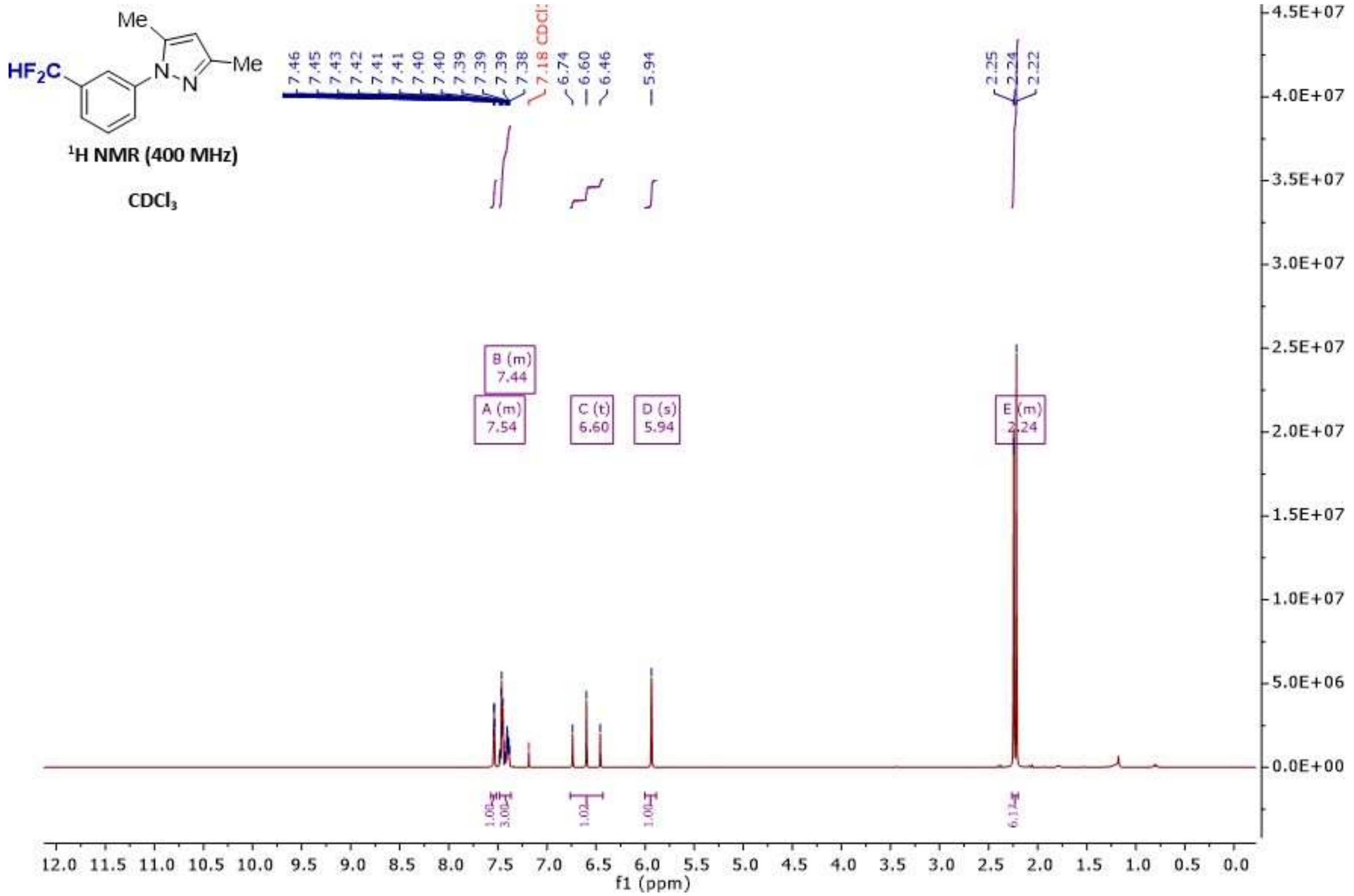

Me
HF2C
N
N
Me
¹H NMR (400 MHz)
CDCl3
7.46
7.45
7.43
7.42
7.41
7.41
7.40
7.40
7.39
7.39
7.39
7.38
7.18 CDCl3
6.74
6.60
6.46
5.94
2.25
2.24
2.22
B (m) 7.44
A (m) 7.54
C (t) 6.60
D (s) 5.94
E (m) 2.24
1.08
3.00
1.02
1.00
6.17
f1 (ppm)

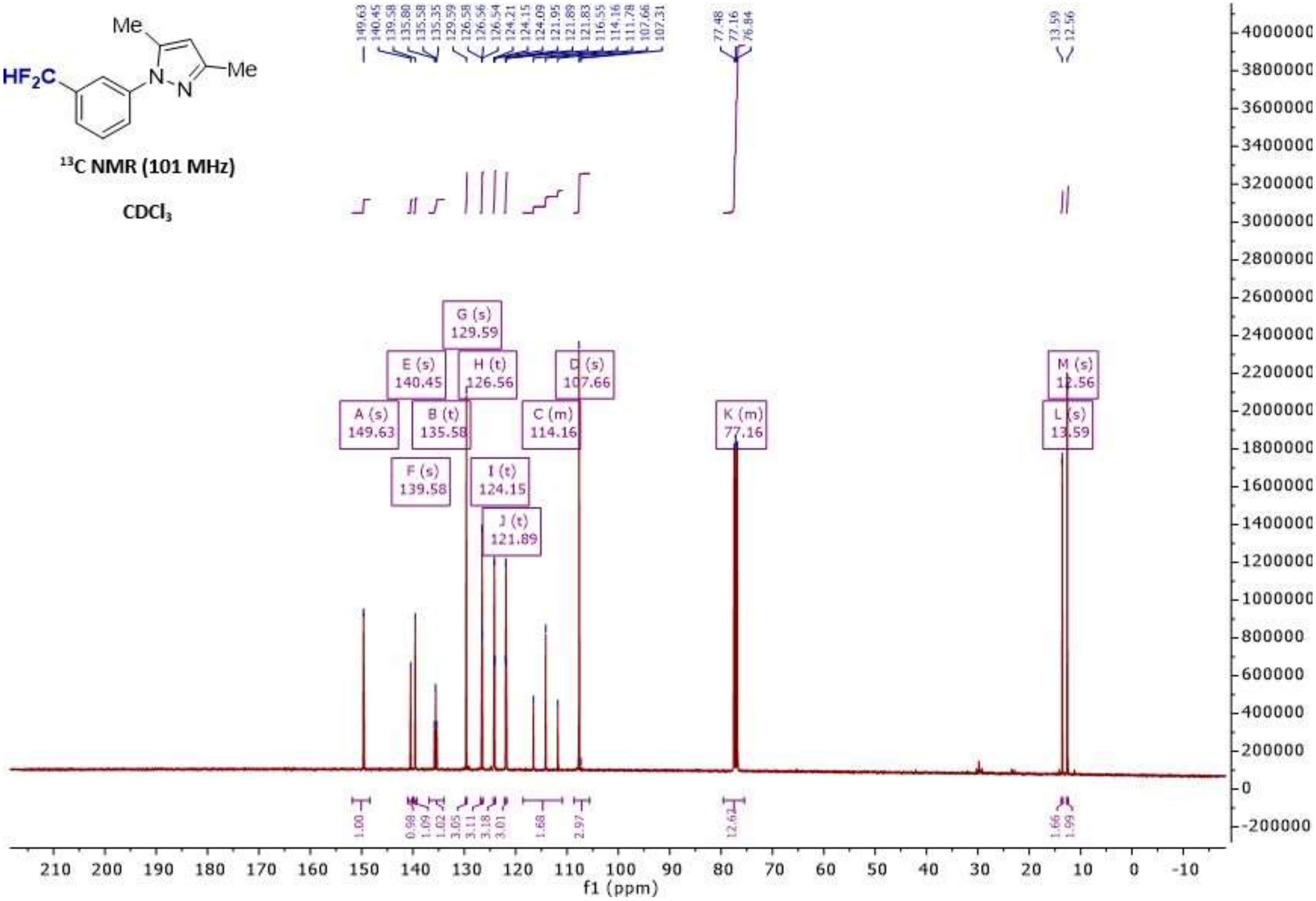

Me
HF2C
N
N
Me
13C NMR (101 MHz)
CDCl3
149.63
140.45
139.58
135.80
135.58
135.35
129.59
126.58
126.56
126.54
124.21
124.15
124.09
121.95
121.89
121.83
116.55
114.16
111.78
107.66
107.31
77.48
77.16
76.84
13.59
12.56
A (s) 149.63
B (t) 135.58
C (m) 114.16
D (s) 107.66
E (s) 140.45
F (s) 139.58
G (s) 129.59
H (t) 126.56
I (t) 124.15
J (t) 121.89
K (m) 77.16
L (s) 13.59
M (s) 12.56
f1 (ppm)

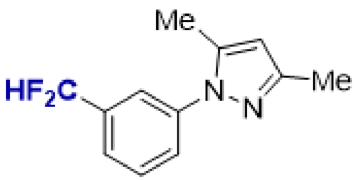


**$^{19}$F NMR (376 MHz)**

**$CDCl_3$**

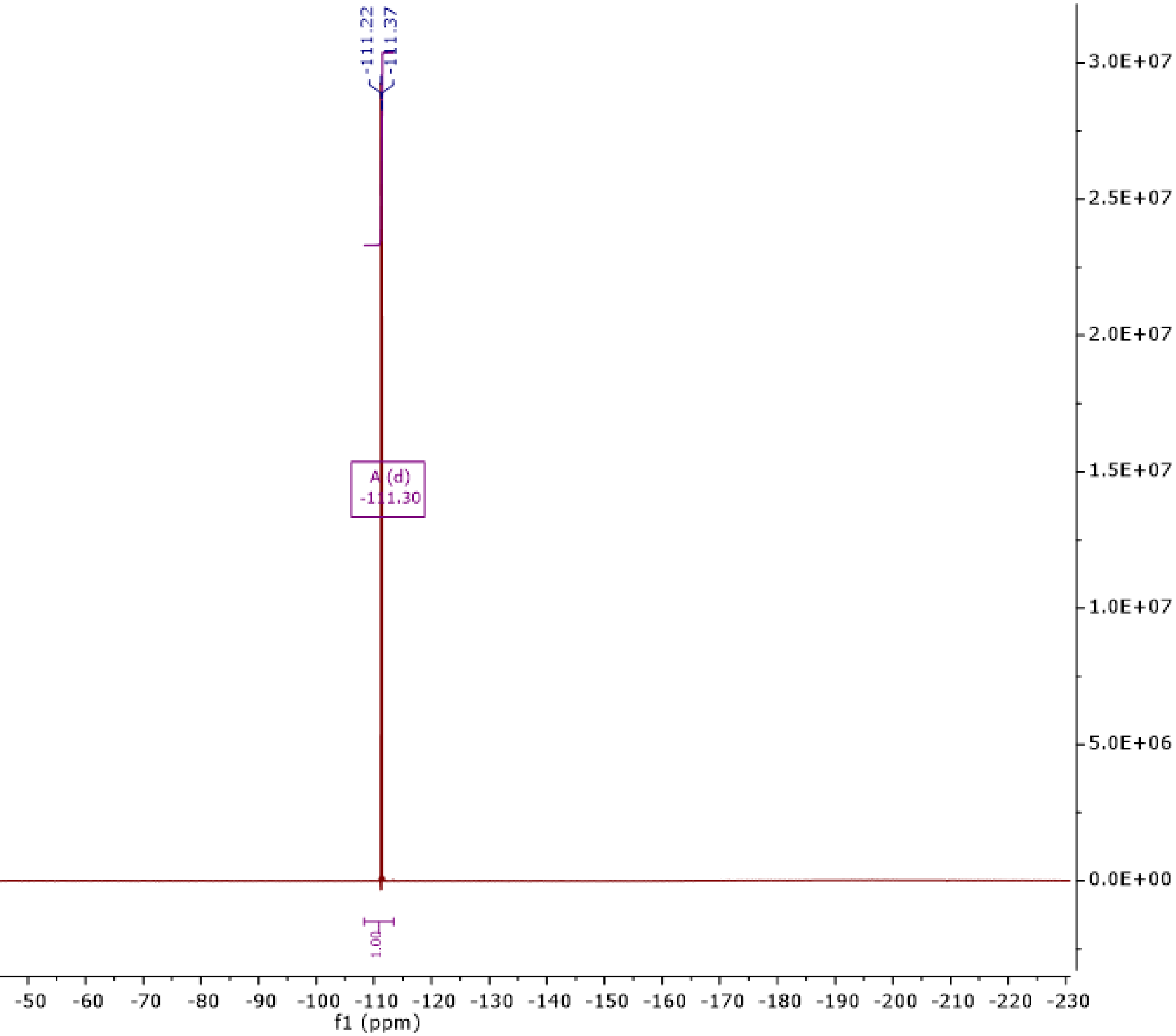

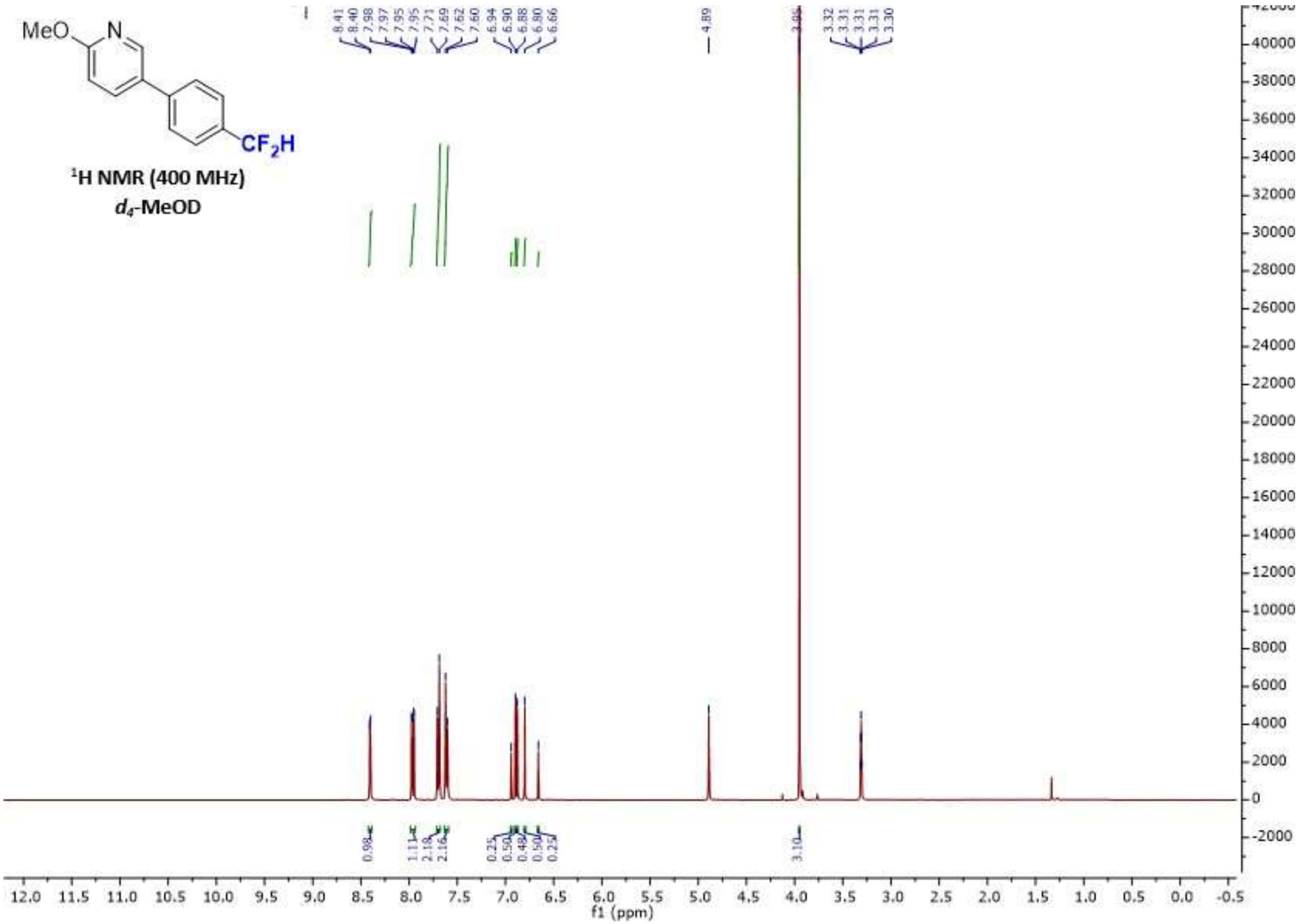
MeO
N
CF2H
1H NMR (400 MHz)
d4-MeOD
f1 (ppm)

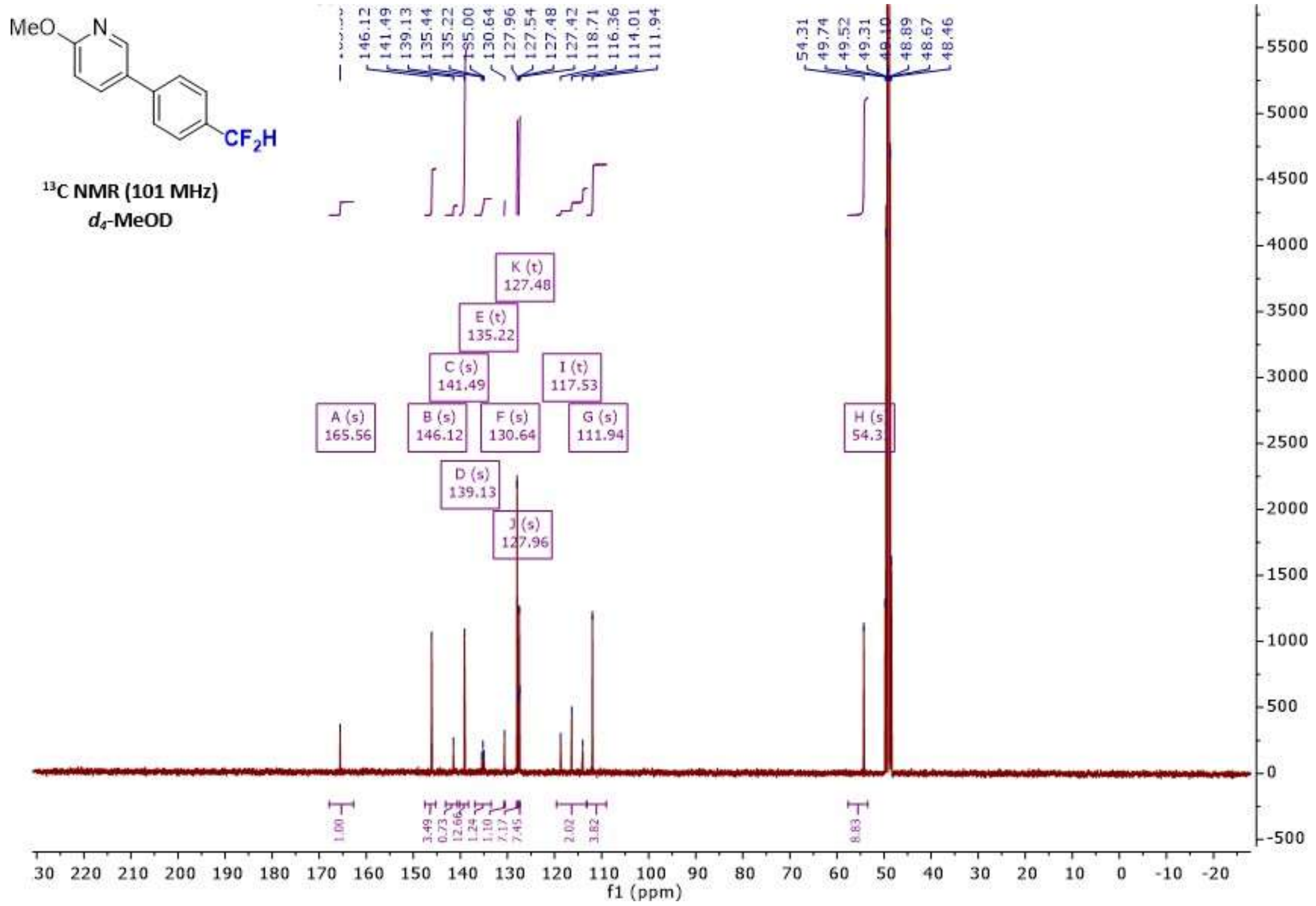
MeO
N
CF2H
13C NMR (101 MHz)
d4-MeOD
146.12
141.49
139.13
135.44
135.22
135.00
130.64
127.96
127.54
127.48
127.42
118.71
116.36
114.01
111.94
54.31
49.74
49.52
49.31
48.89
48.67
48.46
K (t) 127.48
E (t) 135.22
C (s) 141.49
I (t) 117.53
A (s) 165.56
B (s) 146.12
F (s) 130.64
G (s) 111.94
D (s) 139.13
J (s) 127.96
1.00
3.49
0.73
12.66
1.24
1.10
7.17
7.45
2.02
3.82
8.83
f1 (ppm)

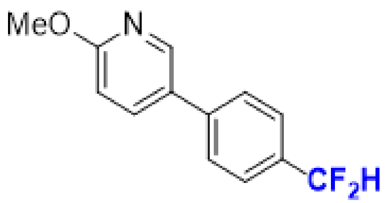


$^{19}$F NMR (376 MHz)
*d*$_4$-MeOD

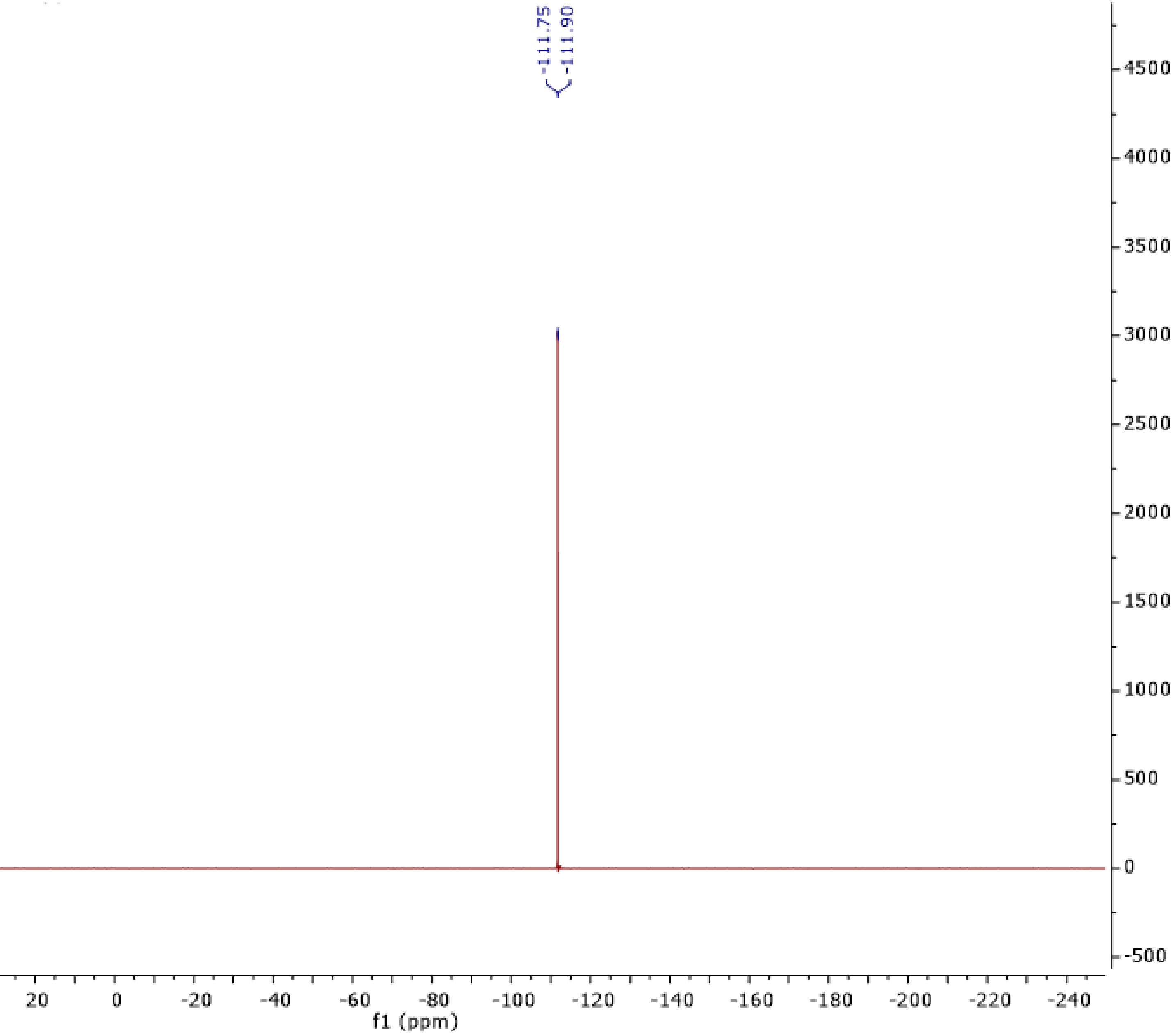

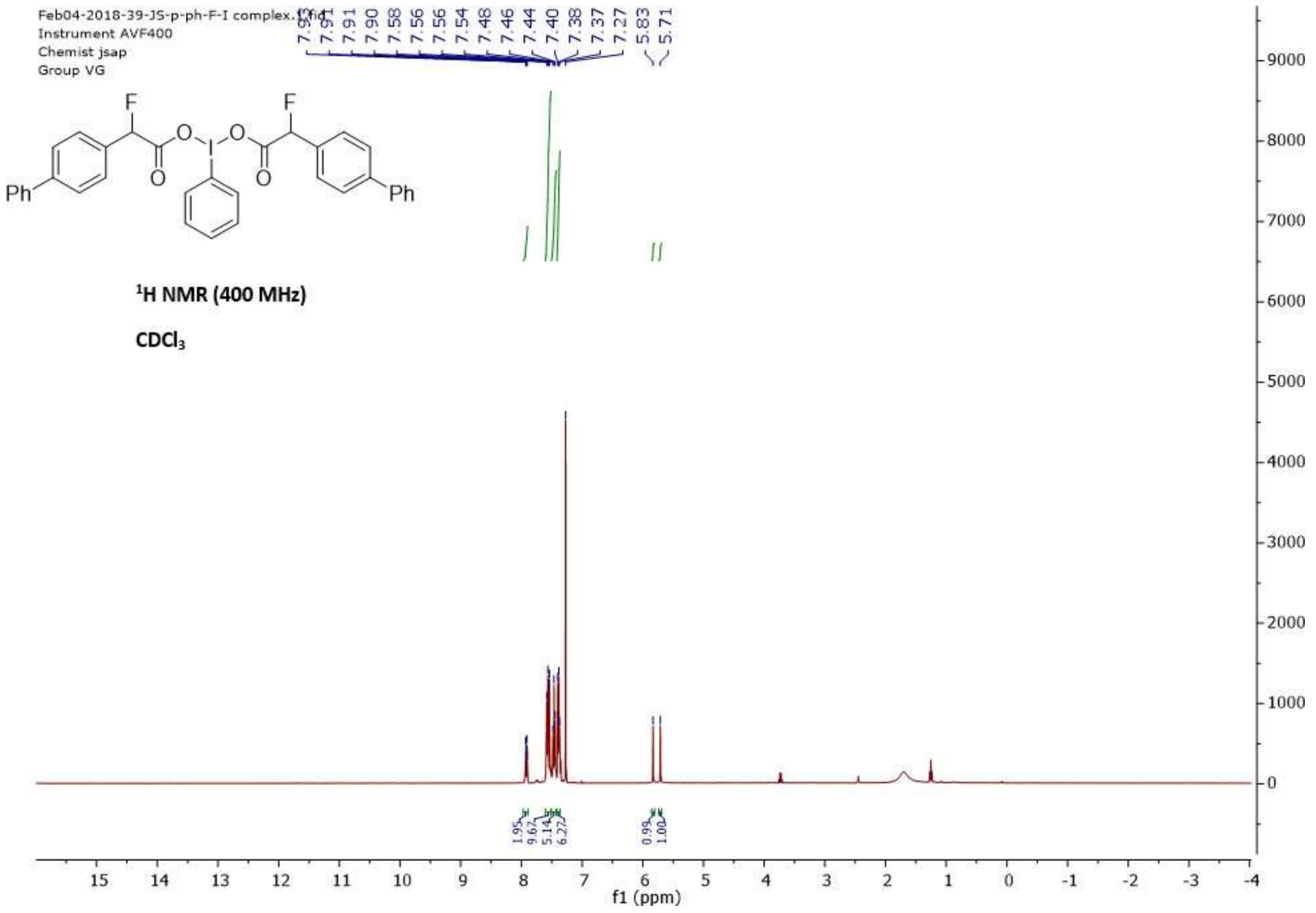
Feb04-2018-39-JS-p-ph-F-I complex.1.fid
Instrument AVF400
Chemist jsap
Group VG
7.93
7.91
7.91
7.90
7.58
7.56
7.56
7.54
7.48
7.46
7.44
7.40
7.38
7.37
7.27
5.83
5.71
F
O
I
O
F
O
O
Ph
Ph
¹H NMR (400 MHz)
CDCl₃
1.95
9.67
5.14
6.27
0.99
1.00
f1 (ppm)

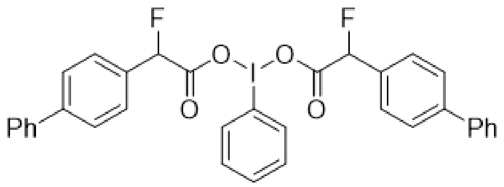

**$^{19}$F NMR (376 MHz)**

**$CDCl_3$**

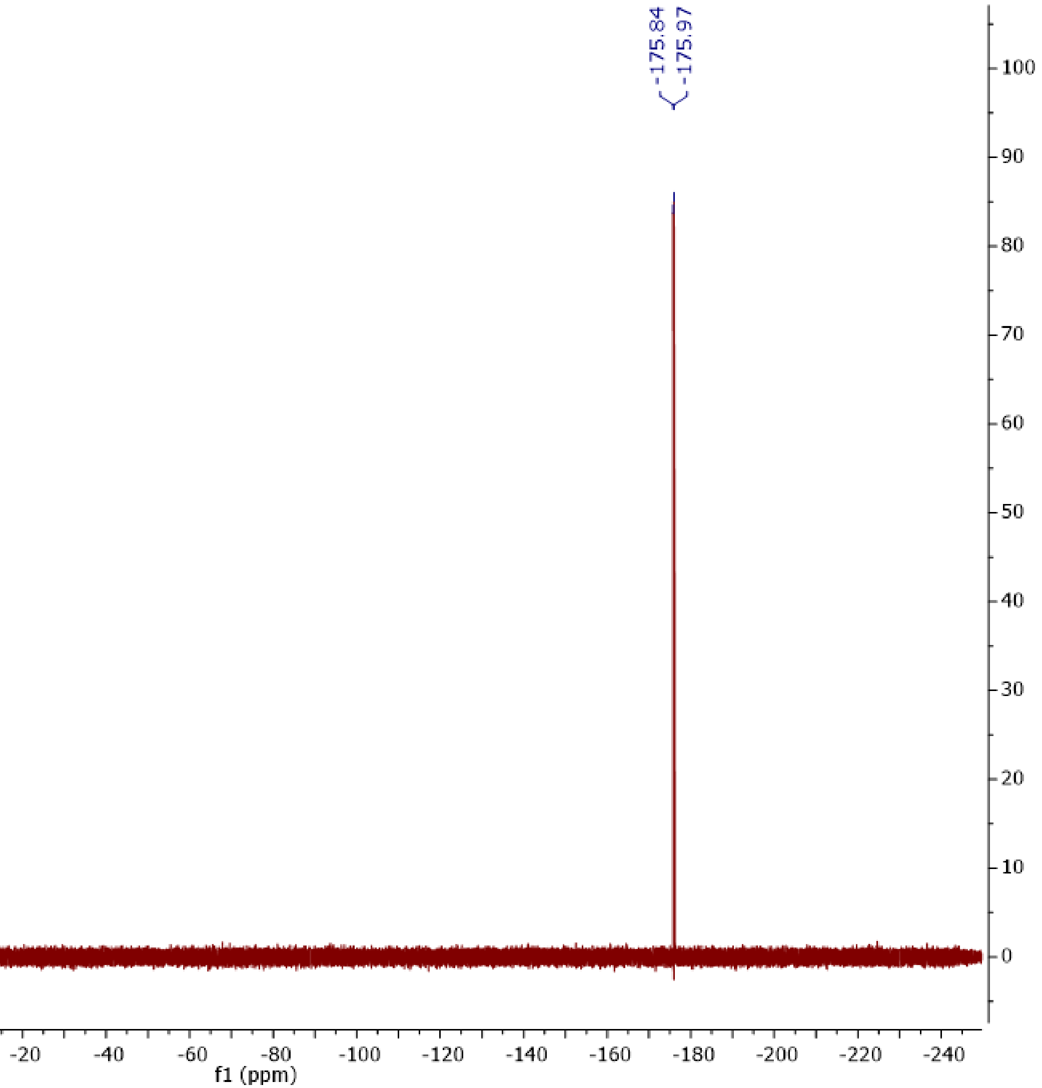

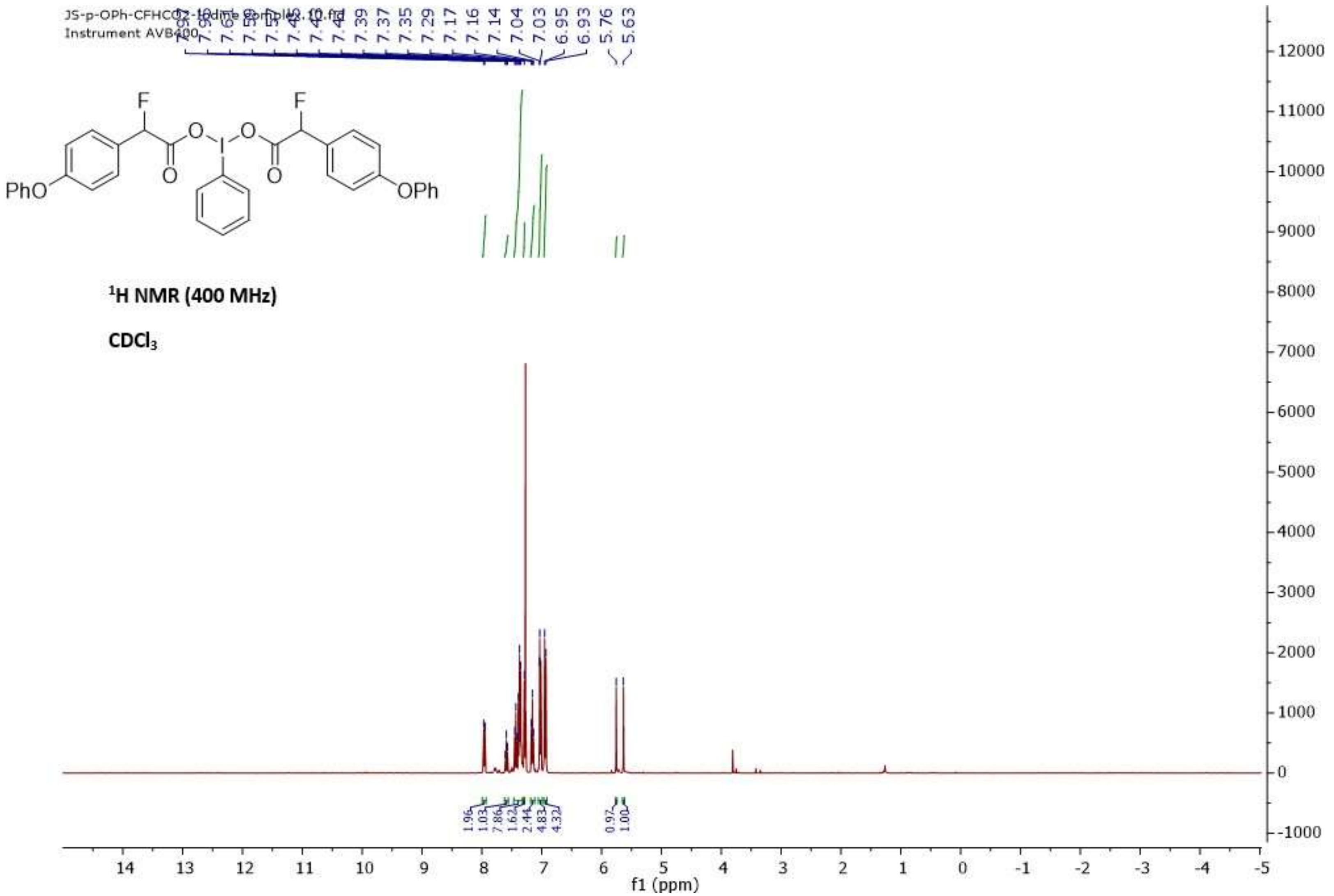
Instrument AVB400
7.39
7.37
7.35
7.29
7.17
7.16
7.14
7.04
7.03
6.95
6.93
5.76
5.63
F
F
O
O
I
O
O
PhO
OPh
¹H NMR (400 MHz)
CDCl3
1.96
1.03
7.86
1.62
2.44
4.83
4.32
0.97
1.00
f1 (ppm)

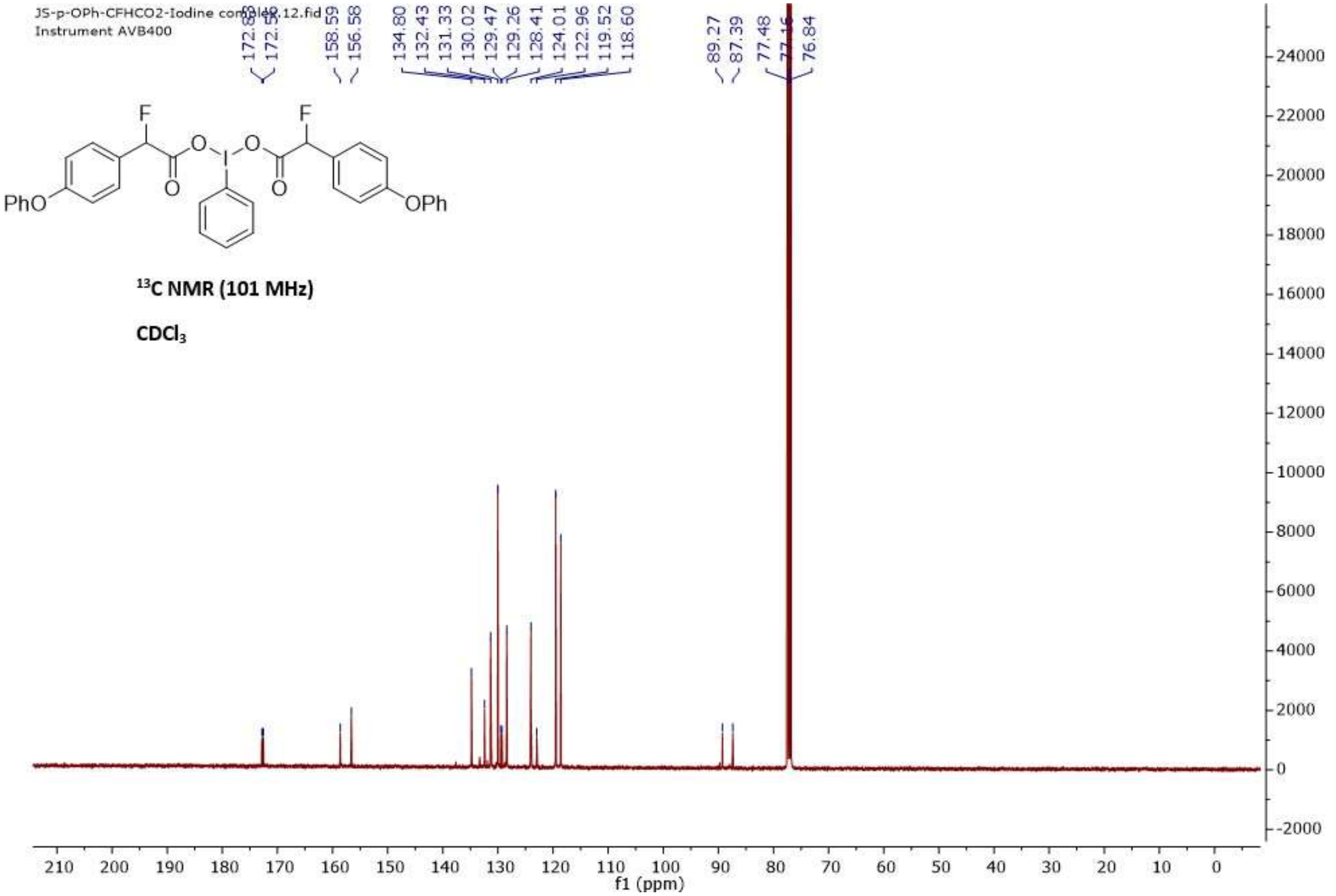

Instrument AVB400
158.59
156.58
134.80
132.43
131.33
130.02
129.47
129.26
128.41
124.01
122.96
119.52
118.60
89.27
87.39
77.48
76.84
F
O
I
PhO
OPh
13C NMR (101 MHz)
CDCl3
f1 (ppm)

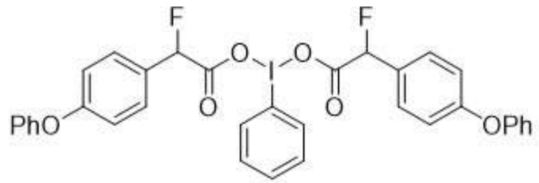


**$^{19}$F NMR (376 MHz)**

**$CDCl_3$**

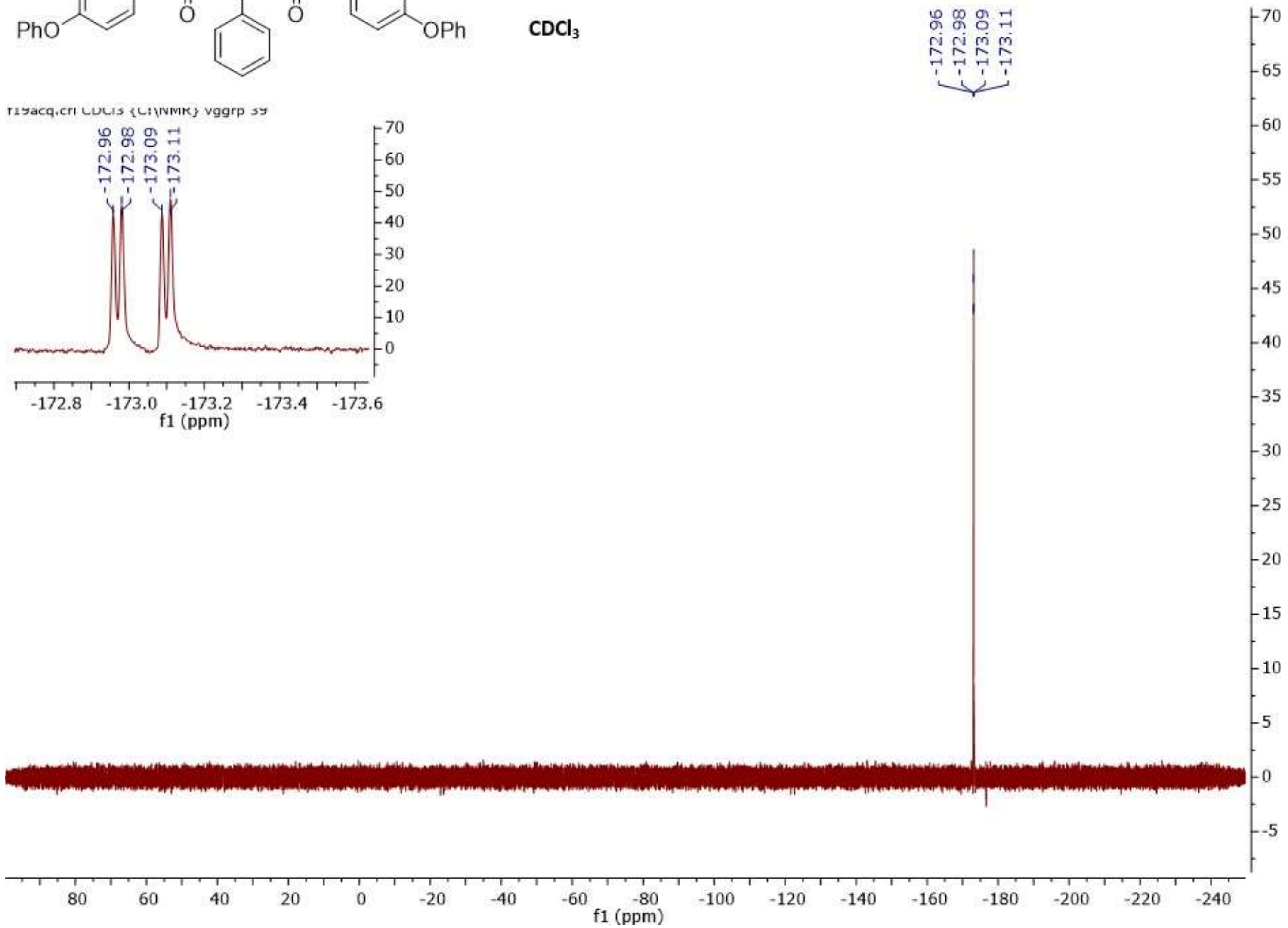

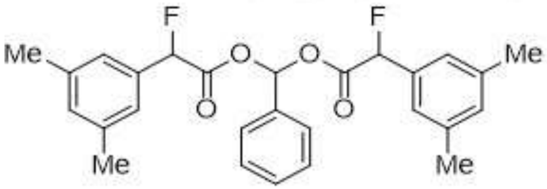


$^{1}$H NMR (400 MHz)

$CDCl_3$

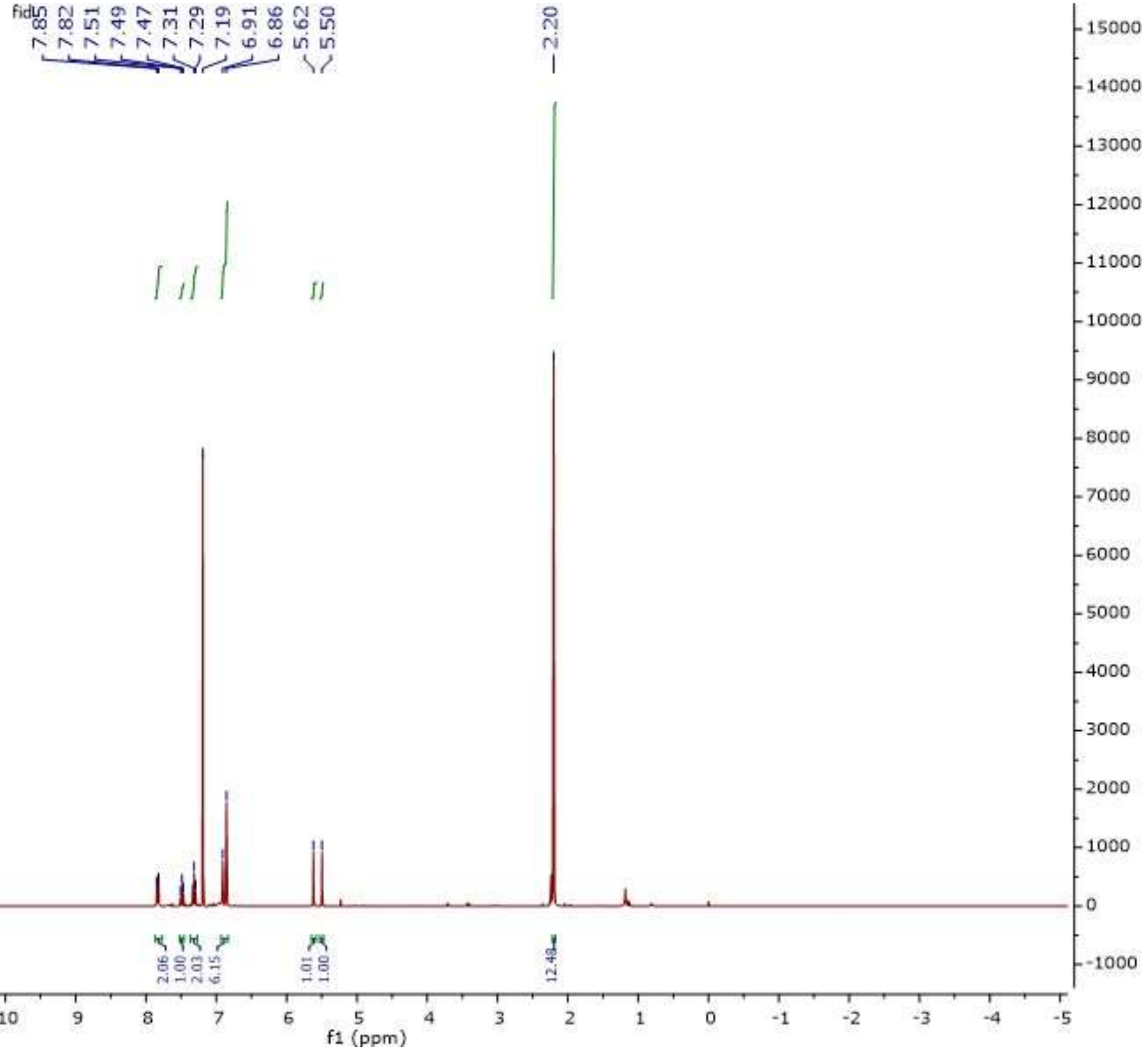

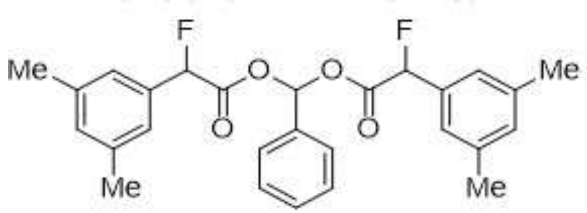


$^{19}$F NMR (376 MHz)

$CDCl_3$

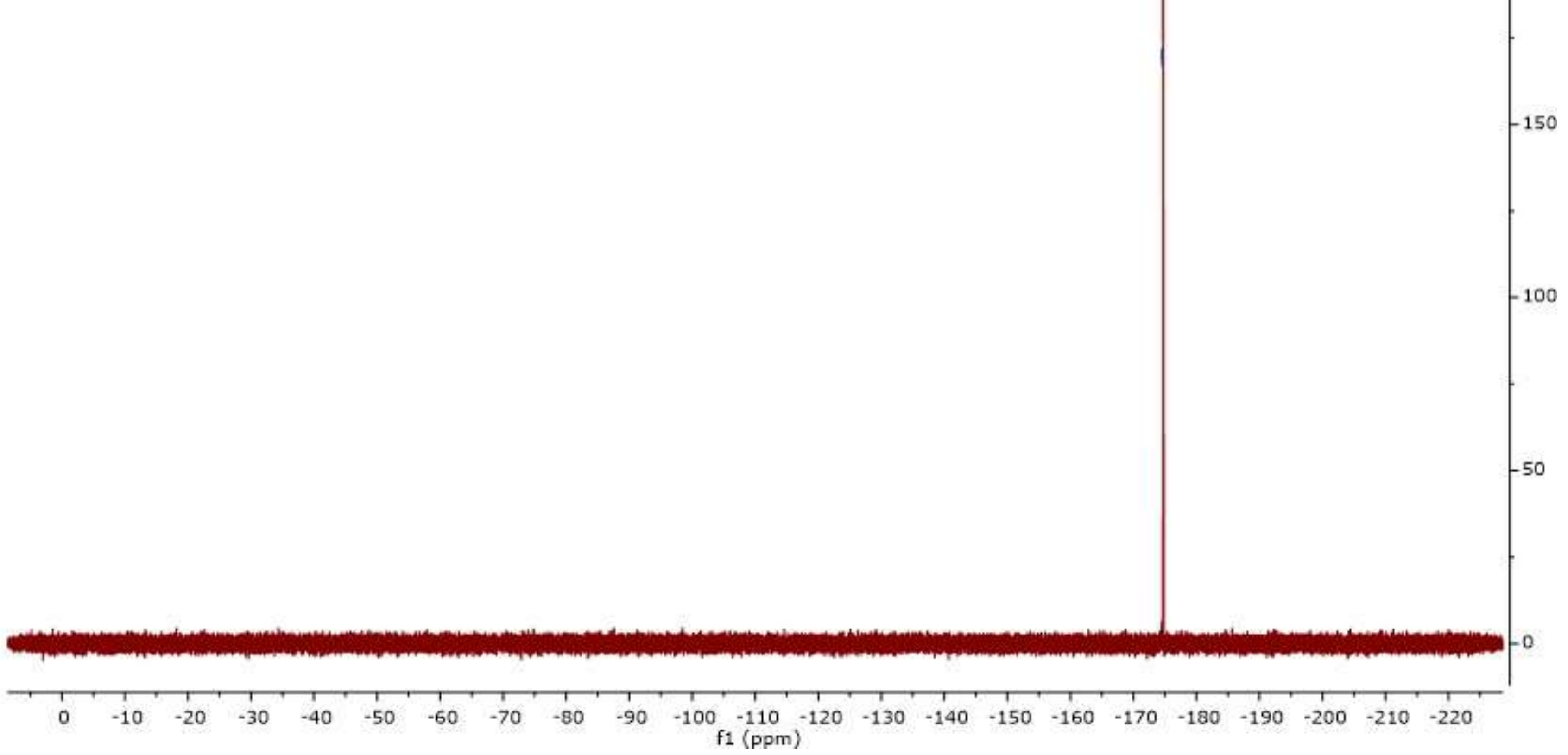

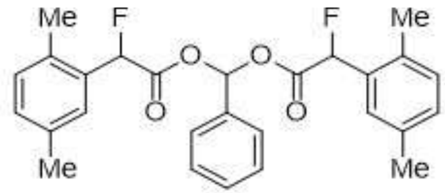


$^{1}H$ NMR (400 MHz)

Acetonitrile-$d_3$

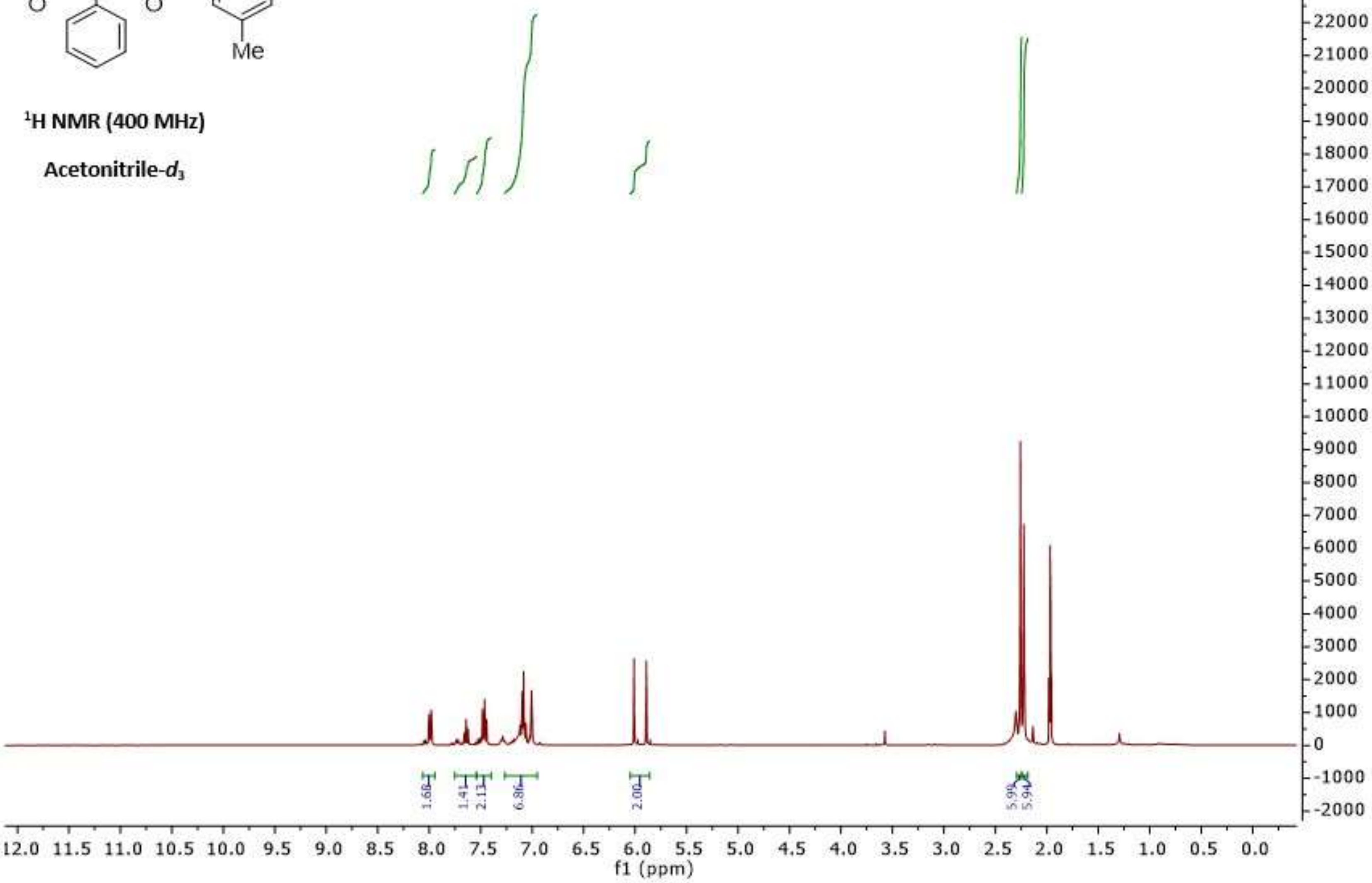

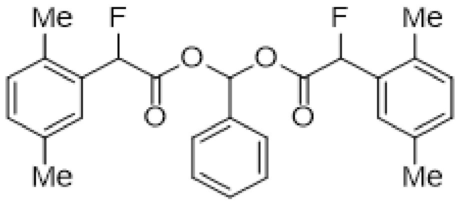

$^{19}$F NMR (376 MHz)

Acetonitrile-$d_3$

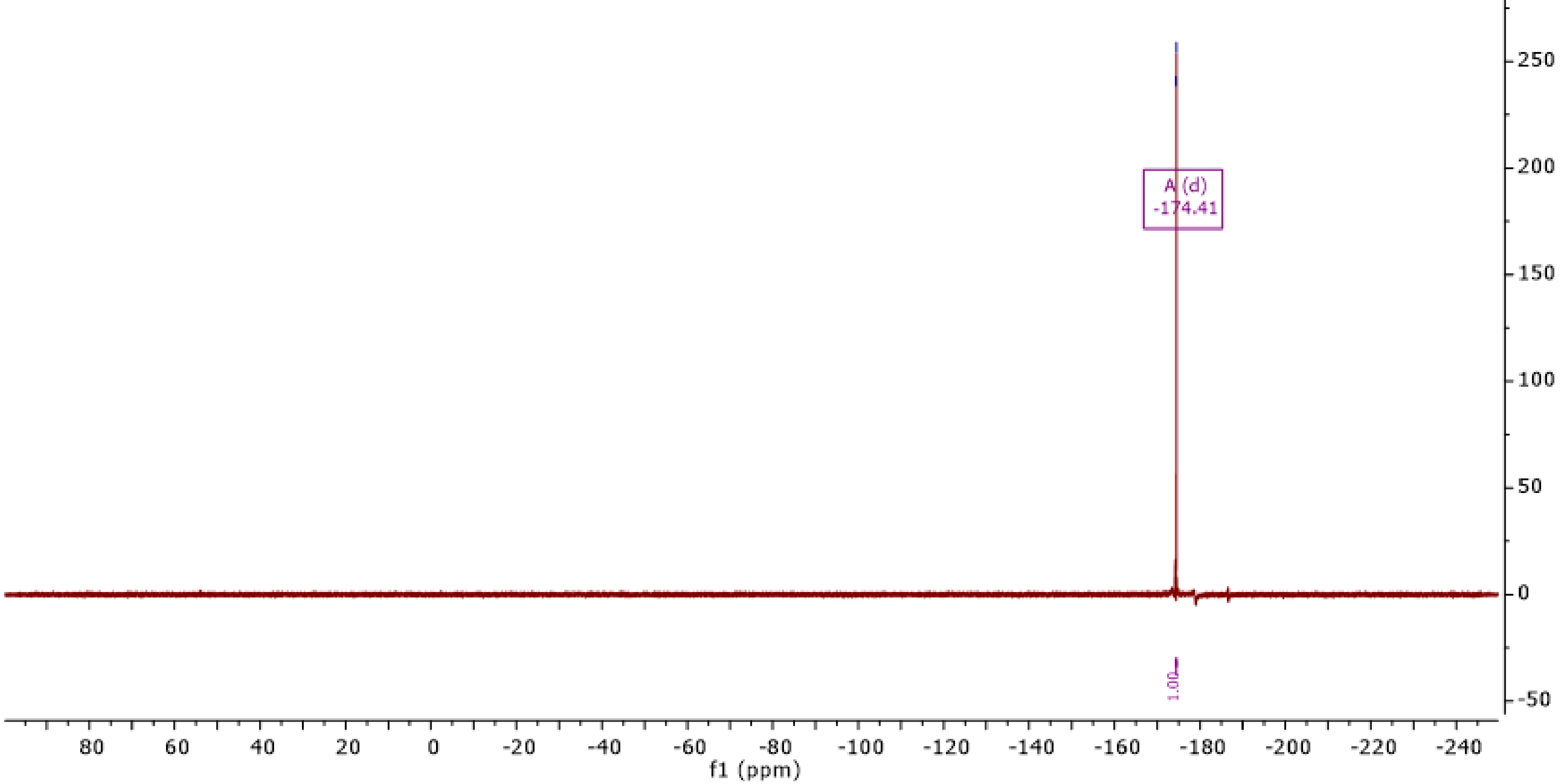

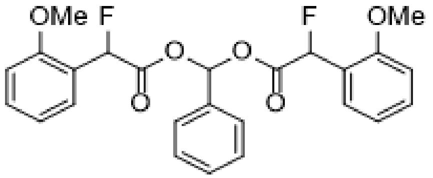


$^1$H NMR (400 MHz)

$CDCl_3$

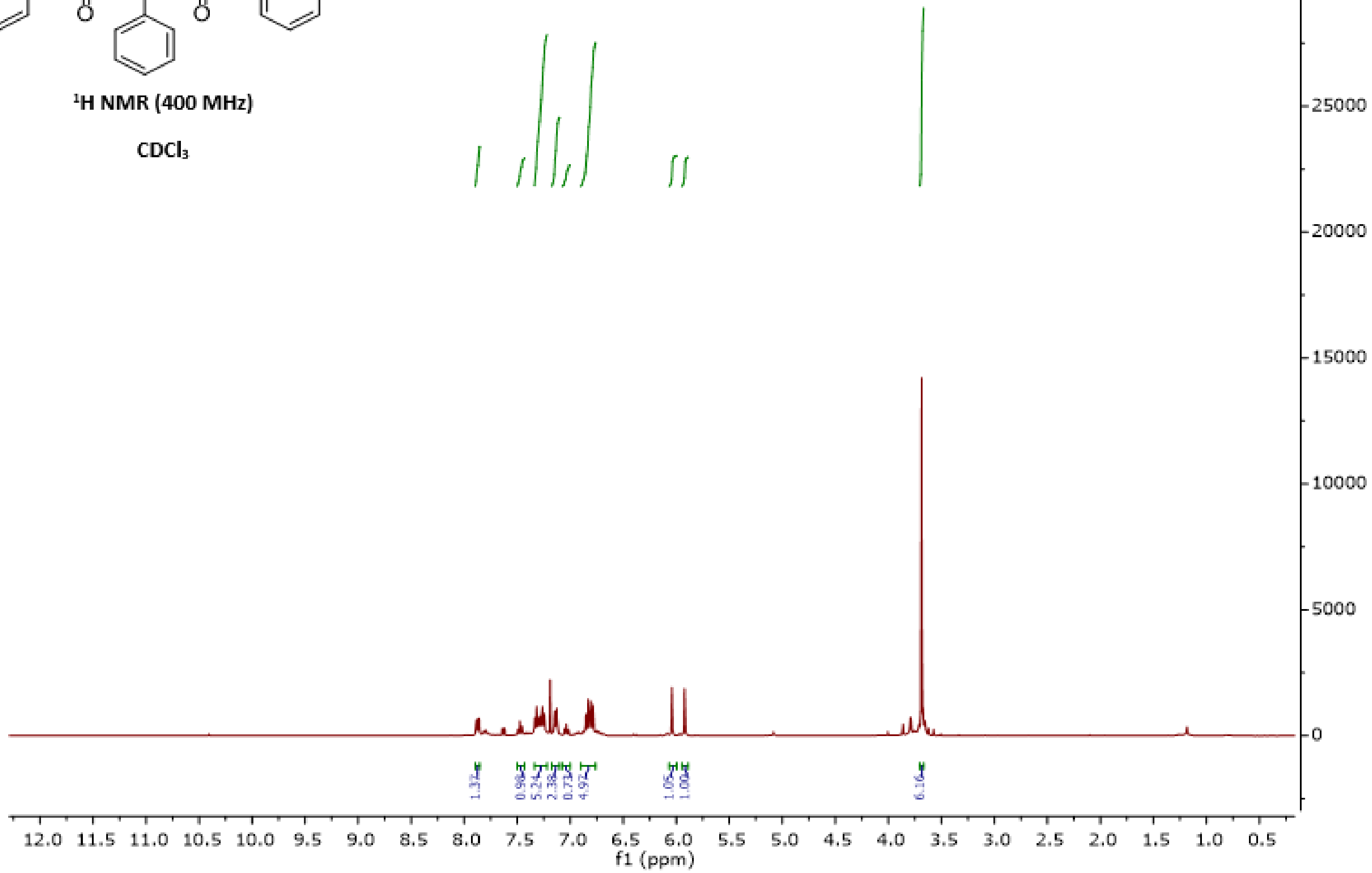

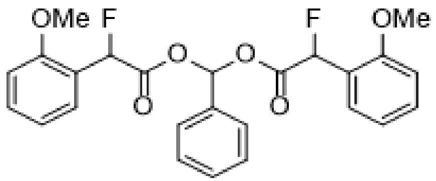

$^{19}$F NMR (376 MHz)

$CDCl_3$

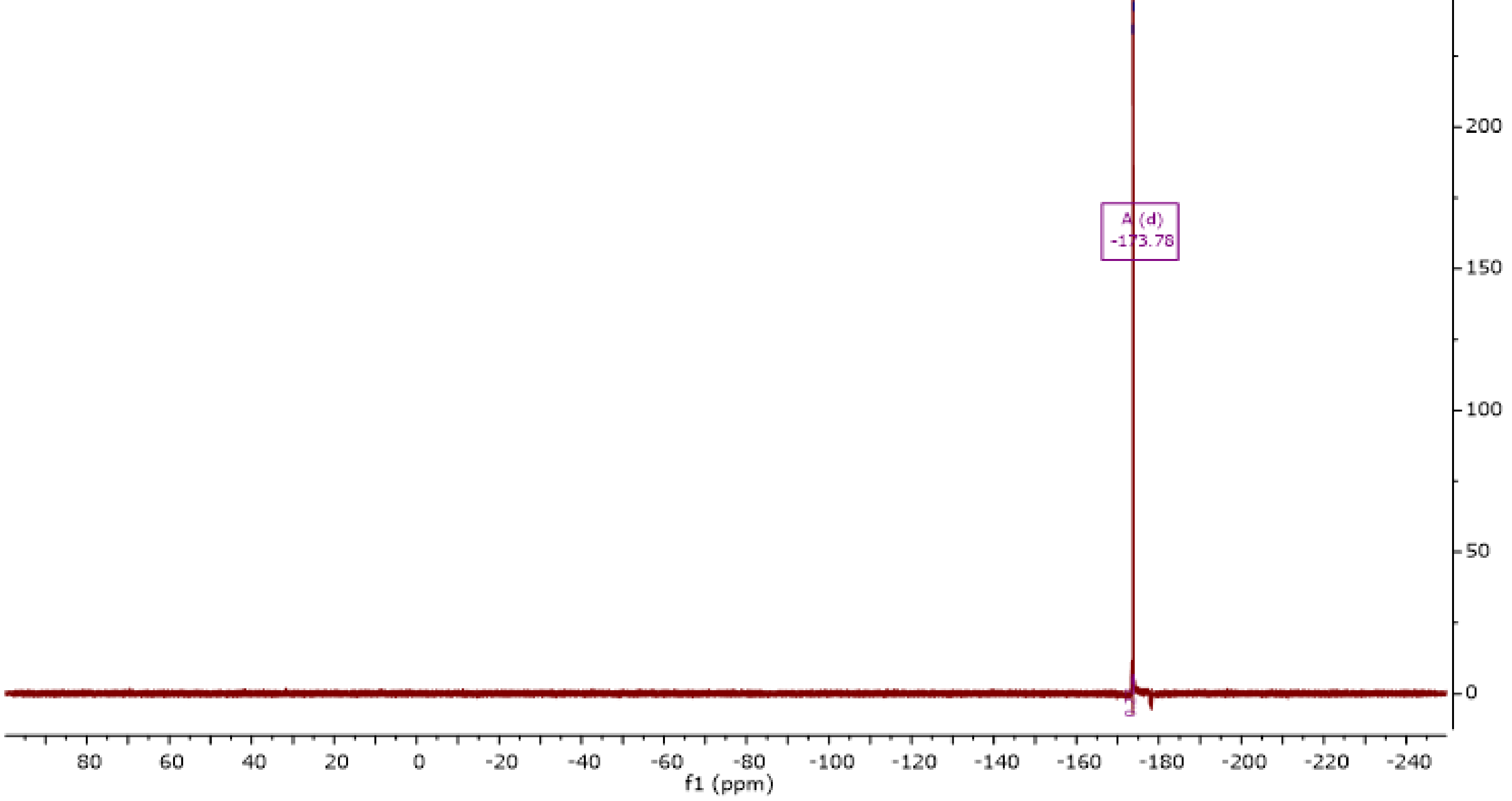

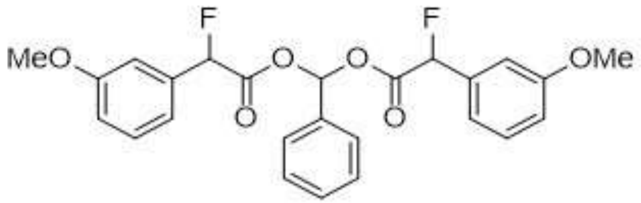


$^{1}$H NMR (400 MHz)

$CDCl_3$

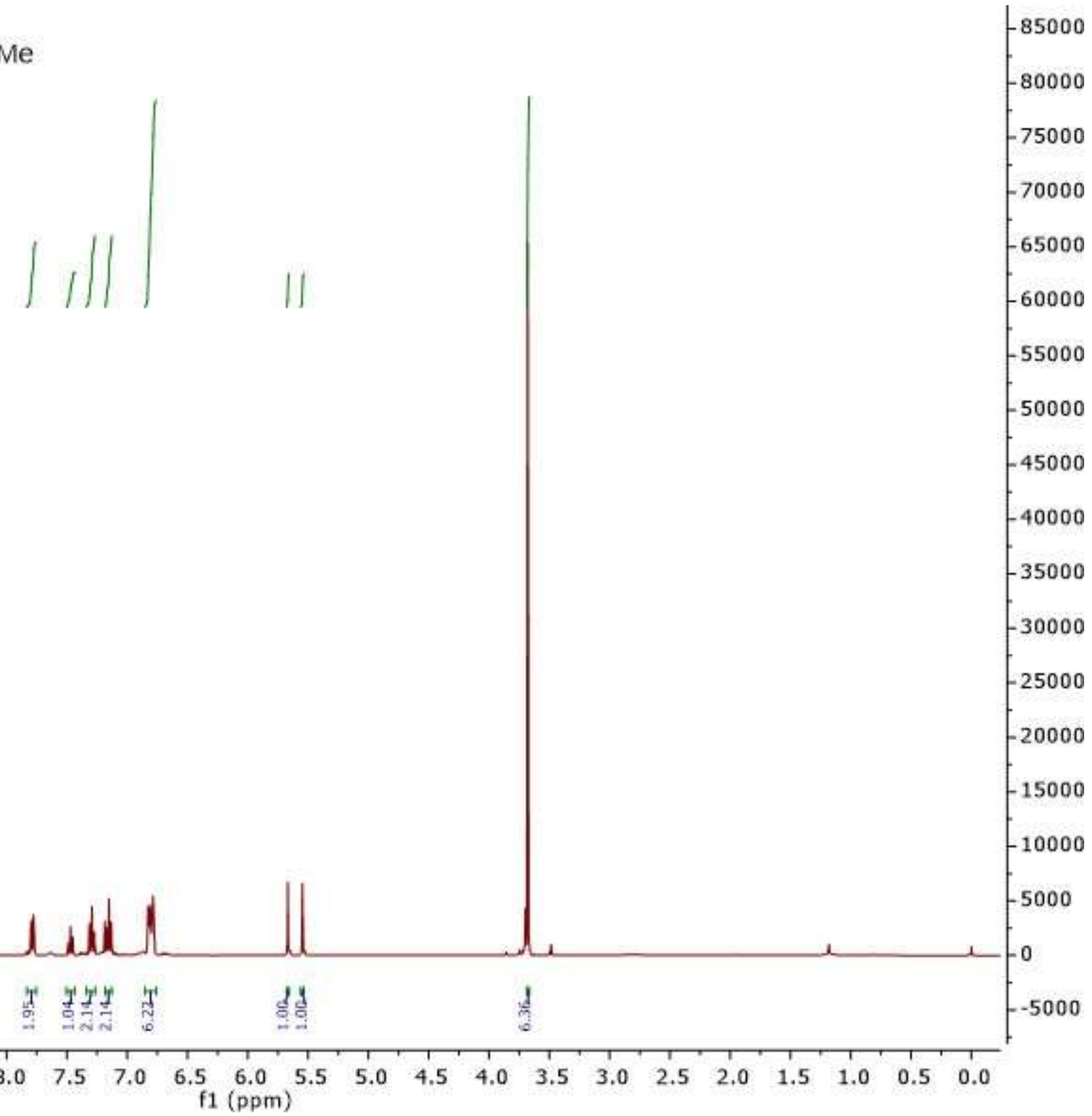

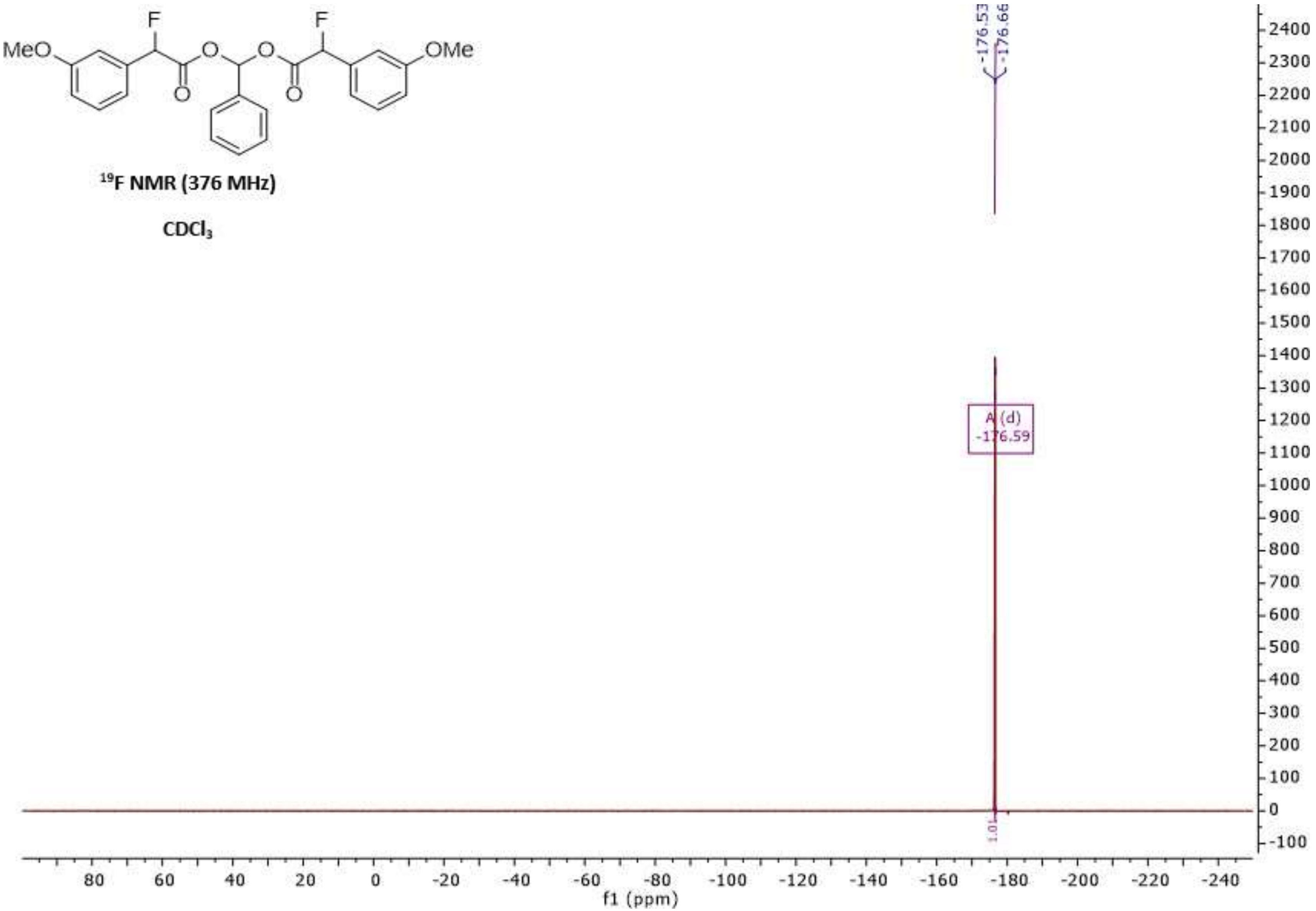


**$^{19}$F NMR (376 MHz)**

**$CDCl_3$**

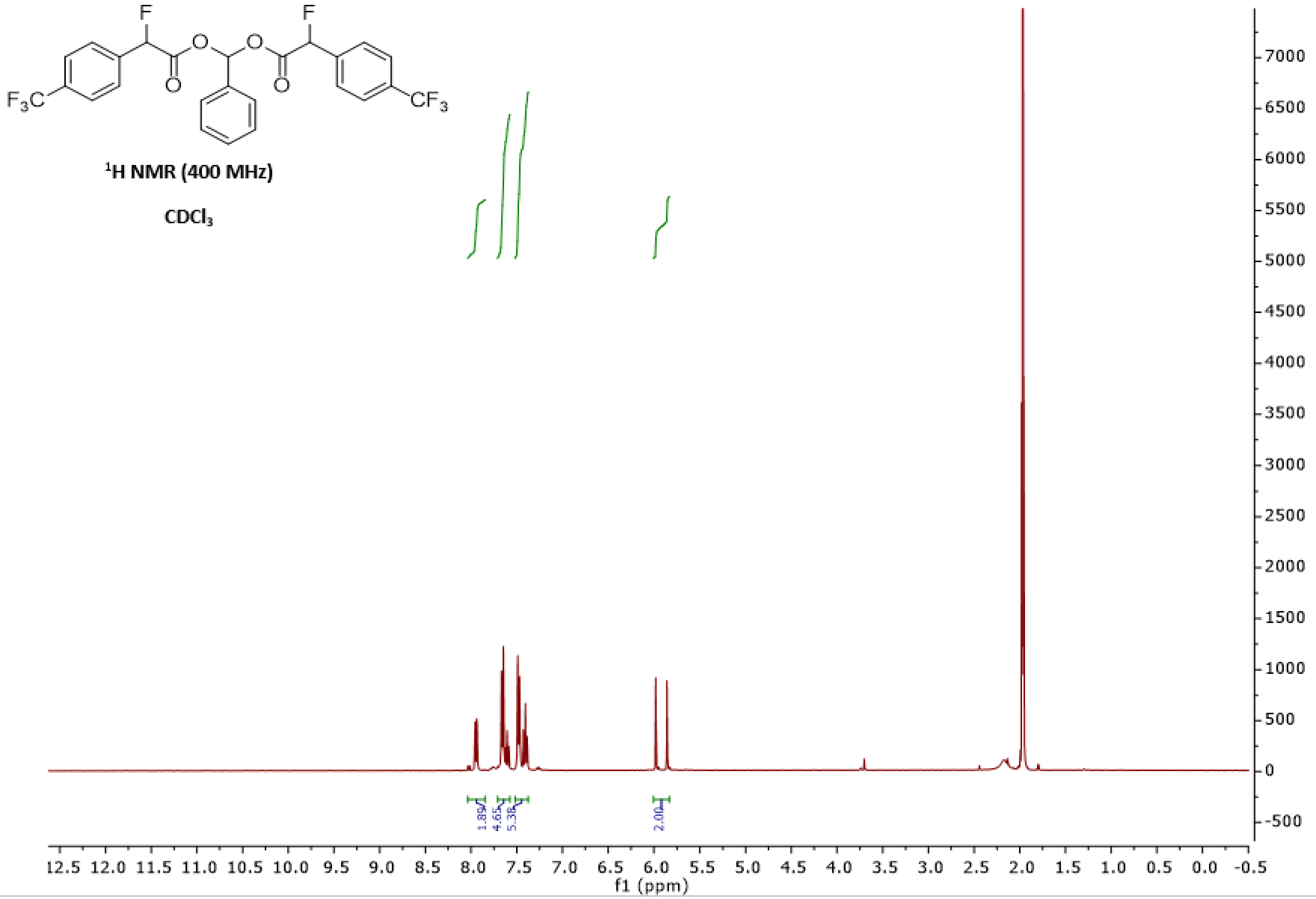

F
F
O
O
O
O
$F_3C$
$CF_3$
$^1$H NMR (400 MHz)
CDCl$_3$
1.89
4.65
5.38
2.00
12.5 12.0 11.5 11.0 10.5 10.0 9.5 9.0 8.5 8.0 7.5 7.0 6.5 6.0 5.5 5.0 4.5 4.0 3.5 3.0 2.5 2.0 1.5 1.0 0.5 0.0 -0.5
f1 (ppm)
7000 6500 6000 5500 5000 4500 4000 3500 3000 2500 2000 1500 1000 500 0 -500

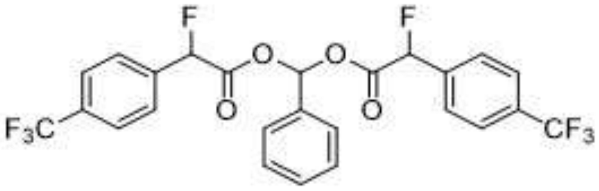

$^1$H NMR (400 MHz)

$CDCl_3$

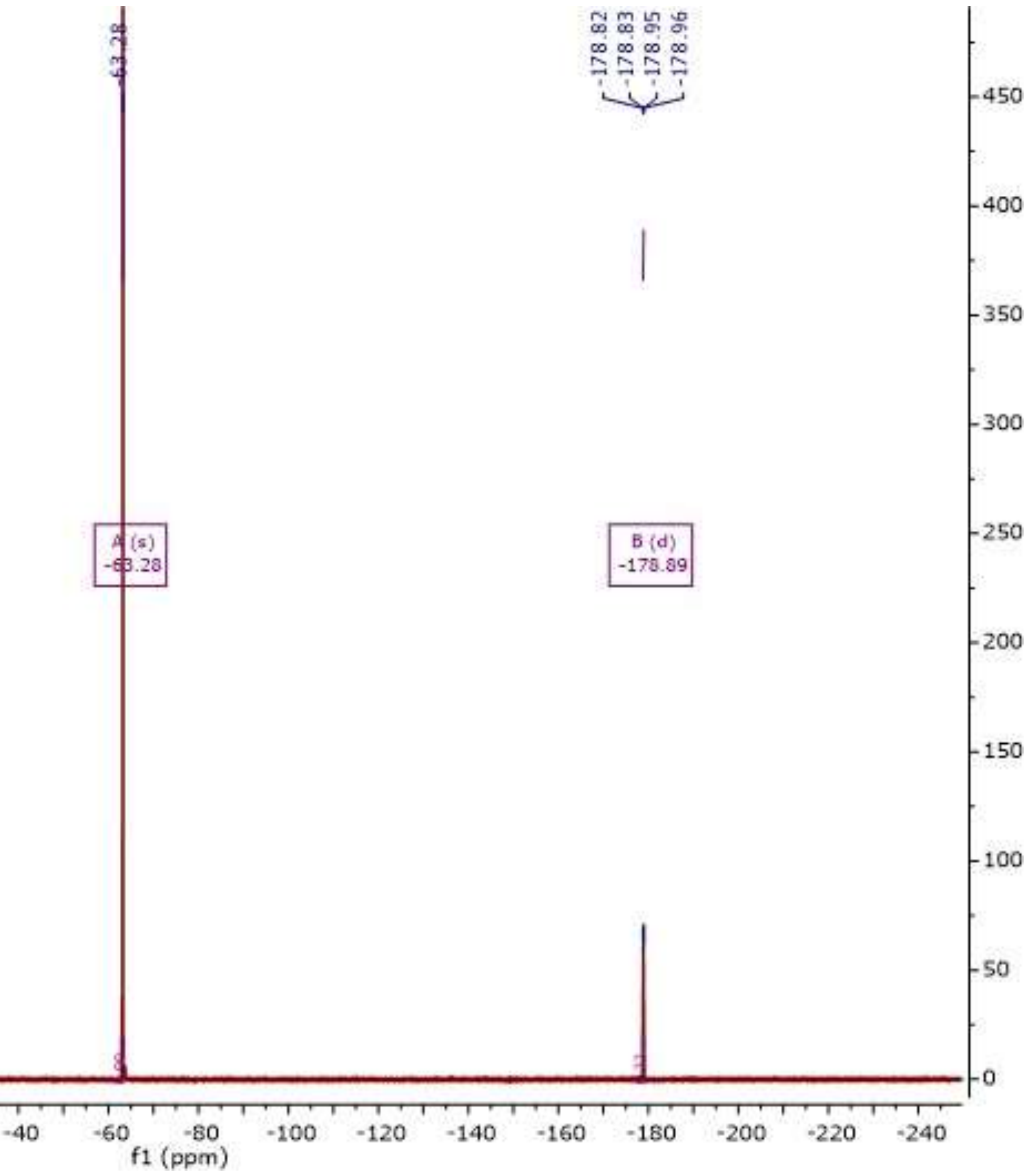

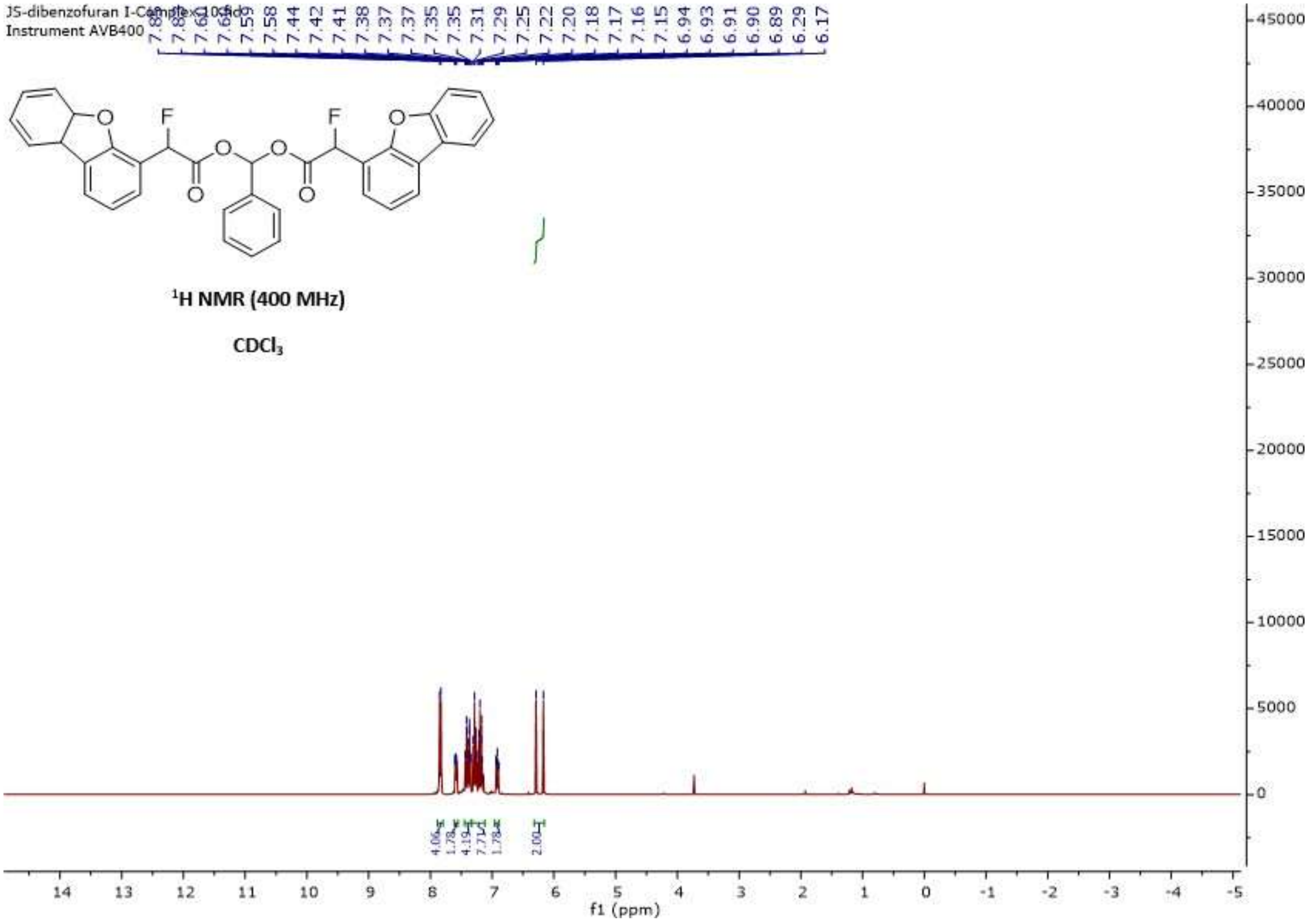
Instrument AVB400
7.59
7.58
7.44
7.42
7.41
7.38
7.37
7.37
7.35
7.35
7.31
7.29
7.25
7.22
7.20
7.18
7.17
7.16
7.15
6.94
6.93
6.91
6.90
6.89
6.29
6.17
F
O
F
O
O
O
O
O
$^1$H NMR (400 MHz)
$CDCl_3$
4.06
1.78
4.19
7.71
1.78
2.00
f1 (ppm)

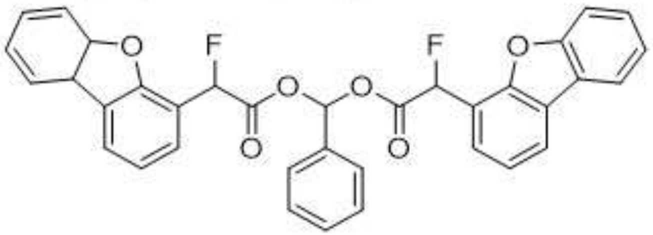


$^{19}$F NMR (376 MHz)

$CDCl_3$

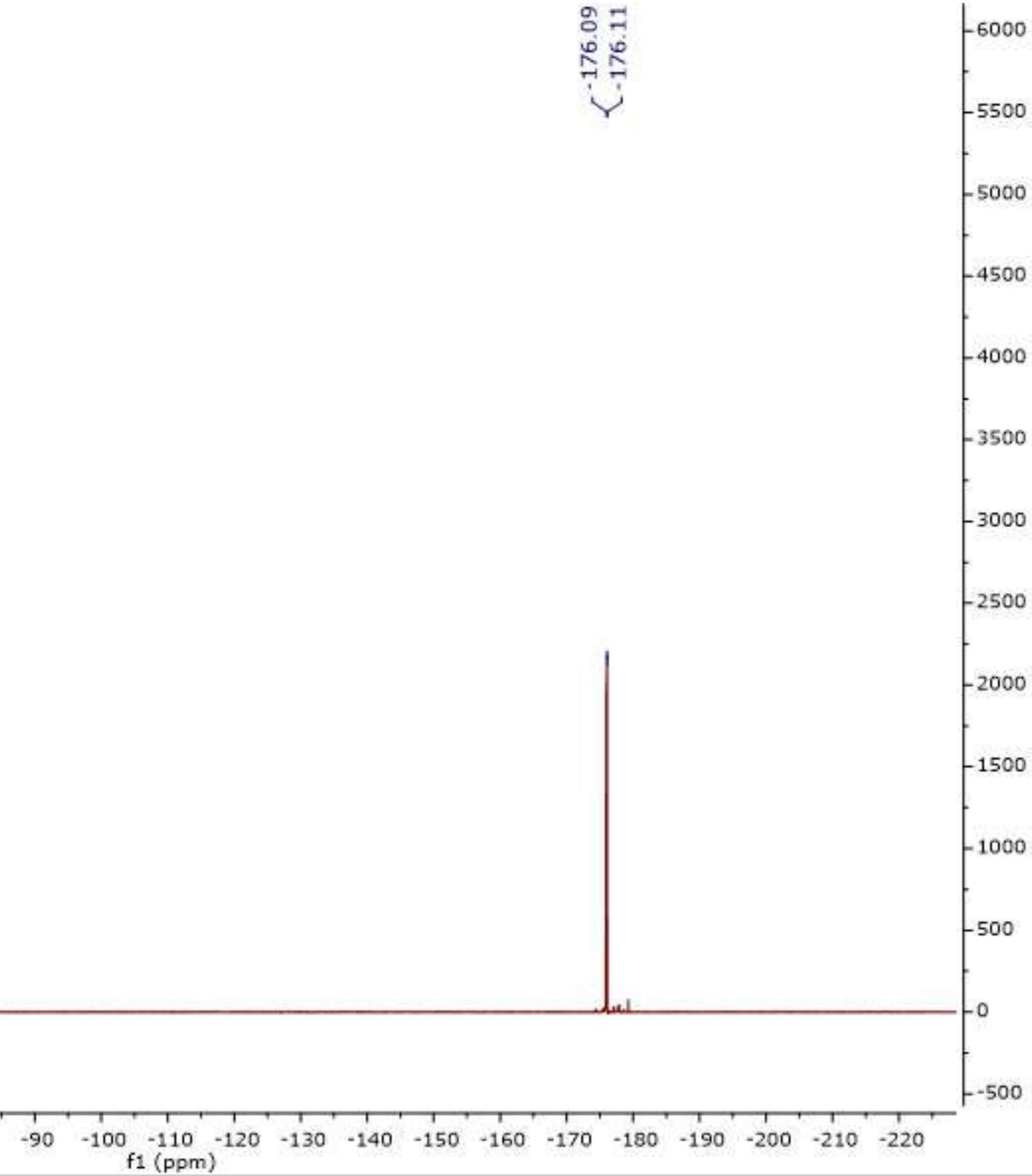

Apr23-2018-1-JS-CF3-Het-I complex.1.fid

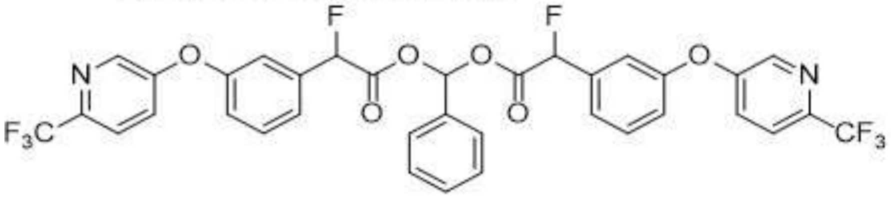

$^1$H NMR (400 MHz)

$CDCl_3$

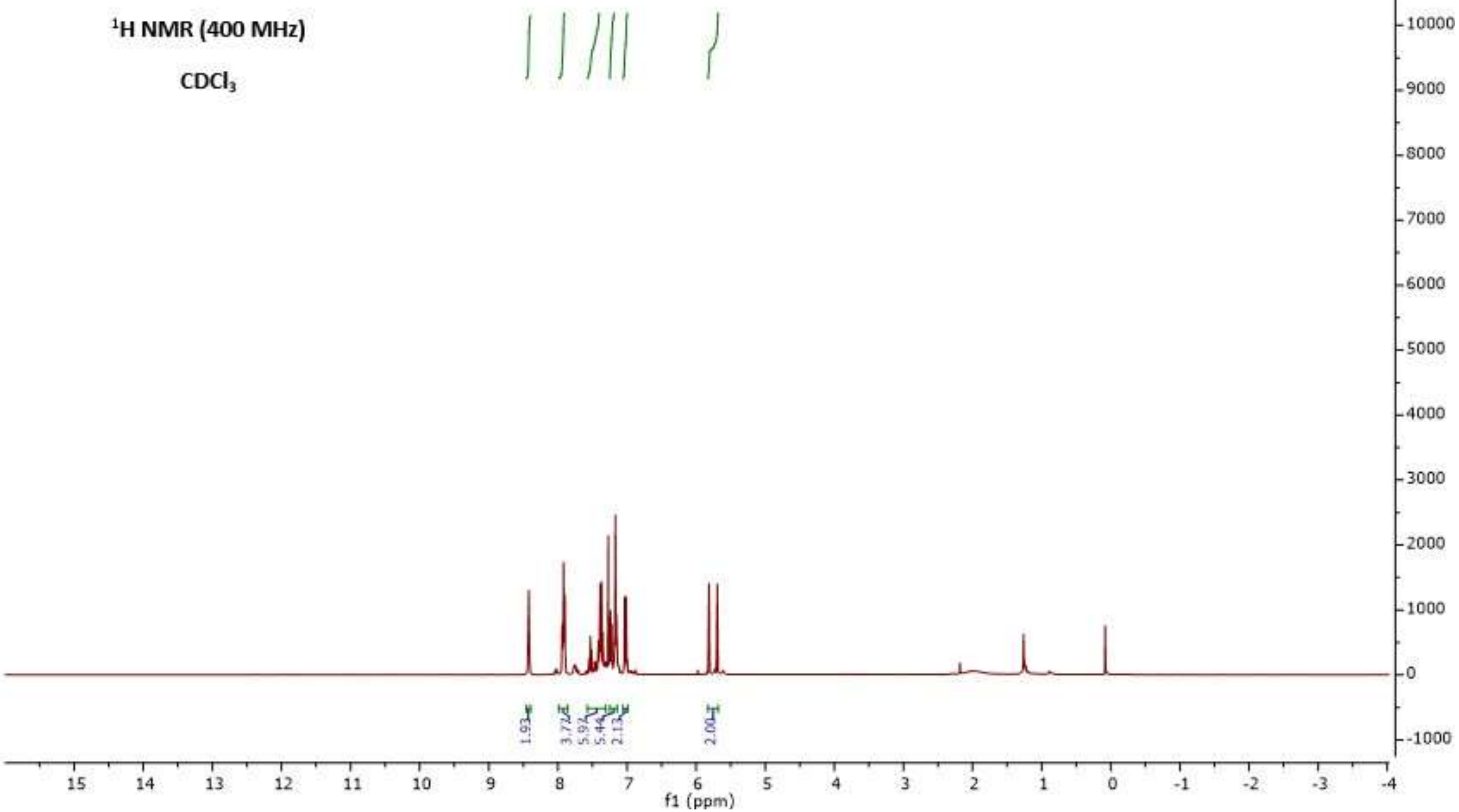

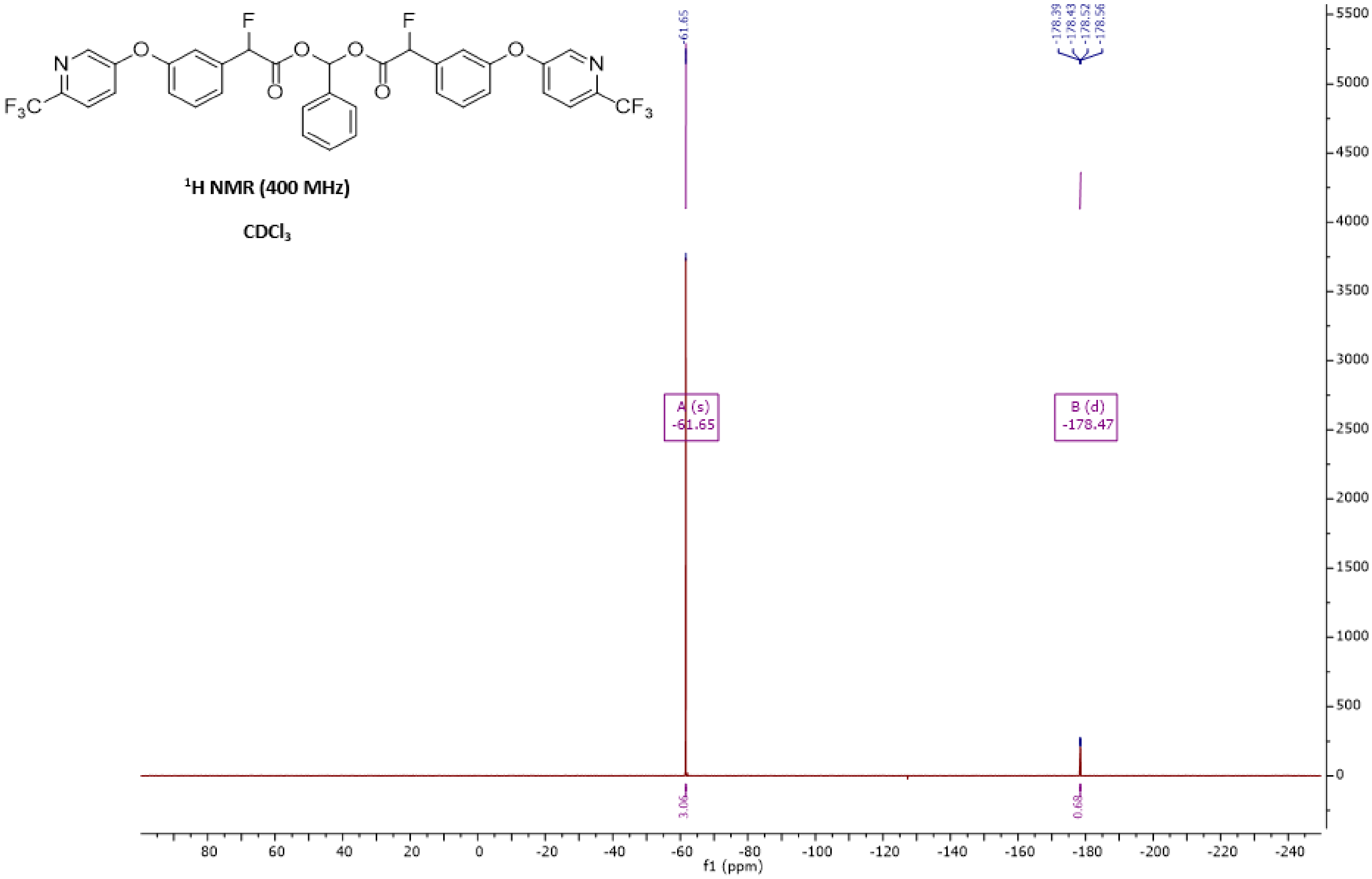

F
F
N
N
O
O
O
O
O
O
$F_3C$
$CF_3$
¹H NMR (400 MHz)
$CDCl_3$
-61.65
-178.39
-178.43
-178.52
-178.56
A (s)
-61.65
B (d)
-178.47
3.06
0.68
f1 (ppm)

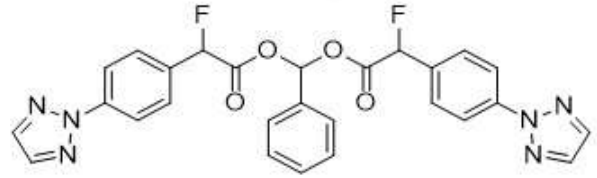

$^1H$ NMR (400 MHz)

$CDCl_3$

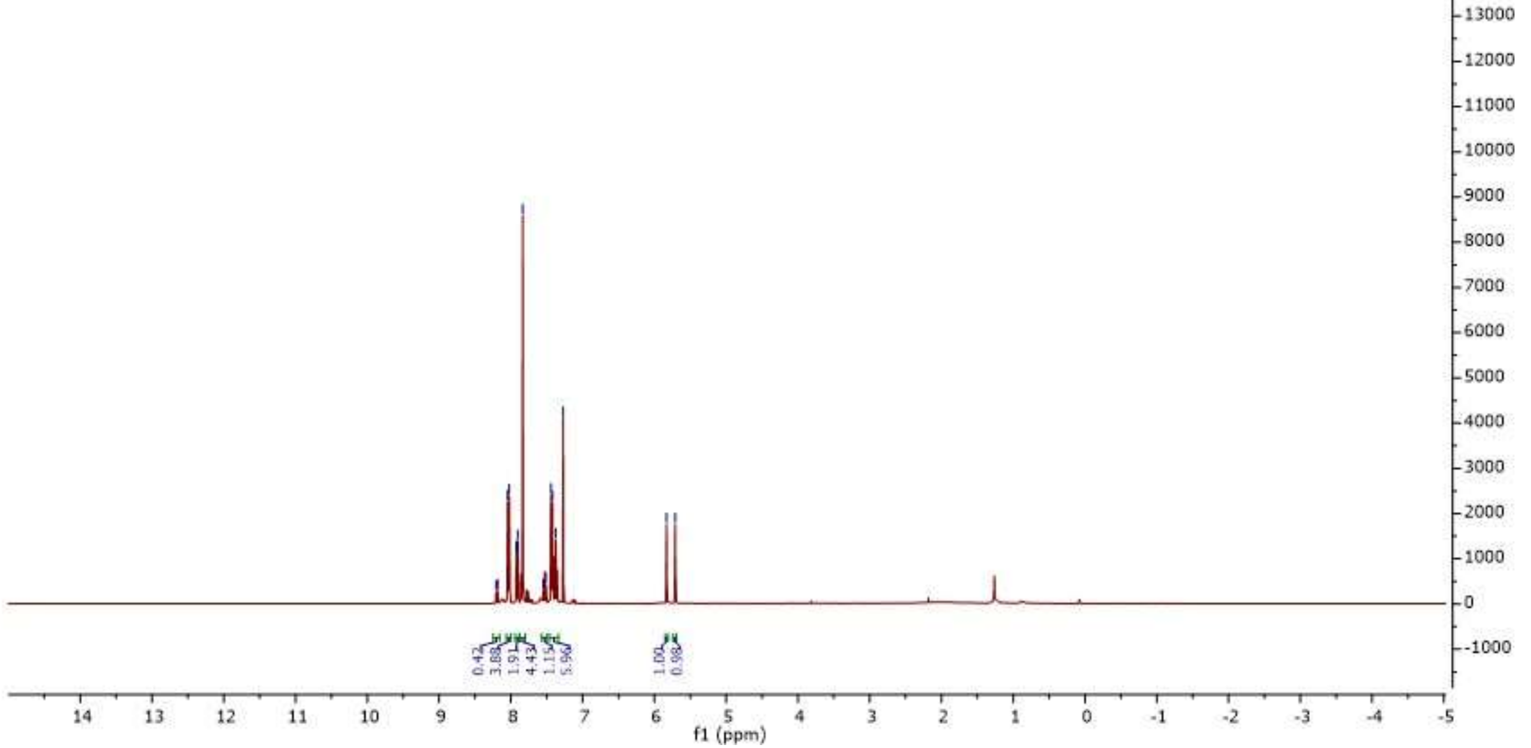

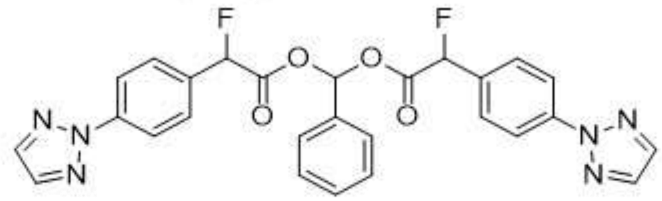

$^{19}$F NMR (376 MHz)

$CDCl_3$

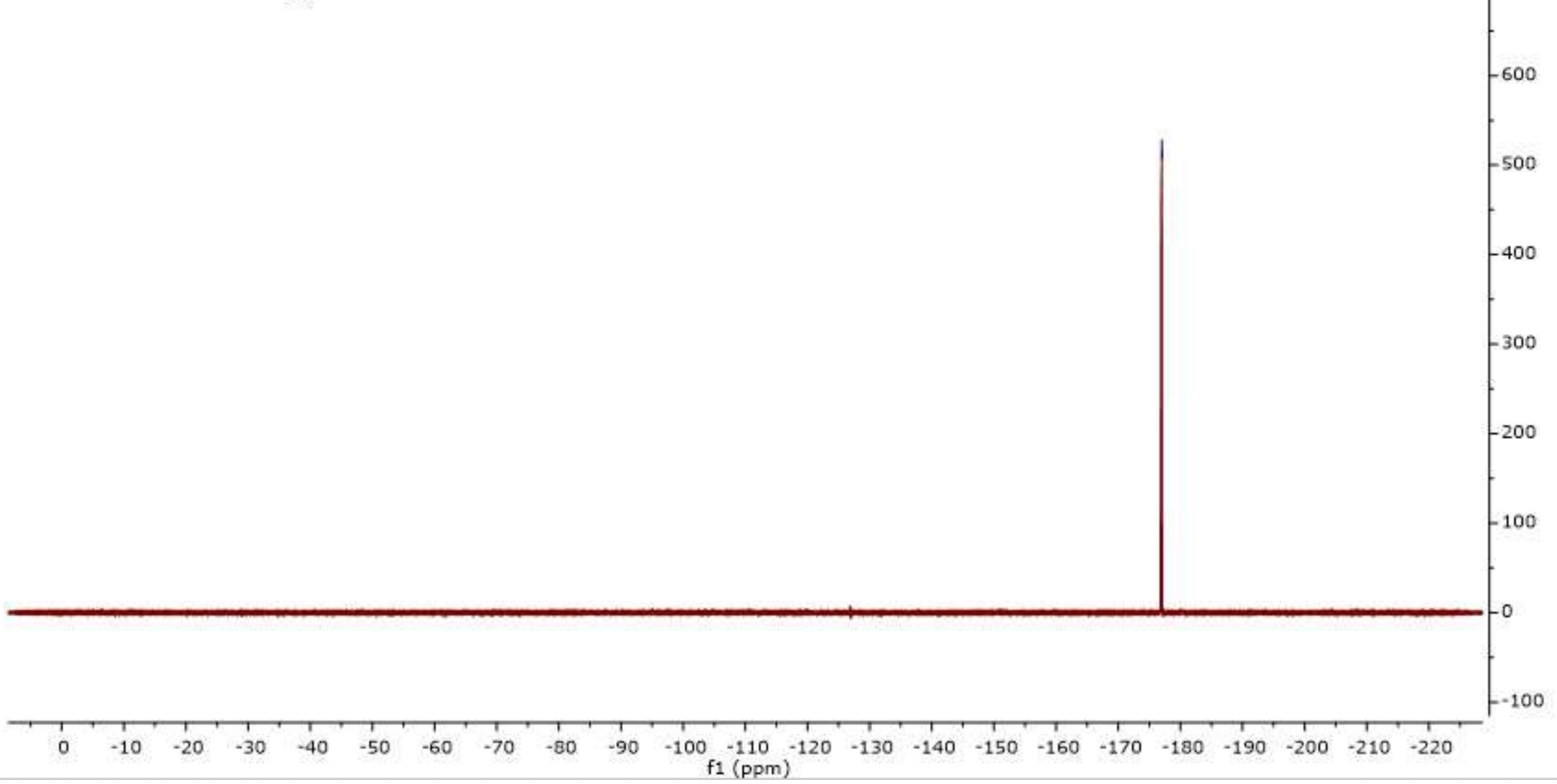

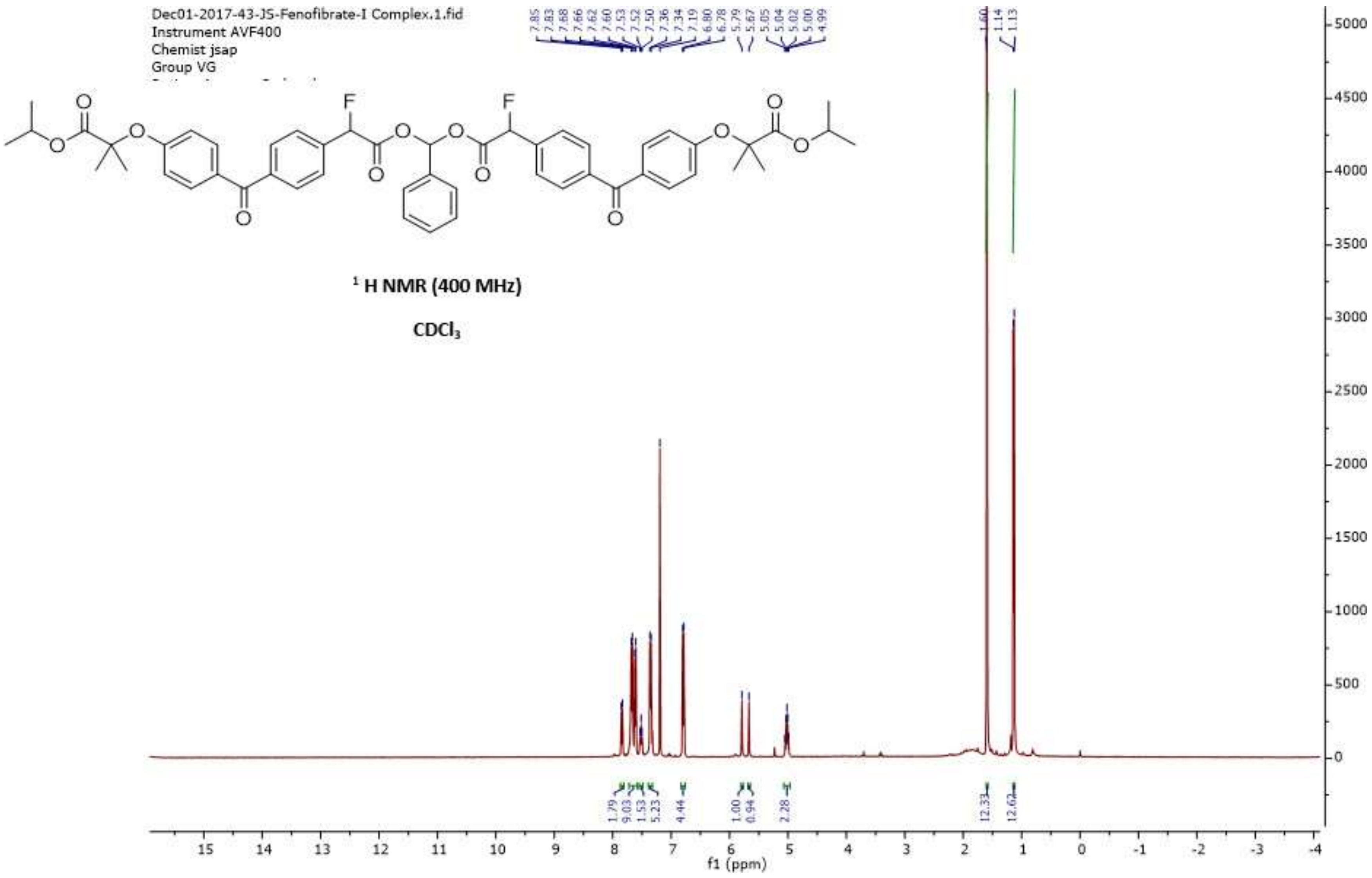

Dec01-2017-43-JS-Fenofibrate-I Complex.1.fid
Instrument AVF400
Chemist jsap
Group VG
¹ H NMR (400 MHz)
CDCl₃
7.85 7.83 7.68 7.66 7.62 7.60 7.53 7.52 7.50 7.36 7.34 7.19 6.80 6.78 5.79 5.67 5.05 5.04 5.02 5.00 4.99
1.60 1.14 1.13
1.79 9.03 1.53 5.23 4.44 1.00 0.94 2.28 12.33 12.62
f1 (ppm)

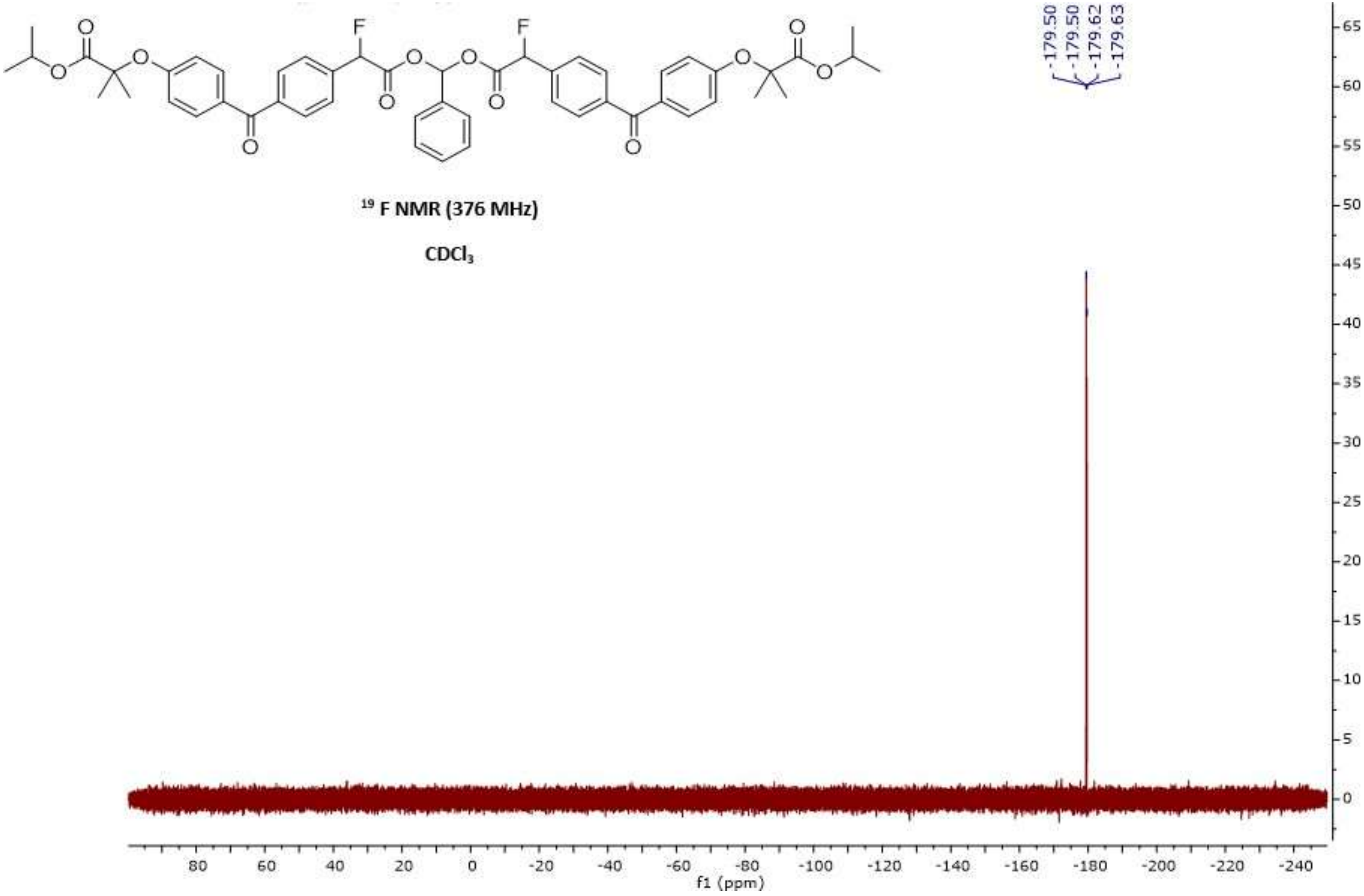


[19] F NMR (376 MHz)

$CDCl_3$

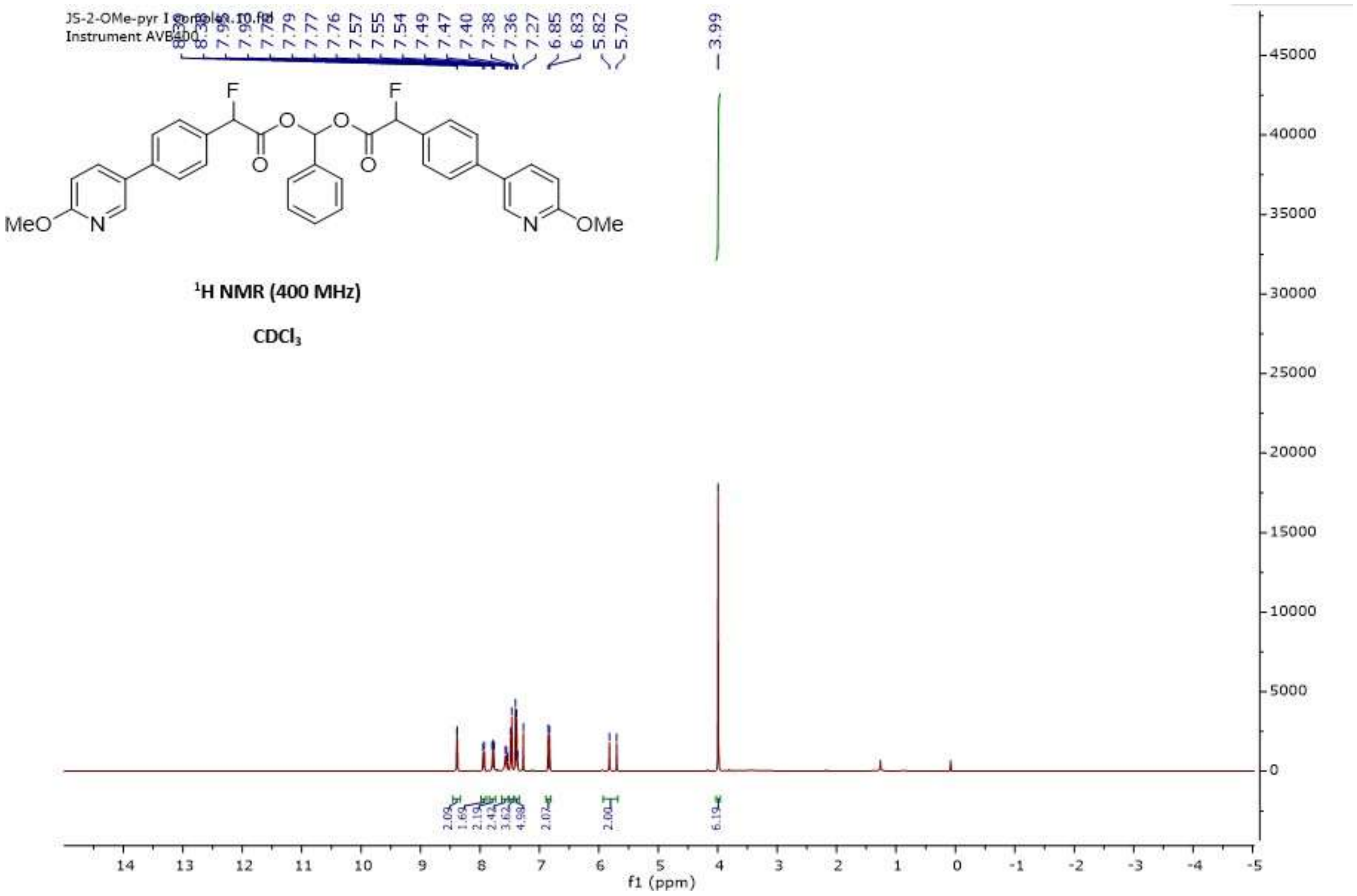
Instrument AVB400
7.79
7.77
7.76
7.57
7.55
7.54
7.49
7.47
7.40
7.38
7.36
7.27
6.85
6.83
5.82
5.70
3.99
F
F
O
O
O
O
MeO
N
N
OMe
¹H NMR (400 MHz)
CDCl₃
2.09
1.69
2.19
2.42
3.62
4.98
2.07
2.00
6.19
f1 (ppm)

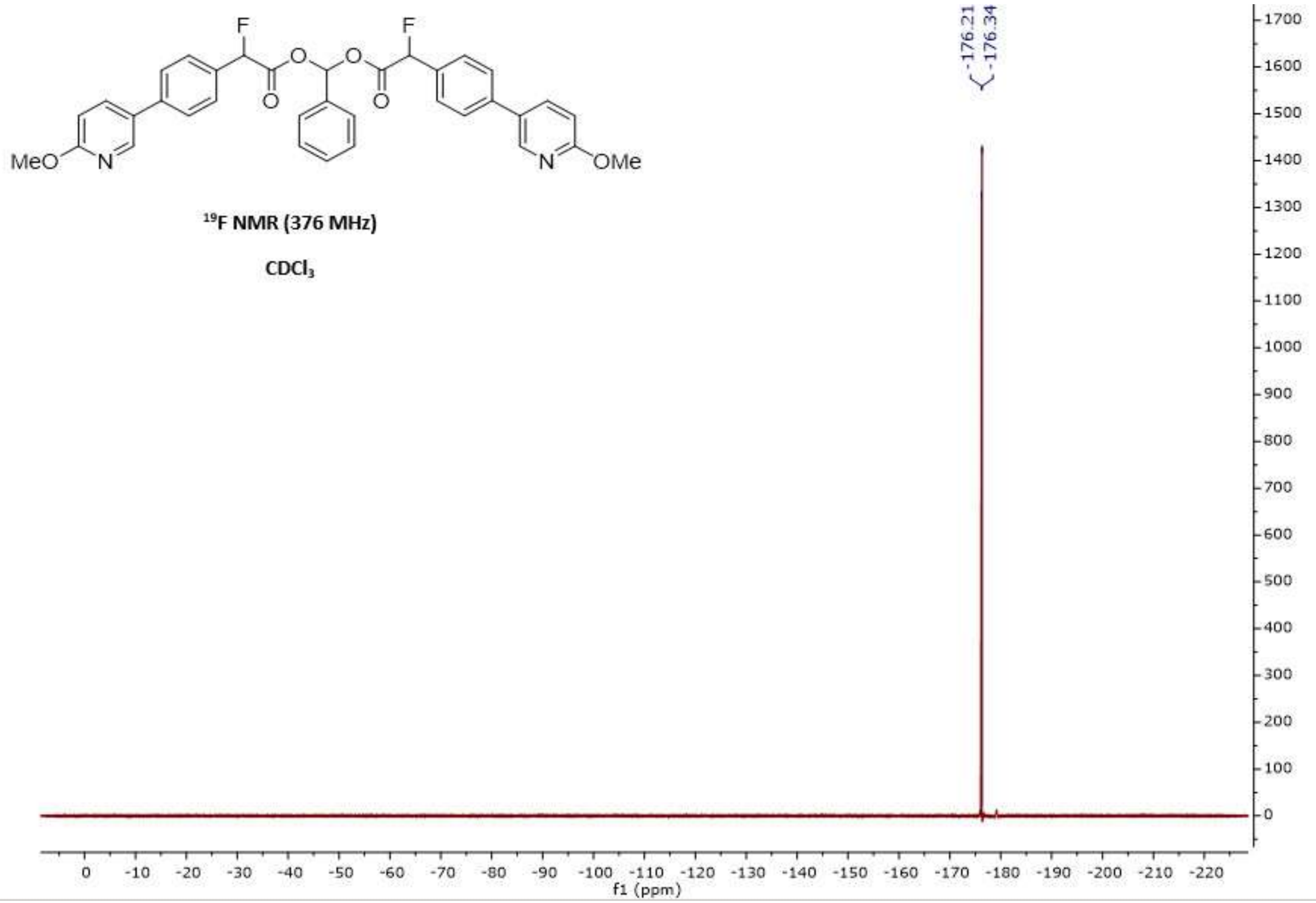

F
F
MeO
N
N
OMe
O
O
O
O
19F NMR (376 MHz)
CDCl3
-176.21
-176.34
1700
1600
1500
1400
1300
1200
1100
1000
900
800
700
600
500
400
300
200
100
0
0 -10 -20 -30 -40 -50 -60 -70 -80 -90 -100 -110 -120 -130 -140 -150 -160 -170 -180 -190 -200 -210 -220
f1 (ppm)

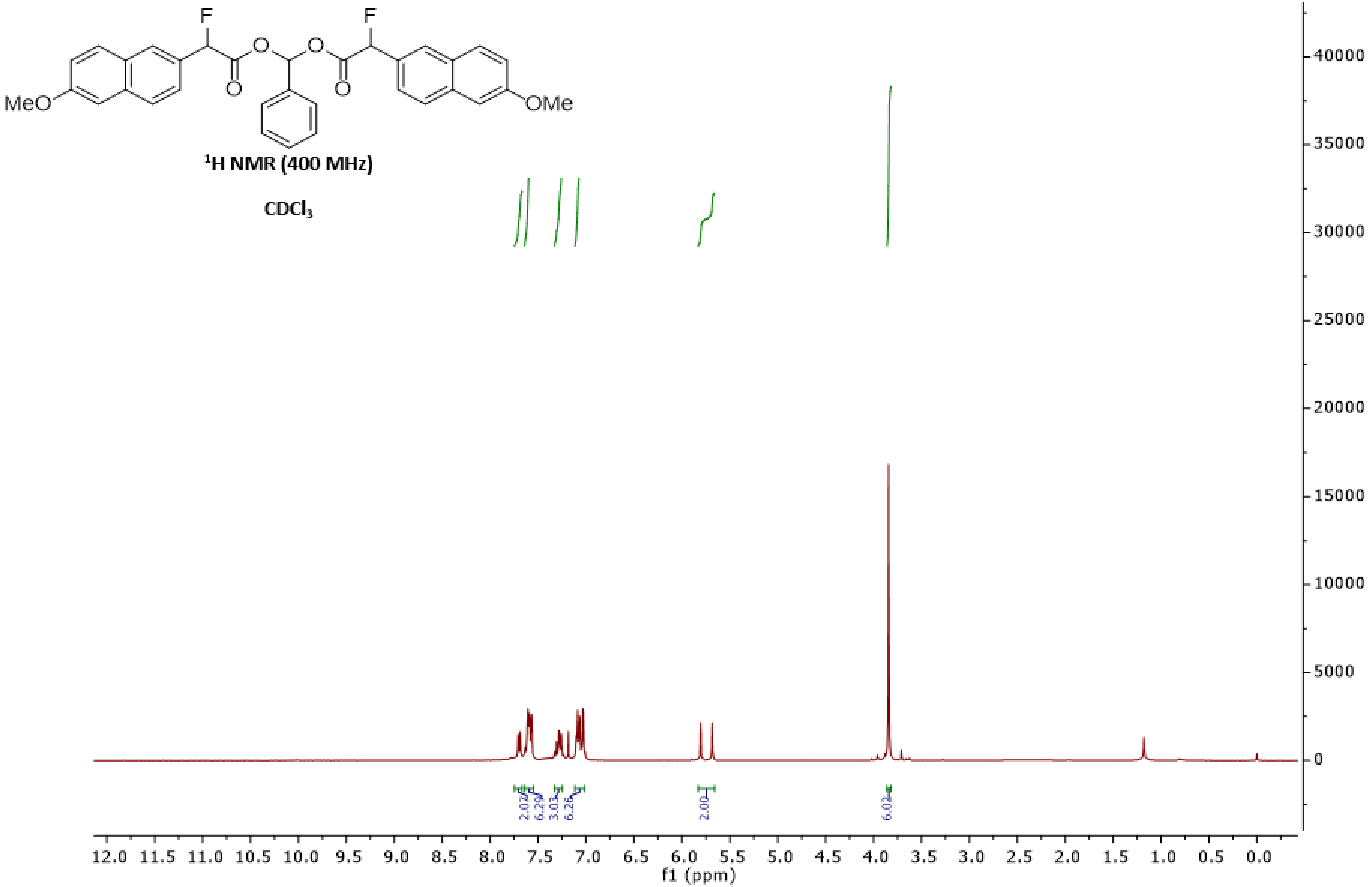
F
F
MeO
OMe
O
O
O
O
¹H NMR (400 MHz)
CDCl3
40000
35000
30000
25000
20000
15000
10000
5000
0
2.02
6.29
3.03
6.26
2.00
6.02
12.0 11.5 11.0 10.5 10.0 9.5 9.0 8.5 8.0 7.5 7.0 6.5 6.0 5.5 5.0 4.5 4.0 3.5 3.0 2.5 2.0 1.5 1.0 0.5 0.0
f1 (ppm)

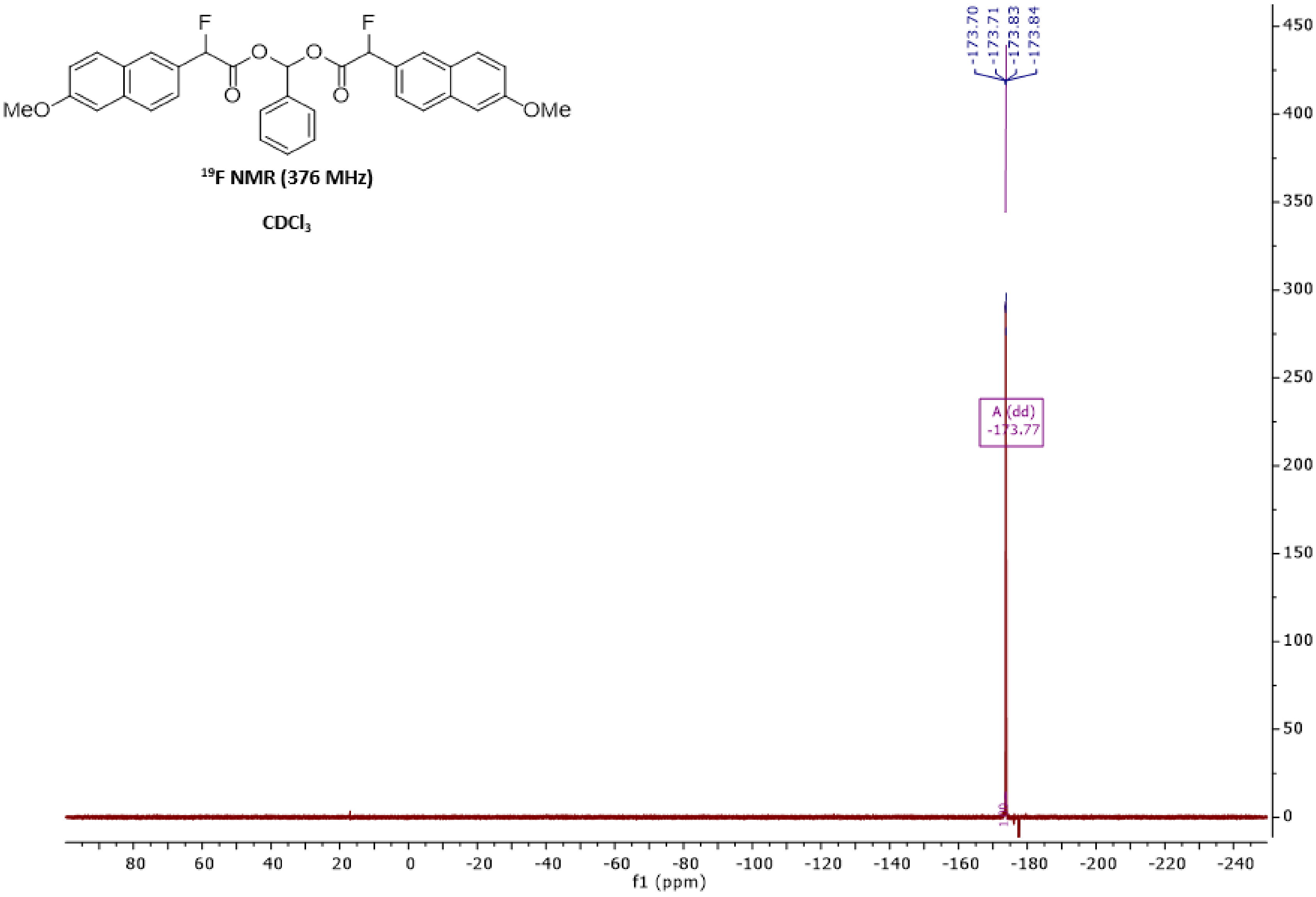


$^{19}$F NMR (376 MHz)

$CDCl_3$

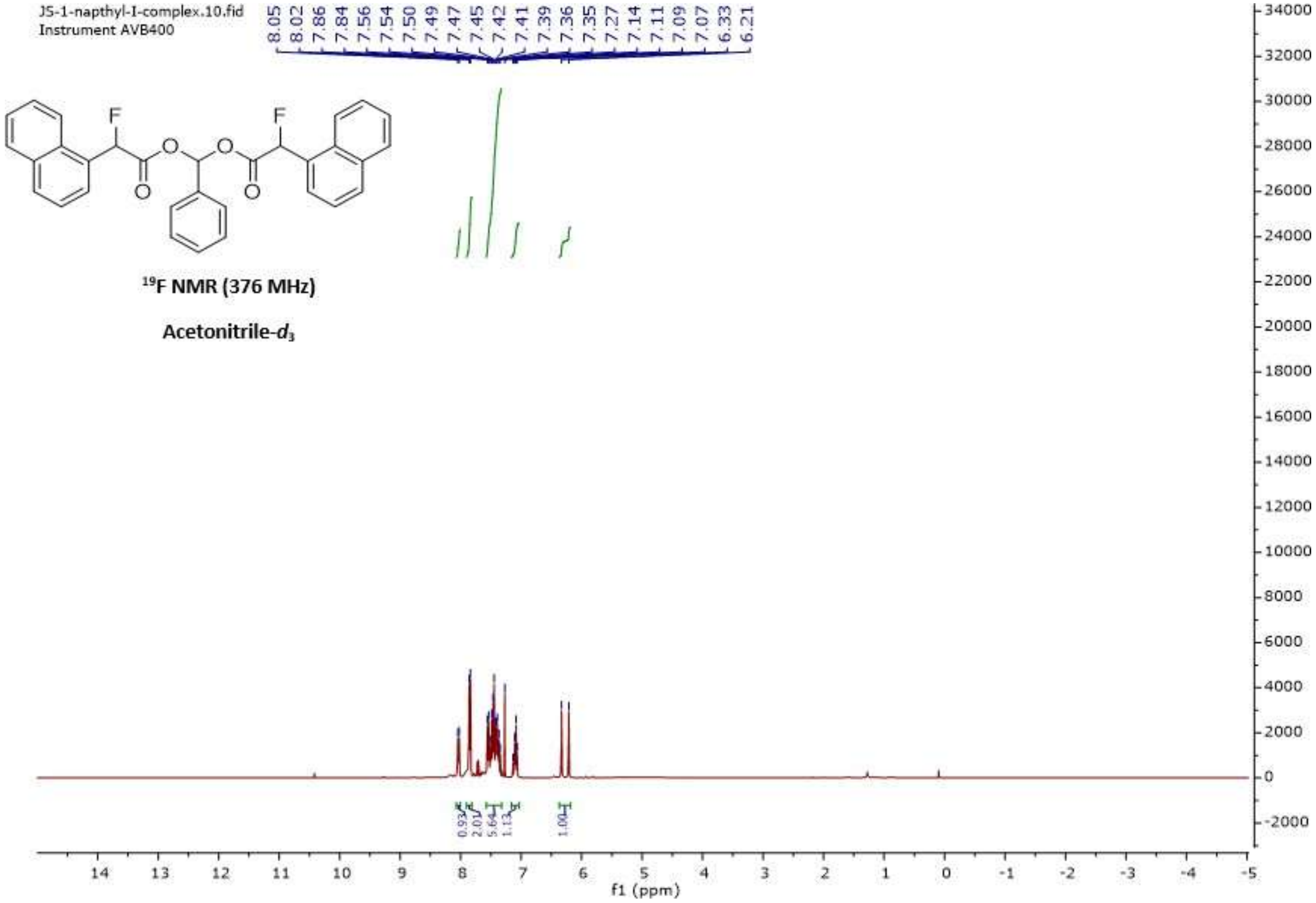
JS-1-napthyl-I-complex.10.fid
Instrument AVB400
8.05
8.02
7.86
7.84
7.56
7.54
7.50
7.49
7.47
7.45
7.42
7.41
7.39
7.36
7.35
7.27
7.14
7.11
7.09
7.07
6.33
6.21
F
F
O
O
O
O
19F NMR (376 MHz)
Acetonitrile-d3
0.93
2.01
5.64
1.13
1.00
f1 (ppm)

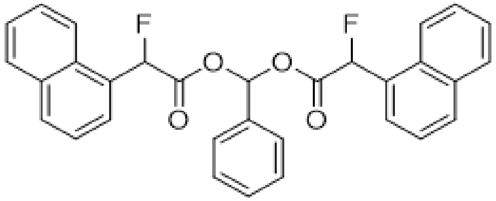

$^{19}$F NMR (376 MHz)

Acetonitrile-$d_3$

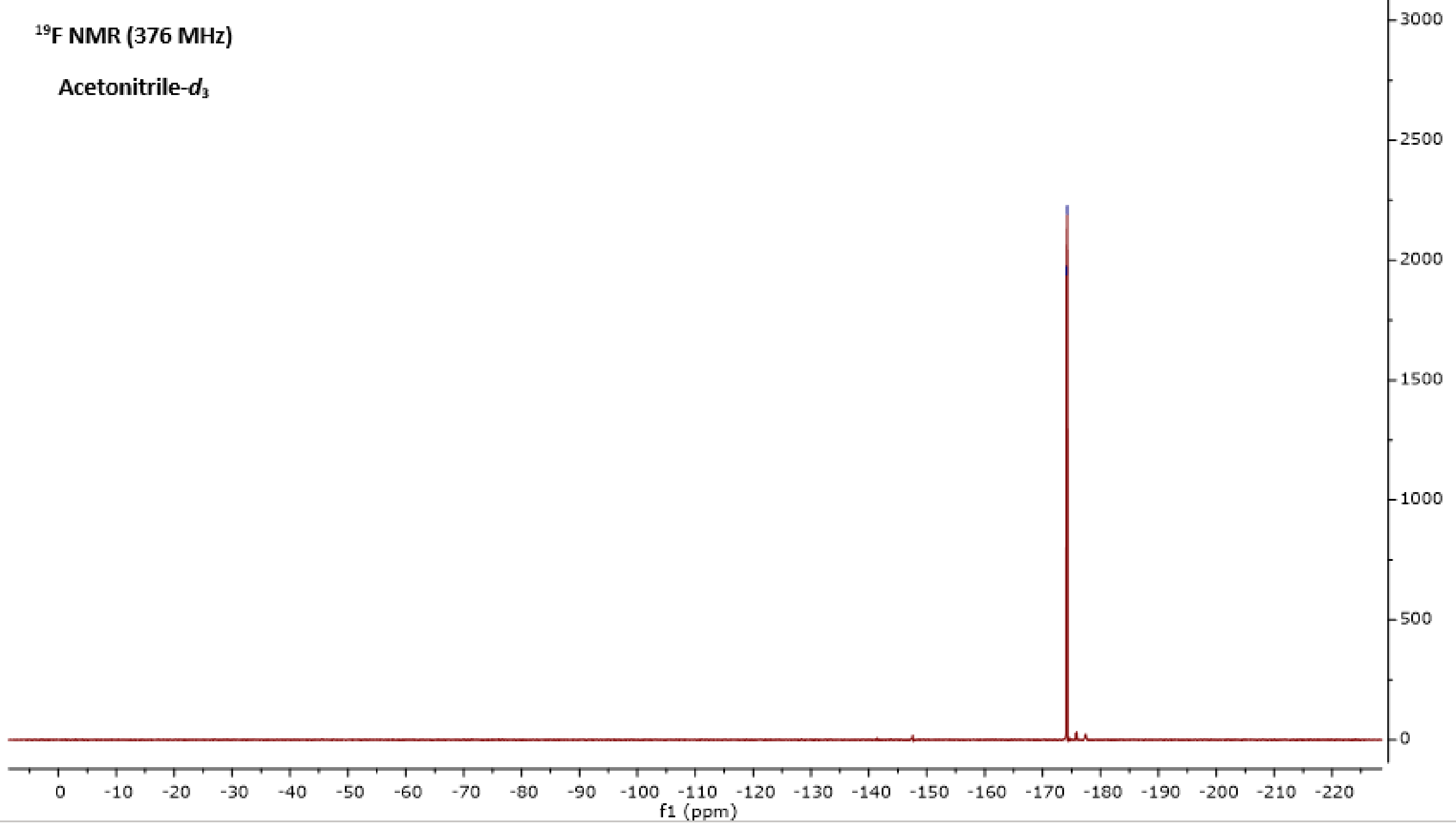

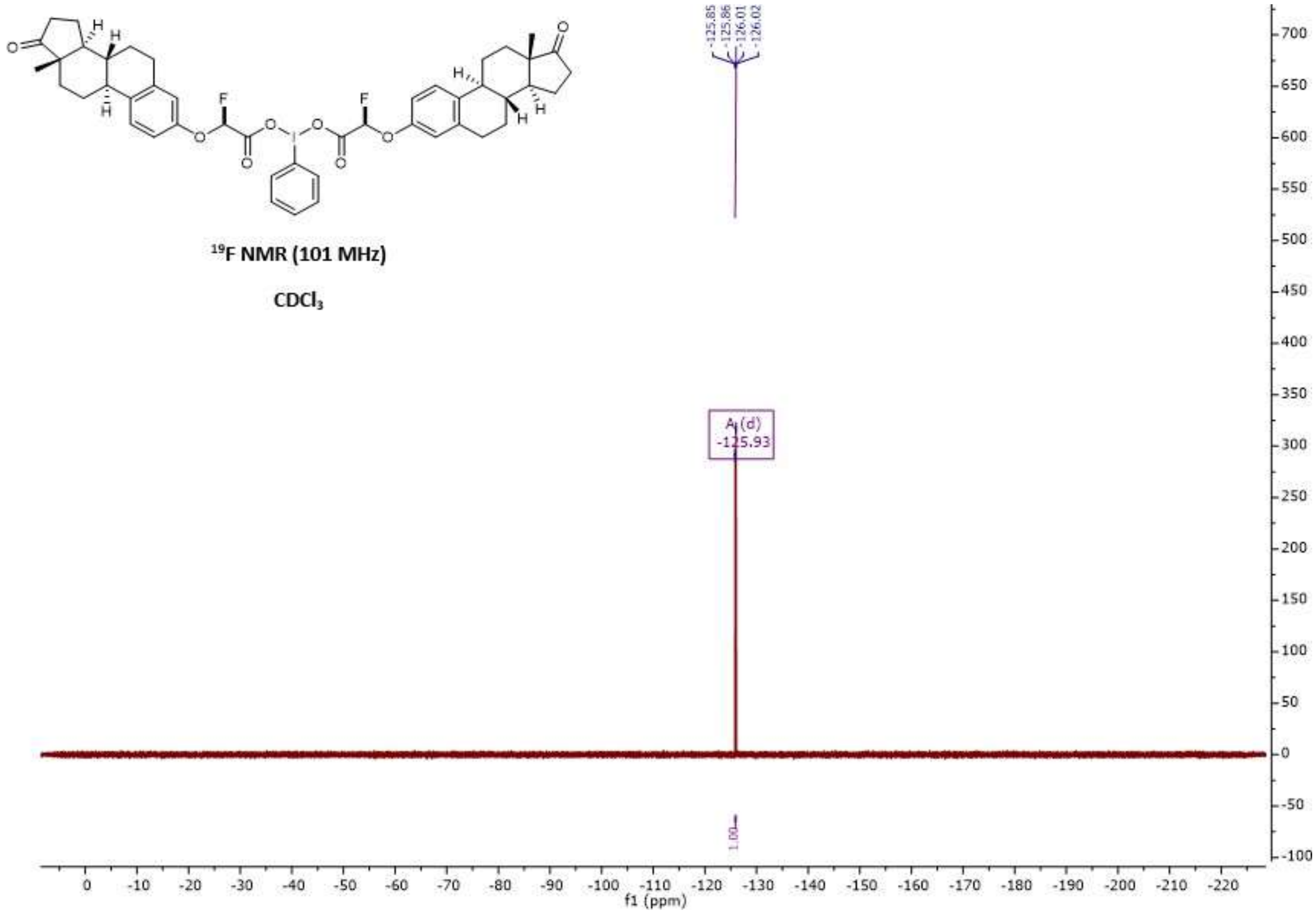


$^{19}$F NMR (101 MHz)
CDCl$_3$
-125.85
-125.86
-126.01
-126.02
A (d)
-125.93
1.00
f1 (ppm)

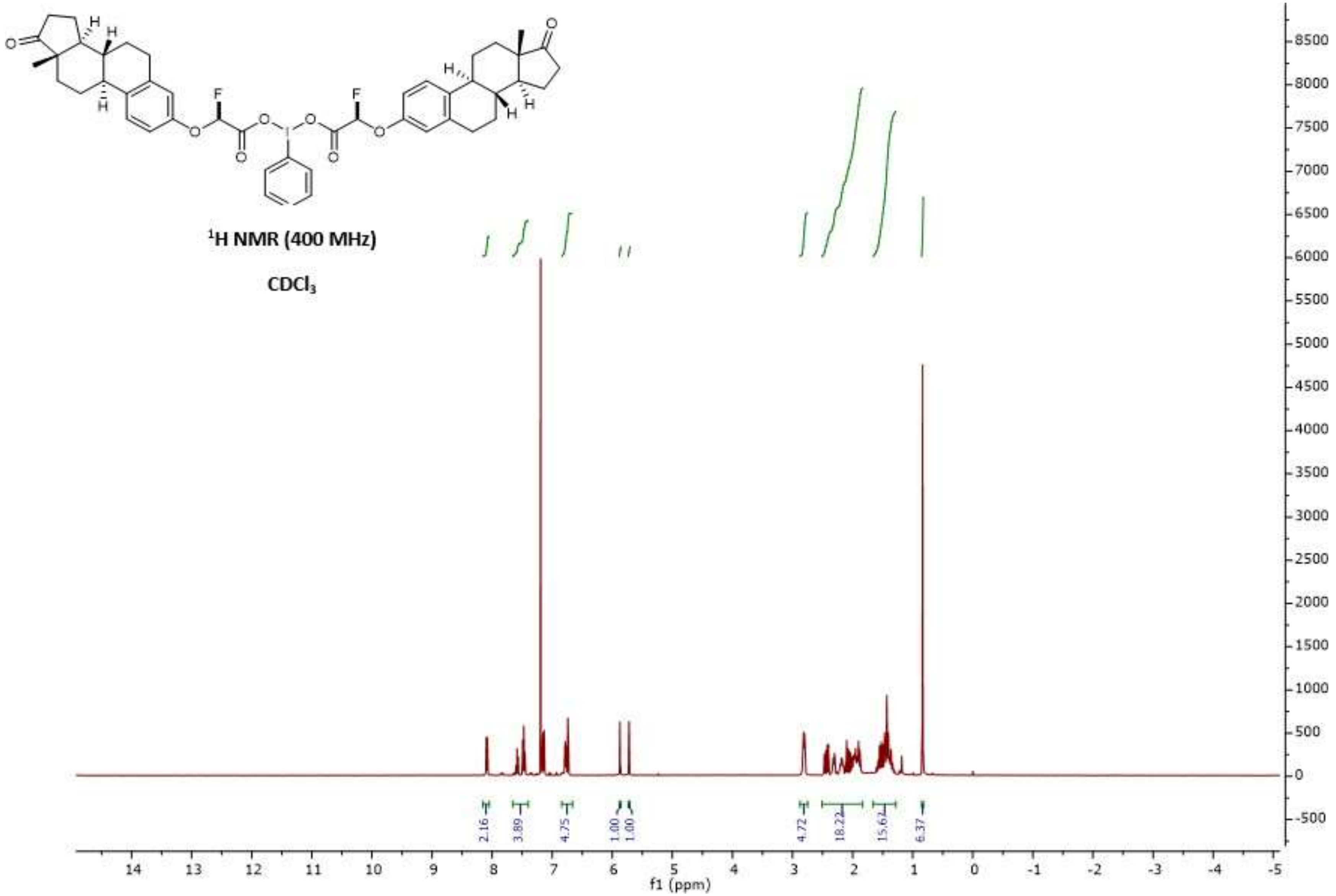


**$^1$H NMR (400 MHz)**

**$CDCl_3$**
